# Highly Regioselective Addition of Allylic Zinc Halides and Various Zinc Enolates to [1.1.1]Propellane

**DOI:** 10.1002/anie.202009340

**Published:** 2020-08-31

**Authors:** Kuno Schwärzer, Hendrik Zipse, Konstantin Karaghiosoff, Paul Knochel

**Affiliations:** ^1^ Department Chemie Ludwig-Maximilians-Universität München Butenandtstraße 5–13, Haus F 81377 München Germany

**Keywords:** [1.1.1]propellane, allylic zinc halides, strained molecules, zinc enolates

## Abstract

We report a range of highly regioselective openings of [1.1.1]propellane with various allylic zinc halides, as well as zinc enolates of ketones, esters and nitriles. The resulting zincated bicyclopentanes (BCPs) were trapped with a range of electrophiles including acyl chlorides, sulfonothioates, hydroxylamino benzoates, tosyl cyanide as well as aryl and allyl halides, generating highly functionalized BCP‐derivatives. The unusually high regioselectivity of these reactions has been rationalized using DFT calculations. A bioisostere of the synthetic opioid pethidine was prepared in 95 % yield in one step using this method.

## Graphical abstract

A wide range of allylic zinc reagents as well as zinc enolates open [1.1.1]propellane to generate zincated bicyclopentanes (BCPs) in a highly regioselective manner. These intermediate zinc reagents are trapped with various electrophiles, giving access to fully functionalized BCPs including a BCP‐analogue of the synthetic opioid pethidine.

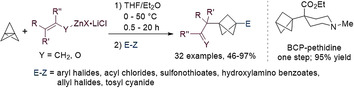

## Introduction

**Scheme 1:**
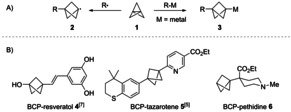
A) Reaction of [1.1.1]propellane (**1**) with radicals and anions. B) BCP‐Analogues of some selected pharmaceutical compounds.

[1.1.1]Propellane (**1**) features an unusual inverted carbon‐carbon bond which results in a unique reactivity. The exact nature of this C_1_−C_3_ bridgehead bond is still being investigated^[1]^ and recent studies by Shaik and co‐workers have described it as a charge‐shift bond.^[2]^ A consequence of this unusual bonding situation is the high reactivity of [1.1.1]propellane (**1**) towards radicals and anions, including polar organometallics. This unique reactivity behaviour gives access to bicyclopentane (BCP)‐radicals (**2**) and ‐organometallics (**3**, Scheme 1),[^1e^, ^3^] which can be further functionalized. The resulting functionalized BCPs are promising bioisosteres for aryl,^[4]^ alkynyl^[5]^ and *tert*‐butyl^[6]^ groups in medicinal chemistry. For example, the substitution of a phenyl group in resveratol with a BCP unit (**4**) as reported by Adsool^[7]^ led to superior in vivo pharmacokinetic properties resulting in an improved bioavailability. Recently, we have reported the replacement of an internal alkyne in tazarotene by a BCP‐unit (**5**), which resulted in a slightly increased basicity without significantly changing the non‐specific binding.^[5]^ As an extension to the previously reported applications of BCPs as bioisosteres we envisioned to prepare the BCP‐analogue of the synthetic opioid pethidine (**6**).^[8]^ In addition to their applications in medicinal chemistry, BCPs have also been utilized as rigid‐linear linkers in rods, liquid crystals, molecular rotors and polymers.^[9]^

**Scheme 2:**
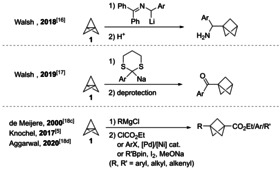
Reactions of [1.1.1]propellane (**1**) with organometallic reagents.

A variety of methods to convert [1.1.1]propellane into functionalized BCPs have been developed, including the “strain release” amination by Baran^[10]^ and Gleason,^[11]^ as well as the radical additions by Wiberg,^[3]^ Uchiyama,^[12]^ Anderson,^[13]^ Bräse^[14]^ and Leonori.^[15]^ The addition of organometallic compounds to [1.1.1]propellane has been achieved using 2‐azaallyllithiums,^[16]^ sodium 2‐aryl‐1,3‐dithiyl anions^[17]^ as well as aryl, alkyl and alkenyl Grignard reagents[^5^, ^18^] (Scheme 2).

**Scheme 3:**
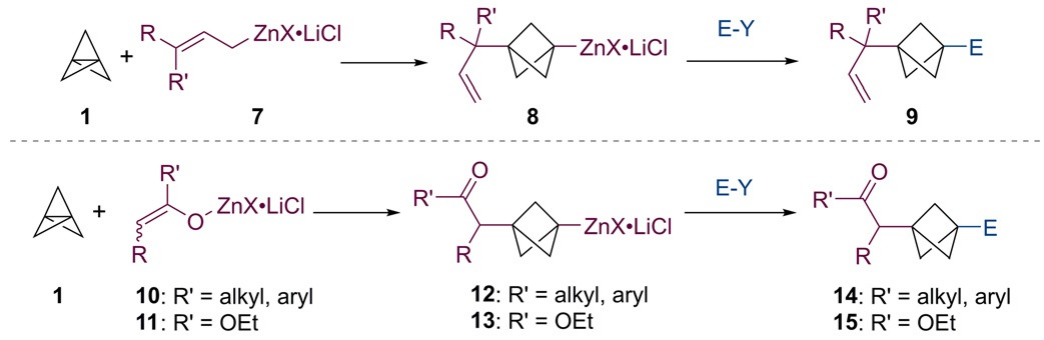
Addition of allylic zinc halides (**7**) and ketone and ester zinc enolates (**10**, **11**) to [1.1.1]propellane (**1**), followed by trapping with electrophiles (E‐Y).

The addition of organozinc halides to [1.1.1]propellane (**1**) has not yet been reported. Indeed, we have observed that alkyl‐, aryl‐ and benzylzinc halides were not able to react with [1.1.1]propellane (**1**) even under harsh conditions (100 °C, up to 60 h). Since allylic zinc halides (**7**) display an enhanced reactivity due to a more polar carbon‐zinc bond,^[19]^ we envisioned that they could add to [1.1.1]propellane (**1**), allowing the formation of zincated BCPs of type **8** (Scheme 3). Subsequent trapping with various electrophiles (E‐Y) would then provide double functionalized BCPs of type **9**. A similar reactivity would be expected for zinc enolates generated from ketones (**10**) and esters (**11**),^[20]^ leading to zincated BCPs of type **12** or **13**, which after electrophilic trapping would generate functionalized BCPs of type **14** and **15**. Herein, we report these highly regioselective reactions, as well as a theoretical rationalization of their high selectivity and a straightforward one step synthesis of the BCP‐bioisostere **6** of the synthetic opioid pethidine (Scheme 1).

## Results and Discussion

**Scheme 4:**
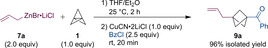
Optimized conditions for the addition of allylzinc bromide complexed with lithium chloride (**7 a**) to [1.1.1]propellane (**1**) followed by copper mediated acylation.

After some experimentation, we have found that the treatment of allylzinc bromide complexed with lithium chloride (**7 a**, 0.90 M in THF, 2.0 equiv)^[21]^ with [1.1.1]propellane (**1**, 0.50 M in Et_2_O, 1.0 equiv) at 25 °C for 2 hours leads to the desired functionalized BCP **9 a** in 96 % yield after a copper mediated acylation with benzoyl chloride (2.5 equiv, Scheme 4). In comparison, the reaction of alkyl‐ and arylmagnesium halides with [1.1.1]propellane (**1**) requires 3–7 days at 25 °C, resulting in moderate yields.[^18a^, ^18b^, ^18c^] A reduction of the amount of zinc reagent **7 a** to 1.5 equivalents lowered the yield to 71 %. Interestingly, the intermediate zincated BCPs of type **8** proved to be very stable, as no significant yield reduction was observed when maintaining the reaction mixture at elevated temperatures for a prolonged time (50 °C, 65 h or 100 °C, 3 h).

**Scheme 5:**
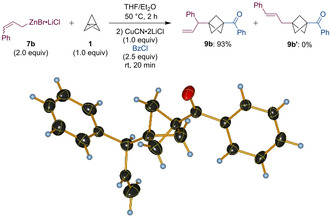
Addition of cinnamyl zinc bromide complexed with lithium chloride (**7 b**) to [1.1.1]propellane (**1**) and X‐ray structure of the resulting product **9 b**.^[22]^

Interestingly, when switching to cinnamylzinc bromide complexed with lithium chloride (**7 b**), only the regioisomer **9 b** was obtained in 93 % yield (Scheme 5). The structure of this product was confirmed via X‐ray analysis^[22]^ and indicates an allylic rearrangement of the organozinc species **7 b** during the reaction. The other regioisomer **9 b′** with the phenyl group attached to the terminal position of the allylic system was not observed.^[23]^

**Scheme 6:**
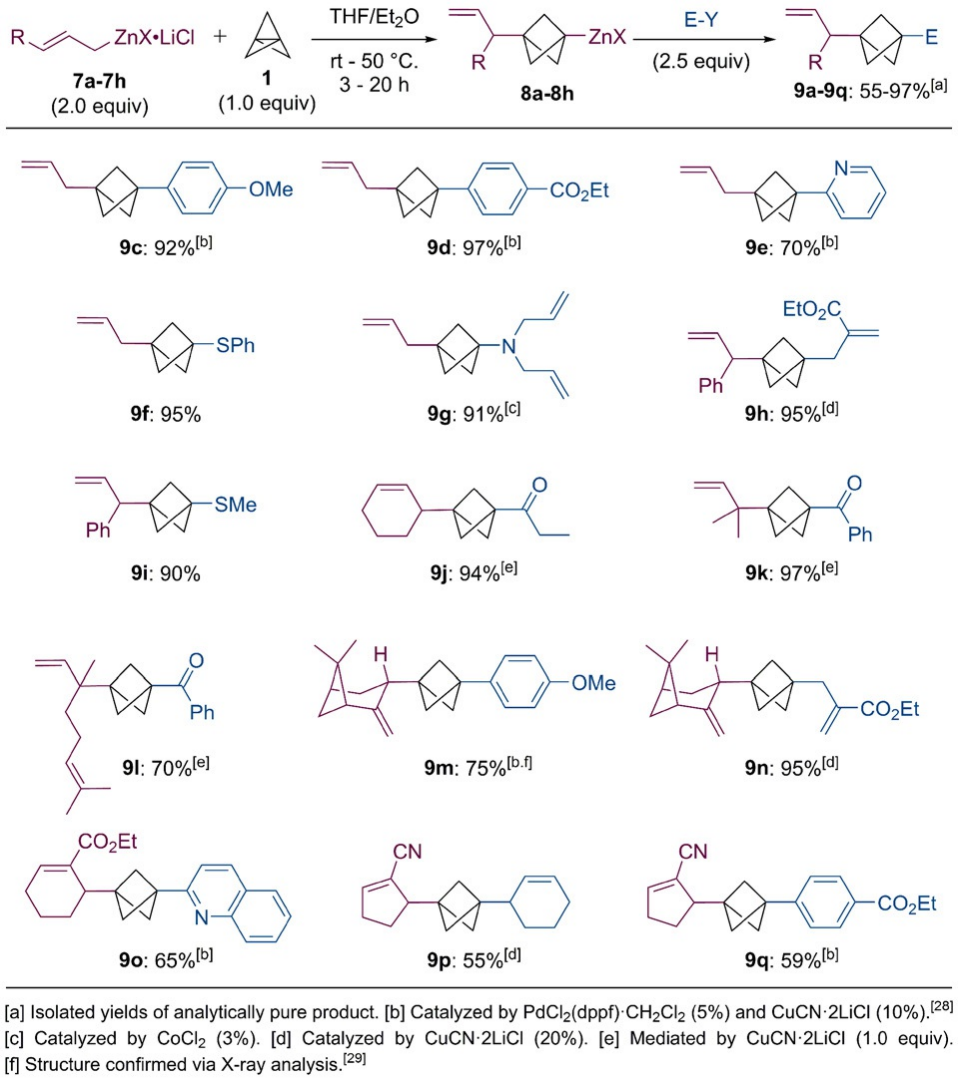
Addition of allylic zinc halides of type **7** to [1.1.1]propellane (**1**) yielding the functionalized BCPs **9 a**–**9 q**.

With this optimized procedure, we have prepared a variety of BCP‐derivatives using allylzinc bromide (**9 a**, **9 c**–**9 g**, Scheme 6), cinnamylzinc bromide (**9 b**, **9 h**, **9 i**) and cyclohex‐2‐en‐1‐ylzinc bromide (**9 j**), as well as allylic zinc reagents derived from terpenoids,^[19]^ such as prenol (**9 k**), geraniol (**9 l**) and (−)‐myrtenol (**9 m**, **9 n**), in 70–97 % yield. In addition, allylic zinc reagents bearing functional groups such as an ester or a nitrile[^19f^, ^24^] reacted smoothly, leading to the corresponding functionalized BCPs **9 o**–**9 q** in 55–65 % yield. In all cases, only a single regioisomer of the product was observed. The intermediate zincated BCPs of type **8** were successfully trapped using *Negishi* cross‐couplings with electron‐rich (**9 c**, **9 m**), electron‐deficient (**9 d**, **9 q**) and heterocyclic (**9 e**, **9 o**) halides in 59–97 % yield, as well as thiolations with *S*‐aryl (**9 f**) and *S*‐alkyl sulfonothioates (**9 i**)^[25]^ in 90–95 % yield. A cobalt‐catalyzed electrophilic amination with *N*,*N*‐diallyl‐*O*‐benzoylhydroxylamine^[26]^ provided the aminated BCP **9 g** in 91 % yield. Finally, the zincated BCPs of type **8** underwent copper‐mediated allylations and acylations using various allylic halides and acid chlorides,^[27]^ leading to the functionalized BCPs **9 a**, **9 b**, **9 h**, **9 j**, **9 k**, **9 l**, **9 n** and **9 p** in 55–97 % yield.

**Scheme 7:**
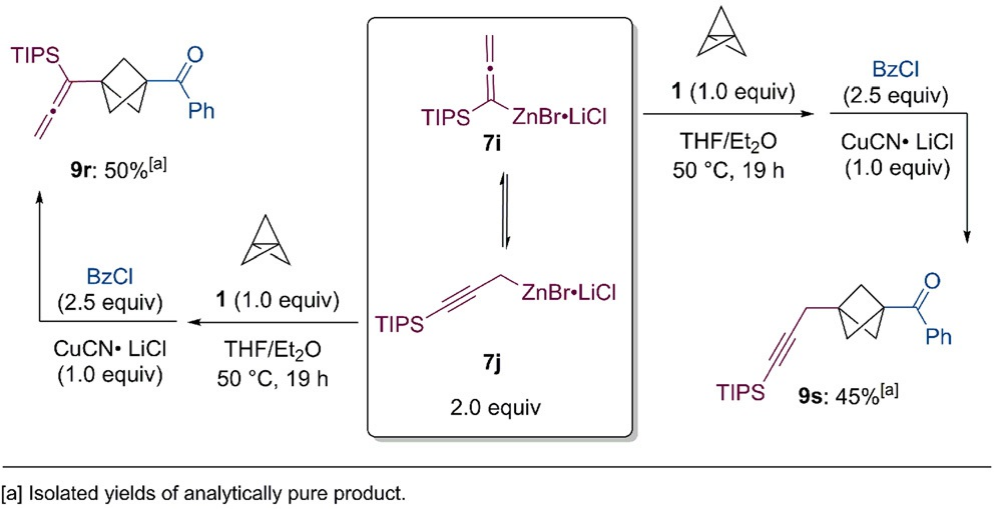
Addition of an equilibrium mixture of the allenic zinc reagent **7 i** and the propargylic zinc reagent **7 j** to [1.1.1]propellane (**1**) yielding the functionalized BCPs **9 r** and **9 s**.

When employing the allenic zinc reagent **7 i**, which is in equilibrium with the propargylic zinc reagent **7 j**,^[30]^ a separable mixture of the allenic product **9 r** (50 % yield) and the propargylic product **9 s** (45 % yield) was obtained after a copper‐mediated acylation with benzoyl chloride (2.5 equiv, Scheme 7). This leads to the assumption, that both of the isomeric zinc reagents **7 i** and **7 j** react with [1.1.1]propellane (**1**) with similar reaction rates.

**Scheme 8:**
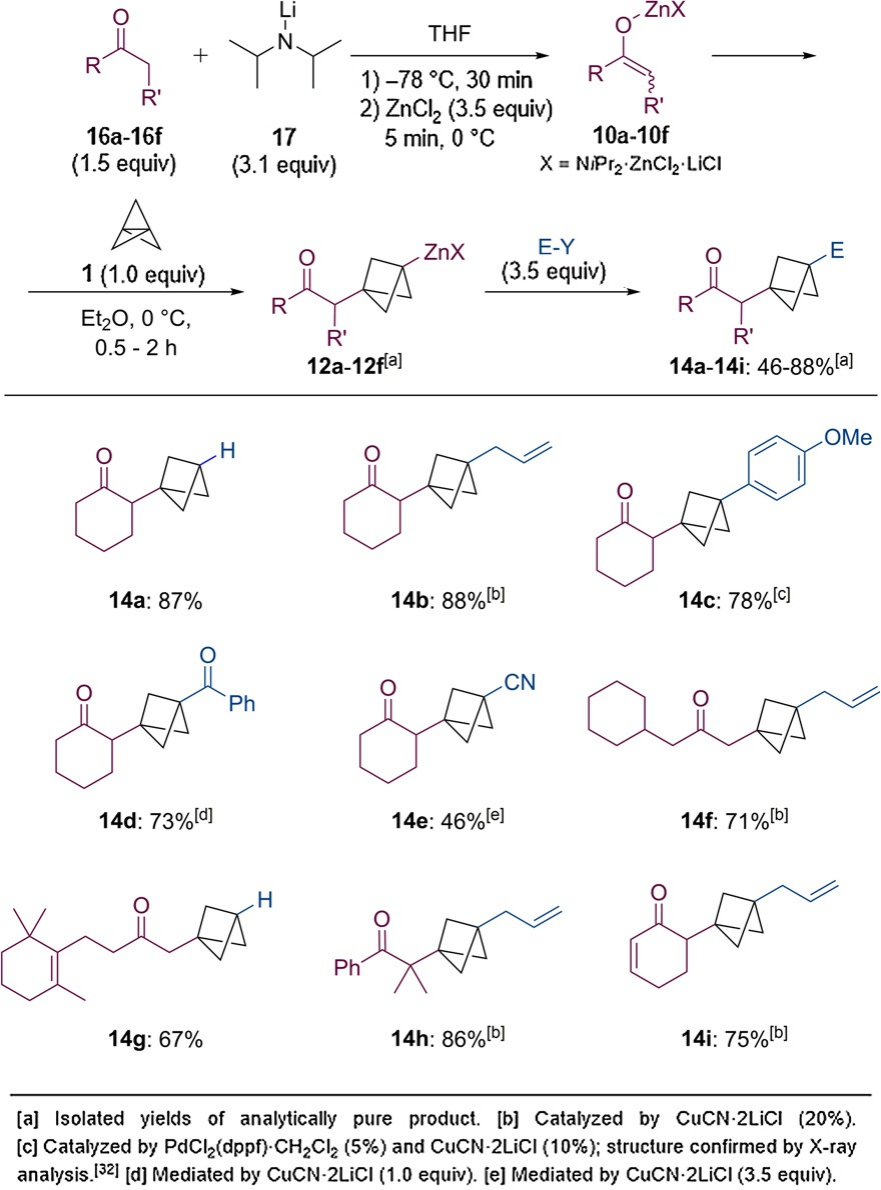
Addition of zinc enolates of type **10** to [1.1.1]propellane (**1**) yielding the functionalized BCPs **14 a**–**14 i** after electrophile trapping.

Next, we turned our attention to zinc enolates. Initially, we treated ketones of type **16** with an equimolar amount of LDA (**17**) at −78 °C, followed by the same amount of ZnCl_2_. The resulting zinc enolates added smoothly to [1.1.1]propellane (**1**, 0.5–2 h, 0 °C, Scheme 8). However, the newly generated zincated BCPs were mostly protonated before they could be trapped with electrophiles, probably due to the competitive deprotonation of the acidic protons in α‐position to the carbonyl group. This problem was solved by using 2 equivalents of LDA for 1 equivalent of the ketone, followed by transmetalation with ZnCl_2_ (2.3 equiv), presumably leading to mixed zinc enolates coordinated with N*i*Pr_2_ (**10**). The zincated BCPs of type **12** that resulted from the addition of these amidozinc enolates to [1.1.1]propellane (**1**, 0.5–2 h, 0 °C, Scheme 8) were apparently much less prone to protonation compared to the standard zincated BCPs.^[30]^ Alternatively, the additional amide might also deprotonate the ketone products, thus removing the acidic protons. Possible trapping reactions included protonation (**14 a**, **14 g**), copper‐catalyzed allylations (**14 b**, **14 f**, **14 h**, **14 i**), a palladium‐catalyzed *Negishi* cross‐coupling (**14 c**), an acylation (**14 d**) and a cyanation performed with tosyl cyanide (**14 e**). The overall yield of the sequence including enolate addition and electrophilic trapping was 46–88 %. In the case of cyclohexyl acetone and dihydro‐β‐ionone a regioselective enolate formation was achieved and only the products **14 f** and **14 g**, in which the BCP unit is attached to the terminal methyl groups, were obtained in 67–71 % yield. Moreover, the sterically hindered isobutyrophenone was added to **1**, leading to the BCP‐derivative **14 h** in 86 % yield. The reaction with cyclohex‐2‐en‐1‐one led to the formation of the expected cyclohexenone derivative **14 i** in 75 % yield.

**Scheme 9:**
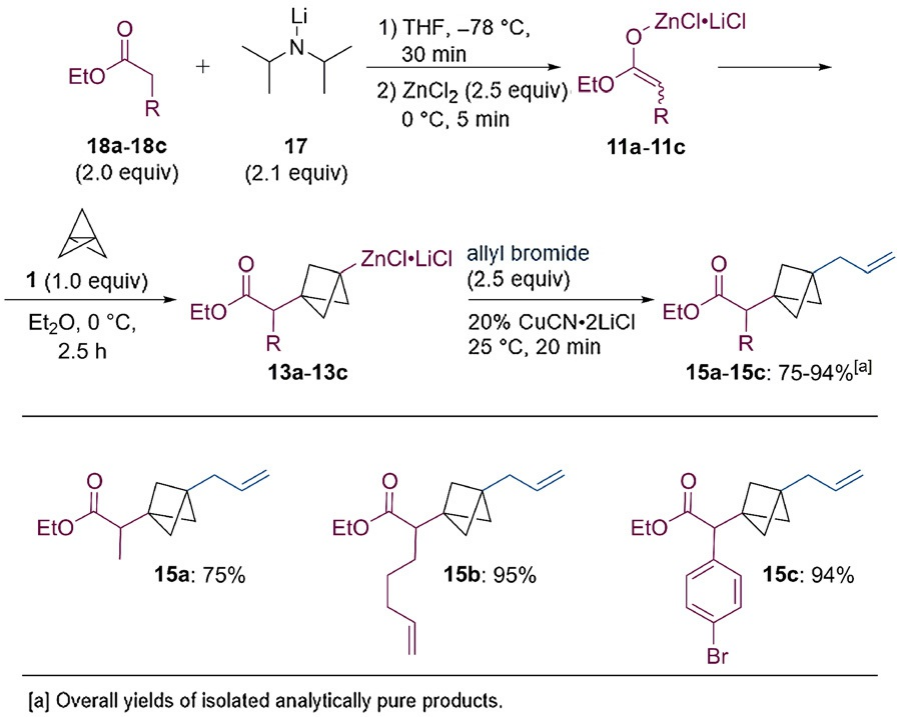
Addition of zinc enolates of type **11** to [1.1.1]propellane (**1**) yielding the functionalized BCPs **15 a**–**15 c**.

When using esters (**18 a**–**18 c**, 2.0 equiv) as starting materials, only a slight excess of LDA (**17**, 2.1 equiv) was necessary to achieve good overall yields (Scheme 9). The zinc enolates of type **11**, obtained after transmetalation with ZnCl_2_ (2.5 equiv), added to [1.1.1]propellane (**1**, 1.0 equiv) within 2.5 h at 25 °C. The resulting zincated BCPs of type **13** were subsequently submitted to a copper‐catalyzed allylation with allyl bromide (2.5 equiv). Thus, ethyl propionate was converted to the BCP **15 a** in 75 % yield. When using ethyl hept‐6‐enoate as a starting material, the expected BCP **15 b** was isolated in 95 % yield without any traces of radical ring‐closure side‐products. The reaction was also compatible with the benzylic ester ethyl 2‐(4‐bromophenyl)acetate, leading to the BCP **15 c** in 94 % yield.

**Scheme 10:**
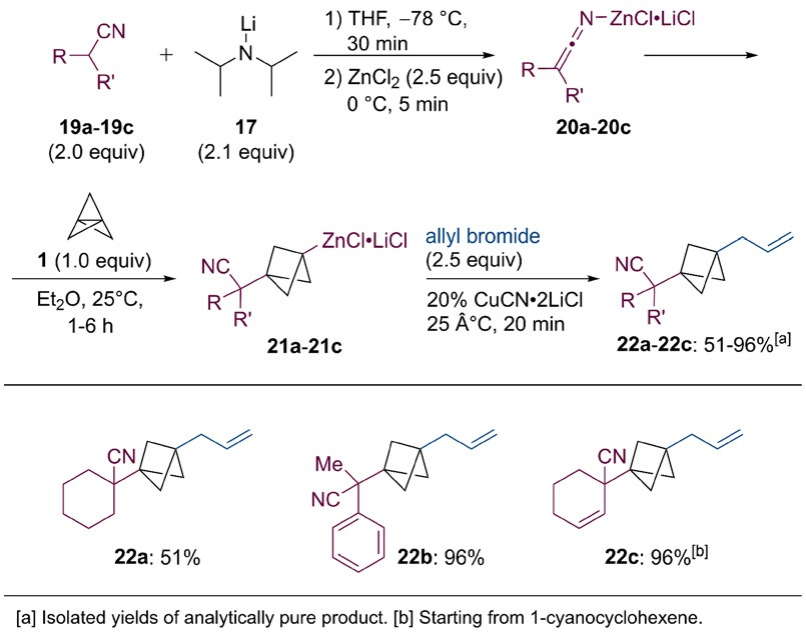
Formation of zincated nitriles of type **20** and their addition to [1.1.1]propellane (**1**) yielding the functionalized BCPs **22 a**–**22 c**.

Finally, the α‐deprotonation of nitriles (**19 a**‐**19 c**, 2.0 equiv) with LDA (**17**, 2.1 equiv) followed by a transmetalation with ZnCl_2_ (2.5 equiv) led to the formation of nitrile‐stabilized carbanions of type **20**,^[33]^ which added to [1.1.1]propellane (**1**) within 1–6 h at 25 °C. The resulting zincated BCPs of type **21** were then submitted to a copper‐catalyzed allylation with allyl bromide (2.5 equiv, Scheme 10). This protocol was used to prepare BCP‐derivatives of cyclohexanecarbonitrile (**22 a**) and 2‐phenylpropanenitrile (**22 b**) in 51–96 % yield. When using 1‐cyanocyclohexene as a starting material, the BCP **22 c** was isolated in 96 % yield. This can be explained due to a rearrangement after the initial deprotonation in the allylic position, resulting in the formation of the most stabilized anion.^[34]^

**Scheme 11:**
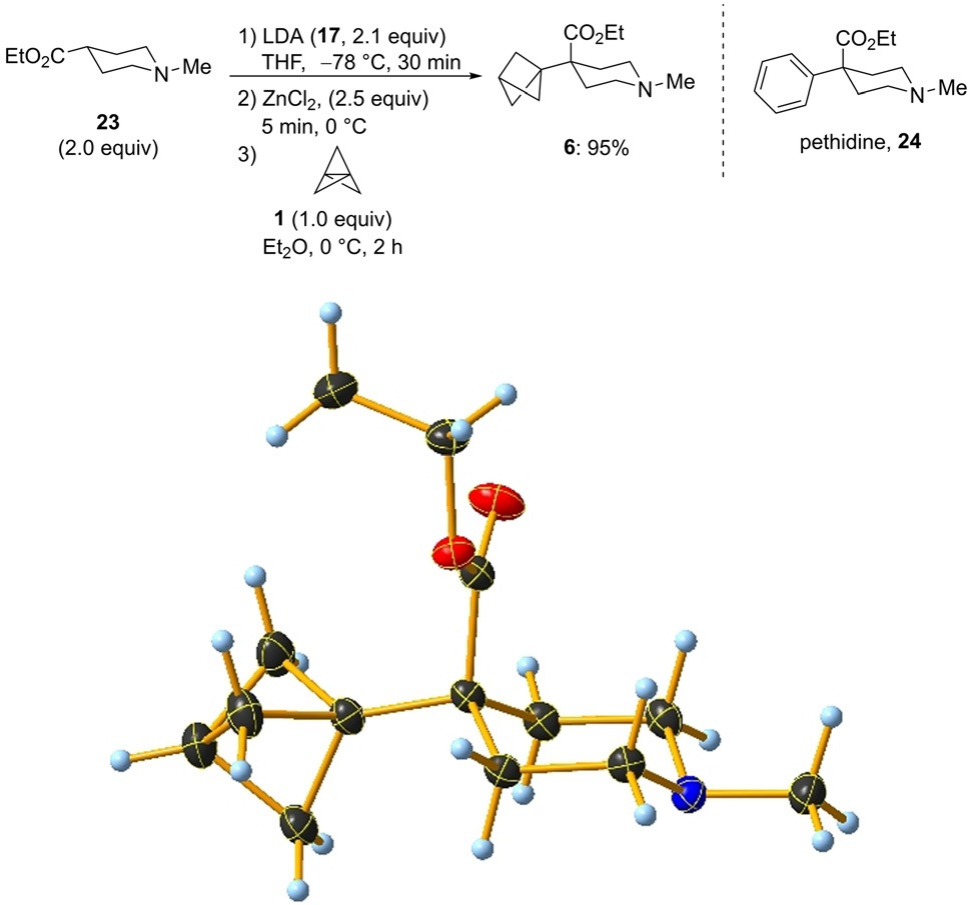
Synthesis and X‐ray structure of BCP‐pethidine **6**.^[35]^

With this optimized procedure, we have carried out the synthesis of the BCP‐analogue of the synthetic opioid pethidine^[8]^ (**24**, Scheme 11). The deprotonation of commercially available ethyl 1‐methylpiperidine‐4‐carboxylate (**23**, 2.0 equiv) with LDA (**17**, 2.1 equiv) proceeded smoothly within 30 min at −78 °C in THF. After the addition of ZnCl_2_ (2.5 equiv) the resulting zinc enolate was reacted with [1.1.1]propellane (**1**, 1.0 equiv) at 0 °C for 2 h. The generated zincated BCP was trapped through the addition of a saturated aqueous solution of NH_4_Cl. The crude mixture was purified using column chromatography, affording the pethidine analogue **6** in 95 % yield on a 1.5 mmol scale in a single step. The structure of the isolated product was confirmed by X‐ray analysis.^[35]^

**Figure 1:**
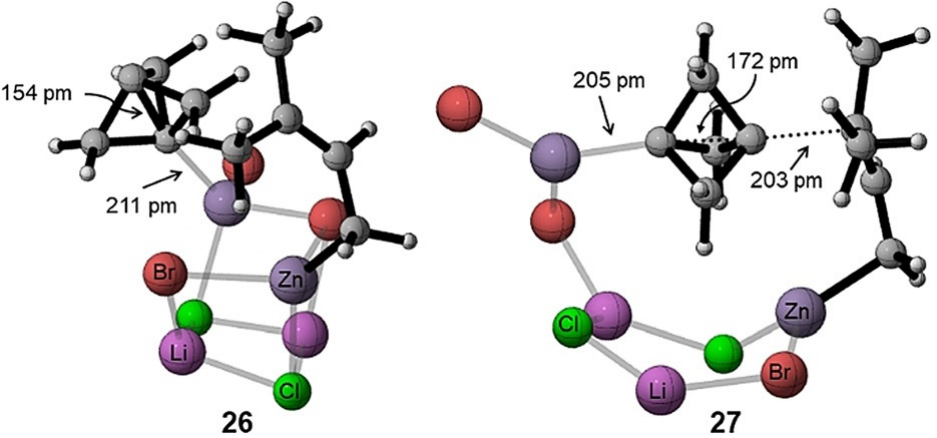
Calculated geometries of [1.1.1]propellane coordinated to a cubic cluster containing prenylzinc bromide (**26**) and the transition state **27**.

**Scheme 12:**
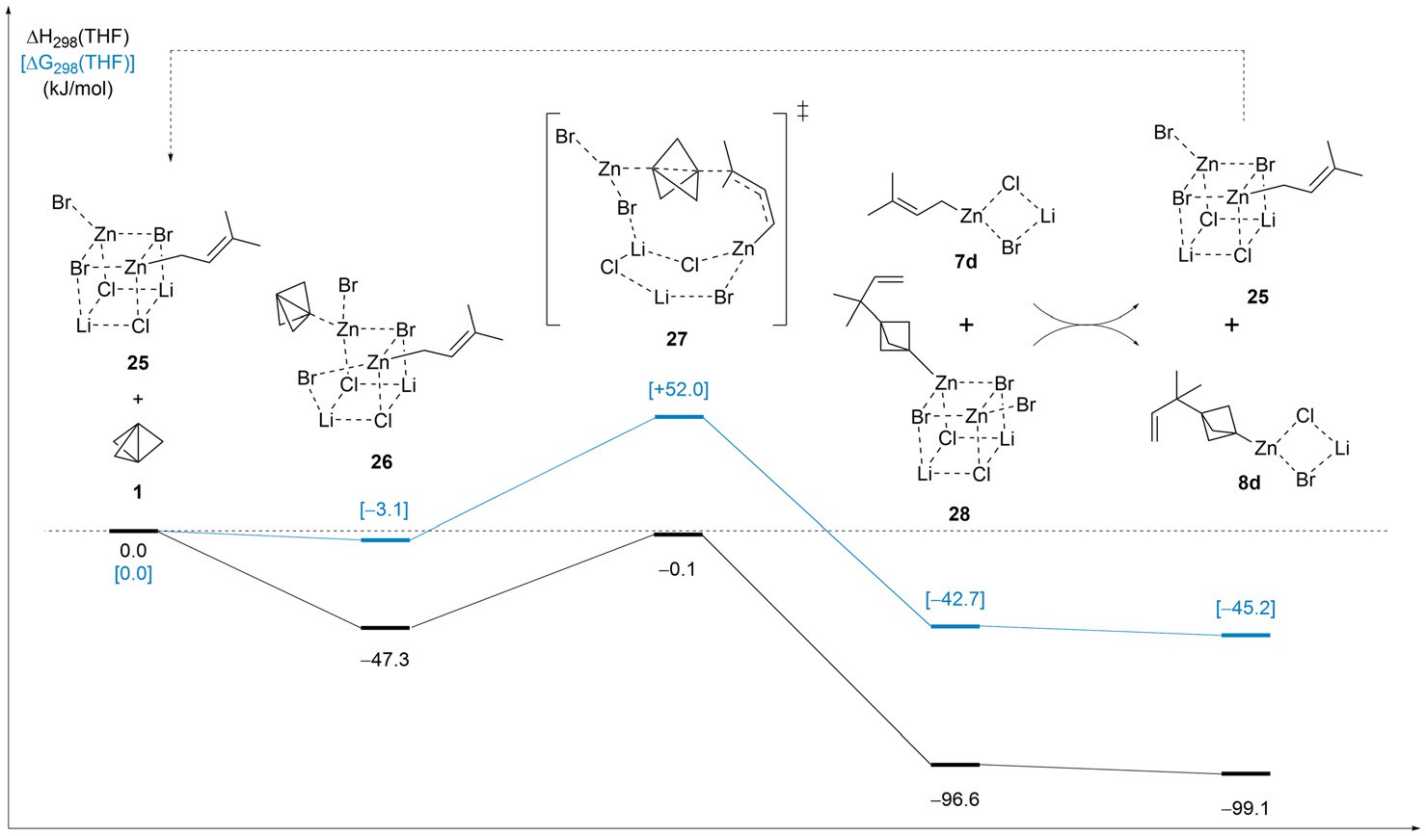
Calculated mechanism for the reaction of a cubic cluster containing prenylzinc bromide (**25**) with [1.1.1]propellane (**1**) (SMD(THF)/B2PLYP‐D3/def2TZVPP//B3LYP‐D3/def2SVP).

In order to rationalize the exquisite regioselectivity observed in transformations with unsymmetrically substituted allylic zinc species, theoretical calculations have been performed for the reaction of propellane (**1**) with prenylzinc bromide complexed with lithium chloride (**7 d**), whose sole reaction product is the BCP **9 k** (Scheme 6). Following effectively the same protocol used in earlier studies of organozinc reagents,^[36]^ free energies in THF solution have been calculated at the SMD(THF)/B2PLYP/def2‐TZVPP level of theory (see SI for further details). Calculations start from the cubic cluster **25** assembled from 2 equivalents of LiCl, ZnBr_2_,^[37]^ and prenylzinc bromide (**7 k**). Complexation of propellane to one of the zinc centres in **25** displaces one of the cluster bromide atoms and generates the adduct **26** in a mildly exergonic first step. Formation of the adduct **26** is accompanied by a minor degree of charge transfer from the propellane unit to the cluster **25** by 0.11e, but leads to practically no change of the propellane structure itself.^[38]^ Backside attack of the prenyl side chain at the bound propellane unit through the transition state **27** then carries the system over to the product side, whose ultimate end point is the cubic cluster **28** located −42.7 kJ mol^−1^ lower than the separate reactants (for additional transient intermediates on the way to the product **28** see SI). That the barrier for this reaction amounts to only 52 kJ mol^−1^ demonstrates the intrinsic flexibility of the salt cluster that thus acts as a template for the reacting organozinc/propellane units. As can readily be seen from the 3D presentation of the transition state **27** in Figure 1, formation of the C−Zn bond is almost complete at *r*(C‐Zn)=205 pm, while formation of the C−C bond between the propellane and prenyl units is still underway with *r*(C−C)=203 pm. From the almost perfect alignment of both reacting bonds along the propellane axis it is also apparent, that the cluster template exerts practically no external strain onto the reacting fragments. The overall reaction cycle is completed by salt metathesis of the product cluster **28** with one equivalent of the prenylzinc reagent **7 d**, yielding the starting cluster **25** and the product organozinc species **8 d** (Scheme 12). This reaction is almost thermoneutral at Δ*G*_298_=−2.5 kJ mol^−1^. The activation of propellane (**1**) by other cluster designs or through other activation modes has also been studied, but all of these variations are energetically less favourable than the reaction pathway shown in Scheme 12 (see SI). This includes the reaction with a cluster containing the regioisomeric form of prenylzinc bromide, in which the zinc is located at the tertiary carbon atom. For this cluster the calculated reaction barrier was 43.6 kJ mol^−1^ higher than the one detailed in Scheme 12. Therefore, the high regioselectivity can be attributed to a kinetic selectivity. Good energetics have been found for the reaction of propellane with a radical version of the cluster **25** (lacking a bromine atom). However, this reaction pathway suffers from the lack of a well‐defined step for the initial radical formation. A reaction pathway starting with the coordination of [1.1.1]propellane to lithium instead of zinc resulted in a reaction barrier that was 82.5 kJ mol^−1^ higher than the one detailed in Scheme 12.

## Conclusion

We have reported a range of new regioselective openings of [1.1.1]propellane (**1**) with various allylic zinc halides, as well as zinc enolates of ketones, esters and nitriles. The resulting zincated BCPs were trapped with a range of electrophiles, including acyl chlorides, aryl and allyl halides as well as sulfonothioates, hydroxylamino benzoates and tosyl cyanide, generating new highly functionalized BCP derivatives. Remarkably, the opening of [1.1.1]propellane (**1**) with unsymmetrical allylic zinc reagents proceeds via a complete allylic rearrangement. This behaviour was rationalized by DFT‐calculations, which showed that the allylic rearrangement proceeds via a cyclic transition state (**27**) involving ZnBr_2_, LiCl, the allylic zinc halide and [1.1.1]propellane. Furthermore, we have demonstrated the utility of our procedure by preparing a bioisostere of the synthetic opioid pethidine in 95 % yield in a single step.

## Conflict of interest

The authors declare no conflict of interest.

## Supporting information

As a service to our authors and readers, this journal provides supporting information supplied by the authors. Such materials are peer reviewed and may be re‐organized for online delivery, but are not copy‐edited or typeset. Technical support issues arising from supporting information (other than missing files) should be addressed to the authors.

Supplementary

## References

[1a] Y. Yang , J. Phys. Chem. A 2012, 116, 10150–10159; 23009383 10.1021/jp304420c

[1b] M. Bremer , H. Untenecker , P. A. Gunchenko , A. A. Fokin , P. R. Schreiner , J. Org. Chem. 2015, 80, 6520–6524; 26011255 10.1021/acs.joc.5b00845

[1c] J. Joy , E. Akhil , E. D. Jemmis , Phys. Chem. Chem. Phys. 2018, 20, 25792–25798; 30283928 10.1039/c8cp05125a

[1d] R. Wang , S. Yang , Q. Li , Molecules 2019, 24, 2601–2611;

[1e] A. J. Sterling , A. B. Dürr , R. C. Smith , E. A. Anderson , F. Duarte , Chem. Sci. 2020, 11, 4895–4903. 10.1039/d0sc01386b PMC8159217 34122945

[2a] S. Shaik , D. Danovich , W. Wu , P. C. Hiberty , Nat. Chem. 2009, 1, 443–449; 21378912 10.1038/nchem.327

[2b] W. Wu , J. Gu , J. Song , S. Shaik , P. C. Hiberty , Angew. Chem. Int. Ed. 2009, 48, 1407–1410; 10.1002/anie.200804965 19072971

[3] K. B. Wiberg , S. T. Waddell , J. Am. Chem. Soc. 1990, 112, 2194–2216.

[4] Y. P. Auberson , C. Brocklehurst , M. Furegati , T. C. Fessard , G. Koch , A. Decker , L. La Vecchia , E. Briard , ChemMedChem 2017, 12, 590–598. 28319646 10.1002/cmdc.201700082

[5] I. S. Makarov , C. E. Brocklehurst , K. Karaghiosoff , G. Koch , P. Knochel , Angew. Chem. Int. Ed. 2017, 56, 12774–12777; 10.1002/anie.201706799 28786520

[6a] M. R. Barbachyn , D. K. Hutchinson , D. S. Toops , R. J. Reid , G. E. Zurenko , B. H. Yagi , R. D. Schaadt , J. W. Allison , Bioorg. Med. Chem. Lett. 1993, 3, 671–676;

[6b] M. V. Westphal , B. T. Wolfstädter , J.-M. Plancher , J. Gatfield , E. M. Carreira , ChemMedChem 2015, 10, 461–469. 25630804 10.1002/cmdc.201402502

[7] Y. L. Goh , Y. T. Cui , V. Pendharkar , V. A. Adsool , ACS Med. Chem. Lett. 2017, 8, 516–520. 28523103 10.1021/acsmedchemlett.7b00018 PMC5430408

[8] L. E. Mather , P. J. Meffin , Clin. Pharmacokinet. 1978, 3, 352–368. 359212 10.2165/00003088-197803050-00002

[9a] A. M. Dilmaç , E. Spuling , A. de Meijere , S. Bräse , Angew. Chem. Int. Ed. 2017, 56, 5684–5718; 10.1002/anie.201603951 27905166

[9b] G. M. Locke , S. S. R. Bernhard , M. O. Senge , Chem. Eur. J. 2019, 25, 4590–4647. 30387906 10.1002/chem.201804225

[10a] R. Gianatassio , J. M. Lopchuk , J. Wang , C.-M. Pan , L. R. Malins , L. Prieto , T. A. Brandt , M. R. Collins , G. M. Gallego , N. W. Sach , J. E. Spangler , H. Zhu , J. Zhu , P. S. Barran , Science 2016, 351, 241–246; 26816372 10.1126/science.aad6252 PMC4730898

[10b] J. M. Lopchuk , K. Fjelbye , Y. Kawamata , L. R. Malins , C.-M. Pan , R. Gianatassio , J. Wang , L. Prieto , J. Bradow , T. A. Brandt , M. R. Collins , J. Elleraas , J. Ewanicki , W. Farrell , O. O. Fadeyi , G. M. Gallego , J. J. Mousseau , R. oliver , N. W. Sach , J. K. Smith , J. E. Spangler , H. Zhu , J. Zhu , P. S. Barran , J. Am. Chem. Soc. 2017, 139, 3209–3226. 28140573 10.1021/jacs.6b13229 PMC5334783

[11] J. M. E. Hughes , D. A. Scarlata , A. C.-Y. Chen , J. D. Burch , J. L. Gleason , Org. Lett. 2019, 21, 6800–6804. 31407916 10.1021/acs.orglett.9b02426

[12] J. Kanazawa , K. Maeda , M. Uchiyama , J. Am. Chem. Soc. 2017, 139, 17791–17794. 29131599 10.1021/jacs.7b11865

[13a] D. F. J. Caputo , C. Arroniz , A. B. Dürr , J. J. Mousseau , A. F. Stepan , S. J. Mansfield , E. A. Anderson , Chem. Sci. 2018, 9, 5295–5300; 29997886 10.1039/c8sc01355a PMC6001403

[13b] M. L. J. Wong , J. J. Mousseau , S. J. Mansfield , E. A. Anderson , Org. Lett. 2019, 21, 2408–2411; 30869907 10.1021/acs.orglett.9b00691

[13c] J. Nugent , C. Arroniz , B. R. Shire , A. J. Sterling , H. D. Pickford , M. L. J. Wong , S. J. Mansfield , D. F. J. Caputo , B. Owen , J. J. Mousseau , F. Duarte , E. A. Anderson , ACS Catal. 2019, 9, 9568–9574;

[13d] J. Nugent , B. R. Shire , D. F. J. Caputo , H. D. Pickford , F. Nightingale , I. T. T. Houlsby , J. J. Mousseau , E. A. Anderson , Angew. Chem. Int. Ed. 2020, 59, 11866–11870; 10.1002/anie.202004090 PMC7383991 32346946

[14] R. M. Bär , S. Kirschner , M. Nieger , S. Bräse , Chem. Eur. J. 2018, 24, 1373–1382. 29044719 10.1002/chem.201704105

[15] J. H. Kim , A. Ruffoni , Y. S. S. Al-Faiyz , N. S. Sheikh , D. Leonori , Angew. Chem. Int. Ed. 2020, 59, 8225–8231; 10.1002/anie.202000140 PMC7318212 32003916

[16] R. A. Shelp , P. J. Walsh , Angew. Chem. Int. Ed. 2018, 57, 15857–15861; 10.1002/anie.201810061 30291667

[17] N. Trongsiriwat , Y. Pu , Y. Nieves-Quinones , R. A. Shelp , M. C. Kozlowski , P. J. Walsh , Angew. Chem. Int. Ed. 2019, 58, 13416–13420; 10.1002/anie.201905531 PMC6788743 31291500

[18a] G. Szeimies in Strain and Implications in Organic Chemistry. NATO ASI Series (Series C: Mathematical and Physical Sciences), Vol. 273 (Eds.: de Meijere A., Blechert S.), Springer, Dordrecht, 1989, pp. 361–381;

[18b] S. Guffler, Wege zu 1,3-disubstituierten Bicyclo[1.1.1]pentanen: synthetische und mechanistische Aspekte, Doctoral dissertation, Humboldt-Universität Berlin, **1998**;

[18c] M. Messner , S. I. Kozhushkov , A. de Meijere , Eur. J. Org. Chem. 2000, 1137–1155;

[18d] S. Yu , C. Jing , A. Noble , V. K. Aggarwal , Angew. Chem. Int. Ed. 2020, 59, 3917–3921; 10.1002/anie.201914875 31912941

[18e] S. Yu , C. Jing , A. Noble , V. K. Aggarwal , Org. Lett. 2020, 22, 5650–5655; 32638587 10.1021/acs.orglett.0c02017

[18f] C. Andersen , V. Ferey , M. Daumas , P. Bernardelli , A. Guérinot , J. Cossy , Org. Lett. 2020, 22, 6021–6025. 32672465 10.1021/acs.orglett.0c02115

[19a] G. Courtois , A. Al-Arnaout , L. Miginiac , Tetrahedron Lett. 1985, 26, 1027–1030;

[19b] P. Knochel , R. Singer , Chem. Rev. 1993, 93, 2117–2188;

[19c] M. Nakamura , A. Hirai , M. Sogi , E. Nakamura , J. Am. Chem. Soc. 1998, 120, 5846–5847;

[19d] I. Marek , G. Sklute , Chem. Commun. 2007, 1683–1691; 10.1039/b615042j 17457409

[19e] H. Ren , G. Dunet , P. Mayer , P. Knochel , J. Am. Chem. Soc. 2007, 129, 5376–5377; 17408274 10.1021/ja071380s

[19f] M. Ellwart , P. Knochel , Angew. Chem. Int. Ed. 2015, 54, 10662–10665; 10.1002/anie.201504354 26189654

[20a] J. Dekker , A. Schouten , P. H. M. Budzelaar , J. Boersma , G. J. M. van der Kerk , J. Organomet. Chem. 1987, 320, 1–12;

[20b] G. K. Jarugumilli , C. Zhu , S. P. Cook , Eur. J. Org. Chem. 2012, 1712–1715;

[20c] A. Baba , M. Yasuda , Y. Nishimoto in Comprehensive Organic Synthesis II, Vol. 2 (Eds.: Knochel P., Molander G. A.), Elsevier, Amsterdam, 2014, pp. 523–542.

[21] Without complexed lithium chloride the reaction with [1.1.1]propellane (**1**) resulted in comparable yields. However, the presence of lithium chloride during the insertion of zinc dust into allylic halides resulted in significantly less homocoupling, thus making the preparation of allylic organozinc reagents more convenient. For an overview of the optimization process see the Supporting Information.

[22] Deposition number 2011116 (**9 b**) contains the supplementary crystallographic data for this paper. These data are provided free of charge by the joint Cambridge Crystallographic Data Centre and Fachinformationszentrum Karlsruhe Access Structures service.

[23] A test reaction with prenylmagnesium chloride showed that the reaction of allylic magnesium halides with [1.1.1]propellane (**1**) is also much quicker than the reaction of alkyl or aryl magnesium halides and was completed within 2 h at room temperature. The exclusive formation of the regioisomer that results from an allylic rearrangement was observed. This suggests that the reactions of allylic zinc and magnesium reagents with [1.1.1]propellane proceed via the same mechanism.

[24a] Y. Dembélé , C. Belaud , P. Hitchcock , J. Villiéras , Tetrahedron: Asymmetry 1992, 3, 351–354;

[24b] V. Nyzam , C. Belaud , F. Zammattio , J. Villiéras , Tetrahedron: Asymmetry 1996, 7, 1835–1843;

[24c] H. C. Sämann , P. Knochel , Synthesis 2013, 45, 1870–1876.

[25] K. Fujiki , N. Tanifuji , Y. Sasaki , T. Yokoyama , Synthesis 2002, 3, 343–348.

[26a] Y.-H. Chen , S. Graßl , P. Knochel , Angew. Chem. Int. Ed. 2018, 57, 1108–1111; 10.1002/anie.201710931 29160920

[26b] S. Graßl , Y.-H. Chen , C. Hamze , C. P. Tüllmann , P. Knochel , Org. Lett. 2019, 212, 494–497. 10.1021/acs.orglett.8b03787 30588813

[27] M. C. P. Yeh , P. Knochel , Tetrahedron Lett. 1988, 29, 2395–2396.

[28] Without CuCN⋅2 LiCl as a cocatalyst the yields of the cross-coupling were approximately 50 % lower. CuI was a significantly less effective cocatalyst than CuCN⋅2 LiCl.

[29] Deposition number 2011113 (**9 m**) contains the supplementary crystallographic data for this paper. These data are provided free of charge by the joint Cambridge Crystallographic Data Centre and Fachinformationszentrum Karlsruhe Access Structures service.

[30a] J. A. Marshall , B. W. Gung , M. L. Grachan in Modern Allene Chemistry, Vol. 1 (Eds.: Krause N., Hashmi A. S. K.), Wiley-VCH, Weinheim, 2004, pp. 493–592;

[30b] J. A. Marshall in The Chemistry of Organozinc Compounds, Vol. 1 (Eds.: Rappoprt Z., Marek I.), Wiley, Hoboken, 2006, pp. 421–455;

[30c] D. R. Fandrick , J. Saha , K. R. Fandrick , S. Sanyal , J. Ogikubo , H. Lee , F. Roschangar , J. J. Song , C. H. Senanayake , Org. Lett. 2011, 13, 5616–5619. 21942658 10.1021/ol202343c

[31] The enhanced stability of the zincated BCPs towards protonation in the presence of additional amide is reminiscent to the effect observed for organozinc pivalates. For reference see:

[31a] C. I. Stathakis , S. Bernhardt , V. Quint , P. Knochel , Angew. Chem. Int. Ed. 2012, 51, 9428–9432; 10.1002/anie.201204526 22907878

[31b] A. Hernán-Gómez , E. Herd , E. Hevia , A. R. Kennedy , P. Knochel , K. Koszinowski , S. M. Manolikakes , R. E. Mulvey , C. Schnegelsberg , Angew. Chem. Int. Ed. 2014, 53, 2706–2710; 10.1002/anie.201309841 24482294

[31c] S. M. Manolikakes , M. Ellwart , C. I. Stathakis , P. Knochel , Chem. Eur. J. 2014, 20, 12289–12297; 25116852 10.1002/chem.201403015

[31d] Y.-H. Chen , M. Ellwart , V. Malakhov , P. Knochel , Synthesis 2017, 49, 3215–3223.

[32] Deposition number 2011115 (**14 c**) contains the supplementary crystallographic data for this paper. These data are provided free of charge by the joint Cambridge Crystallographic Data Centre and Fachinformationszentrum Karlsruhe Access Structures service.

[33a] S. Arseniyadis , K. S. Kyler , D. S. Watt in Organic Reactions, Vol. 31 (Eds.: Dauben W. G.), Wiley, Hoboken, 1984, pp. 1–344;

[33b] X. Yang , F. F. Fleming , Acc. Chem. Res. 2017, 50, 2556–2568. 28930437 10.1021/acs.accounts.7b00329

[34] X. Yang , D. Nath , J. Morse , C. Ogle , E. Yurtoglu , R. Altundas , F. Fleming , J. Org. Chem. 2016, 81, 4098–4102. 27171565 10.1021/acs.joc.6b00367

[35] Deposition number 2011117 (**6**) contains the supplementary crystallographic data for this paper. These data are provided free of charge by the joint Cambridge Crystallographic Data Centre and Fachinformationszentrum Karlsruhe Access Structures service.

[36] M. Ellwart , I. S. Makarov , F. Achrainer , H. Zipse , P. Knochel , Angew. Chem. Int. Ed. 2016, 55, 10502–10506; 10.1002/anie.201603923 27430745

[37] Small amounts of ZnBr_2_ are present in the allylic zinc reagents as a result of partial homocoupling during the zinc insertion.

[38] An extensive discussion of the bonding situation and charge distribution in selected intermediates can be found in the Supporting Information.

