# Supplementary material for: Highly Regioselective Addition of Allylic Zinc Halides and Various Zinc Enolates to [1.1.1]Propellane
Source: Angew Chem Int Ed Engl. 2020 Aug 31;59(45):20235–41. doi: 10.1002/anie.202009340 (PMC7693202; doi:10.1002/anie.202009340)
Supplement: Supplementary file 1 — Supplementary [file ANIE-59-20235-s001.pdf]

Angewandte Chemie
Eine Zeitschrift der Gesellschaft Deutscher Chemiker

Supporting Information

Highly Regioselective Addition of Allylic Zinc Halides and Various Zinc Enolates to [1.1.1]Propellane

*Kuno Schwärzer, Hendrik Zipse, Konstantin Karaghiosoff, and Paul Knochel**

anie_202009340_sm_miscellaneous_information.pdf

Table of Contents

Optimization of reaction conditions .... SI 2

NMR-studies .... SI 2

DOSY-spectroscopy... SI 4

General information .... SI 6

Preparation of starting materials .... SI 10

Typical procedures.. SI 12

Products.. SI 14

NMR-spectra... SI 44

Details for X-ray data collection and structure refinement .... SI 79

Optimization of reaction conditions

Reaction conditions were optimized using allylzinc bromide complexed with lithium chloride. Under the conditions, that had been optimized for the addition of arylmagnesium halides to [1.1.1]propellane[1] (2.0 equiv of organometallic species, 50 °C, 65 h or 100 °C, 3 h, Table S1, entries 1 and 2), the desired functionalized BCP **9a** was obtained in 95-96% yield after a copper mediated acylation with benzoyl chloride. This indicates, that the intermediary zincated BCP of type **8** is very stable even at high temperatures. Further reaction temperature variation showed that the addition was completed after only 2 hours at 25 °C (entries 3 and 4). A reduction of the amount of zinc reagent to 1.5 equiv lowered the yield to 71% (entry 5).

Table S1. Screening of reaction conditions for the addition of allyl zinc bromide complexed with lithium chloride (**7a**) to [1.1.1]propellane (**1**) followed by copper-catalyzed acylation.

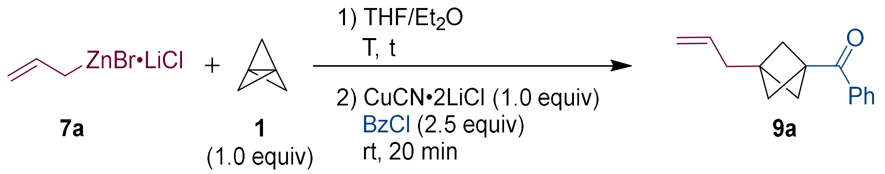

entry	equivalents of **7a**	reaction conditions	yield [%][a]
1	2.0	50 °C, 65 h	96[b]
2	2.0	100 °C, 3 h	95
3	2.0	25 °C, 1 h	88
4	2.0	25 °C, 2 h	96[b]
5	1.5	25 °C, 2 h	71[c]

[a] GC-yields using undecane as an internal standard. [b] Isolated yield of analytically pure product. [c] No yield improvement was observed after an additional hour of reaction time.

NMR-studies

To study the influence of lithium and zinc salts on [1.1.1]propellane a series of ^{13}C-NMR-spectra was measured (Figure S1). All of the NMR samples were prepared in a mixture of THF and Et_2O (1:1) with a sealed capillary tube filled with deuterated benzene (**d**) as NMR-standard for shimming. All samples contained traces of dibutyl ether (**b**) and bromobenzene (**c**), which result from the preparation and distillation of the [1.1.1]propellane (see below for a detailed procedure).

[1] I. S. Makarov, C. E. Brocklehurst, K. Karaghiosoff, G. koch, P. Knochel, *Angew. Chem. Int. Ed.* **2017**, *56*, 12774-12777; *Angew. Chem.* **2017**, *129*, 12949-12953.

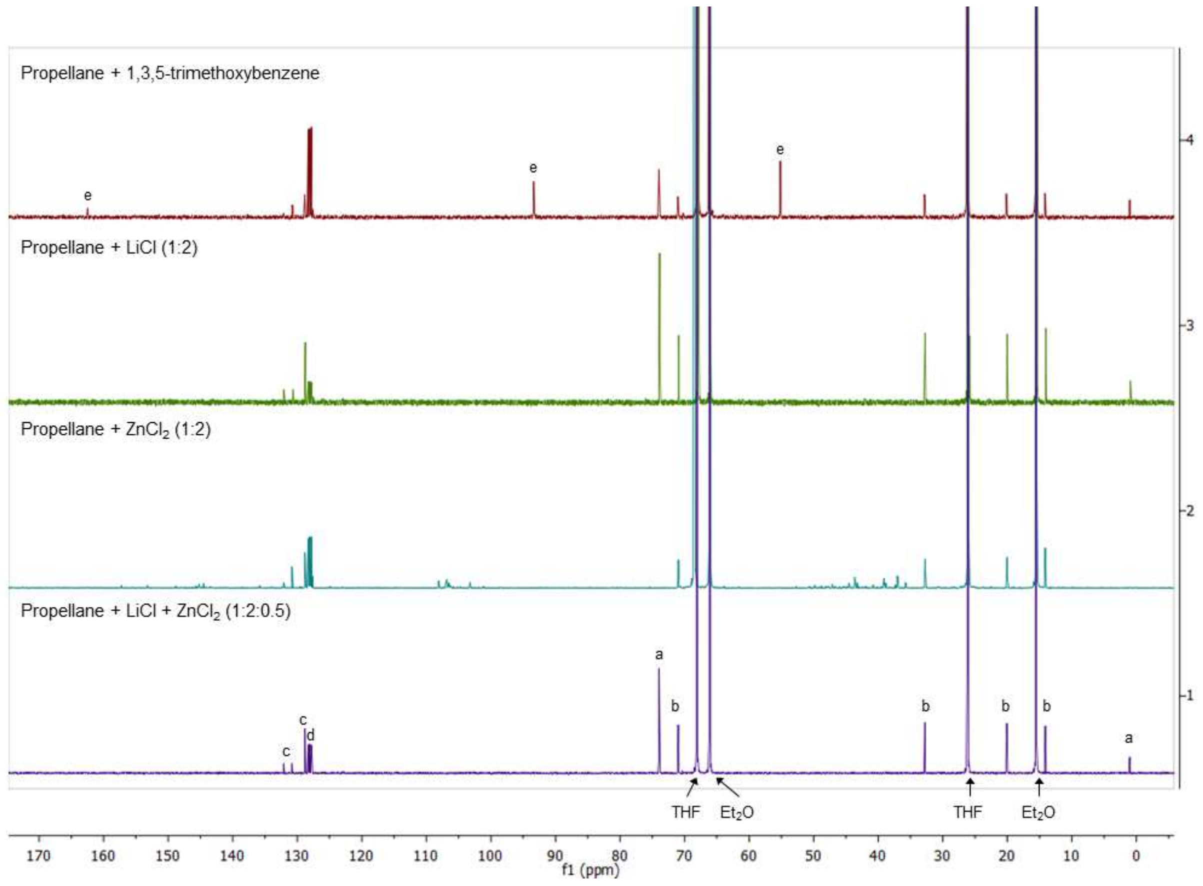

Figure S1. ^{13}C-NMR spectra of [1.1.1]propellane solutions containing lithium and zinc salts (**a** = [1.1.1]propellane, **b** = Bu_2O, **c** = PhBr, **d** = C_6D_6, **e** = 1,3,5-trimethoxybenzene).

As a reference a sample of [1.1.1]propellane with the NMR-standard 1,3,5-trimethoxybenzene (**e**) was measured, showing the two expected signals of [1.1.1]propellane (**a**) at 1.0 and 74.0 ppm. The addition of 2 equivalents of LiCl had no effect on the [1.1.1]propellane. However, the addition of 2 equivalents of $ZnCl_2$ let to a complete decomposition of the [1.1.1]propellane within 1 minute at room temperature as evidenced by the disappearance of the initial signals. A multitude of new signals between 35-55 ppm, 100-110 ppm and 130-160 ppm hints at the formation of a complex mixture of different products. A decomposition of [1.1.1]propellane in the presence of Lewis acidic transition-metal ions has been reported by Wiberg in the case of $AgBF_4$ and $[Rh(CO)_2Cl]_2$, leading to mixtures of different oligomers.[2] Interestingly, no decomposition was observed when the $ZnCl_2$ was premixed with 4 equivalents of LiCl in THF. This observation can be explained by assuming the formation of a zincate ($Li^+ZnCl_3^-$), which significantly reduces the Lewis acidity of the zinc. In addition, this leads to the conclusion that the complexes present in the solutions of allylic zinc halides (with or without complexated LiCl) are not Lewis acidic enough to cause the decomposition of

[2] K. B. Wiberg, S. T. Waddell, *J. Am. Chem. Soc.* **1990**, *112*, 2194-2216.

[1.1.1]propellane, thus allowing the formation of the respective ring-opening products in high yields.

DOSY-spectroscopy

In order to explore the aggregation state of the allylic zinc reagents a DOSY (Diffusion-Ordered NMR Spectroscopy) experiment was performed (Figure S2).[3] Therefore, a solution of allylzinc bromide coordinated with lithium chloride in THF was prepared according to procedure detailed below. The solvent was removed under high vacuum using a cooling trap and a small amount of the resulting solid was transferred to an NMR-tube under argon. In addition, 2,2,3,3,-tetramethylbutane (TMB), anthracene and tetrakis(trimethylsilyl)silane (0.06 mmol each) were added as internal standards. The solids were dissolved in 0.5 mL of THF-d_8 and the sample was submitted to the DOSY-experiment (measured on a *Bruker* AV400TR with a 5 mm PABBO BB/19F-1H/19F/D Z-GRD Z863001/0025 probe at 400.13 MHz using TopSpin by *Bruker Biospin*, Karlsruhe; pulse sequence: ledbpgp2s, 32 scans; transformation was performed in *MestreNova* 12.0 using Peak Heights Fit).

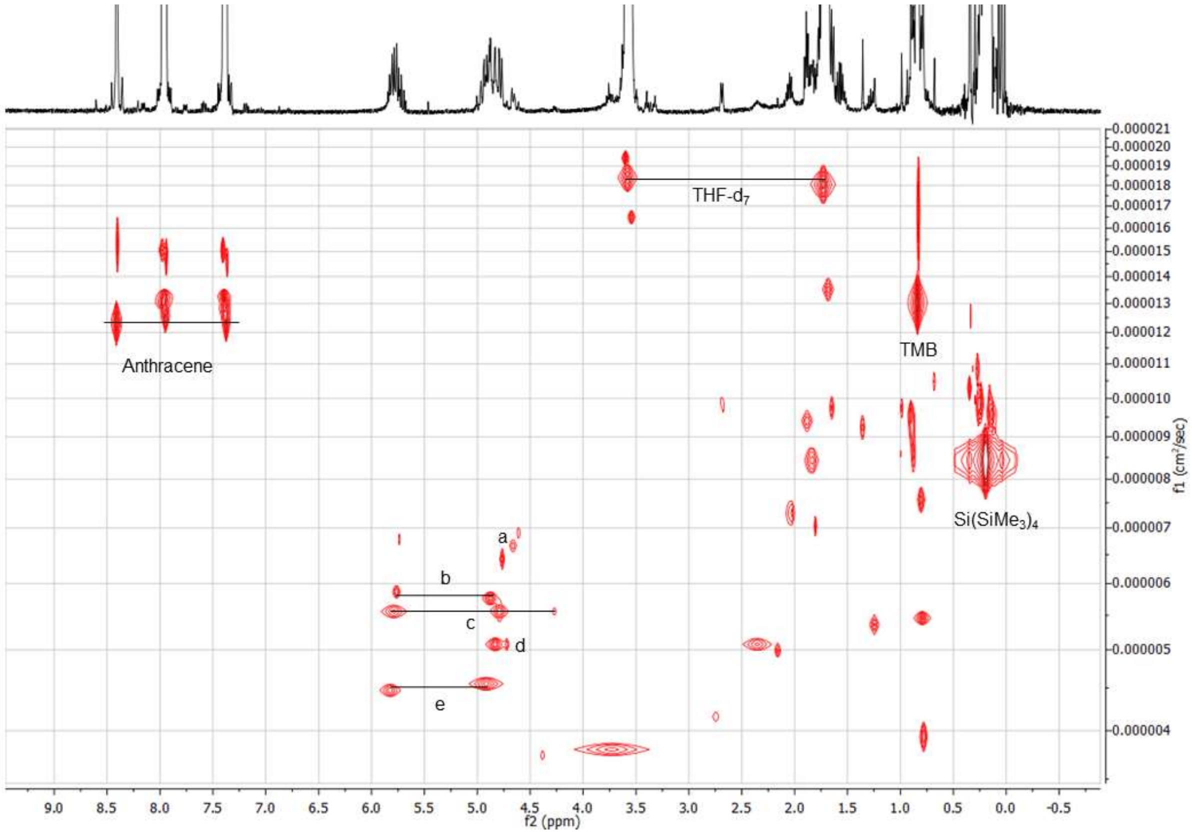

Figure S2. DOSY-NMR of allylzinc bromide coordinated with lithium chloride in THF-d_8.

---

[3] D. Li, I. Keresztes, R. Hopson, P. G. Williard, *Acc. Chem. Res.* **2009**, *42*, 270-280.

The DOSY-spectrum showed a multitude of different signals (**a**-**e**) corresponding to the allylic protons between 4.5 and 6.0 ppm. The diffusion coefficients D of these signals ranged from $6.43*10^{-6}$ to $4.51*10^{-6}$ cm^2/s. The signals of the internal standards were used to determine a calibration curve (Figure S3).

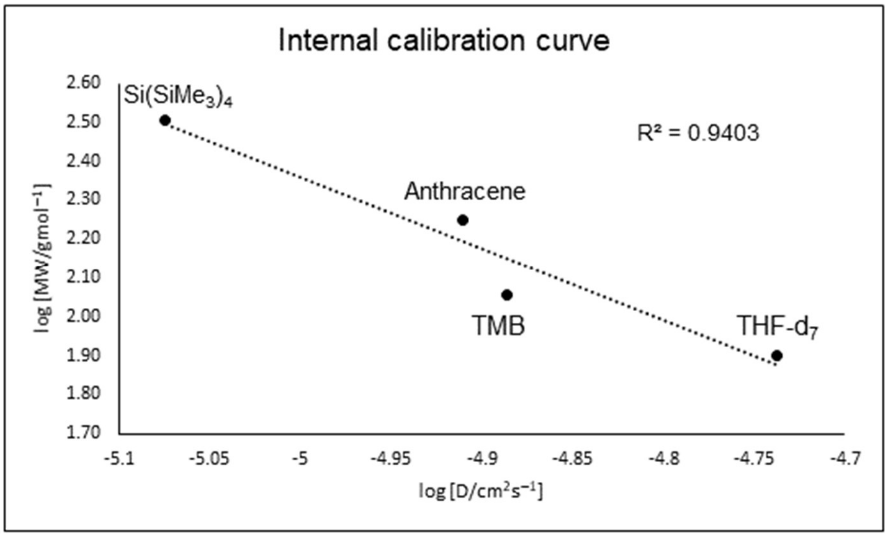

Figure S3. Internal calibration curve of the DOSY-spectrum of allylzinc bromide coordinated with lithium chloride.

The multitude of allylic signals leads to the conclusion, that the solution contains various clusters of allylzinc bromide in different aggregation states. The molecular weights of these clusters were estimated with the help of the internal calibration curve (Table S2).

Table S2. Diffusion parameters and calculated molecular weights for the different clusters present in allylzinc bromide coordinated with lithium chloride.

Compound	D [cm^2/s]	log [D/cm^2s^{-1}]	MW [g/mol]	log [$MW_{calc}/gmol^{-1}$]	MW_{calc} [g/mol]
**THF-d7**	1,83E-05	-4,737549	79,1	1,88	75
**TMB**	1,30E-05	-4,886056	114,2	2,15	141
**Anthracene**	1,23E-05	-4,9100949	178,2	2,19	156
**$Si(SiMe_3)_4$**	8,43E-06	-5,0741724	320,8	2,50	313
**a**	6,43E-06	-5,1917890	-	2,71	516±124
**b**	5,82E-06	-5,2350770	-	2,79	620±149
**c**	5,60E-06	-5,2518120	-	2,82	665±160
**d**	5,08E-06	-5,2941363	-	2,90	796±191
**e**	4,51E-06	-5,3458235	-	3,00	992±238

When applying the internal calibration to the diffusion parameters of the standards, the calculated molecular weights deviate from the actual values by 3% ($Si(SiMe_3)_4$) to 24% (TMB). By assuming similar margins of error for the allylic organozinc clusters, the molecular weights of the clusters **a**-**e** were estimated to range from 392 to 1230 g/mol. These molecular weights

are significantly higher than the molecular weight of a monomer of allylzinc bromide coordinated by one equivalent of LiCl and THF-d_8 (309 g/mol). Therefore, the DOSY experiments confirm the existence of oligomeric aggregates. A dimeric cluster of the formula $(allylZnBr)_2{\cdot}(LiCl)_2{\cdot}(THF\text{-}d_8)_2$ possesses a molecular weight of 618 g/mol, which is in good accordance with the value obtained for the signal **b** (620±149 g/mol). When substituting one of the allylzinc bromides by $ZnBr_2$, the resulting cluster $(allylZnBr){\cdot}(ZnBr_2){\cdot}(LiCl)_2{\cdot}(THF\text{-}d_8)_2$ weights 657 g/mol, which is close to the molecular weight determined for the signal **c** (665±160 g/mol). The signals **d** and **e** most likely correspond to more highly aggregated clusters.

General information

All reactions were carried out under an argon atmosphere in flame-dried glassware. Syringes which were used to transfer anhydrous solvents or reagents were purged with argon prior to use. Reactions at high temperatures were conducted in BIOTAGE Microwave vials sealed with the appropriate caps (up to 3 mL total volume) or Ace pressure vials. Yields refer to isolated yields of compounds estimated to be >95% pure as determined by ^{1}H-NMR (25 °C) and capillary GC. THF was continuously refluxed and freshly distilled from sodium benzophenone ketyl under nitrogen and stored under argon over molecular sieves. Solvents for column chromatography were distilled prior to use. All reagents were obtained from commercial sources and used without further purification unless otherwise stated. Reaction mixtures were cooled using an acetone / dry ice bath. The suspension formed during the work-up of reactions containing CuCN·2LiCl was dissolved by adding appropriate amounts of concentrated aqueous ammonia solution.

Reagents

*n*BuLi and PhLi solutions in hexane or dibutyl ether were purchased from Albemarle or Sigma Aldrich and the concentration was determined by titration against *N*-benzylbenzamide in THF at –40 °C or 0 °C respectively.[4]

TMPH was purchased from Albemarle (Frankfurt, Germany), freshly distilled over CaH_2 and stored under argon.

[4] A. F. Burchat, J. M. Chong, N. Nielsen, *J. Organomet. Chem.* **1997**, *542*, 281-283.

Diisopropylamine was purchased from Acros Organics, freshly distilled over CaH_2 and stored under argon.

CuCN·2LiCl solution (1.00 M in THF) was prepared by drying CuCN (8.96 g, 100 mmol, 1.00 equiv) and LiCl (8.48 g, 200 mmol, 2.00 equiv) in a *Schlenk*-flask under vacuum for 5 h at 150 °C. After cooling to 25 °C, dry THF (100 mL) was added and the resulting mixture was stirred until the salts were dissolved.

$ZnCl_2$ solution (1.00 M in THF) was prepared by drying $ZnCl_2$ (27.3 g, 200 mmol) in a *Schlenk*-flask under vacuum for 5 h at 150 °C. After cooling to 25 °C, dry THF (200 mL) was added and the resulting mixture was stirred until the salts were dissolved.

Chromatography

Flash column chromatographical purifications were performed using SiO_2 60 (0.040–0.063 mm, 230–400 mesh ASTM) or Al_2O_3 90 active acidic (0.063–0.200 mm, 70–230 mesh ASTM) from Merck. The activity of the Al_2O_3 was set to grade III by adding 4.5% distilled water and stirring at 40 °C for 2 h. Thin layer chromatography (TLC) was performed using aluminium plates covered with SiO_2 (Merck 60, F–254). Spots were visualized by UV light irradiation and/or by staining of the TLC plate with one of the reagents below, followed by heating with a heat gun if necessary.

- $KMnO_4$ (0.3 g), K_2CO_3 (20 g) and KOH (0.3 g) in water (300 mL).
- $Ce(SO_4)_2$ (5.0 g), $(NH_4)_6Mo_7O_{24}•4H_2O$ (25 g) and conc. H_2SO_4 (50 mL) in water (450 mL).
- Neat iodine absorbed on silica gel (no heating required).
- Vanillin (15 g) and conc. H_2SO_4 (2.5 mL) in EtOH (250 mL).
- *p*-Anisaldehyde (3.7 mL), conc. H_2SO_4 (5 mL) and acetic acid (1.5 mL) in EtOH (135 mL).

Preparative HPLC

For purification, an *Agilent Technologies* 1260 Infinity HPLC-System was used, consisting of two prep-pumps (acetonitrile/water, no additives), a MWD-detector (210 nm wavelength, 40 nm bandwidth, ref-wavelength 400 nm, ref-bandwidth 100 nm) and a fraction collector. Three different columns were used: 1) *Kinetix* EVO C18 5 µm column (length: 150 mm,

diameter: 10 mm), 2) *Kinetix* EVO C18 5 µm column (length: 150 mm, diameter: 21.2 mm) and 3) *Waters* XBridge Prep C8 5 µm column (length: 150 mm, diameter: 30 mm).

Analytical data

NMR spectra were recorded on *Bruker* ARX 200, AC 300, WH 400 or AMX 600 instruments. Chemical shifts are reported as δ-values in ppm relative to the deuterated solvent peak: $CDCl_3$ (δ_H: 7.26; δ_C: 77.16). For the observation of the observed signal multiplicities, the following abbreviations and combinations thereof were used: s (singlet), d (doublet), t (triplet), q (quartet), quint (quintet), sext (sextet), sept (septet), m (multiplet) and br (broad). If not otherwise noted, the coupling constants given are either H-H or H-F coupling constants for proton signals and C-F coupling constants for carbon signals. In the cases where a steric centre is located next to a bicyclopentane core, the protons of the methylene groups in the BCP unit are diastereotopic and show the splitting pattern expected for the resulting $[AB]_3$-system.

Melting points are uncorrected. and were measured on a *Büchi* B.540 apparatus.

Infrared spectra were recorded from 4000-400 cm^{-1} on a *Perkin Elmer* Spectrum BX-59343 instrument. For detection a Smiths Detection DuraSampl IR II Diamond ATR sensor was used. The main absorption peaks are reported in cm^{-1}. The abbreviations used to describe the signals are vs (very strong, above 80% of max intensity), s (strong, from 80% to 60% of max intensity), m (medium, from 60% to 40% of max intensity), w (weak, from 40% to 20% of max intensity) and vw (very weak, below 20% of max intensity).

Gas chromatographical analysis (GC) was performed with instruments of the type Hewlett-Packard 6890 or 5890 Series II, using a column of the type HP 5 (Hewlett-Packard, 5% phenylmethylpolysiloxane; length: 10 m, diameter: 0.25 mm, film thickness 0.25 µm). The detection was accomplished using a flame ionization detector. Mass spectra (MS) and high resolution mass spectra (HRMS) were recorded on a *Finnigan* MAT 95Q or *Finnigan* MAT 90 instrument for electron impact ionization (EI). For the combination of gas chromatography with mass spectroscopic detection, a GC–MS of the type *Hewlett-Packard* 6890/MSD 5793 networking was used (column: HP 5–MS, Hewlett–Packard; 5% phenylmethylpolysiloxane; length: 15 m, diameter: 0.25 mm, film thickness: 0.25 µm).

Single crystals of compounds suitable for X-ray diffraction were obtained by slow evaporation of either EtOAc or $CDCl_3$ solutions. The crystals were introduced into perfluorinated oil and a suitable single crystal was carefully mounted on the top of a thin glass wire. Data collection

was performed with an Oxford Xcalibur 3 diffractometer equipped with a Spellman generator (50 kV, 40 mA) and a Kappa CCD detector, operating with Mo-K_α radiation (λ = 0.71071 Å).

Data collection was performed with the CrysAlis CCD software;[5] CrysAlis RED software[6] was used for data reduction. Absorption correction using the SCALE3 ABSPACK multiscan method[7] was applied. The structures were solved with SHELXS-97,[8] refined with SHELXL-97[9] and finally checked using PLATON.[10] Details for data collection and structure refinement are summarized in the tables at the end of this document.

The 1-octanol/water partitioning coefficient (logP) was determined using a miniaturized Shake-Flask equilibrium method. Prior to start the experiment the two phases were pre-saturated, so "water-saturated 1-octanol" and "1-octanol-saturated water" were used. The samples were initially dissolved in DMSO as a 10mM stock concentration. The samples and an internal standard were dispensed in a 1ml deepwell plate and DMSO is evaporated prior to be dissolved in 1-octanol at a target concentration of 150µM by shaking at 1000rpm during 8 hours. The pH 7.4 buffer was added with a phase ratio K of 1 (where K = $V_{water}/V_{octanol}$) and then the samples were shaken 4 hours on a shaker at 1000rpm. The deepwell plate was centrifuged at 3000rpm prior to phase separation. A x10 dilution for the aqueous phase and a x1000 dilution for the octanol phase are prepared and quantified by LC-HRMS against an internal standard (Dexamethasone) with a known logD=1.9 with the following equation:[11]

$$logD = \log\left(\frac{{}^{\left(Analyte\ peak\ area\ in\ octanol * 1000\right)}\!/\!{}_{IS\ peak\ area\ in\ octanol} \Big/ 0.794}{{}^{Analyte\ peak\ area\ in\ aqueous * 10}\!/\!{}_{IS\ peak\ area\ in\ aqueous}}\right)$$

Column used: Zorbax_SB_AQ 50 x 2.1 mm 1.8µm – Column oven temperature = 50 °C

[5] CrysAlis CCD, Oxford Diffraction Ltd., Version 1.171.27p5 beta (release 01-04-2005 CrysAlis171.NET) (compiled Apr 1 2005, 17:53:34).
[6] CrysAlis RED, Oxford Diffraction Ltd., Version 1.171.27p5 beta (release 01-04-2005 CrysAlis171.NET) (compiled Apr 1 2005, 17:53:34).
[7] SCALE3 ABSPACK – An Oxford Diffraction Program (1.0.4, gui:1.0.3) (C), Oxford Diffraction, Ltd., 2005.
[8] G. M. Sheldrick (1997) SHELXS-97: *Program for Crystal Structure Solution*, University of Göttingen, Germany.
[9] G. M. Sheldrick (1997) SHELXS-97: *Program for the Refinement of Crystal Structures*, University of Göttingen, Germany.
[10] A. L. Spek (1999) PLATON: *A Multipurpose Crystallographic Tool*, Utrecht University, Utrecht, the Netherlands.
[11] Y. W. Low, F. Blasco, P. Vachaspati, *Eur. J. Pharm. Sci.* **2016**, *92*, 110-116.

Mobile phase: A= 100% water UHPLC grade + 0.08% formic acid. B= 100% ACN + 0.08% Formic acid. Flow rate = 0.5ml/min. Gradient mode: starting at 95% A up to 95% B in 0.5min and kept constant during 1min before to restore initial conditions within 0.1min. Vinj = 5µl. Full MS acquisition mode – Full scan 130 to 1800m/z and resolution = 35'000. $[M+H]^+$ ion chromatogram was extracted for each compounds.

Potentiometric ionization constants were determined on the commercial SiriusT3 instruments (Pion-inc.com) as described by Takács-Novák et al. 1997. Briefly, 0.3 to 1 mM of test solutions were titrated from pH 2 to 12 for bases or 12 to 2 for acids. Titrations were conducted at 25°C and in 0.15 M ionic strength. Aqueous titrations were performed in triplicate in 0.15 M KCl, while sparingly soluble test compounds were titrated in 10-60 %wt methanol, 1,4-dioxane, or dimethyl sulfoxide cosolvent. A minimum of three titrations in varying amounts of cosolvent were performed for extrapolation to the aqueous pKa. For each titration, initial estimates of pKa values were obtained from Bjerrum difference plots (number of bound protons versus pH) and then were refined by a weighted non-linear least-squares procedure (Avdeef 1992, 1993) available in the instrument software. Experimental variability was determined from 389 duplicate measurements from different days and experimentalists, with a standard deviation of 0.28.

Preparation of starting materials

The following reagents were prepared according to literature procedures: 1,1-dibromo-2,2-bis(chloromethyl)cyclopropane,[12] *N,N*-diallyl-*O*-benzoylhydroxylamine,[13] *S*-phenyl benzene-sulfonothioate *,* *S*-methyl benzenesulfonothioate,[14] ethyl 2-(bromomethyl)acrylate,[15] ethyl 6-chlorocyclohex-1-ene-1-carboxylate[16], (1*R*)-myrtenyl bromide[17] and 5-bromocyclopent-1-enecarbonitrile.[18]

[12] K. R. Mondanaro, W. P. Dailey, *Org. Synth.* **1998**, *75*, 98-101.
[13] Y.-H. Chen, S. Graßl, P. Knochel, *Angew. Chem. Int. Ed.* **2018**, *57*, 1108-1111; *Angew. Chem.* **2018**, *130*, 1120-1124.
[14] K. Fujiki, N. Tanufuji, Y. Sasaki, T. Yokoyama, *Synthesis* **2002**, *3*, 343-348.
[15] J. Caillé, M. Pantin, F. Boeda, M. S. M. Pearson-Long, P. Bertus, *Synthesis* **2019**, *51*, 1329-1341.
[16] Z. Peng, T. D. Blümke, P. Mayer, P. Knochel, *Angew. Chem. Int. Ed.* **2010**, *49*, 8516-8519; *Angew. Chem.* **2010**, *122*, 8695-8698.
[17] R. K. de Richter, M. Bonato, M. Follet, J.-M. Kamenka, *J. Org. Chem.* **1990**, *55*, 2855-2860
[18] G. L. Lackner, K. W. Quasdorf, G. Pratsch, L. E. Overman, *J. Org. Chem.* **2015**, *80*, 6012-6024.

Preparation of the solution of [1.1.1]propellane (1) in diethyl ether

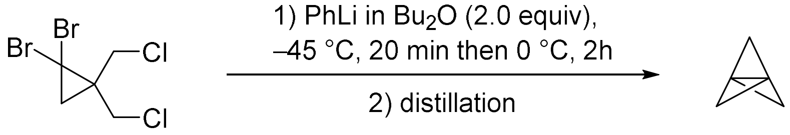

In a dry Schlenk-flask 1,1-dibromo-2,2-bis(chloromethyl)cyclopropane (5.94 g, 20 mmol, 1.0 equiv) was dissolved in Et_2O (20 mL) and cooled to –45 °C. Phenyllithium (1.80 M in dibutyl ether, 22.2 mL, 40 mmol, 2.0 equiv) was added dropwise over 15 min using a syringe pump. The resulting mixture was stirred for 5 min at –45 °C and 2 h at 0 °C. Then an argon flushed distillation head connected to a collector Schlenk tube immersed into an aceton/ dry ice bath (T = –78 °C) was attached and the distillation was started at 200 mbar. The reaction flask was removed from the ice bath and the pressure was gradually lowered to 45 mbar. After approximately 30 min the distillation was stopped and the system was filled with argon. An aliquot (0.1 mL) of the solution was analysed by NMR spectroscopy with 1,3,5-trimethoxybenzene (16.8 mg, 0.1 mmol) as a standard to determine the concentration. The average concentration of the [1.1.1]propellane solution was 0.5-0.7 M. The solution was stored at –25 °C under argon.

General procedure for the preparation of allylic organozinc reagents of type 7 in THF

A Schlenk flask was loaded with zinc dust (2.0 equiv) and lithium chloride (1.2 equiv) and flame dried with a heat gun under vacuum at 450 °C. Once the flask was cooled down it was filled with argon and THF (1 mL/mmol halide). The zinc was activated by adding a drop of 1,2-dibromoethane and heating until a slight gas evolution started. Then the reaction mixture was cooled to 0 °C and the respective organohalide (1.0 equiv) was added dropwise. The reaction mixture was stirred over night while slowly warming up to room temperature. The remaining zinc dust was removed using a syringe filter and the concentration was determined via titration against iodine.[19]

[19] A. Krasovskiy, P. Knochel, *Synthesis* **2006**, 890.

Typical procedures

TP1: Typical procedure for the reaction of allylic organozinc reagents of type 7 with [1.1.1]propellane (1) followed by electrophile addition

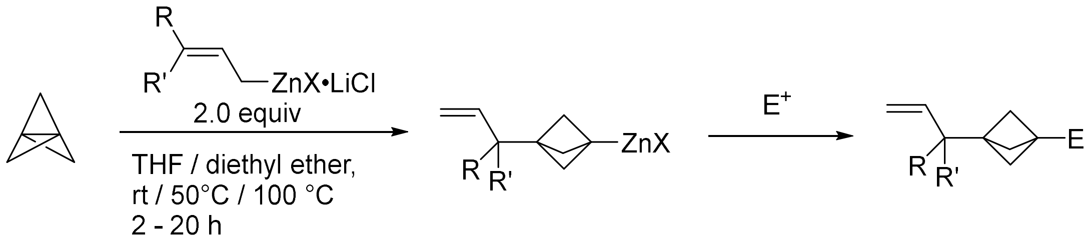

A BIOTAGE Microwave vial under argon was loaded with the allylic organozinc reagent in THF (0.40 mmol, 2.0 equiv), followed by [1.1.1]propellane in diethyl ether (0.20 mmol, 1.0 equiv). The vial was sealed and stirred at the respective temperature (25 °C / 50 °C / 100 °C) for the indicated amount of time. Then the reaction mixture was cooled down to room temperature and the electrophile was added. The reaction was stirred until GCMS analysis showed full conversion. Afterwards a saturated aqueous solution of NH_4Cl (1 mL) was added and the reaction mixture was extracted with EtOAc (3 times), washed with brine, dried over $MgSO_4$ and concentrated *in vacuo*. The crude product was purified via silica gel column chromatography using an appropriate mixture of *i*-hexane and EtOAc as eluent.

TP2: Typical procedure for the reaction of zinc enolates of type 10 with [1.1.1]propellane (1) followed by electrophile addition

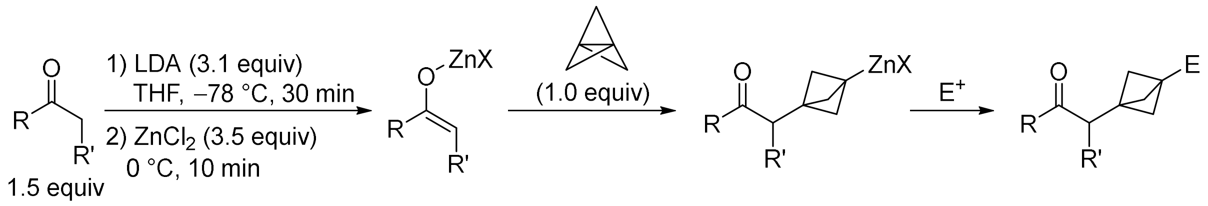

Diisopropylamine (63 mg, 0.62 mmol, 3.1 equiv) was dissolved in THF (0.6 mL) and BuLi (2.55 M in hexane, 0.24 mL, 0.62 mmol, 3.1 equiv) was added dropwise at 0 °C. The mixture was stirred for 5 min and cooled to –78 °C. Then the ketone (0.30 mmol, 1.5 equiv) was added. After 30 min a solution of $ZnCl_2$ in THF (1.0 M, 0.70 mL, 0.70 mmol, 3.5 equiv) was added and the mixture was stirred at 0 °C for 5 min before adding the [1.1.1]propellane in diethyl ether (0.20 mmol, 1.0 equiv). After stirring at 0 °C or 25 °C for the indicated time the electrophile was added and the mixture was stirred until GCMS analysis showed full conversion. Then a saturated aqueous solution of NH_4Cl (1 mL) was added and the reaction mixture was extracted with EtOAc (3 times), washed with brine, dried over $MgSO_4$ and concentrated *in vacuo*. The

crude product was purified via silica gel column chromatography using an appropriate mixture of *i*-hexane and EtOAc as eluent.

TP3: Typical procedure for the reaction of zinc ester enolates of type 11 with [1.1.1]propellane (1) followed by electrophile addition

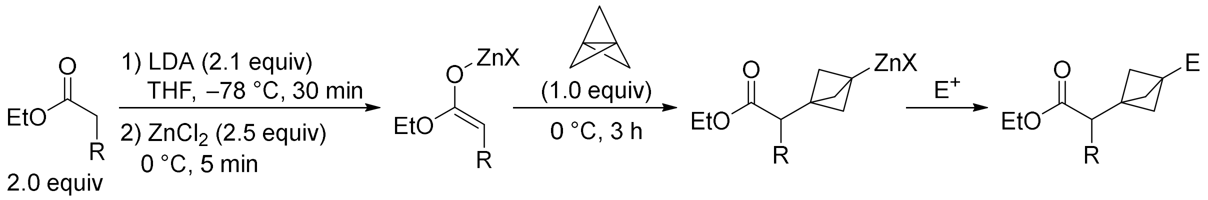

Diisopropylamine (43 mg, 0.42 mmol, 2.1 equiv) was dissolved in THF (0.5 mL) and BuLi (2.55 M in hexane, 0.16 mL, 0.42 mmol, 2.1 equiv) was added dropwise at 0 °C. The mixture was stirred for 5 min and cooled to –78 °C. Then the ester (0.40 mmol, 2.0 equiv) was added. After 30 min a solution of $ZnCl_2$ in THF (1.0 M, 0.50 mL, 0.50 mmol, 2.5 equiv) was added and the mixture was stirred at 0 °C for 5 min before adding the [1.1.1]propellane in diethyl ether (0.20 mmol, 1.0 equiv). After stirring at 0 °C for 3 h the electrophile was added and the mixture was stirred until GCMS analysis showed full conversion. Then a saturated aqueous solution of NH_4Cl (1 mL) was added and the reaction mixture was extracted with EtOAc (3 times), washed with brine, dried over $MgSO_4$ and concentrated *in vacuo*. The crude product was purified via silica gel column chromatography using an appropriate mixture of *i*-hexane and EtOAc as eluent.

TP4: Typical procedure for the reaction of nitrile-stabilized zinc enolates of type 19 with [1.1.1]propellane (1) followed by copper-catalyzed allylation

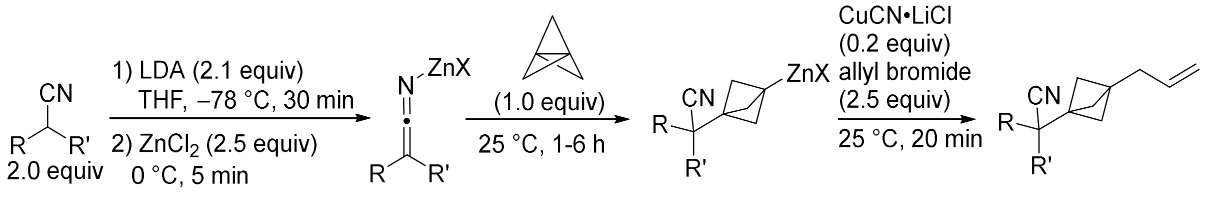

Diisopropylamine (43 mg, 0.42 mmol, 2.1 equiv) was dissolved in THF (0.5 mL) and BuLi (2.55 M in hexane, 0.16 mL, 0.42 mmol, 2.1 equiv) was added dropwise at 0 °C. The mixture was stirred for 5 min and cooled to –78 °C. Then the nitrile (0.40 mmol, 2.0 equiv) was added. After 30 min a solution of $ZnCl_2$ in THF (1.0 M, 0.50 mL, 0.50 mmol, 2.5 equiv) was added and the mixture was stirred at 0 °C for 5 min before adding the [1.1.1]propellane in diethyl ether

(0.20 mmol, 1.0 equiv). After stirring at 25 °C for the indicated time CuCN·2LiCl in THF (1.0 M, 0.04 mL, 0.04 mmol, 20 mol%) and allyl bromide (61 mg, 0.50 mmol, 2.5 equiv) were added and the mixture was stirred for 20 min at 25 °C. Then a saturated aqueous solution of NH_4Cl (1 mL) was added and the reaction mixture was extracted with EtOAc (3 times), washed with brine, dried over $MgSO_4$ and concentrated *in vacuo*. The crude product was purified via silica gel column chromatography using an appropriate mixture of *i*-hexane and EtOAc as eluent.

Products

(3-Allylbicyclo[1.1.1]pentan-1-yl)(phenyl)methanone (9a)

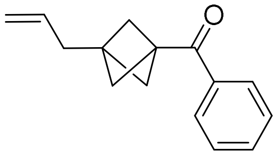

(3-Allylbicyclo[1.1.1]pentan-1-yl)(phenyl)methanone was prepared according to **TP1** using allylzinc bromide coordinated with lithium chloride (0.90 M, 0.44 mL, 0.40 mmol, 2.0 equiv). The reaction was stirred at 25 °C for 2 h before adding CuCN·2LiCl in THF (1.0 M, 0.2 mL, 0.20 mmol, 1.0 equiv) and benzoyl chloride (70 mg, 0.50 mmol, 2.5 equiv). The resulting mixture was stirred at 25 °C for 4 h. Workup according to **TP1** and purification via column chromatography (*i*Hex / EtOAc = 49 / 1) afforded the desired compound **9a** (41 mg, 0.19 mmol, 96%) as a colorless liquid.

^{1}H-NMR ($CDCl_3$, 400 MHz, ppm): δ = 7.5, 1.2 Hz, 2 H), 7.53 (tt, J = 7.5, 1.2 Hz, 1 H), 7.43 (t, J = 7.5 Hz, 2 H), 5.82 – 5.68 (m, 1 H), 5.10 – 5.00 (m, 2 H), 2.30 (dt, J = 7.2, 1.2 Hz, 2 H), 2.15 (s, 6 H).

^{13}C-NMR ($CDCl_3$, 101 MHz, ppm): δ = 197.9, 136.8, 134.7, 132.9, 129.0, 128.5, 116.7, 53.4, 45.0, 39.8, 36.5.

MS (70 eV, EI) *m/z* (%): 212 (1) $[M]^{+}$, 197 (11), 171 (23), 153 (11), 143 (10), 141 (11), 128 (19), 105 (100), 91 (20), 79 (13), 77 (43).

IR (ATR) $\widetilde{\nu}$ (cm^{-1}): 3074 (vw), 2974 (w), 2910 (vw), 2874 (w), 1719 (vw), 1662 (s), 1641 (w), 1598 (w), 1579 (w), 1510 (vw), 1447 (w), 1340 (s), 1302 (w), 1289 (w), 1267 (w), 1204 (s), 1176 (m), 1086 (vw), 1070 (vw), 1025 (w), 992 (w), 912 (m), 867 (m), 759 (m) 712 (w), 692 (vs), 675 (s).

HRMS (EI) calculated for $C_{15}H_{15}O^{\cdot+}$: 211.1117, found 212.1118 $[M–H]^{\cdot+}$.

Phenyl(3-(1-phenylallyl)bicyclo[1.1.1]pentan-1-yl)methanone (9b)

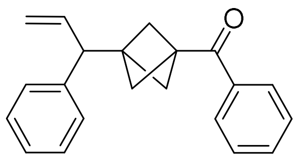

Phenyl(3-(1-phenylallyl)bicyclo[1.1.1]pentan-1-yl)methanone was prepared according to **TP1** using cinnamylzinc bromide coordinated with lithium chloride (0.53 M, 0.75 mL, 0.40 mmol, 2.0 equiv). The reaction was stirred at 50 °C for 7 h before adding CuCN·2LiCl in THF (1.0 M, 0.2 mL, 0.20 mmol, 1.0 equiv) and benzoyl chloride (70 mg, 0.50 mmol, 2.5 equiv). The resulting mixture was stirred at 25 °C for 3 h. Workup according to **TP1** and purification via column chromatography (*i*Hex / EtOAc = 99 / 1) afforded the desired compound **9b** (54 mg, 0.19 mmol, 93%) as colorless crystals. The structure was confirmed via single crystal X-ray diffraction studies.

^{1}H-NMR ($CDCl_3$, 400 MHz, ppm): δ = 7.96 (d, J = 7.1 Hz, 2 H), 7.53 (tt, J = 7.5, 2.0 Hz, 1 H), 7.42 (t, J = 7.7 Hz, 2 H), 7.34 (t, J = 7.4 Hz, 2 H), 7.28 – 7.22 (m, 1 H), 7.18 (d, J = 6.8 Hz, 2 H), 6.12 (ddd, J = 17.0, 10.3, 8.4 Hz, 1 H), 5.21 – 5.07 (m, 2 H), 3.50 (d, J = 8.4 Hz, 1 H), 2.15 (A part of an $[AB]_3$-system, 3 H), 2.09 (B part of an $[AB]_3$-system, 3 H).

^{13}C-NMR ($CDCl_3$, 101 MHz, ppm): δ = 197.9, 141.3, 137.6, 136.7, 132.9, 129.0, 128.6, 128.5, 128.0, 126.6, 116.6, 52.3, 51.7, 45.0, 43.5.

MS (70 eV, EI) *m/z* (%): 287 (3) $[M–H]^{·+}$, 183 (14), 171 (20), 168 (17), 167 (23), 165 (12), 155 (20), 153 (30), 152 (12), 143 (15), 141 (38), 129 (14), 128 (55), 117 (33), 115 (61), 105 (100), 91 (22), 77 (38).

IR (ATR) $\tilde{\nu}$ (cm^{-1}): 2978 (vw), 1661 (s), 1636 (vw), 1595 (w), 1577 (vw), 1488 (vw), 1447 (w), 1331 (m), 1277 (w), 1203 (m), 1175 (w), 1069 (w), 1022 (w), 998 (w), 931 (s), 877 (w), 839 (w), 784 (w), 759 (m), 730 (m), 699 (vs), 676 (m).

HRMS (EI) calculated for $C_{21}H_{19}O^{·+}$: 287.1430, found 287.1432 $[M–H]^{·+}$.

mp: 86.8 - 88.4 °C.

1-Allyl-3-(4-methoxyphenyl)bicyclo[1.1.1]pentane (9c)

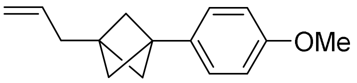

1-Allyl-3-(4-methoxyphenyl)bicyclo[1.1.1]pentane was prepared according to **TP1** using allylzinc bromide coordinated with lithium chloride (0.90 M, 0.44 mL, 0.40 mmol, 2.0 equiv). The reaction was stirred at 50 °C for 3 h before adding 4-iodoanisole (117 mg, 0.50 mmol, 2.5 equiv), $PdCl_2(dppf){\cdot}CH_2Cl_2$ (8.2 mg, 0.01 mmol, 5 mol%) and CuCN·2LiCl in THF (1.0 M, 0.02 mL, 0.02 mmol, 10 mol%). The resulting mixture was stirred at 45 °C for 3 h. Workup according to **TP1** and purification via column chromatography (*i*Hex / EtOAc = 99 / 1) and HPLC afforded the desired compound **9c** (39 mg, 0.18 mmol, 92%) as a colorless liquid.

^{1}H-NMR ($CDCl_3$, 400 MHz, ppm): δ = 7.14 (d, J = 8.6 Hz, 2 H), 6.84 (d, J = 8.6 Hz, 2 H), 5.79 (ddt, J = 17.3, 10.1, 7.2 Hz, 1 H), 5.09 – 4.97 (m, 2 H), 3.79 (s, 3 H), 2.30 (dt, J = 7.3, 1.2 Hz, 2 H), 1.88 (s, 6 H).

^{13}C-NMR ($CDCl_3$, 101 MHz, ppm): δ = 158.3, 135.8, 134.0, 127.2, 115.9, 113.6, 55.4, 52.2, 41.9, 37.8, 36.9.

MS (70 eV, EI) *m/z* (%): 214 (18) $[M]^{+}$, 199 (42), 186 (97), 185 (34), 184 (33), 173 (43), 171 (66), 159 (20), 158 (99), 155 (29), 148 (64), 145 (18), 144 (17), 141 (30), 133 (100), 129 (21), 128 (37), 121 (55), 118 (22), 115 (42), 105 (15), 103 (21), 91 (30), 89 (16), 79 (17), 77 (18).

IR (ATR) $\tilde{\nu}$ (cm^{-1}): 2956 (w), 2903 (w), 2865 (w), 2834 (vw), 1641 (vw), 1610 (w), 1578 (vw), 1519 (m), 1503 (m), 1463 (w), 1441 (w), 1413 (vw), 1353 (vw), 1294 (m), 1263 (m), 1243 (vs), 1172 (m), 1132 (w), 1098 (w), 1037 (s), 991 (m), 908 (m), 832 (s), 801 (m), 791 (m), 666 (w).

HRMS (EI) calculated for $C_{15}H_{18}O^{\cdot+}$: 214.1352, found 214.1350 $[M]^{\cdot+}$.

Ethyl 4-(3-allylbicyclo[1.1.1]pentan-1-yl)benzoate (9d)

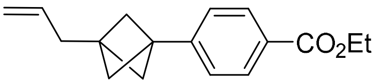

Ethyl 4-(3-allylbicyclo[1.1.1]pentan-1-yl)benzoate was prepared according to **TP1** using allylzinc bromide coordinated with lithium chloride (0.90 M, 0.44 mL, 0.40 mmol, 2.0 equiv). The reaction was stirred at 50 °C for 3 h before adding 4-iodobenzoate (138 mg, 0.50 mmol, 2.5 equiv), $PdCl_2(dppf){\cdot}CH_2Cl_2$ (8.2 mg, 0.01 mmol, 5 mol%) and CuCN·2LiCl in THF (1.0 M, 0.02 mL, 0.02 mmol, 10 mol%). The resulting mixture was stirred at 45 °C for 19 h. Workup

according to **TP1** and purification via column chromatography (*i*Hex / EtOAc = 99 / 1) and HPLC afforded the desired compound **9d** (50 mg, 0.19 mmol, 97%) as a colorless liquid.

^{1}H-NMR ($CDCl_3$, 400 MHz, ppm): δ = 7.96 (d, J = 8.3 Hz, 2 H), 7.25 (d, J = 8.3 Hz, 2 H), 5.77 (ddt, J = 17.3, 10.2, 7.2 Hz, 1 H), 5.12 – 4.96 (m, 2 H), 4.36 (q, J = 7.1 Hz, 2 H), 2.31 (dt, J = 7.2, 1.2 Hz, 2 H), 1.93 (s, 6 H), 1.38 (t, J = 7.1 Hz, 3 H).

^{13}C-NMR ($CDCl_3$, 101 MHz, ppm): δ = 166.8, 146.6, 135.4, 129.6, 128.5, 126.2, 116.2, 60.9, 52.2, 42.3, 38.2, 36.7, 14.5.

MS (70 eV, EI) *m/z* (%): 256 (1) $[M]^{\cdot+}$, 228 (20), 211 (11), 200 (13), 183 (16), 169 (10), 168 (15), 167 (16), 165 (10), 156 (12), 155 (100), 154 (15), 153 (31), 152 (11), 145 (23), 143 (30), 142 (20), 141 (94), 129 (27), 128 (60), 117 (11), 115 (52), 91 (24), 77 (11).

IR (ATR) $\widetilde{\nu}$ (cm^{-1}): 2964 (w), 2906 (vw), 2868 (w), 1713 (vs), 1641 (vw), 1610 (w), 1445 (vw), 1407 (w), 1367 (w), 1307 (w), 1266 (vs), 1174 (m), 1156 (w), 1104 (vs), 1093 (s), 1020 (m), 991 (w), 907 (m), 857 (w), 780 (w), 755 (m), 700 (s).

HRMS (EI) calculated for $C_{17}H_{20}O_2^{\cdot+}$: 256.1458, found 256.1457 $[M]^{\cdot+}$.

2-(3-Allylbicyclo[1.1.1]pentan-1-yl)pyridine (9e)

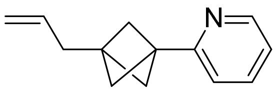

2-(3-Allylbicyclo[1.1.1]pentan-1-yl)pyridine was prepared according to **TP1** using allylzinc bromide coordinated with lithium chloride (0.90 M, 0.44 mL, 0.40 mmol, 2.0 equiv). The reaction was stirred at 25 °C for 2 h before adding 2-bromopyridine (79 mg, 0.50 mmol, 2.5 equiv), $PdCl_2(dppf)\cdot CH_2Cl_2$ (8.2 mg, 0.01 mmol, 5 mol%) and CuCN·2LiCl in THF (1.0 M, 0.02 mL, 0.02 mmol, 10 mol%). The resulting mixture was stirred at 45 °C for 19 h. Workup according to **TP1** and purification via column chromatography (*i*Hex / EtOAc = 19 / 1) and HPLC afforded the desired compound **9e** (26 mg, 0.14 mmol, 70%) as a colorless liquid.

^{1}H-NMR ($CDCl_3$, 400 MHz, ppm): δ = 8.54 (ddd, J = 4.9, 1.8, 0.9 Hz, 1 H), 7.60 (td, J = 7.7, 1.8 Hz, 1 H), 7.17 (dt, J = 7.8, 1.1 Hz, 1 H), 7.11 (ddd, J = 7.6, 4.9, 1.2 Hz, 1 H), 5.78 (ddt, J = 17.2, 10.2, 7.2 Hz, 1 H), 5.10 – 4.98 (m, 2 H), 2.32 (dt, J = 7.2, 1.3 Hz, 2 H), 2.00 (s, 7 H).

^{13}C-NMR ($CDCl_3$, 101 MHz, ppm): δ = 160.1, 149.3, 136.4, 135.4, 121.5, 120.8, 116.2, 51.9, 43.1, 38.3, 36.7.

MS (70 eV, EI) *m/z* (%): 184 (22) $[M-H]^{\cdot+}$, 170 (16), 168 (15), 158 (10), 156 (15), 154 (10), 145 (10), 144 (100), 143 (32), 142 (15), 130 (13), 117 (17).

IR (ATR) $\tilde{\nu}$ (cm^{-1}): 3076 (w), 2967 (m), 2907 (w), 2869 (w), 1641 (w), 1589 (vs), 1567 (m), 1514 (w), 1474 (vs), 1432 (s), 1362 (vw), 1289 (w), 1265 (vs), 1169 (s), 1050 (w), 992 (m), 912 (s), 788 (w), 753 (m), 698 (w).

HRMS (EI) calculated for $C_{13}H_{14}N^{\cdot+}$: 184.1121, found 184.1119 $[M-H]^{\cdot+}$.

(3-Allylbicyclo[1.1.1]pentan-1-yl)(phenyl)sulfane (9f)

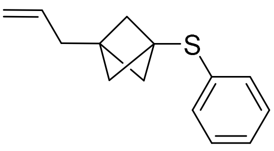

(3-Allylbicyclo[1.1.1]pentan-1-yl)(phenyl)sulfane was prepared according to **TP1** using allylzinc bromide coordinated with lithium chloride (0.90 M, 0.44 mL, 0.40 mmol, 2.0 equiv). The reaction was stirred at 50 °C for 3 h before adding *S*-phenyl benzenesulfonothioate (125 mg, 0.50 mmol, 2.5 equiv). The resulting mixture was stirred at 25 °C for 4 h. Workup according to **TP1** and purification via column chromatography (*i*Hex) afforded the desired compound **9f** (41 mg, 0.19 mmol, 95%) as a colorless liquid.

^{1}H-NMR (CDCl$_3$, 400 MHz, ppm): δ = 7.39 – 7.32 (m, 2 H), 7.25 – 7.16 (m, 3 H), 5.65 – 5.49 (m, 1 H), 4.96 – 4.78 (m, 2 H), 2.15 (d, J = 7.2 Hz, 2 H), 1.71 (s, 6 H).

^{13}C-NMR (CDCl$_3$, 101 MHz, ppm): δ = 135.0, 134.3, 133.6, 128.8, 127.5, 116.4, 54.1, 42.4, 40.3, 36.5.

MS (70 eV, EI) *m/z* (%): 216 (10) $[M]^{\cdot+}$, 188 (22), 173 (22), 147 (16), 142 (11), 141 (11), 135 (31), 134 (14), 123 (11), 111 (10), 110 (21), 109 (16), 107 (35), 105 (27), 91 (100), 79 (87), 77 (16), 10 (65).

IR (ATR) $\tilde{\nu}$ (cm^{-1}): 3075 (vw), 2975 (w), 2907 (w), 2870 (w), 1640 (vw), 1583 (vw), 1473 (w), 1438 (w), 1272 (w), 1188 (vs), 1131 (vw), 1091 (vw), 1066 (vw), 1024 (w), 1011 (w), 991 (w), 912 (m), 891 (s), 741 (vs), 690 (vs).

HRMS (EI) calculated for $C_{14}H_{16}S^{\cdot+}$: 216.0967, found 216.0966 $[M]^{\cdot+}$.

N,N,3-Triallylbicyclo[1.1.1]pentan-1-amine (9g)

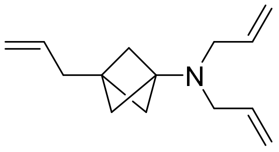

N,*N*,*3*-Triallylbicyclo[1.1.1]pentan-1-amine was prepared according to **TP1** using allylzinc bromide coordinated with lithium chloride (0.90 M, 0.44 mL, 0.40 mmol, 2.0 equiv). The reaction was stirred at 50 °C for 3 h before adding $CoCl_2$ (0.8 mg, 0.006 mmol, 3 mol%.) and *N,N*-diallyl-*O*-benzoylhydroxylamine (109 mg, 0.50 mmol, 2.5 equiv). The resulting mixture was stirred at 45 °C for 17 h. Workup according to **TP1** and purification via column chromatography (*i*Hex / EtOAc = 9 / 1) afforded the desired compound **9g** (37 mg, 0.18 mmol, 91%) as a colorless liquid.

^{1}H-NMR ($CDCl_3$, 400 MHz, ppm): δ = 5.85 (ddt, J = 16.8, 10.1, 6.5 Hz, 2 H), 5.70 (ddt, J = 17.2, 10.1, 7.1 Hz, 1 H), 5.18 – 5.04 (m, 4 H), 5.04 – 4.92 (m, 2 H), 3.15 (dt, J = 6.5, 1.4 Hz, 4 H), 2.26 (dt, J = 7.1, 1.3 Hz, 2 H), 1.65 (s, 6 H).

^{13}C-NMR ($CDCl_3$, 101 MHz, ppm): δ = 136.5, 135.9, 117.0, 115.8, 57.8, 53.0, 50.1, 35.6, 34.9.

MS (70 eV, EI) *m/z* (%): 202 (46) $[M–H]^{\cdot+}$, 188 (43), 174 (57), 172 (10), 163 (11), 162 (93), 160 (62), 157 (10), 148 (23), 147 (16), 146 (53), 136 (22), 134 (55), 132 (54), 122 (36), 121 (21), 120 (100), 118 (31), 106 (31), 105 (25), 94 (32), 93 (26), 91 (75), 81 (21), 80 (28), 79 (84), 77 (42), 67 (21), 41 (71).

IR (ATR) $\tilde{\nu}$ (cm^{-1}): 3075 (vw), 2961 (m), 2923 (m), 2867 (w), 1858 (vw), 1783 (m), 1722 (m), 1641 (w), 1446 (w), 1418 (m), 1379 (w), 1277 (m), 1237 (s), 1113 (w), 1047 (s), 993 (s), 913 (vs), 807 (w), 712 (m).

HRMS (EI) calculated for $C_{14}H_{20}N^{\cdot+}$: 202.1590, found 202.1590 $[M–H]^{\cdot+}$.

Ethyl 2-((3-(1-phenylallyl)bicyclo[1.1.1]pentan-1-yl)methyl)acrylate (9h)

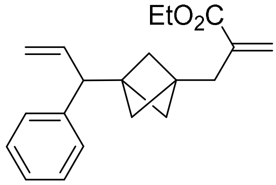

Ethyl 2-((3-(1-phenylallyl)bicyclo[1.1.1]pentan-1-yl)methyl)acrylate was prepared according to **TP1** using cinnamylzinc bromide coordinated with lithium chloride (0.53 M, 0.75 mL,

0.40 mmol, 2.0 equiv). The reaction was stirred at 50 °C for 7 h before adding CuCN·2LiCl in THF (1.0 M, 0.04 mL, 0.04 mmol, 20 mol%) and ethyl 2-(bromomethyl)acrylate (97 mg, 0.50 mmol, 2.5 equiv). The resulting mixture was stirred at 25 °C for 16 h. Workup according to **TP1** and purification via column chromatography (*i*Hex / EtOAc = 99 / 1) and HPLC afforded the desired compound **9h** (54 mg, 0.19 mmol, 93%) as a colorless liquid.

^{1}H-NMR ($CDCl_3$, 400 MHz, ppm): δ = 7.28 (t, J = 7.3 Hz, 2 H), 7.19 (tt, J = 7.3, 2.1 Hz, 1 H), 7.12 (d, J = 6.7 Hz, 2 H), 6.12 (d, J = 1.7 Hz, 1 H), 6.05 (ddd, J = 17.0, 10.3, 8.3 Hz, 1 H), 5.42 (dt, J = 1.8, 1.0 Hz, 1 H), 5.11 – 4.97 (m, 2 H), 4.17 (q, J = 7.1 Hz, 2 H), 3.38 (d, J = 8.3 Hz, 1 H), 2.48 (d, J = 0.9 Hz, 2 H), 1.46 (A part of an $[AB]_3$-system, 3 H), 1.41 (B part of an $[AB]_3$-system, 3 H), 1.28 (t, J = 7.1 Hz, 3 H).

^{13}C-NMR ($CDCl_3$, 101 MHz, ppm): δ = 167.3, 142.2, 138.6, 138.5, 128.3, 128.0, 126.1, 125.8, 115.6, 60.7, 52.0, 49.2, 42.5, 39.5, 34.9, 14.3.

MS (70 eV, EI) *m/z* (%): 223 (5) $[M–CO_2Et]^{·+}$, 207 (19), 193 (14), 192 (11), 184 (11), 183 (79), 181 (25), 179 (22), 178 (16), 168 (38), 167 (40), 166 (15), 165 (33), 155 (56), 154 (10), 153 (26), 142 (19), 141 (86), 133 (35), 131 (23), 129 (42), 128 (59), 117 (65), 116 (10), 115 (100), 107 (11), 105 (97), 103 (13), 91 (83), 79 (30), 77 (15).

IR (ATR) $\tilde{\nu}$ (cm^{-1}): 2964 (w), 2905 (w), 2867 (w), 1715 (s), 1631 (w), 1601 (vw), 1493 (vw), 1445 (w), 1368 (w), 1308 (w), 1251 (s), 1234 (s), 1155 (vs), 111 (m), 1094 (m), 1027 (m), 943 (w), 914 (m), 859 (w), 821 (w), 755 (w), 699 (vs).

HRMS (EI) calculated for $C_{19}H_{21}O_2^{·+}$: 281.1536, found 281.1533 $[M–CH_3]^{·+}$.

Methyl(3-(1-phenylallyl)bicyclo[1.1.1]pentan-1-yl)sulfane (9i)

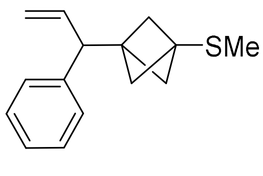

Methyl(3-(1-phenylallyl)bicyclo[1.1.1]pentan-1-yl)sulfane was prepared according to **TP1** using cinnamylzinc bromide coordinated with lithium chloride (0.53 M, 0.75 mL, 0.40 mmol, 2.0 equiv). The reaction was stirred at 50 °C for 7 h before adding *S*-methyl benzene-sulfonothioate (94 mg, 0.50 mmol, 2.5 equiv). The resulting mixture was stirred at 25 °C for 16 h. Workup according to **TP1** and purification via column chromatography (*i*Hex / EtOAc = 99 / 1) afforded the desired compound **9i** (41 mg, 0.18 mmol, 90%) as a colorless liquid.

^{1}H-NMR (CDCl$_3$, 400 MHz, ppm): δ = 7.31 (t, J = 7.3 Hz, 2 H), 7.22 (tt, J = 7.3, 2.1 Hz, 1 H), 7.14 (d, J = 6.8 Hz, 2 H), 6.06 (ddd, J = 17.0, 10.2, 8.3 Hz, 1 H), 5.17 – 5.02 (m, 2 H), 3.47 (d, J = 8.4 Hz, 1 H), 2.03 (s, 3 H), 1.77 (A part of an [AB]$_3$-system, 3 H), 1.72 (B part of an [AB]$_3$-system, 3 H).

^{13}C-NMR (CDCl$_3$, 101 MHz, ppm): δ = 141.6, 137.9, 128.4, 127.9, 126.4, 116.3, 51.8, 51.6, 43.6, 41.8, 13.7.

MS (70 eV, EI) *m/z* (%): 215 (17), 202 (30), 187 (12), 183 (21), 181 (24), 168 (18), 167 (89), 166 (17), 165 (28), 156 (12), 155 (100), 154 819), 153 (33), 152 (19), 142 (11), 141 (95), 129 (15), 128 (30), 117 (12), 115 (59), 91 (37).

IR (ATR) $\tilde{\nu}$ (cm^{-1}): 3027 (vw), 2975 (w), 2906 (w), 2870 (w), 1637 (vw), 1601 (vw), 1492 (vw), 1451 (w), 1317 (vw), 1259 (vw), 1195 (vs), 1131 (vw), 1070 (vw), 1030 (w), 990 (w), 960 (w), 915 (m), 895 (w), 840 (vw), 754 (m), 715 (s), 698 (vs), 660 (w).

HRMS (EI) calculated for $C_{15}H_{17}S^{\cdot+}$: 229.1045, found 229.1043 [M–H]$^{\cdot+}$.

1-(3-(Cyclohex-2-en-1-yl)bicyclo[1.1.1]pentan-1-yl)propan-1-one (9j)

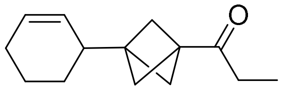

1-(3-(Cyclohex-2-en-1-yl)bicyclo[1.1.1]pentan-1-yl)propan-1-one was prepared according to **TP1** using cyclohex-2-en-1-ylzinc bromide coordinated with lithium chloride (0.34 M, 1.18 mL, 0.40 mmol, 2.0 equiv). The reaction was stirred at 50 °C for 6 h before adding CuCN·2LiCl in THF (1.0 M, 0.20 mL, 0.20 mmol, 1.0 equiv) and propionyl chloride (46 mg, 0.50 mmol, 2.5 equiv). The resulting mixture was stirred at 25 °C for 1 h. Workup according to **TP1** and purification via column chromatography (*i*Hex / EtOAc = 49 / 1) and HPLC afforded the desired compound **9j** (38 mg, 0.19 mmol, 94%) as a colorless liquid.

^{1}H-NMR (CDCl$_3$, 400 MHz, ppm): δ = 5.73 (dtd, J = 10.0, 3.7, 2.6 Hz, 1 H), 5.54 – 5.46 (m, 1 H), 2.46 (q, J = 7.3 Hz, 2 H), 2.22 – 2.13 (m, 1 H), 1.98 – 1.91 (m, 2 H), 1.89 – 1.80 (([AB]$_3$-system, 6 H), 1.71 – 1.64 (m, 2 H), 1.55 – 1.44 (m, 1 H), 1.31 – 1.20 (m, 1 H), 1.02 (t, J = 7.3 Hz, 3 H).

^{13}C-NMR (CDCl$_3$, 101 MHz, ppm): δ = 209.7, 128.4, 127.5, 49.6, 43.9, 42.4, 36.1, 32.0, 25.5, 25.1, 21.3, 7.5.

MS (70 eV, EI) *m/z* (%): 203 (2) $[M–H]^{·+}$, 189 (12), 176 (41), 175 (45), 161 (65), 143 (33), 133 (18), 117 (51), 105 (76), 91 (100), 77 (94).

IR (ATR) $\tilde{\nu}$ (cm^{-1}): 3349 (vw), 2977 (w), 2913 (w), 2876 (w), 1694 (s), 1653 (m), 1509 (vw), 1449 (w), 1408 (w), 1361 (w), 1268 (w), 1170 (m), 1020 (w), 957 (m), 890 (w), 823 (w), 733 (vs), 702 (m).

HRMS (EI) calculated for $C_{14}H_{19}O^{·+}$: 203.1430, found 203.1431 $[M–H]^{·+}$.

(3-(2-Methylbut-3-en-2-yl)bicyclo[1.1.1]pentan-1-yl)(phenyl)methanone (9k)

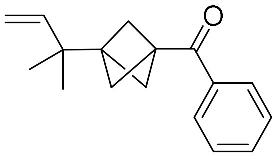

(3-(2-Methylbut-3-en-2-yl)bicyclo[1.1.1]pentan-1-yl)(phenyl)methanone was prepared according to **TP1** using prenylzinc bromide coordinated with lithium chloride (0.61 M, 0.66 mL, 0.40 mmol, 2.0 equiv). The reaction was stirred at 50 °C for 17 h before adding CuCN·2LiCl in THF (1.0 M, 0.20 mL, 0.20 mmol, 1.0 equiv) and benzoyl chloride (70 mg, 0.50 mmol, 2.5 equiv). The resulting mixture was stirred at 25 °C for 1 h. Workup according to **TP1** and purification via column chromatography (*i*Hex / EtOAc = 99 / 1) and HPLC afforded the desired compound **9k** (47 mg, 0.19 mmol, 97%) as a colorless liquid.

^{1}H-NMR (CDCl$_3$, 400 MHz, ppm): δ = 8.02 – 7.94 (m, 2 H), 7.54 (tt, J = 7.4, 1.3 Hz, 1 H), 7.44 (t, J = 7.6 Hz, 2 H), 5.81 (dd, J = 17.3, 10.9 Hz, 1 H), 5.03 – 4.93 (m, 2 H), 2.07 (s, 6 H), 1.00 (s, 6 H).

^{13}C-NMR (CDCl$_3$, 101 MHz, ppm): δ = 198.5, 144.6, 136.8, 132.9, 129.0, 128.5, 111.8, 50.2, 47.3, 42.7, 36.0, 22.9.

MS (70 eV, EI) *m/z* (%): 225 (14) $[M–CH_3]^{+}$, 171 (41), 153 (12), 143 (15), 128 (22), 105 (100), 93 (10), 91 (18), 77 (41).

IR (ATR) $\tilde{\nu}$ (cm^{-1}): 2965 (w), 2910 (vw), 2874 (w), 1663 (s), 1638 (w), 1598 (w), 1579 (w), 1509 (vw), 1461 (vw), 1448 (m), 1414 (vw), 1377 (vw), 1360 (w), 1331 (s), 1280 (w), 1205 (vs), 1177 (m), 1134 (vw), 1055 (vw), 1025 (w), 1001 (w), 913 (s), 872 (s), 820 (vw), 765 (s), 694 (vs), 676 (s).

HRMS (EI) calculated for $C_{17}H_{19}O^{·+}$: 239.1430, found 239.1428 $[M–H]^{·+}$.

(3-(3,7-Dimethylocta-1,6-dien-3-yl)bicyclo[1.1.1]pentan-1-yl)(phenyl)methanone (9l)

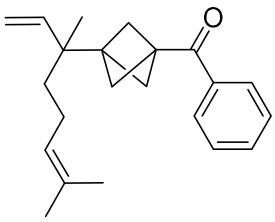

(3-(3,7-Dimethylocta-1,6-dien-3-yl)bicyclo[1.1.1]pentan-1-yl)(phenyl)methanone was prepared according to **TP1** using geranylzinc bromide coordinated with lithium chloride (0.40 M, 5.0 mL, 2.0 mmol, 2.0 equiv) and [1.1.1]propellane dissolved in diethyl ether (0.43 M, 2.3 mL, 1.0 mmol, 1.0 equiv). The reaction was stirred at 50 °C for 6 h before adding CuCN·2LiCl in THF (1.0 M, 1.0 mL, 1.0 mmol, 1.0 equiv) and benzoyl chloride (353 mg, 2.5 mmol, 2.5 equiv). The resulting mixture was stirred at 25 °C for 17 h. Workup according to **TP1** and purification via column chromatography (*i*Hex / EtOAc = 49 / 1) and HPLC afforded the desired compound **9l** (216 mg, 0.70 mmol, 70%) as a colorless liquid.

^{1}H-NMR ($CDCl_3$, 400 MHz, ppm): δ = 7.99 (d, J = 7.0 Hz, 2 H), 7.54 (tt, J = 7.5, 2.0 Hz, 1 H), 7.43 (t, J = 7.6 Hz, 2 H), 5.67 (dd, J = 17.5, 10.8 Hz, 1 H), 5.16 – 5.04 (m, 2 H), 4.96 (dd, J = 17.5, 1.4 Hz, 1 H), 2.08 ($[AB]_3$-system, 6 H), 1.94 – 1.78 (m, 2 H), 1.69 (d, J = 1.5 Hz, 3 H), 1.59 (d, J = 1.4 Hz, 3 H), 1.41 – 1.24 (m, 2 H), 0.98 (s, 3 H).

^{13}C-NMR ($CDCl_3$, 101 MHz, ppm): δ = 198.5, 142.9, 136.8, 132.9, 131.5, 129.0, 128.5, 124.9, 113.5, 50.3, 47.5, 43.0, 39.2, 36.9, 25.9, 23.1, 17.8, 17.7.

MS (70 eV, EI) *m/z* (%): 211 (7), 171 (10), 128 (10), 119 (12), 105 (100), 93 (9), 91 (19), 77 (30).

IR (ATR) $\tilde{\nu}$ (cm^{-1}): 2970 (w), 2913 (w), 2874 (w), 1664 (s), 1598 (w), 1579 (w), 1508 (vw), 1448 (m), 1412 (vw), 1373 (w), 1205 (vs), 1176 (m), 1143 (w), 1025 (w), 1001 (w), 912 (m), 873 (s), 843 (vw), 765 (m), 694 (vs), 676 (s).

HRMS (EI) calculated for $C_{22}H_{27}O^{\cdot+}$: 307.2056, found 307.2054 $[M{-}H]^{\cdot+}$.

(1*R*,3*R*,5*R*)-3-(3-(4-Methoxyphenyl)bicyclo[1.1.1]pentan-1-yl)-6,6-dimethyl-2-methylene-bicyclo[3.1.1]heptane (9m)

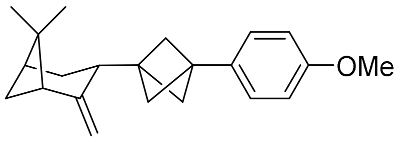

(1*R*,3*R*,5*R*)-3-(3-(4-Methoxyphenyl)bicyclo[1.1.1]pentan-1-yl)-6,6-dimethyl-2-methylenebicyclo[3.1.1]heptane was prepared according to **TP1** using myrtenylzinc bromide coordinated with lithium chloride (0.43 M, 0.93 mL, 0.40 mmol, 2.0 equiv). The reaction was stirred at 50 °C for 8 h before adding 4-iodoanisole (117 mg, 0.50 mmol, 2.5 equiv), $PdCl_2(dppf)\cdot CH_2Cl_2$ (8.2 mg, 0.01 mmol, 5 mol%) and CuCN·2LiCl in THF (1.0 M, 0.02 mL, 0.02 mmol, 10 mol%). The resulting mixture was stirred at 45 °C for 15 h. Workup according to **TP1** and purification via column chromatography (*i*Hex / EtOAc = 99 / 1) and HPLC afforded the desired compound **9m** (46 mg, 0.15 mmol, 75%) as colorless crystals. The structure was confirmed via single crystal X-ray diffraction studies.

^{1}H-NMR ($CDCl_3$, 400 MHz, ppm): δ = 7.17 (d, J = 8.6 Hz, 2 H), 6.85 (d, J = 8.6 Hz, 2 H), 4.71 (dt, J = 22.0, 1.9 Hz, 2 H), 3.80 (s, 3 H), 2.72 – 2.66 (m, 1 H), 2.42 (t, J = 5.4 Hz, 1 H), 2.33 – 2.24 (m, 1 H), 2.05 – 1.95 (m, 2 H), 1.90 (s, 6 H), 1.78 (dt, J = 14.2, 3.5 Hz, 1 H), 1.26 (s, 3 H), 1.19 (d, J = 9.9 Hz, 1 H), 0.77 (s, 3 H).

^{13}C-NMR ($CDCl_3$, 101 MHz, ppm): δ = 158.2, 152.1, 134.1, 127.2, 113.6, 109.0, 55.4, 52.1, 51.1, 43.5, 41.7, 40.5, 40.4, 36.2, 27.6, 27.4, 26.0, 21.7.

MS (70 eV, EI) *m/z* (%): 308 (2) $[M]^{+}$, 265 (12), 237 (18), 223 (13), 211 (11), 209 (12), 197 (11), 185 (12), 173 (26), 171 (12), 165 (12), 159 (11), 158 (24), 157 (71), 148 (15), 147 (13), 145 (13), 143 (20), 142 (36), 141 (13), 135 (54), 134 (10), 133 (40), 131 (41), 130 (10), 129 (100), 128 (18), 121 (86), 117 (20), 115 (32), 105 (19), 91 (33), 79 (11), 77 (10).

IR (ATR) $\tilde{\nu}$ (cm^{-1}): 2948 (m), 1918 (m), 2865 (m), 1636 (w), 1608 (w), 1578 (vw), 1519 (m), 1501 (m), 1455 (m), 1380 (w), 1366 (w), 1345 (vw), 1293 (m), 1259 (m), 1244 (vs), 1175 (s), 1159 (m), 1132 (w), 1098 (w), 1034 (vs), 934 (vw), 878 (s), 857 (w), 835 (vs), 822 (s), 791 (vs), 697 (w).

HRMS (EI) calculated for $C_{22}H_{28}O^{\cdot+}$: 308.2135, found 308.2136 $[M–H]^{\cdot+}$.

mp: 99.7 - 100.9 °C.

Ethyl 2-((3-((1*R*,3*R*,5*R*)-6,6-dimethyl-2-methylenebicyclo[3.1.1]heptan-3-yl)bicyclo-[1.1.1]pentan-1-yl)methyl)acrylate (9n)

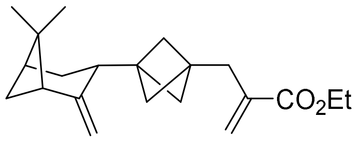

Ethyl 2-((3-((1*R*,3*R*,5*R*)-6,6-dimethyl-2-methylenebicyclo[3.1.1]heptan-3-yl)bicyclo[1.1.1]pentan-1-yl)methyl)acrylate was prepared according to **TP1** using myrtenylzinc bromide coordinated with lithium chloride (0.43 M, 0.93 mL, 0.40 mmol, 2.0 equiv). The reaction was stirred at 50 °C for 8 h before adding CuCN·2LiCl in THF (1.0 M, 0.04 mL, 0.04 mmol, 20 mol%) and ethyl 2-(bromomethyl)acrylate (97 mg, 0.50 mmol, 2.5 equiv). The resulting mixture was stirred at 25 °C for 2 h. Workup according to **TP1** and purification via column chromatography (*i*Hex / EtOAc = 99 / 1) and HPLC afforded the desired compound **9n** (60 mg, 0.19 mmol, 95%) as a colorless liquid.

^{1}H-NMR ($CDCl_3$, 400 MHz, ppm): δ = 6.13 (d, J = 1.7 Hz, 1 H), 5.47 – 5.44 (m, 1 H), 4.65 (t, J = 1.9 Hz, 1 H), 4.57 (t, J = 1.9 Hz, 1 H), 4.19 (q, J = 7.1 Hz, 2 H), 2.58 – 2.52 (m, 1 H), 2.51 (d, J = 0.9 Hz, 2 H), 2.33 (t, J = 5.4 Hz, 1 H), 2.20 (dtd, J = 9.9, 5.9, 2.1 Hz, 1 H), 1.94 – 1.85 (m, 2 H), 1.65 (ddd, J = 14.1, 4.0, 2.8 Hz, 1 H), 1.47 (s, 6 H), 1.29 (t, J = 7.1 Hz, 3 H), 1.21 (s, 3 H), 1.09 (d, J = 9.8 Hz, 1 H), 0.70 (s, 3 H).

^{13}C-NMR ($CDCl_3$, 101 MHz, ppm): δ = 167.5, 152.2, 138.8, 125.7, 108.8, 60.7, 52.9, 52.1, 49.3, 44.5, 40.4, 39.1, 36.3, 35.0, 27.5, 27.3, 26.0, 21.7, 14.4.

MS (70 eV, EI) *m/z* (%): 225 (13), 207 (32), 197 (62), 185 (12), 183 (19), 182 (11), 181 (14), 171 (32), 169 (54), 167 (13), 161 (20), 159 (59), 157 (72), 156 (24), 155 (79), 145 (71), 143 (71), 142 (27), 141 (57), 133 (27), 131 (77), 130 (11), 129 (86), 128 (42), 119 (50), 117 (100), 115 (38), 107 (15), 105 (85), 93 (25), 91 (84), 79 (30), 77 (18).

IR (ATR) $\widetilde{\nu}$ (cm^{-1}): 2959 (m), 2906 (m), 2865 (m), 1717 (vs), 1663 (vw), 1534 (w), 1455 (w), 1383 (w), 1367 (m), 1300 (m), 1255 (s), 1236 (s), 1174 (vs), 1154 (vs), 1097 (m), 1027 (m), 940 (m), 881 (m), 856 (w), 820 (w), 729 (w), 696 (vw).

HRMS (EI) calculated for $C_{21}H_{30}O_2^{\cdot+}$: 299.2006, found 299.2004 $[M{-}CH_3]^{\cdot+}$.

Ethyl 6-(3-(quinolin-2-yl)bicyclo[1.1.1]pentan-1-yl)cyclohex-1-ene-1-carboxylate (9o)

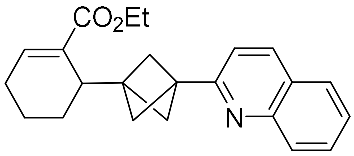

Ethyl 6-(3-(quinolin-2-yl)bicyclo[1.1.1]pentan-1-yl)cyclohex-1-ene-1-carboxylate was prepared according to **TP1** using (2-(ethoxycarbonyl)cyclohex-2-en-1-yl)zinc chloride coordinated with lithium chloride (0.74 M, 0.54 mL, 0.40 mmol, 2.0 equiv). The reaction was stirred at 50 °C for 15 h before adding 2-bromoquinoline (104 mg, 0.50 mmol, 2.5 equiv), $PdCl_2(dppf)\cdot CH_2Cl_2$ (8.2 mg, 0.01 mmol, 5 mol%) and CuCN·2LiCl in THF (1.0 M, 0.02 mL, 0.02 mmol, 10 mol%). The resulting mixture was stirred at 45 °C for 7 h. Workup according to **TP1** and purification via column chromatography (*i*Hex / EtOAc = 9 / 1) and HPLC afforded the desired compound **9o** (45 mg, 0.13 mmol, 65%) as a colorless liquid.

^{1}H-NMR ($CDCl_3$, 400 MHz, ppm): δ = 8.07 (t, J = 8.8 Hz, 2 H), 7.75 (dd, J = 8.1, 1.5 Hz, 1 H), 7.66 (ddd, J = 8.4, 6.9, 1.5 Hz, 1 H), 7.47 (ddd, J = 8.0, 6.8, 1.2 Hz, 1 H), 7.32 (d, J = 8.4 Hz, 1 H), 7.01 (t, J = 3.9 Hz, 1 H), 4.28 – 4.12 (m, 2 H), 2.99 – 2.88 (m, 1 H), 2.30 – 2.03 (m, 8 H), 1.95 – 1.86 (m, 1 H), 1.74 – 1.53 (m, 3 H), 1.31 (t, J = 7.1 Hz, 3 H).

^{13}C-NMR ($CDCl_3$, 101 MHz, ppm): δ = 168.3, 160.4, 147.9, 140.1, 136.3, 131.7, 129.5, 129.2, 127.6, 127.0, 126.0, 119.2, 60.4, 52.2, 42.4, 42.2, 33.2, 25.7, 25.1, 18.0, 14.4.

MS (70 eV, EI) *m/z* (%): 347 (1) $[M]^{\cdot+}$, 274 (5), 195 (14), 194 (100), 193 (8), 192 (8), 180 (8), 167 (10).

IR (ATR) $\tilde{\nu}$ (cm^{-1}): 2967 (w), 2905 (w), 2868 (w), 1707 (s), 1643 (w), 1618 (w), 1599 (m), 1559 (vw), 1514 (vw), 1501 (w), 1446 (w), 1425 (w), 1373 (w), 1345 (vw), 1328 (vw), 1296 (w), 1253 (s), 1235 (vs), 1181 (m), 1139 (w), 1114 (w), 1091 (m), 1061 (s), 1049 (m), 1017 (w), 941 (w), 929 (w), 913 (w), 875 (vw), 837 (m), 802 (w), 786 (m), 749 (vs), 735 (m), 702 (vw), 675 (vw).

HRMS (EI) calculated for $C_{23}H_{25}NO_2^{\cdot+}$: 347.1880, found 347.1879 $[M]^{\cdot+}$.

5-(3-(Cyclohex-2-en-1-yl)bicyclo[1.1.1]pentan-1-yl)cyclopent-1-ene-1-carbonitrile (9p)

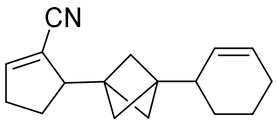

5-(3-(Cyclohex-2-en-1-yl)bicyclo[1.1.1]pentan-1-yl)cyclopent-1-ene-1-carbonitrile was prepared according to **TP1** using (2-cyanocyclopent-2-en-1-yl)zinc bromide coordinated with lithium chloride (0.54 M, 0.74 mL, 0.40 mmol, 2.0 equiv). The reaction was stirred at 25 °C for 17 h before adding CuCN·2LiCl in THF (1.0 M, 0.04 mL, 0.04 mmol, 20 mol%) and 3-bromocyclohex-1-ene (81 mg, 0.50 mmol, 2.5 equiv). The resulting mixture was stirred at 25 °C for 1 h. Workup according to **TP1** and purification via column chromatography (*i*Hex / EtOAc = 49 / 1) and HPLC afforded the desired compound **9p** (26 mg, 0.11 mmol, 55%) as a colorless liquid.

^{1}H-NMR ($CDCl_3$, 400 MHz, ppm): δ = 6.65 (td, J = 2.7, 1.7 Hz, 1 H), 5.75 – 5.67 (m, 1H), 5.52 (dq, J = 10.1, 2.3 Hz, 1 H), 3.02 – 2.94 (m, 1 H), 2.49 – 2.41 (m, 2 H), 2.20 – 2.12 (m, 1 H), 2.06 – 1.98 (m, 1 H), 1.97 – 1.91 (m, 2 H), 1.81 – 1.74 (m, 1 H), 1.61 – 1.49 (m, 8 H), 1.31 – 1.20 (m, 2 H).

^{13}C-NMR ($CDCl_3$, 101 MHz, ppm): δ = 149.6, 128.3, 127.9, 117.5, 117.0, 48.3, 47.1, 42.9, 40.8, 36.4, 32.9, 26.7, 25.7, 25.2, 21.4.

MS (70 eV, EI) *m/z* (%): 238 (19) $[M–H]^{\cdot+}$, 224 (10), 210 (11), 196 (16), 182 (16), 170 (10), 168 (12), 156 (15), 147 (21), 131 (11), 130 (16), 129 13), 119 (29), 117 (16), 116 (13), 115 (16), 107 (32), 105 (86), 93 (18), 92 (15), 91 (100), 81 (12), 79 (99), 78 (11), 77 (22), 65 (12).

IR (ATR) $\tilde{\nu}$ (cm^{-1}): 3425 (vw), 2957 (s), 2924 (s), 2865 (s), 2216 (m), 1727 (w), 1653 (w), 1612 (w), 1446 (m), 1433 (m), 1409 (w), 1304 (w), 1257 (vs), 1227 (w), 1171 (m), 1142 (m), 1020 (w), 950 (m), 895 (w), 869 (w), 822 (w), 740 (m), 721 (s), 668 (w).

HRMS (EI) calculated for $C_{17}H_{20}N^{\cdot+}$: 238.1590, found 238.1590 $[M–H]^{\cdot+}$.

5-(3-(4-Methoxyphenyl)bicyclo[1.1.1]pentan-1-yl)cyclopent-1-ene-1-carbonitrile (9q)

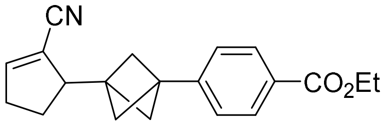

5-(3-(4-Methoxyphenyl)bicyclo[1.1.1]pentan-1-yl)cyclopent-1-ene-1-carbonitrile was prepared according to **TP1** using (2-cyanocyclopent-2-en-1-yl)zinc bromide coordinated with lithium

chloride (0.54 M, 0.74 mL, 0.40 mmol, 2.0 equiv). The reaction was stirred at 25 °C for 17 h before adding 4-iodobenzoate (138 mg, 0.50 mmol, 2.5 equiv), $PdCl_2(dppf)\cdot CH_2Cl_2$ (8.2 mg, 0.01 mmol, 5 mol%) and CuCN·2LiCl in THF (1.0 M, 0.02 mL, 0.02 mmol, 10 mol%). The resulting mixture was stirred at 45 °C for 5 h. Workup according to **TP1** and purification via column chromatography (*i*Hex / EtOAc = 19 / 1) afforded the desired compound **9q** (36 mg, 0.12 mmol, 59%) as a colorless solid.

^{1}H-NMR ($CDCl_3$, 400 MHz, ppm): δ = 7.97 (d, J = 8.3 Hz, 2 H), 7.26 (d, J = 8.3 Hz, 2 H), 6.71 (q, J = 2.6 Hz, 1 H), 4.36 (q, J = 7.1 Hz, 2 H), 3.13 – 3.05 (m, 1 H), 2.56 – 2.47 (m, 2 H), 2.15 – 1.97 (m, 7 H), 1.89 – 1.79 (m, 1 H), 1.38 (t, J = 7.1 Hz, 3 H).

^{13}C-NMR ($CDCl_3$, 101 MHz, ppm): δ = 166.7, 150.0, 145.8, 129.6, 128.7, 126.2, 117.3, 116.5, 61.0, 51.0, 47.9, 42.1, 40.5, 32.9, 26.6, 14.5.

MS (70 eV, EI) *m/z* (%): 307 (1) $[M]^{\cdot+}$, 281 (27), 262 (17), 261 (36), 253 (13), 246 (18), 234 (47), 233 (19), 232 (10), 225 (16), 219 (11), 218 (15), 215 (23), 208 (13), 207 (100), 206 (13), 191 (18), 177 (32), 169 (17), 162 (34), 145 (55), 143 (55), 142 (19), 141 (36), 131 (10), 129 (18), 128 (47), 117 (11), 115 (40), 91 (18).

IR (ATR) $\tilde{\nu}$ (cm^{-1}): 2968 (w), 2945 (w), 2906 (w), 2868 (w), 2214 (w), 1714 (s), 1608 (w), 1467 (vw), 1451 (vw), 1428 (w), 1407 (w), 1367 (w), 1307 (w), 1294 (w), 1268 (vs), 1212 (vw), 1176 (m), 1146 (w), 1130 (w), 1104 (s), 1092 (vs), 1045 (w), 1017 (m), 978 (w), 944 (w), 923 (vw), 904 (vw), 875 (w), 860 (m), 850 (w), 815 (w), 800 (w), 761 (s), 719 (vw), 700 (s).

HRMS (EI) calculated for $C_{20}H_{21}NO_2^{\cdot+}$: 307.1567, found 307.1568 $[M]^{\cdot+}$.

mp: 84.1 – 85.9 °C.

Phenyl(3-(1-(triisopropylsilyl)propa-1,2-dien-1-yl)bicyclo[1.1.1]pentan-1-yl)methanone (9r) and phenyl(3-(3-(triisopropylsilyl)prop-2-yn-1-yl)bicyclo[1.1.1]pentan-1-yl)methanone (9s)

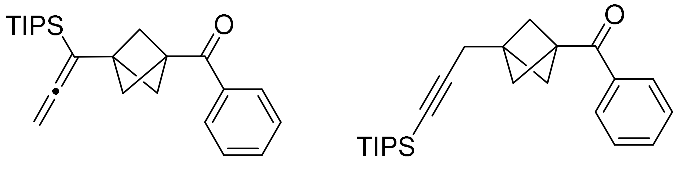

Phenyl(3-(1-(triisopropylsilyl)propa-1,2-dien-1-yl)bicyclo[1.1.1]pentan-1-yl)methanone and phenyl(3-(3-(triisopropylsilyl)prop-2-yn-1-yl)bicyclo[1.1.1]pentan-1-yl)methanone were prepared according to **TP1** using (3-(triisopropylsilyl)prop-2-yn-1-yl)zinc bromide coordinated with

lithium chloride (0.24 M, 1.67 mL, 0.40 mmol, 2.0 equiv). The reaction was stirred at 50 °C for 19 h before adding CuCN·2LiCl in THF (1.0 M, 0.20 mL, 0.20 mmol, 1.0 equiv) and benzoyl chloride (70 mg, 0.50 mmol, 2.5 equiv). The resulting mixture was stirred at 25 °C for 1 h. Workup according to **TP1** and purification via column chromatography (*i*Hex / EtOAc = 99 / 1) and HPLC afforded phenyl(3-(1-(triisopropylsilyl)propa-1,2-dien-1-yl)bicyclo-[1.1.1]pentan-1-yl)methanone **9r** (37 mg, 0.10 mmol, 50%) as a colorless solid and phenyl(3-(3-(triisopropylsilyl)-prop-2-yn-1-yl)bicyclo[1.1.1]pentan-1-yl)methanone **9s** (33 mg, 0.09 mmol, 45%) as a colorless liquid.

Phenyl(3-(1-(triisopropylsilyl)propa-1,2-dien-1-yl)bicyclo[1.1.1]pentan-1-yl)methanone (9r):

^{1}H-NMR ($CDCl_3$, 400 MHz, ppm): δ = 8.02 – 7.95 (m, 2 H), 7.54 (tt, J = 7.3, 1.3 Hz, 1 H), 7.44 (t, J = 7.6 Hz, 2 H), 4.44 (s, 2 H), 2.35 (s, 6 H), 1.28 – 1.15 (m, 3 H), 1.13 – 1.04 (m, 18 H).

^{13}C-NMR ($CDCl_3$, 101 MHz, ppm): δ = 212.7, 198.1, 136.8, 133.0, 129.0, 128.6, 91.1, 69.5, 56.3, 44.6, 39.8, 18.8, 12.0.

MS (70 eV, EI) *m/z* (%): 324 (17), 323 (82) $[M{-}C_3H_7]^{\cdot+}$, 281 (16), 253 (12), 225 (21), 209 (11), 208 (10), 207 (70), 193 (31), 191 (15), 179 (15), 178 (100), 165 (21), 152 (14), 131 (19), 115 (11), 105 (11), 103 (55), 77 (12), 75 (87), 73 (16), 61 (14), 59 (10).

IR (ATR) $\widetilde{\nu}$ (cm^{-1}): 2955 (m), 2940 (m), 2863 (m), 2176 (vw), 1922 (w), 1665 (s), 1598 (vw), 1578 (vw), 1505 (vw), 1460 (m), 1446 (w), 1381 (vw), 1364 (vw), 1332 (m), 1312 (w), 1268 (w), 1209 (s), 1176 (w), 1070 (w), 1018 (w), 990 (w), 941 (m), 920 (w), 882 (vs), 839 (w), 822 (vw), 805 (vs), 766 (s), 699 (s), 674 (vs).

HRMS (EI) calculated for $C_{24}H_{33}OSi^{\cdot+}$: 365.2295, found 365.2298 $[M{-}H]^{\cdot+}$.

mp: 77.7 – 79.2 °C.

Phenyl(3-(3-(triisopropylsilyl)prop-2-yn-1-yl)bicyclo[1.1.1]pentan-1-yl)methanone (9s):

^{1}H-NMR ($CDCl_3$, 400 MHz, ppm): δ = 8.02 – 7.95 (m, 2 H), 7.55 (tt, J = 7.4, 1.2 Hz, 1 H), 7.44 (t, J = 7.6 Hz, 2 H), 2.54 (s, 2 H), 2.24 (s, 6 H), 1.11 – 1.02 (m, 21 H).

^{13}C-NMR ($CDCl_3$, 101 MHz, ppm): δ = 197.7, 136.7, 133.0, 129.0, 128.6, 104.8, 82.1, 53.5, 44.3, 38.4, 23.7, 18.8, 11.4.

MS (70 eV, EI) *m/z* (%): 324 (23), 323 (100) $[M{-}C_3H_7]^{\cdot+}$, 295 (29), 281 (11), 233 (12), 207 (20), 193 (44), 191 (17), 179 (16), 178 (79), 165 (24), 153 (10), 152 (11), 145 (10), 129 (13), 128 (13), 115 (17), 105 (30), 91 (13), 77 (17), 75 (36), 61 (11).

IR (ATR) $\tilde{\nu}$ (cm^{-1}): 2941 (m), 2864 (m), 2172 (w), 1728 (vw), 1666 (s), 1598 (vw), 1580 (vw), 1511 (vw), 1462 (w), 1448 (w), 1419 (vw), 1382 (vw), 1338 (s), 1297 (w), 1266 (w), 1205 (s), 1176 (w), 1086 (w), 1070 (w), 1027 (w), 1011 (w), 994 (w), 926 (w), 881 (s), 760 (w), 694 (vs), 675 (vs), 659 (vs).

HRMS (EI) calculated for $C_{24}H_{34}OSi^{\cdot+}$: 366.2373, found 366.2380 $[M]^{\cdot+}$.

2-(Bicyclo[1.1.1]pentan-1-yl)cyclohexan-1-one (14a)

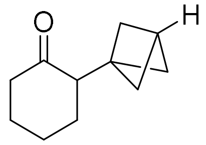

2-(Bicyclo[1.1.1]pentan-1-yl)cyclohexan-1-one was prepared according to **TP2** using cyclopentanone (29 mg, 0.30 mmol, 1.5 equiv). The reaction was stirred at 0 °C for 30 min before adding a saturated aqueous solution of NH_4Cl (1 mL). The resulting mixture was stirred at 25 °C for 10 min. Workup according to **TP2** and purification via column chromatography (*i*Hex / EtOAc = 49 / 1) afforded the desired compound **14a** (29 mg, 0.17 mmol, 87%) as a colorless liquid.

^{1}H-NMR (CDCl$_3$, 400 MHz, ppm): δ = 2.48 (s, 1 H), 2.36 (dd, J = 8.4, 5.8 Hz, 1 H), 2.27 (dd, J = 7.3, 5.8 Hz, 2 H), 1.97 – 1.72 (m, 10 H), 1.66 – 1.56 (m, 2 H).

^{13}C-NMR (CDCl$_3$, 101 MHz, ppm): δ = 212.3, 51.6, 50.2, 45.0, 42.2, 30.3, 28.0, 27.3, 23.6.

MS (70 eV, EI) *m/z* (%): 163 (3) $[M–H]^{\cdot+}$, 149 (42), 135 (43), 131 (13), 121 (11), 117 (10), 107 (15), 105 (54), 95 (17), 93 (89), 92 (22), 91 (80), 81 (12), 79 (100), 77 (42), 67 (32).

IR (ATR) $\tilde{\nu}$ (cm^{-1}): 2958 (s), 2939 (s), 2865 (s), 2175 (vw), 2006 (vw), 1921 (vw), 1708 (vs), 1665 (m), 1598 (vw), 1578 (vw), 1447 (m), 1365 (w), 1333 (w), 1265 (w), 1196 (s), 1125 (w), 1070 (w), 1018 (w), 990 (w), 941 (w), 883 (m), 837 (w), 805 (m), 766 (w), 699 (m), 674 (m).

HRMS (EI) calculated for $C_{11}H_{15}O^{\cdot+}$: 163.1117, found 163.1116 $[M–H]^{\cdot+}$.

2-(3-Allylbicyclo[1.1.1]pentan-1-yl)cyclohexan-1-one (14b)

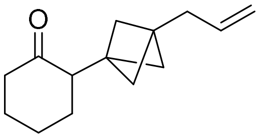

2-(3-Allylbicyclo[1.1.1]pentan-1-yl)cyclohexan-1-one was prepared according to **TP2** using cyclopentanone (29 mg, 0.30 mmol, 1.5 equiv). The reaction was stirred at 0 °C for 30 min before adding CuCN·2LiCl in THF (1.0 M, 0.04 mL, 0.04 mmol, 20 mol%) and allyl bromide (85 mg, 0.70 mmol, 3.5 equiv). The resulting mixture was stirred at 25 °C for 2 h. Workup according to **TP2** and purification via column chromatography (*i*Hex / EtOAc = 49 / 1) afforded the desired compound **14b** (36 mg, 0.18 mmol, 88%) as a colorless liquid.

^{1}H-NMR ($CDCl_3$, 400 MHz, ppm): δ = 5.70 (ddt, J = 16.7, 10.4, 7.2 Hz, 1 H), 5.01 – 4.93 (m, 2 H), 2.38 (dd, J = 8.7, 5.8 Hz, 1 H), 2.27 (dd, J = 7.1, 6.0 Hz, 2 H), 2.20 (dt, J = 7.1, 1.3 Hz, 2 H), 1.97 – 1.70 (m, 4 H), 1.64 – 1.53 (m, 8 H).

^{13}C-NMR ($CDCl_3$, 101 MHz, ppm): δ = 212.3, 135.8, 115.8, 51.0, 50.0, 42.2, 40.0, 39.1, 36.9, 30.5, 27.4, 23.7.

MS (70 eV, EI) *m/z* (%): 189 (11) $[M–CH_3]^{\cdot+}$, 164 (12), 163 (100), 145 (13), 143 (13), 135 (13), 133 (10), 129 (12), 119 (21), 117 (18), 107 (24), 106 (11), 105 (52), 93 (20), 91 (86), 79 (44), 77 (21), 67 (10).

IR (ATR) $\widetilde{\nu}$ (cm^{-1}): 2937 (m), 2866 (m), 1705 (vs), 1640 (w), 1447 (w), 1425 (w), 1370 (w), 1316 (w), 1255 (s), 1218 (m), 1146 (m), 1124 (s), 1066 (m), 991 (m), 946 (m), 910 (s), 652 (m).

HRMS (EI) calculated for $C_{14}H_{19}O^{\cdot+}$: 203.1430, found 203.1429 $[M–H]^{\cdot+}$.

2-(3-(4-Methoxyphenyl)bicyclo[1.1.1]pentan-1-yl)cyclohexan-1-one (14c)

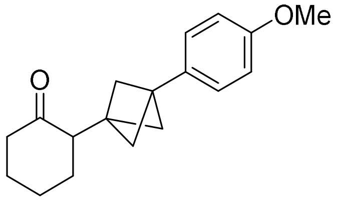

2-(3-(4-Methoxyphenyl)bicyclo[1.1.1]pentan-1-yl)cyclohexan-1-one was prepared according to **TP2** using cyclopentanone (29 mg, 0.30 mmol, 1.5 equiv). The reaction was stirred at 0 °C for 30 min before adding 4-iodoanisole (164 mg, 0.70 mmol, 3.5 equiv), $PdCl_2(dppf)·CH_2Cl_2$ (8.2 mg, 0.01 mmol, 5 mol%) and CuCN·2LiCl in THF (1.0 M, 0.02 mL, 0.02 mmol, 10 mol%).

The resulting mixture was stirred at 40 °C for 3 h. Workup according to **TP2** and purification via column chromatography (*i*Hex / EtOAc = 19 / 1) afforded the desired compound **14c** (42 mg, 0.16 mmol, 78%) as a colorless solid. The structure was confirmed via single crystal X-ray diffraction studies.

^{1}H-NMR ($CDCl_3$, 400 MHz, ppm): δ = 7.14 (d, J = 8.7 Hz, 2 H), 6.83 (d, J = 8.8 Hz, 2 H), 3.78 (s, 3 H), 2.49 (dd, J = 9.2, 5.6 Hz, 1 H), 2.32 (dd, J = 7.4, 5.7 Hz, 2 H), 2.06 – 1.58 (m, 12 H).

^{13}C-NMR ($CDCl_3$, 101 MHz, ppm): δ = 212.2, 158.3, 133.6, 127.2, 113.6, 55.4, 51.9, 50.7, 42.4, 41.8, 38.5, 30.6, 27.4, 24.0.

MS (70 eV, EI) *m/z* (%): 270 (1) $[M]^{·+}$, 256 (19), 255 (100), 199 (8), 173 (51), 172 (18), 171 (10), 163 (8), 158 (39), 141 (7), 133 (9), 128 (7), 115 (7).

IR (ATR) $\tilde{\nu}$ (cm^{-1}): 2934 (m), 2867 (m), 1704 (s), 1608 (w), 1576 (vw), 1518 (m), 1503 (m), 1460 (w), 1442 (m), 1373 (vw), 1343 (w), 1315 (m), 1292 (m), 1266 (w), 1243 (vs), 1211 (m), 1185 (w), 1172 (s), 1163 (m), 1148 (w), 1133 (m), 1123 (m), 1067 (w), 1034 (vs), 953 (w), 923 (w), 905 (w), 889 (w), 862 (w), 846 (m), 829 (vs), 791 (m), 783 (s), 720 (w), 702 (w), 659 (w).

HRMS (EI) calculated for $C_{18}H_{22}O_2^{·+}$: 270.1614, found 270.1617 $[M]^{·+}$.

mp: 120.0 – 121.8 °C.

2-(3-Benzoylbicyclo[1.1.1]pentan-1-yl)cyclohexan-1-one (14d)

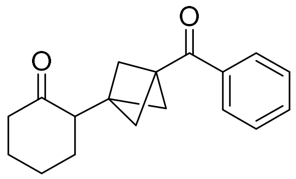

2-(3-Benzoylbicyclo[1.1.1]pentan-1-yl)cyclohexan-1-one was prepared according to **TP2** using cyclopentanone (29 mg, 0.30 mmol, 1.5 equiv). The reaction was stirred at 0 °C for 30 min before adding CuCN·2LiCl in THF (1.0 M, 0.02 mL, 0.02 mmol, 10 mol%) and benzoyl chloride (141 mg, 1.0 mmol, 5.0 equiv). The resulting mixture was stirred at 25 °C for 1 h. Workup according to **TP2** and purification via column chromatography (*i*Hex / EtOAc = 9 / 1) and HPLC afforded the desired compound **14d** (30 mg, 0.15 mmol, 73%) as a colorless solid.

^{1}H-NMR ($CDCl_3$, 400 MHz, ppm): δ = 8.04 – 7.98 (m, 2 H), 7.54 (tt, J = 7.3, 1.2 Hz, 1 H), 7.44 (t, J = 7.5 Hz, 2 H), 2.46 (dd, J = 10.9, 5.6 Hz, 1 H), 2.36 – 2.25 (m, 8 H), 2.11 – 1.96 (m, 2 H), 1.91 – 1.83 (m, 1 H), 1.78 – 1.62 (m, 2 H), 1.59 – 1.47 (m, 1 H).

^{13}C-NMR (CDCl$_3$, 101 MHz, ppm): δ = 211.7, 197.7, 136.6, 133.0, 129.1, 128.5, 53.0, 50.3, 45.0, 42.5, 40.2, 30.6, 27.5, 24.4.

MS (70 eV, EI) *m/z* (%): 198 (19) [M–C_4H_6O]$^{·+}$, 179 (12), 171 (17), 163 (15), 123 (47), 105 (100), 91 (19), 77 (37).

IR (ATR) $\tilde{\nu}$ (cm^{-1}): 2981 (w), 2928 (w), 2875 (w), 1706 (vs), 1659 (vs), 1595 (w), 1578 (w), 1509 (vw), 1446 (m), 1374 (w), 1333 (m), 1313 (w), 1284 (m), 1203 (vs), 1171 (m), 1123 (w), 1106 (w), 1071 (w), 1041 (vw), 1018 (w), 967 (vw), 931 (w), 875 (m), 848 (vw), 815 (w), 766 (s), 712 (s), 699 (vs), 679 (m).

HRMS (EI) calculated for $C_{18}H_{20}O_2^{·+}$: 268.1458, found 268.1460 [M]$^{·+}$.

mp: 82.2 – 83.9 °C.

3-(2-Oxocyclohexyl)bicyclo[1.1.1]pentane-1-carbonitrile (14e)

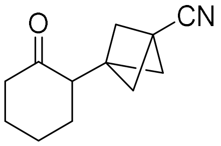

3-(2-Oxocyclohexyl)bicyclo[1.1.1]pentane-1-carbonitrile was prepared according to **TP2** using cyclopentanone (29 mg, 0.30 mmol, 1.5 equiv). The reaction was stirred at 0 °C for 30 min before adding CuCN·2LiCl in THF (1.0 M, 070 mL, 0.70 mmol, 3.5 equiv) and tosyl cyanide (145 mg, 0.80 mmol, 4.0 equiv). The resulting mixture was stirred at 25 °C for 18 h. Workup according to **TP2** and purification via column chromatography (*i*Hex / EtOAc = 85 / 15) afforded the desired compound **14e** (17 mg, 0.09 mmol, 46%) as a colorless solid.

^{1}H-NMR (CDCl$_3$, 400 MHz, ppm): δ = 2.37 (dd, J = 11.9, 5.5 Hz, 1 H), 2.23 (s, 8 H), 2.07 – 1.93 (m, 2 H), 1.91 – 1.78 (m, 1 H), 1.68 – 1.54 (m, 2 H), 1.44 – 1.31 (m, 1 H).

^{13}C-NMR (CDCl$_3$, 101 MHz, ppm): δ = 210.7, 117.9, 53.6, 49.6, 43.9, 42.4, 30.6, 27.4, 24.5, 24.4.

MS (70 eV, EI) *m/z* (%): 174 (10) [M–CH_3]$^{·+}$, 161 (10), 160 (100), 146 (21), 145 (11), 144 (100), 133 (13), 132 (24), 130 (69), 118 (31), 117 (36), 116 (20), 115 (10), 105 (15), 104 (40), 93 (13), 91 (46), 79 (38), 78 (11), 77 (27), 67 (17).

IR (ATR) $\tilde{\nu}$ (cm^{-1}): 2936 (m), 2861 (w), 2228 (w), 1706 (vs), 1446 (w), 1430 (w), 1377 (w), 1338 (vw), 1303 (w), 1272 (vw), 1256 (vw), 1225 (s), 1211 (w), 1145 (w), 1124 (m), 1105 (w),

1081 (w), 1068 (w), 1041 (vw), 1009 (vw), 963 (vw), 931 (w), 914 (vw), 892 (w), 847 (w), 793 (w), 771 (w), 705 (vw), 660 (w).

HRMS (EI) calculated for $C_{12}H_{14}NO^{\cdot+}$: 188.1070, found 188.1069 $[M–H]^{\cdot+}$.

mp: 45.8 – 47.6 °C.

1-(3-Allylbicyclo[1.1.1]pentan-1-yl)-3-cyclohexylpropan-2-one (14f)

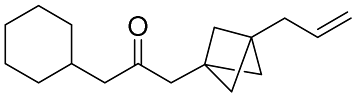

1-(3-Allylbicyclo[1.1.1]pentan-1-yl)-3-cyclohexylpropan-2-one was prepared according to **TP2** using cyclohexyl acetone (42 mg, 0.30 mmol, 1.5 equiv). The reaction was stirred at 0 °C for 2 h before adding CuCN·2LiCl in THF (1.0 M, 0.04 mL, 0.04 mmol, 20 mol%) and allyl bromide (85 mg, 0.70 mmol, 3.5 equiv). The resulting mixture was stirred at 25 °C for 2 h. Workup according to **TP2** and purification via column chromatography (*i*Hex / EtOAc = 99 / 1) afforded the desired compound **14f** (35 mg, 0.14 mmol, 71%) as a colorless liquid.

^{1}H-NMR ($CDCl_3$, 400 MHz, ppm): δ = 5.76 – 5.62 (m, 1 H), 5.02 – 4.93 (m, 2 H), 2.53 (s, 2 H), 2.24 (d, J = 6.8 Hz, 2 H), 2.19 (d, J = 7.2 Hz, 2 H), 1.86 – 1.75 (m, 1 H), 1.71 – 1.61 (m, 5 H), 1.59 (s, 6 H), 1.29 – 1.21 (m, 2 H), 1.18 – 1.09 (m, 1 H), 0.96 – 0.83 (m, 2 H).

^{13}C-NMR ($CDCl_3$, 101 MHz, ppm): δ = 209.5, 135.6, 115.9, 51.4, 51.4, 46.3, 39.7, 36.8, 36.5, 33.7, 33.4, 26.4, 26.3.

MS (70 eV, EI) *m/z* (%): 205 (0.5) $[M–C_3H_5]^{\cdot+}$, 125 (20), 98 (7), 97 (100), 91 (7), 79 (7), 69 (12), 55 (29).

IR (ATR) $\widetilde{\nu}$ (cm^{-1}): 3076 (vw), 2921 (vs), 2852 (s), 1711 (vs), 1640 (w), 1611 (vw), 1447 (m), 1404 (w), 1354 (w), 1293 (w), 1255 (vs), 1206 (w), 1148 (w), 1047 (m), 991 (m), 965 (w), 909 (vs), 803 (w), 708 (w).

HRMS (EI) calculated for $C_{16}H_{23}O^{\cdot+}$: 231.1743, found 231.1740 $[M–CH_3]^{\cdot+}$.

1-(Bicyclo[1.1.1]pentan-1-yl)-4-(2,6,6-trimethylcyclohex-1-en-1-yl)butan-2-one (14g)

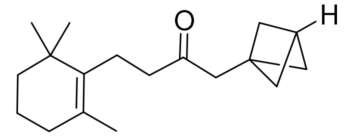

1-(Bicyclo[1.1.1]pentan-1-yl)-4-(2,6,6-trimethylcyclohex-1-en-1-yl)butan-2-one was prepared according to **TP2** using dihydro-β-ionone (58 mg, 0.30 mmol, 1.5 equiv). The reaction was stirred at 0 °C for 2 h before adding a saturated aqueous solution of NH_4Cl (1 mL). The resulting mixture was stirred at 25 °C for 10 min. Workup according to **TP2** and purification via column chromatography (*i*Hex / EtOAc = 99 / 1) afforded the desired compound **14g** (35 mg, 0.14 mmol, 67%) as a colorless liquid.

^{1}H-NMR ($CDCl_3$, 400 MHz, ppm): δ = 2.54 (s, 2 H), 2.50 – 2.41 (m, 3 H), 2.27 – 2.19 (m, 2 H), 1.89 (t, J = 6.2 Hz, 2 H), 1.78 (s, 6 H), 1.60 – 1.51 (m, 5 H), 1.44 – 1.36 (m, 2 H), 0.97 (s, 6 H).

^{13}C-NMR ($CDCl_3$, 101 MHz, ppm): δ = 209.5, 136.1, 127.8, 51.6, 46.5, 44.4, 41.4, 39.8, 35.2, 32.9, 28.6, 28.5, 22.1, 19.9, 19.6.

MS (70 eV, EI) *m/z* (%): 260 (8) $[M]^{\cdot+}$, 245 (12), 242 (18), 227 (11), 179 (13), 163 (15), 161 (35), 145 (12), 137 (29), 136 (52), 135 (16), 123 (38), 122 (14), 121 (100), 109 (14), 104 (10), 95 (25), 67 (14).

IR (ATR) $\tilde{\nu}$ (cm^{-1}): 2962 (s), 2926 (s), 2906 (s), 2868 (s), 1711 (vs), 1472 (w), 1457 (w), 1407 (w), 1360 (m), 1279 (w), 1256 (m), 1195 (s), 1147 (w), 1116 (w), 1083 (w), 1066 (w), 1041 (w), 1020 (vw), 970 (vw), 953 (vw), 873 (vw).

HRMS (EI) calculated for $C_{18}H_{28}O^{\cdot+}$: 260.2135, found 260.2131 $[M]^{\cdot+}$.

2-(3-Allylbicyclo[1.1.1]pentan-1-yl)-2-methyl-1-phenylpropan-1-one (14h)

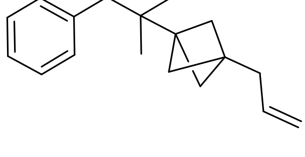

2-(3-Allylbicyclo[1.1.1]pentan-1-yl)-2-methyl-1-phenylpropan-1-one was prepared according to **TP2** using isobutyrophenone (44 mg, 0.30 mmol, 1.5 equiv). The reaction was stirred at 0 °C for 2 h before adding CuCN·2LiCl in THF (1.0 M, 0.04 mL, 0.04 mmol, 20 mol%) and allyl bromide (85 mg, 0.70 mmol, 3.5 equiv). The resulting mixture was stirred at 25 °C for 2 h.

Workup according to **TP2** and purification via column chromatography (*i*Hex / EtOAc = 49 / 1) afforded the desired compound **14h** (44 mg, 0.17 mmol, 86%) as a colorless liquid.

^{1}H-NMR ($CDCl_3$, 400 MHz, ppm): δ = 7.62 – 7.56 (m, 2 H), 7.45 – 7.41 (m, 1 H), 7.40 – 7.34 (m, 2 H), 5.68 (ddt, J = 16.4, 10.9, 7.2 Hz, 1 H), 5.02 – 4.92 (m, 2 H), 2.19 (dt, J = 7.3, 1.3 Hz, 2 H), 1.53 (s, 6 H), 1.27 (s, 6 H).

^{13}C-NMR ($CDCl_3$, 101 MHz, ppm): δ = 208.1, 140.3, 135.5, 130.7, 127.9, 127.7, 115.8, 48.2, 47.8, 45.6, 37.1, 36.6, 23.4.

MS (70 eV, EI) *m/z* (%): 253 (1) $[M–H]^{\cdot+}$, 239 (3), 148 (3), 147 (4), 119 (6), 107 (5), 106 (8), 105 (100), 93 (5), 91 (12), 79 (5), 77 (28).

IR (ATR) $\widetilde{\nu}$ (cm^{-1}): 3075 (vw), 2963 (m), 2906 (w), 1921 (vw), 1668 (s), 1641 (w), 1597 (vw), 1578 (vw), 1467 (w), 1445 (m), 1385 (w), 1362 (w), 1331 (vw), 1258 (s), 1209 (w), 1168 (m), 1126 (vw), 1077 (vw), 1018 (vw), 991 (w), 967 (s), 909 (s), 883 (w), 806 (w), 795 (w), 767 (vw), 739 (m), 698 (vs), 676 (w).

HRMS (EI) calculated for $C_{18}H_{21}O^{\cdot+}$: 253.1587, found 253.1590 $[M–H]^{\cdot+}$.

6-(3-Allylbicyclo[1.1.1]pentan-1-yl)cyclohex-2-en-1-one (14i)

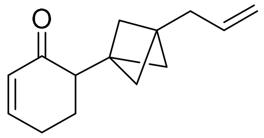

6-(3-Allylbicyclo[1.1.1]pentan-1-yl)cyclohex-2-en-1-one was prepared according to **TP2** using cyclohex-2-en-1-one (29 mg, 0.30 mmol, 1.5 equiv). The reaction was stirred at 0 °C for 30 min before adding CuCN·2LiCl in THF (1.0 M, 0.04 mL, 0.04 mmol, 20 mol%) and allyl bromide (85 mg, 0.70 mmol, 3.5 equiv). The resulting mixture was stirred at 0 °C for 30 min. Workup according to **TP2** and purification via column chromatography (*i*Hex / EtOAc = 19 / 1) afforded the desired compound **14i** (30 mg, 0.15 mmol, 75%) as a colorless liquid.

^{1}H-NMR ($CDCl_3$, 400 MHz, ppm): δ = 6.89 (dt, J = 10.1, 4.0 Hz, 1 H), 5.93 (dt, J = 10.1, 2.1 Hz, 1 H), 5.69 (ddt, J = 16.5, 10.5, 7.2 Hz, 1 H), 5.01 – 4.91 (m, 2 H), 2.48 – 2.35 (m, 2 H), 2.32 – 2.22 (m, 1 H), 2.19 (d, J = 7.2 Hz, 2 H), 2.05 (ddt, J = 13.0, 7.6, 5.3 Hz, 1 H), 1.87 (td, J = 13.3, 5.9 Hz, 1 H), 1.65 – 1.55 ($[AB]_3$-system, 6 H).

^{13}C-NMR ($CDCl_3$, 101 MHz, ppm): δ = 200.0, 149.6, 135.7, 130.0, 115.8, 50.3, 47.2, 40.3, 38.8, 36.8, 25.7, 24.3.

MS (70 eV, EI) *m/z* (%): 187 (8) $[M–CH_3]^{·+}$, 161 (57), 159 (16), 143 (15), 131 (14), 128 (15), 119 (37), 117 (25), 115 (13), 105 (33), 96 (13), 95 (17), 93 (24), 91 (100), 79 (22), 77 (23).

IR (ATR) $\tilde{\nu}$ (cm^{-1}): 3075 (vw), 2961 (w), 2904 (w), 1673 (vs), 1640 (w), 1620 (vw), 1448 (w), 1428 (w), 1411 (vw), 1386 (m), 1352 (vw), 1307 (w), 1286 (vw), 1252 (s), 1170 (vw), 1118 (w), 1019 (vw), 991 (w), 946 (w), 909 (m), 835 (w), 763 (w), 713 (w), 686 (m).

HRMS (EI) calculated for $C_{14}H_{17}O^{·+}$: 201.1274, found 201.1272 $[M–H]^{·+}$.

Ethyl 2-(3-allylbicyclo[1.1.1]pentan-1-yl)propanoate (15a)

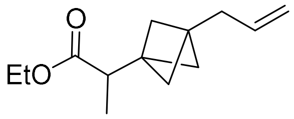

Ethyl 2-(3-allylbicyclo[1.1.1]pentan-1-yl)propanoate was prepared according to **TP3** using ethyl propionate (41 mg, 0.40 mmol, 2.0 equiv). After stirring at 0 °C for 3 h, CuCN·2LiCl in THF (1.0 M, 0.04 mL, 0.04 mmol, 20 mol%) and allyl bromide (61 mg, 0.50 mmol, 2.5 equiv) were added. Workup according to **TP3** and purification via column chromatography (*i*Hex / EtOAc = 49 / 1) afforded the desired compound **15a** (31 mg, 0.15 mmol, 75%) as a colorless liquid.

^{1}H-NMR (CDCl$_3$, 400 MHz, ppm): δ = 5.77 – 5.62 (m, 1 H), 5.02 – 4.93 (m, 2 H), 4.19 – 4.03 (m, 2 H), 2.54 (q, J = 7.0 Hz, 1 H), 2.20 (dt, J = 7.2, 1.3 Hz, 2 H), 1.51 (s, 6 H), 1.24 (t, J = 7.1 Hz, 3 H), 1.05 (d, J = 7.0 Hz, 3 H).

^{13}C-NMR (CDCl$_3$, 101 MHz, ppm): δ = 174.6, 135.7, 115.9, 60.2, 49.1, 41.15, 41.1, 37.9, 36.8, 14.6, 13.5.

MS (70 eV, EI) *m/z* (%): 167 (8) $[M–C_3H_5]^{·+}$, 139 (73), 135 (17), 133 (11), 119 (50), 107 (77), 106 (10), 105 (53), 93 (52), 91 (100), 79 (60), 77 (26), 44 (18).

IR (ATR) $\tilde{\nu}$ (cm^{-1}): 2962 (m), 2907 (m), 2869 (w), 1733 (vs), 1641 (vw), 1456 (w), 1409 (vw), 1374 (w), 1336 (w),1289 (w), 1255 (vs), 1227 (w), 1183 (vs), 1148 (s), 1096 (s), 1053 (m), 1019 (s), 1009 (s), 911 (w), 859 (w), 822 (w), 791 (s), 708 (w), 668 (w).

HRMS (EI) calculated for $C_{10}H_{15}O_2^{·+}$: 167.1067, found 167.1066 $[M–C_3H_5]^{·+}$.

Ethyl 2-(3-allylbicyclo[1.1.1]pentan-1-yl)hept-6-enoate (15b)

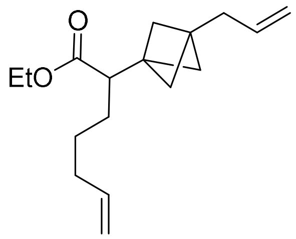

Ethyl 2-(3-allylbicyclo[1.1.1]pentan-1-yl)hept-6-enoate was prepared according to **TP3** using ethyl hept-6-enoate (156 mg, 1.0 mmol, 2.0 equiv) and [1.1.1]propellane in diethyl ether (0.50 mmol, 1.0 equiv). After stirring at 0 °C for 3 h, CuCN·2LiCl in THF (1.0 M, 0.10 mL, 0.10 mmol, 20 mol%) and allyl bromide (151 mg, 1.3 mmol, 2.5 equiv) were added. Workup according to **TP3** and purification via column chromatography (*i*Hex / EtOAc = 99 / 1) and HPLC afforded the desired compound **15b** (125 mg, 0.48 mmol, 95%) as a colorless liquid.

^{1}H-NMR ($CDCl_3$, 400 MHz, ppm): δ = 5.85 – 5.62 (m, 2 H), 5.03 – 4.90 (m, 4 H), 4.12 (qd, J = 7.1, 3.8 Hz, 2 H), 2.47 – 2.39 (m, 1 H), 2.20 (dt, J = 7.2, 1.3 Hz, 2 H), 2.08 – 1.99 (m, 2 H), 1.63 – 1.58 (m, 1 H), 1.53 (A part of an $[AB]_3$-system, 3 H), 1.49 (B part of an $[AB]_3$-system, 3 H), 1.42 – 1.29 (m, 3 H), 1.25 (t, J = 7.1 Hz, 3 H).

^{13}C-NMR ($CDCl_3$, 101 MHz, ppm): δ = 174.0, 138.7, 135.7, 115.9, 114.7, 60.1, 49.5, 47.4, 40.6, 38.1, 36.8, 33.8, 28.5, 27.2, 14.6.

MS (70 eV, EI) *m/z* (%): 221 (2) $[M–C_3H_5]^{·+}$, 193 (10), 165 (10), 147 (57), 145 (18), 137 (61), 133 (24), 131 (32), 125 (16), 121 (10), 119 (61), 117 (23), 107 (61), 93 (34), 91 (100), 81 (15), 79 (67), 77 (23), 67 (13).

IR (ATR) $\tilde{\nu}$ (cm^{-1}): 3077 (vw), 2963 (w), 2905 (w), 2868 (w), 1732 (vs), 1641 (w), 1444 (vw), 1370 (w), 1344 (w), 1254 (s), 1174 (m), 1124 (m), 1027 (w), 991 (m), 909 (vs), 805 (vw).

HRMS (EI) calculated for $C_{14}H_{21}O_2^{·+}$: 221.1536, found 221.1535 $[M–C_3H_5]^{·+}$.

Ethyl 2-(3-allylbicyclo[1.1.1]pentan-1-yl)-2-(4-bromophenyl)acetate (15c)

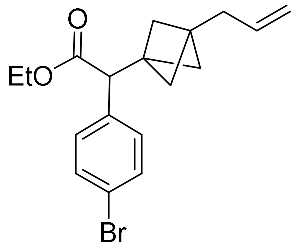

Ethyl 2-(3-allylbicyclo[1.1.1]pentan-1-yl)-2-(4-bromophenyl)acetate was prepared according to **TP3** using ethyl 2-(4-bromophenyl)acetate (97 mg, 1.0 mmol, 2.0 equiv). After stirring at 0 °C for 3 h, CuCN·2LiCl in THF (1.0 M, 0.10 mL, 0.10 mmol, 20 mol%) and allyl bromide (151 mg, 1.3 mmol, 2.5 equiv) were added. Workup according to **TP3** and purification via column chromatography (*i*Hex / EtOAc = 49 / 1) afforded the desired compound **15c** (66 mg, 0.19 mmol, 94%) as a colorless liquid.

^{1}H-NMR ($CDCl_3$, 400 MHz, ppm): δ = 7.42 (d, J = 8.4 Hz, 2 H), 7.17 (d, J = 8.5 Hz, 2 H), 5.73 – 5.57 (m, 1 H), 5.00 – 4.90 (m, 2 H), 4.22 – 4.05 (m, 2 H), 3.68 (s, 1 H), 2.18 (dt, J = 7.2, 1.3 Hz, 2 H), 1.52 (s, 6 H), 1.24 (t, J = 7.1 Hz, 3 H).

^{13}C-NMR ($CDCl_3$, 101 MHz, ppm): δ = 171.7, 136.0, 135.4, 131.4, 130.4, 121.2, 116.0, 60.8, 53.0, 49.7, 41.0, 39.3, 36.7, 14.4.

MS (70 eV, EI) *m/z* (%): 307 (6) $[M–C_3H_5]^{·+}$, 281 (41), 279 (41), 215 (13), 214 (13), 196 (58), 195 (20), 182 (14), 181 (100), 180 (19), 179 (27), 172 (13), 171 (43), 169 (43), 168 (25), 167 (47), 166 (32), 165 (45), 155 (32), 154 (63), 153 (72), 152 (28), 151 (78), 141 (25), 135 (15), 134 (16), 129 (21), 128 (47), 116 (16), 115 (50), 107 (29), 105 (22), 91 (34), 89 (20), 79 (33), 77 (18).

IR (ATR) $\widetilde{\nu}$ (cm^{-1}): 3075 (vw), 2965 (w), 2905 (w), 2868 (w), 1732 (vs), 1640 (vw), 1488 (s), 1464 (vw), 1445 (w), 1408 (w), 1367 (w), 1336 (w), 1302 (w), 1248 (s), 1197 (s), 1156 (vs), 1121 (s), 1073 (s), 1011 (vs), 991 (m), 911 (m), 880 (vw), 835 (m), 757 (w), 737 (w), 712 (w), 683 (vw).

HRMS (EI) calculated for $C_{18}H_{21}BrO_2^{·+}$: 348.0719, found 348.0700 $[M]^{·+}$.

1-(3-Allylbicyclo[1.1.1]pentan-1-yl)cyclohexane-1-carbonitrile (22a)

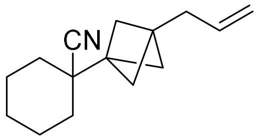

1-(3-Allylbicyclo[1.1.1]pentan-1-yl)cyclohexane-1-carbonitrile was prepared according **TP4** using cyclohexanecarbonitrile (44 mg, 0.40 mmol, 2.0 equiv). The reaction mixture was stirred 6 h at 25 °C. Trapping and workup according to **TP4** and purification via column chromatography (*i*Hex / EtOAc = 49 / 1) afforded the desired compound **22a** (22 mg, 0.10 mmol, 51%) as a colorless solid.

^{1}H-NMR ($CDCl_3$, 400 MHz, ppm): δ = 5.70 (ddt, J = 16.4, 11.0, 7.2 Hz, 1 H), 5.05 – 4.93 (m, 2 H), 2.25 (dt, J = 7.2, 1.3 Hz, 2 H), 1.87 – 1.69 (m, 5 H), 1.56 (s, 8 H), 1.21 – 1.06 (m, 3 H).

^{13}C-NMR ($CDCl_3$, 101 MHz, ppm): δ = 135.2, 122.4, 116.3, 47.1, 43.8, 39.7, 37.3, 36.5, 32.0, 25.3, 23.1.

MS (70 eV, EI) *m/z* (%): 214 (6) $[M–H]^{·+}$, 186 (13), 172 (18), 160 (19), 158 (13), 146 (29), 144 (21), 132 (14), 131 (11), 130 (11), 118 (11), 117 (13), 107 (40), 105 (33), 93 (12), 92 (14), 91 (67), 81 (14), 79 (100), 77 (16), 67 (15).

IR (ATR) $\tilde{\nu}$ (cm^{-1}): 3076 (vw), 2963 (m), 2933 (s), 2907 (m), 2861 (s), 2227 (vw), 1641 (w), 1447 (m), 1286 (vw), 1265 (s), 1249 (vs), 1213 (vw), 1179 (w), 1136 (w), 991 (s), 937 (vw), 910 (vs), 870 (w), 811 (vw), 713 (w).

HRMS (EI) calculated for $C_{15}H_{20}N^{·+}$: 214.1590, found 214.1588 $[M–H]^{·+}$.

mp: 33.5 – 35.3 °C.

2-(3-Allylbicyclo[1.1.1]pentan-1-yl)-2-phenylpropanenitrile (22b)

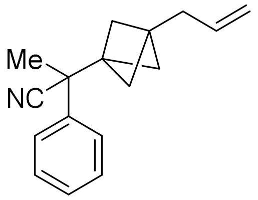

2-(3-Allylbicyclo[1.1.1]pentan-1-yl)-2-phenylpropanenitrile was prepared according **TP4** using α-methylbenzyl cyanide (52 mg, 0.40 mmol, 2.0 equiv). The reaction mixture was stirred 3 h at 25 °C. Trapping and workup according to **TP4** and purification via column chromatography

(*i*Hex / EtOAc = 49 / 1) afforded the desired compound **22b** (46 mg, 0.19 mmol, 96%) as a colorless liquid.

^{1}H-NMR (CDCl$_3$, 400 MHz, ppm): δ = 7.39 – 7.27 (m, 5 H), 5.72 – 5.58 (m, 1 H), 5.02 – 4.92 (m, 2 H), 2.21 (dt, J = 7.2, 1.3 Hz, 2 H), 1.70 (s, 3 H), 1.51 (s, 6 H).

^{13}C-NMR (CDCl$_3$, 101 MHz, ppm): δ = 137.7, 134.9, 128.6, 127.8, 126.0, 122.3, 116.4, 47.6, 45.1, 43.3, 37.7, 36.4, 21.9.

MS (70 eV, EI) *m/z* (%): 236 (5) [M–H]$^{\cdot+}$, 222 (6), 194 (6), 181 (10), 180 (11), 167 (9), 154 (12), 153 (10), 141 (12), 131 (51), 130 (34), 129 (22), 128 (21), 127 (11), 116 (14), 115 (21), 107 (51), 105 (26), 104 (16), 103 (53), 102 (10), 92 (11), 91 (79), 79 (100), 78 (18), 77 (63), 67 (15), 65 (22), 53 (14), 51 (22).

IR (ATR) $\widetilde{\nu}$ (cm^{-1}): 2970 (w), 2909 (vw), 2872 (w), 2235 (vw), 1641 (vw), 1601 (vw), 1493 (w), 1446 (w), 1378 (vw), 1325 (vw), 1247 (s), 1174 (w), 1134 (w), 1076 (w), 1026 (w), 992 (w), 913 (m), 801 (w), 744 (s), 697 (vs), 657 (m).

HRMS (EI) calculated for $C_{17}H_{18}N^{\cdot+}$: 236.1434, found 236.1433 [M–H]$^{\cdot+}$.

1-(3-Allylbicyclo[1.1.1]pentan-1-yl)cyclohex-2-ene-1-carbonitrile (22c)

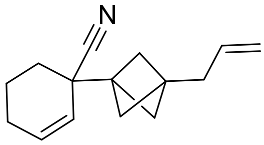

1-(3-Allylbicyclo[1.1.1]pentan-1-yl)cyclohex-2-ene-1-carbonitrile was prepared according **TP4** using 1-cyanocyclohexene (43 mg, 0.40 mmol, 2.0 equiv). The reaction mixture was stirred 2 h at 25 °C. Trapping and workup according to **TP4** and purification via column chromatography (*i*Hex / EtOAc = 49 / 1) afforded the desired compound **22c** (41 mg, 0.19 mmol, 96%) as a colorless liquid.

^{1}H-NMR (CDCl$_3$, 400 MHz, ppm): δ = 5.92 (ddd, J = 9.9, 4.6, 2.9 Hz, 1 H), 5.76 – 5.63 (m, 1 H), 5.52 – 5.46 (m, 1 H), 5.04 – 4.96 (m, 2 H), 2.25 (dt, J = 7.2, 1.3 Hz, 2 H), 2.13 – 2.03 (m, 1 H), 2.02 – 1.91 (m, 2 H), 1.82 – 1.73 (m, 2 H), 1.64 – 1.55 ([AB]$_3$-system, 6 H), 1.55 – 1.48 (m, 1 H).

^{13}C-NMR (CDCl$_3$, 101 MHz, ppm): δ = 134.9, 131.8, 123.4, 121.8, 116.3, 47.3, 42.9, 38.1, 38.0, 36.4, 29.7, 24.4, 19.2.

MS (70 eV, EI) *m/z* (%): 212 (4) $[M{-}H]^{\cdot+}$, 184 (9), 170 (16), 156 (23), 145 (16), 144 (15), 143 (15), 142 (11), 131 (12), 130 (30), 129 (29), 128 (16), 117 (24), 116 (15), 115 (22), 107 (21), 105 (32), 103 (12), 92 (10), 91 (81), 80 (10), 79 (100), 77 (36), 65 (11).

IR (ATR) $\widetilde{\nu}$ (cm^{-1}): 3028 (vw), 2966 (m), 2908 (m), 2870 (m), 2230 (vw), 1641 (w), 1446 (w), 1432 (w), 1257 (vs), 1230 (vw), 1194 (w), 1144 (vw), 1019 (vw), 992 (m), 912 (s), 884 (w), 845 (w), 730 (vs), 695 (s), 668 (w).

HRMS (EI) calculated for $C_{15}H_{18}N^{\cdot+}$: 212.1434, found 212.1433 $[M{-}H]^{\cdot+}$.

Ethyl 4-(bicyclo[1.1.1]pentan-1-yl)-1-methylpiperidine-4-carboxylate (6)

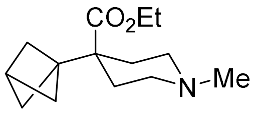

Diisopropylamine (319 mg, 3.15 mmol, 2.1 equiv) was dissolved in THF (3.5 mL) and BuLi (2.27 M in hexane, 1.39 mL, 3.15 mmol, 2.1 equiv) was added dropwise at 0 °C. The mixture was stirred for 5 min and cooled to −78 °C. Then ethyl 1-methylpiperidine-4-carboxylate (514 mg, 3.0 mmol, 2.0 equiv) was added dropwise. After 30 min a solution of $ZnCl_2$ in THF (1.0 M, 3.75 mL, 3.75 mmol, 2.5 equiv) was added and the mixture was stirred at 0 °C for 5 min before adding the [1.1.1]propellane in diethyl ether (0.53 M, 2.83 mL, 1.5 mmol, 1.0 equiv). After stirring at 0 °C for 2 h a saturated aqueous solution of NH_4Cl (5 mL) was added. The reaction mixture was stirred for another 10 min at 25 °C, extracted with EtOAc (3 times), washed with brine, dried over $MgSO_4$ and concentrated *in vacuo*. Purification via column chromatography (alumina, grade III, *i*Hex / NEt_3 = 19 / 1) afforded the desired compound **6** (338 mg, 1.42 mmol, 95%) as colorless crystals. The structure was confirmed via single crystal X-ray diffraction studies.

^{1}H-NMR ($CDCl_3$, 400 MHz, ppm): δ = 4.16 (q, J = 7.1 Hz, 2 H), 2.75 (d, J = 11.8 Hz, 2 H), 2.44 (s, 1 H), 2.22 (s, 3 H), 2.06 (d, J = 13.5 Hz, 2 H), 1.84 (t, J = 12.2 Hz, 2 H), 1.62 (s, 6 H), 1.45 (td, J = 13.2, 3.8 Hz, 2 H), 1.26 (t, J = 7.1 Hz, 3 H).

^{13}C-NMR ($CDCl_3$, 101 MHz, ppm): δ = 173.8, 60.3, 53.6, 49.3, 47.9, 46.4, 46.1, 30.1, 25.3, 14.7.

MS (70 eV, EI) *m/z* (%): 237 (28) $[M]^{\cdot+}$, 236 (21), 222 (11), 208 (42), 192 (19), 168 (26), 165 (12), 164 (100), 162 (42), 148 (13), 140 (20), 136 (24), 122 (14), 120 (11), 105 (10), 96 (22), 94 (27), 91 (19), 79 (13), 71 (14), 70 (35), 42 (15).

IR (ATR) $\widetilde{\nu}$ (cm^{-1}): 2964 (m), 2935 w), 2908 (w), 2872 (w), 2840 (vw), 2739 (vw), 1723 (vs), 1467 (w), 1441 (w), 1427 (vw), 1378 (w), 1365 (vw), 1315 (w), 1290 (m), 1229 (vw), 1208 (m), 1199 (s), 1183 (m), 1155 (s), 1142 (vs), 1094 (s), 1058 (w), 1024 (w), 1004 (w), 966 (w), 949 (vw), 891 (vw), 863 (vw), 852 (vw), 789 (vw), 762 (w), 701 (vw).

HRMS (EI) calculated for $C_{14}H_{23}NO_2^{\cdot+}$: 237.1723, found 237.1725 $[M]^{\cdot+}$.

mp: 27.4 – 29.5 °C.

The impact of the substitution of the terminal phenyl group with a BCP unit was evaluated by measuring some physicochemical properties of the BCP-pethidine **6**. We found the bioisoster to be slightly more basic than the original drug **24**, with the pK_a of the conjugated acid increasing from 8.8 to 9.0. The lipophilicity was estimated by measuring the 1-octanol/water partition coefficient (logP) and the distribution coefficient at pH 7.4 (log$D_{7.4}$) and comparing them to the literature values of pethidine (**24**).[20] The measured logP (2.9) and log$D_{7.4}$ (1.3) were very close to the reported values for pethidine (respectively 2.7 and 1.4). This indicates that the lipophilicity of the bioisoster **6** is similar to that of pethidine. Contrastingly, the substitution of an internal *para*-phenyl group with a BCP unit resulted in a significant decrease of the lipophilicity for a number of different drug candidates.[21] Overall, in this case the substitution of the terminal phenyl group seems to have a relatively small impact on the physicochemical properties of the compound.

[20] a) L. Z. Benet, F. Broccatelli, T. I. Oprea, *AAPS J.* **2011**, *13*, 519-547; b) L. Xu, L. Li, J. Huang, S. Yu, J. Wang, N. Li, *J. Pharm. Biomed. Anal.* **2015**, *102*, 409-416.

[21] Y. P. Auberson, C. Brocklehurst, M. Furegati, T. C. Fessard, G. Koch, A. Decker, L. La Vecchia, E. Briard, *ChemMedChem* **2017**, *12*, 590-598.

NMR Spectra

(3-Allylbicyclo[1.1.1]pentan-1-yl)(phenyl)methanone (9a)

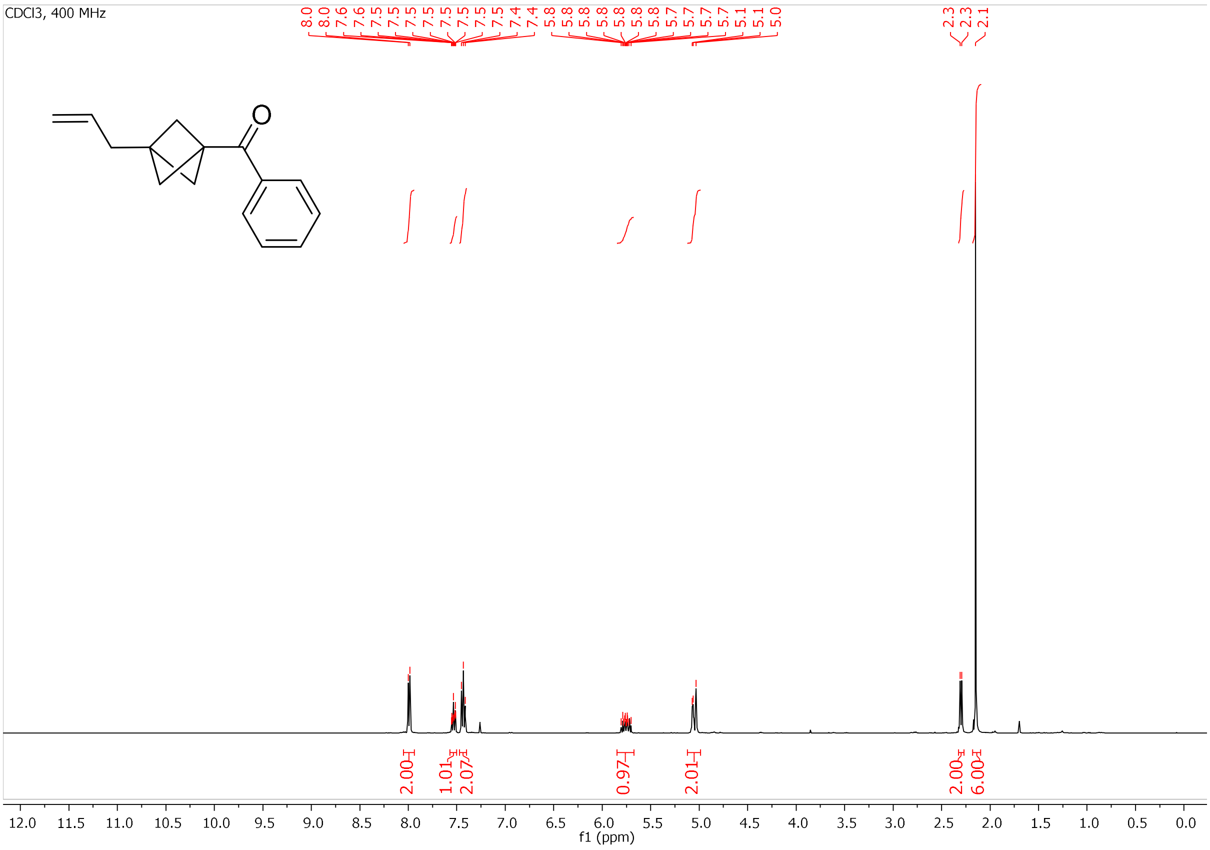

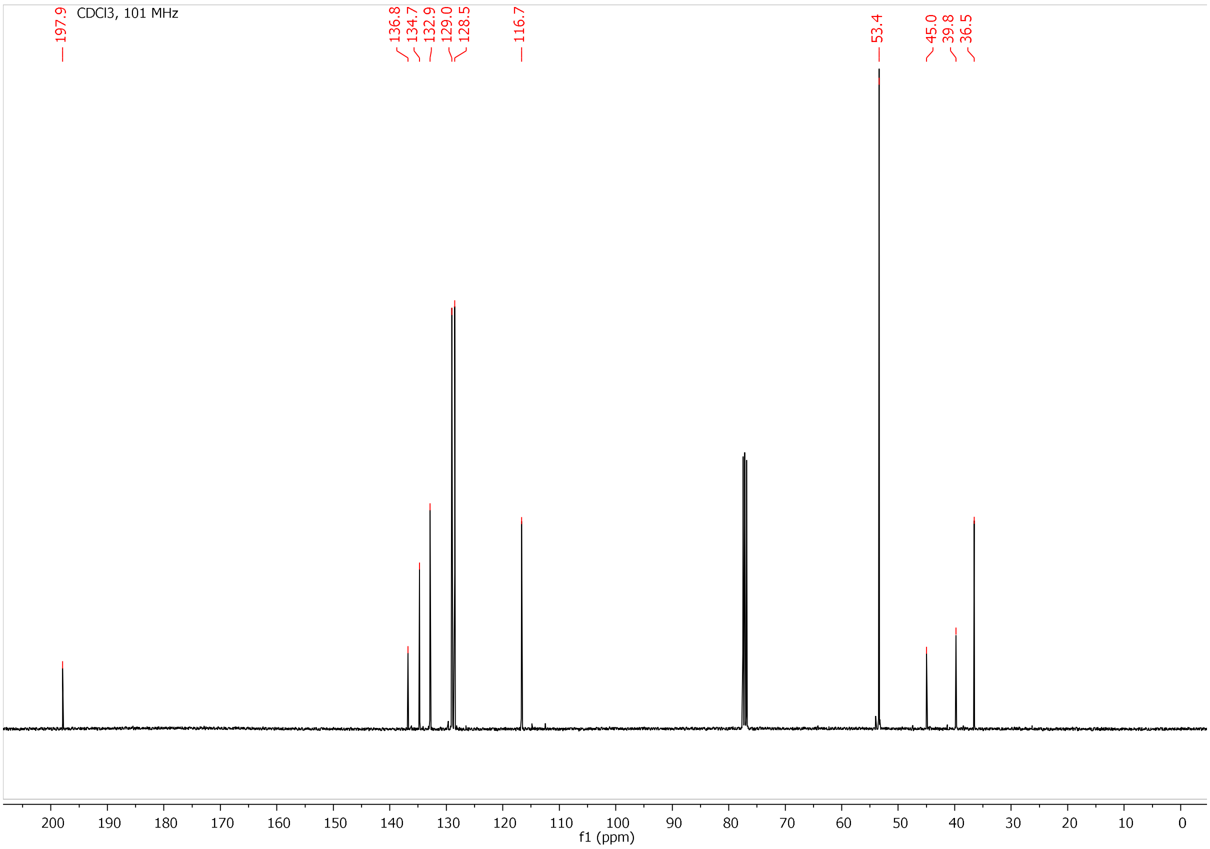

Phenyl(3-(1-phenylallyl)bicyclo[1.1.1]pentan-1-yl)methanone (9b)

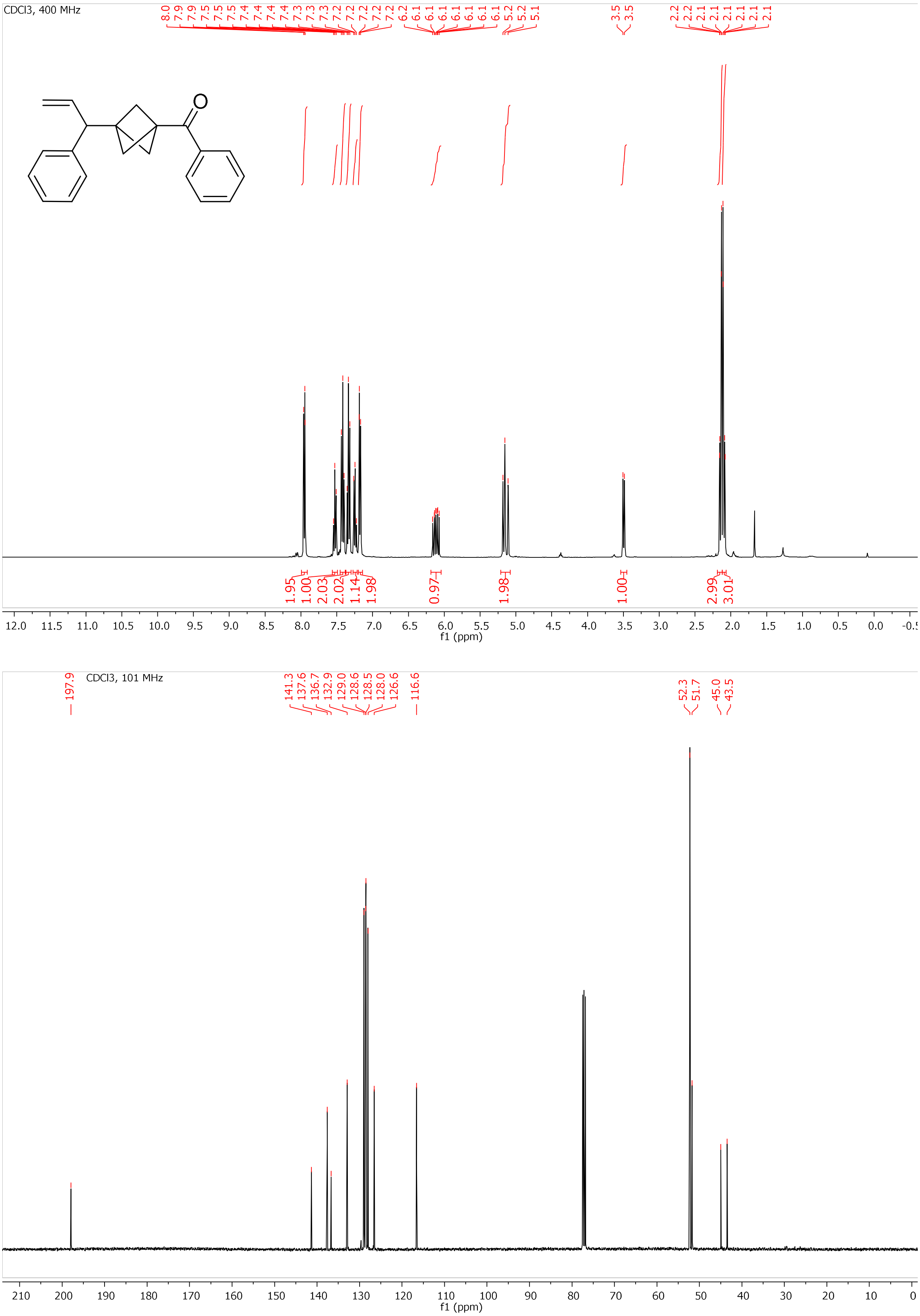

1-Allyl-3-(4-methoxyphenyl)bicyclo[1.1.1]pentane (9c)

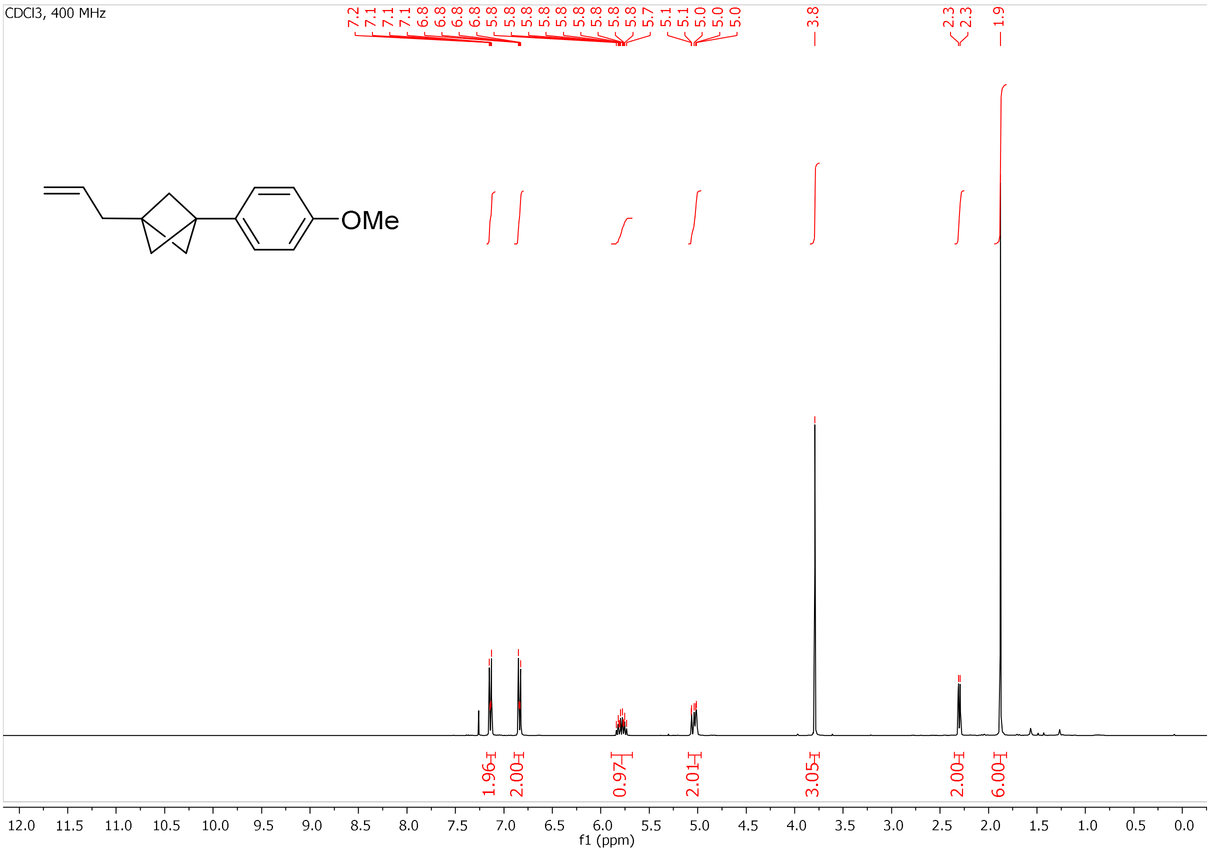

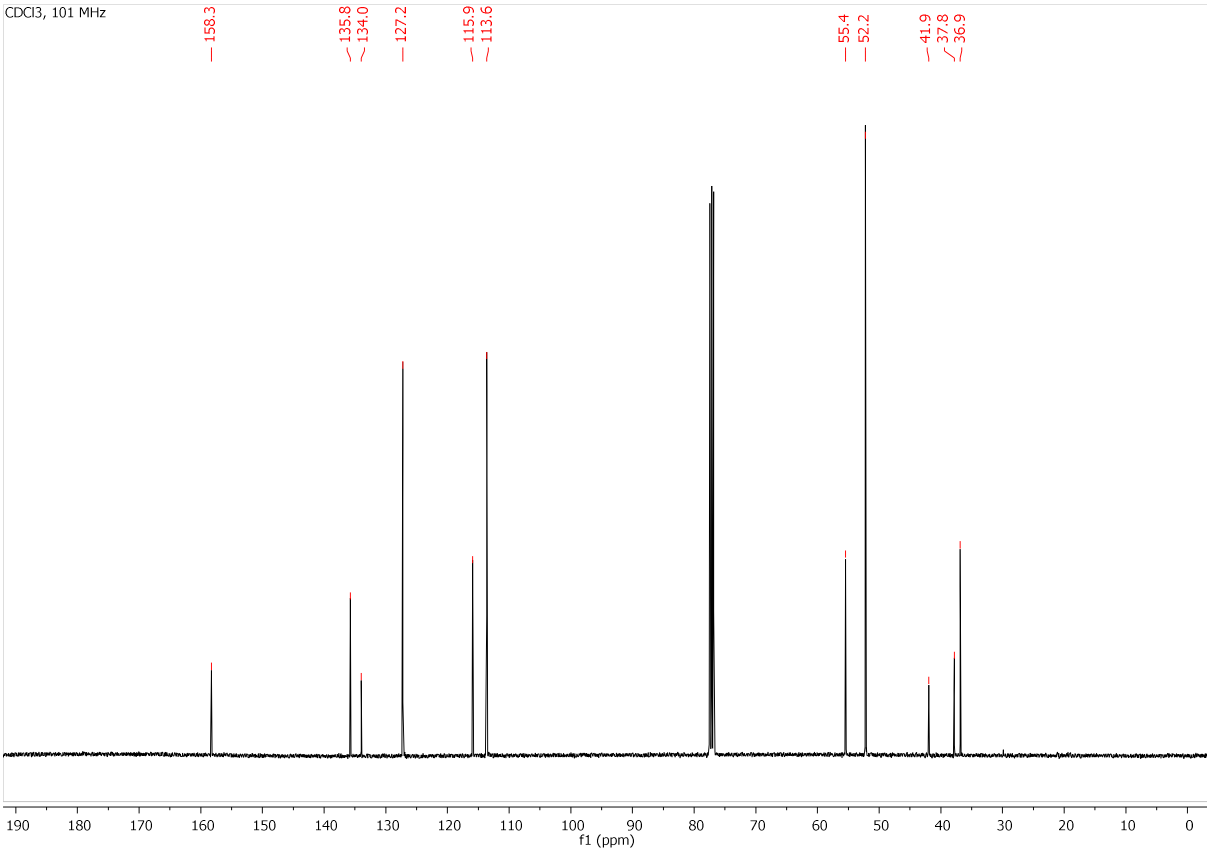

Ethyl 4-(3-allylbicyclo[1.1.1]pentan-1-yl)benzoate (9d)

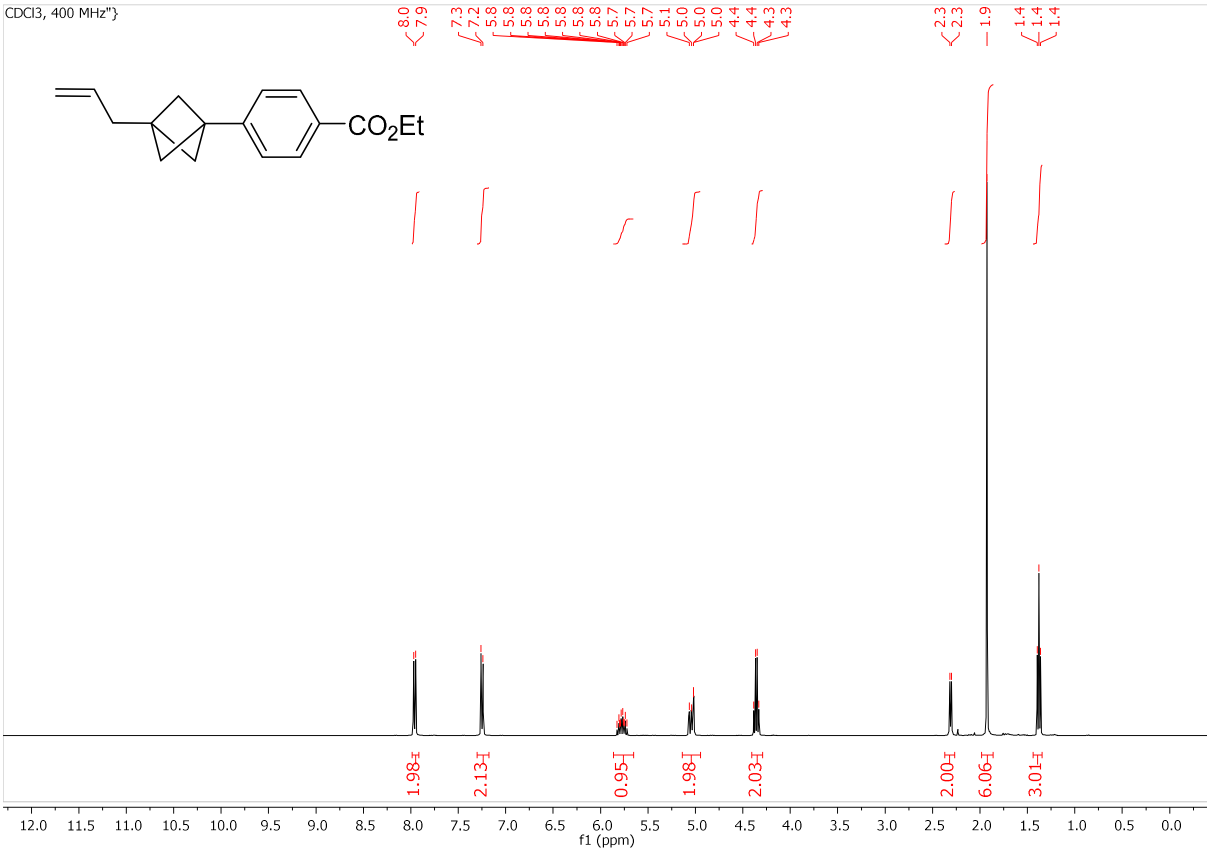

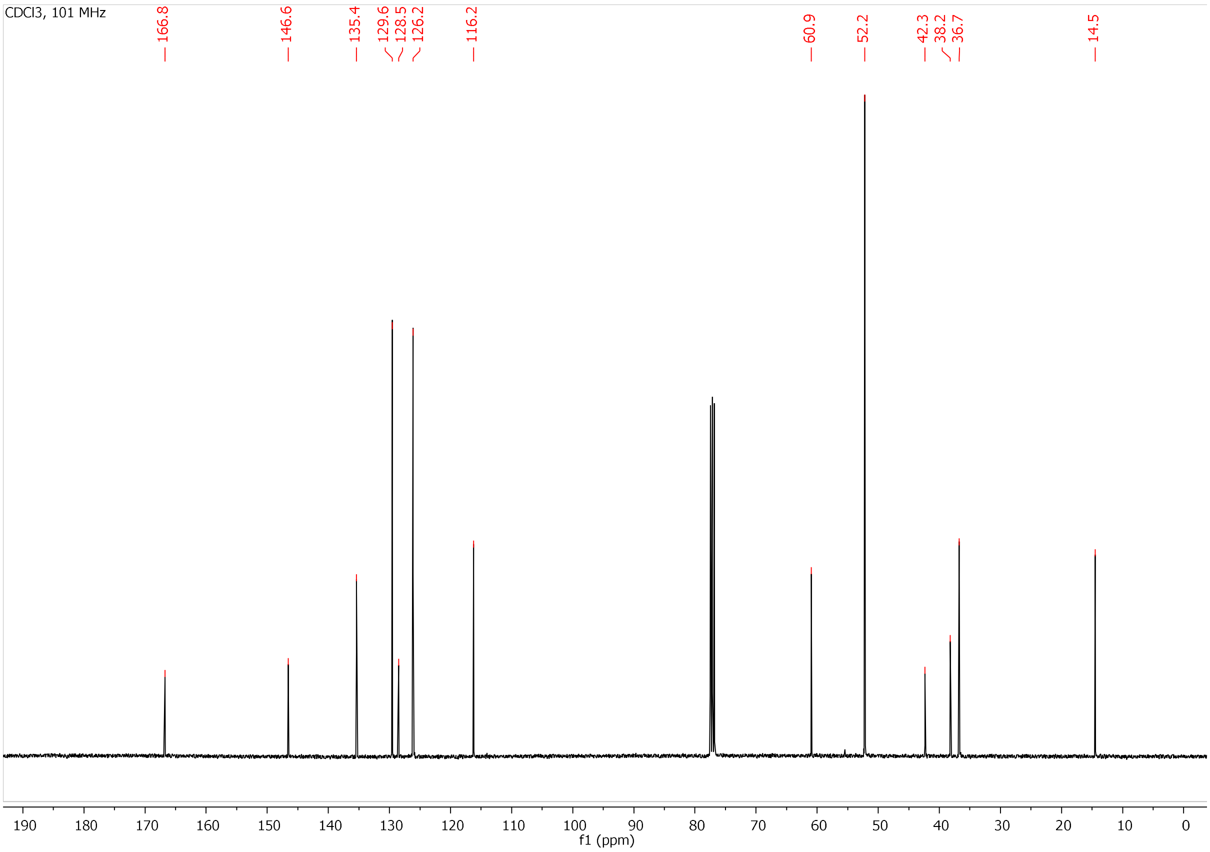

2-(3-Allylbicyclo[1.1.1]pentan-1-yl)pyridine (9e)

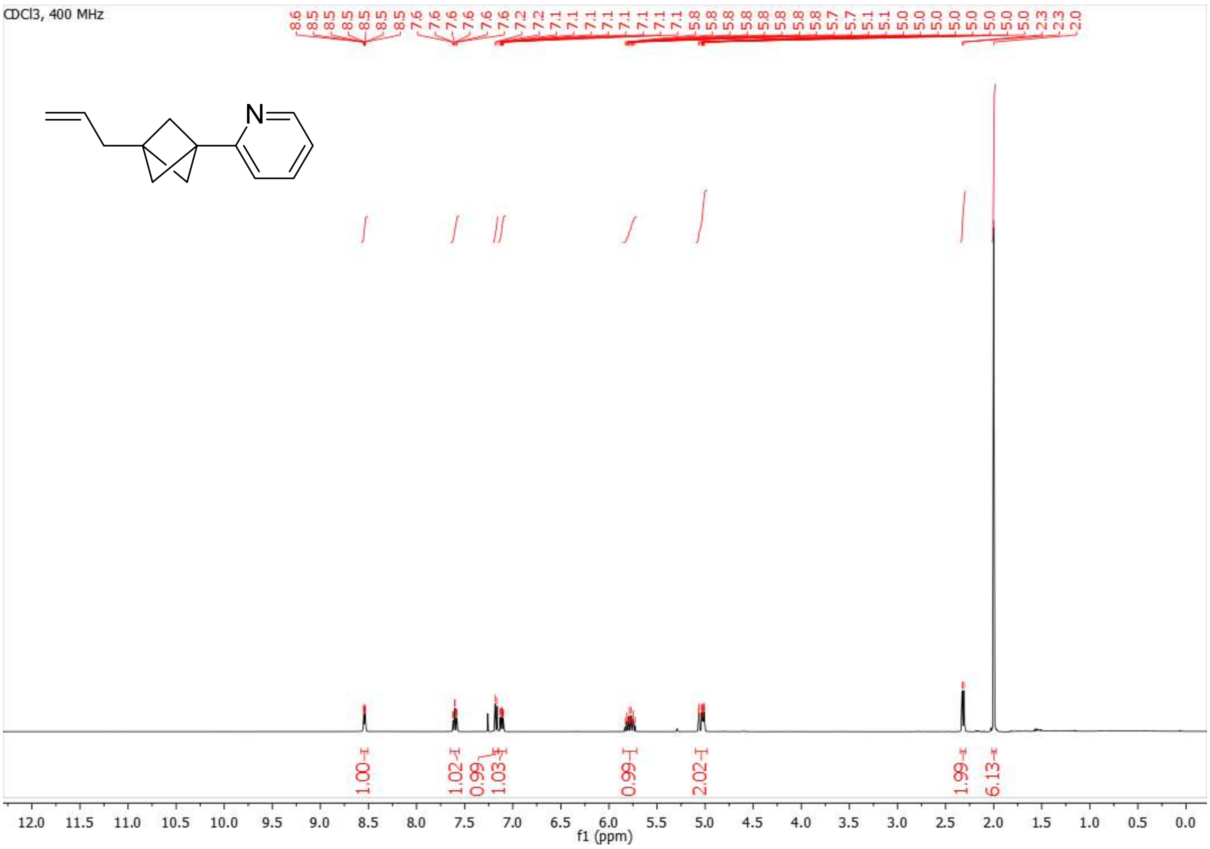

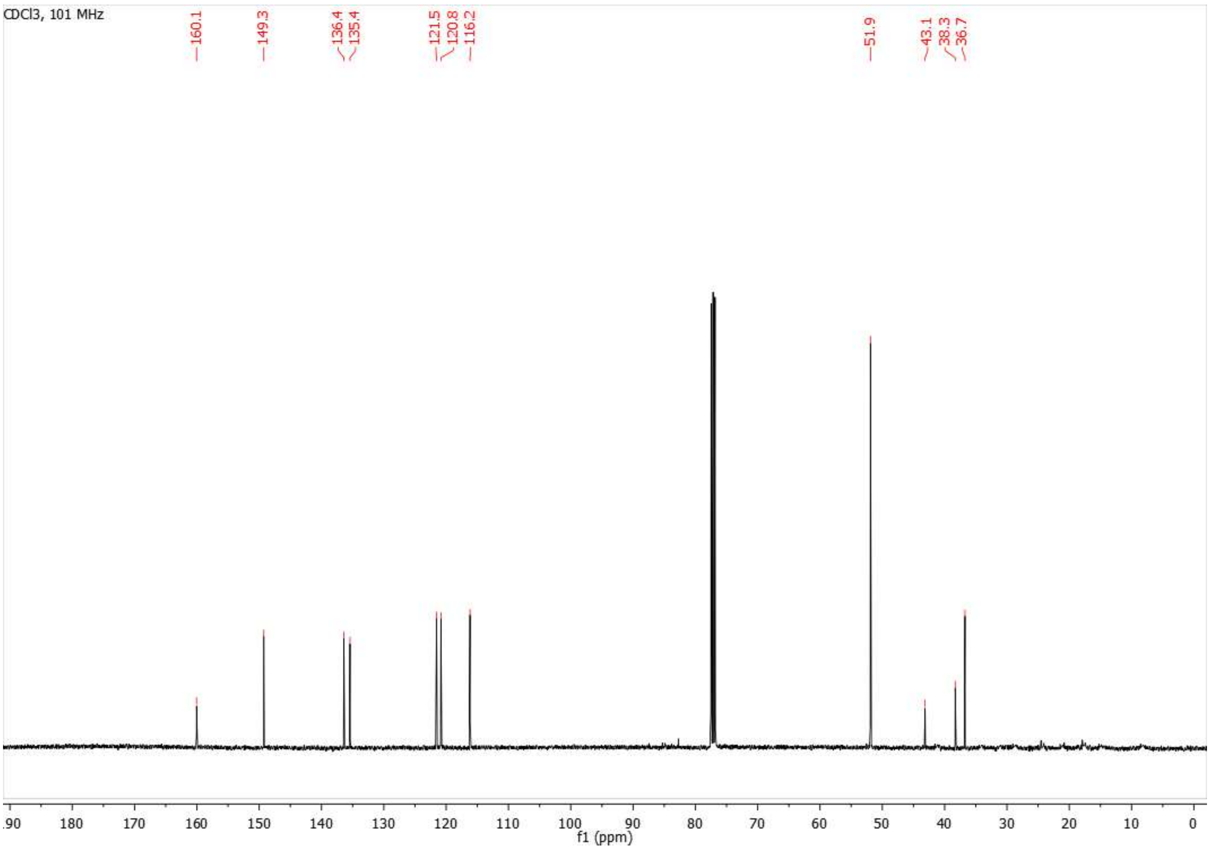

(3-Allylbicyclo[1.1.1]pentan-1-yl)(phenyl)sulfane (9f)

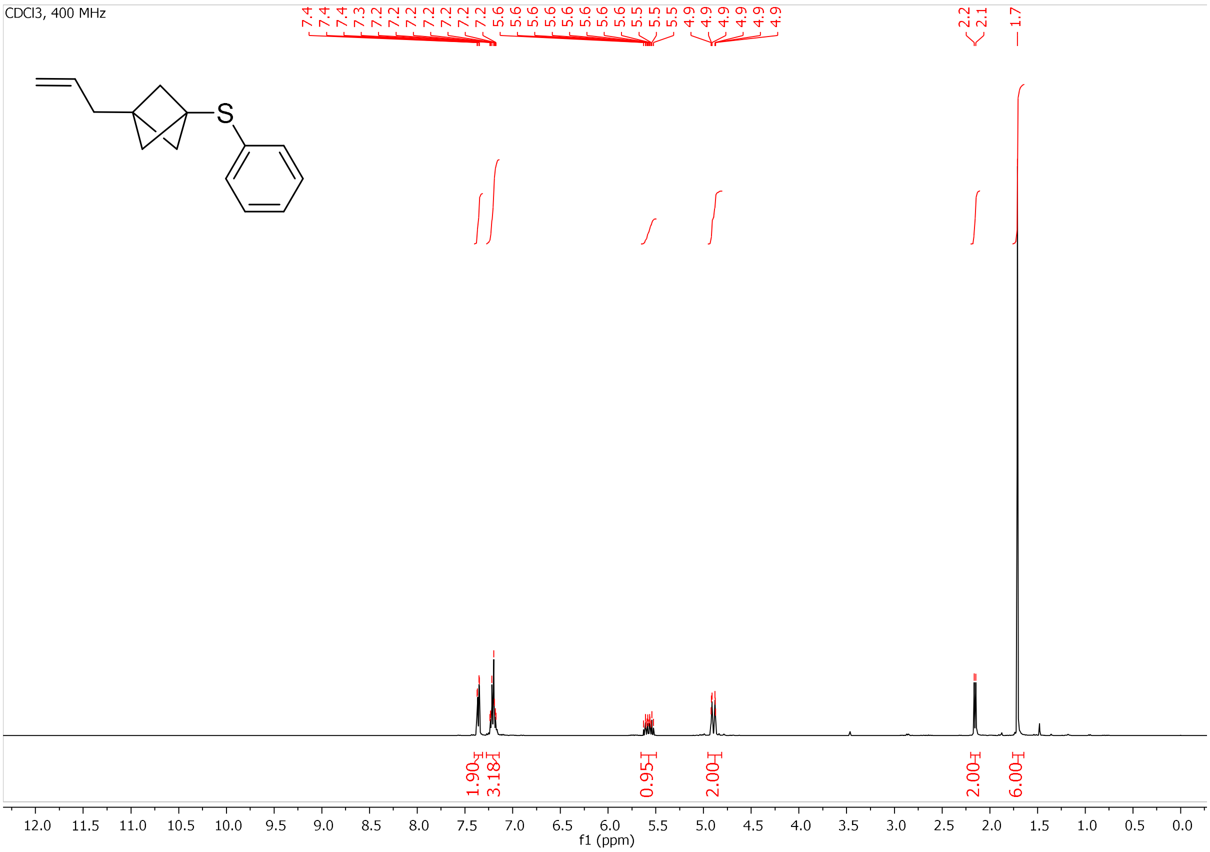

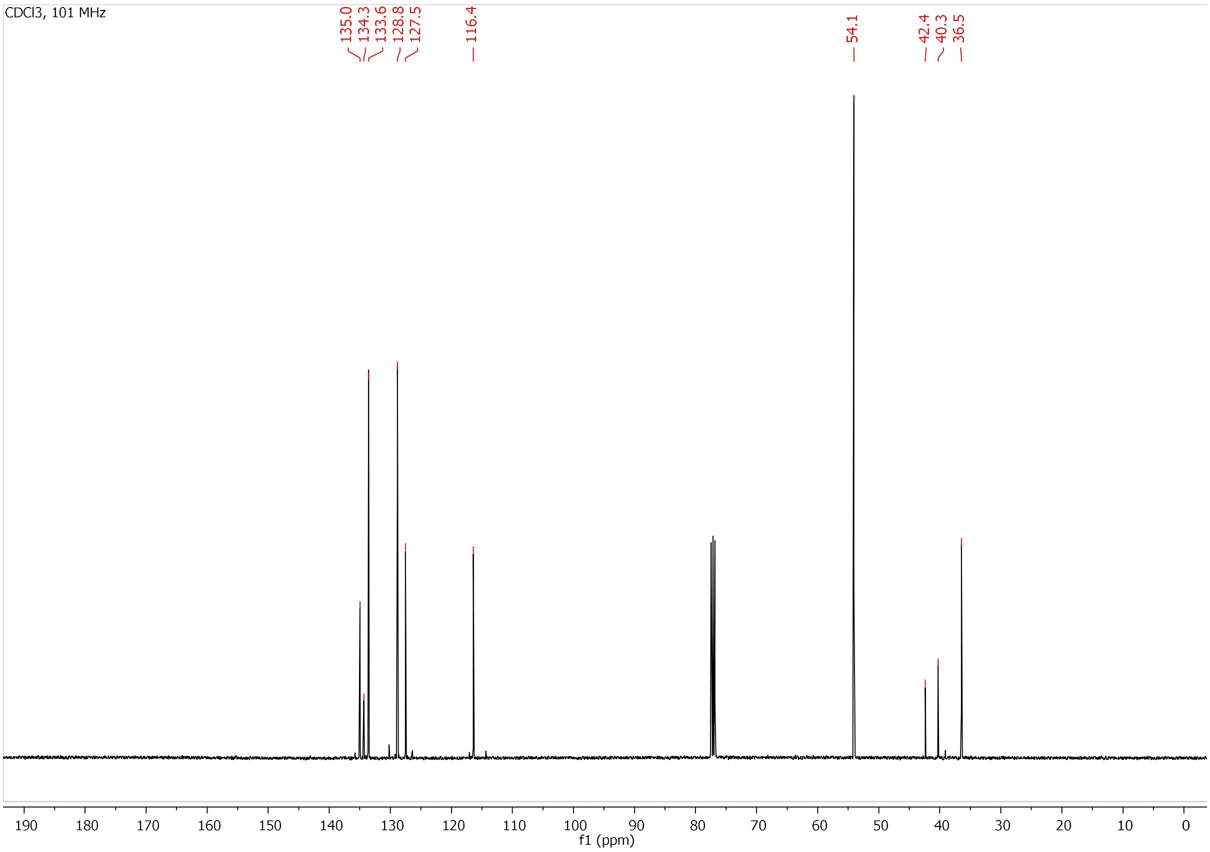

***N*,*N*,*3*-Triallylbicyclo[1.1.1]pentan-1-amine (9g)**

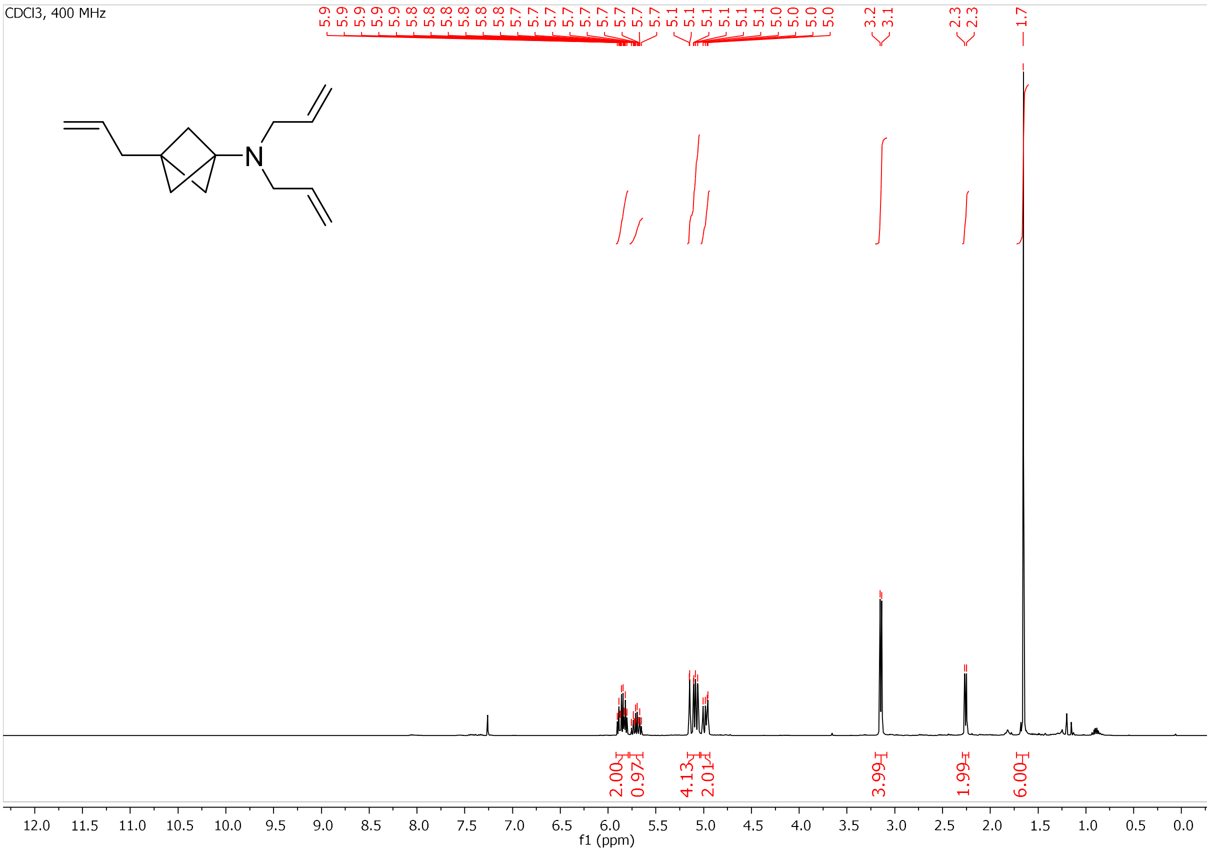

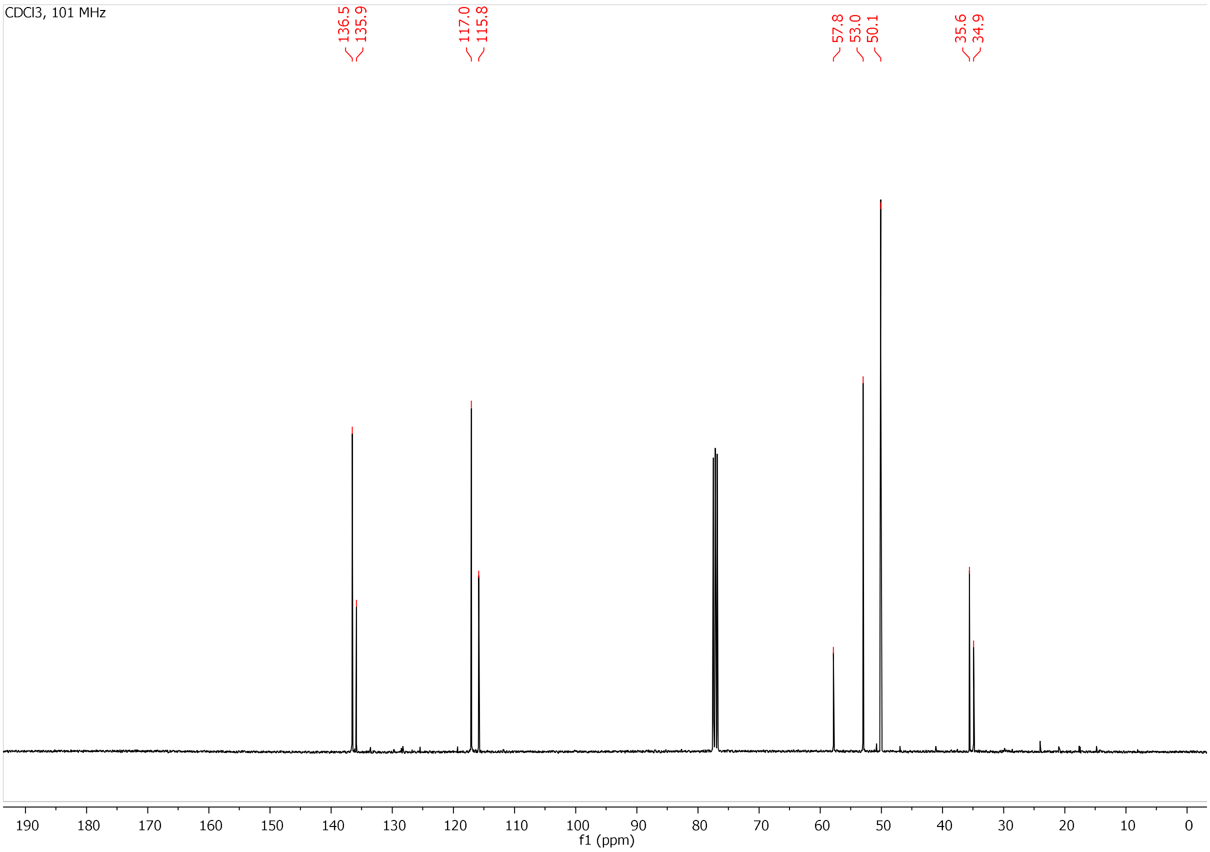

Ethyl 2-((3-(1-phenylallyl)bicyclo[1.1.1]pentan-1-yl)methyl)acrylate (9h)

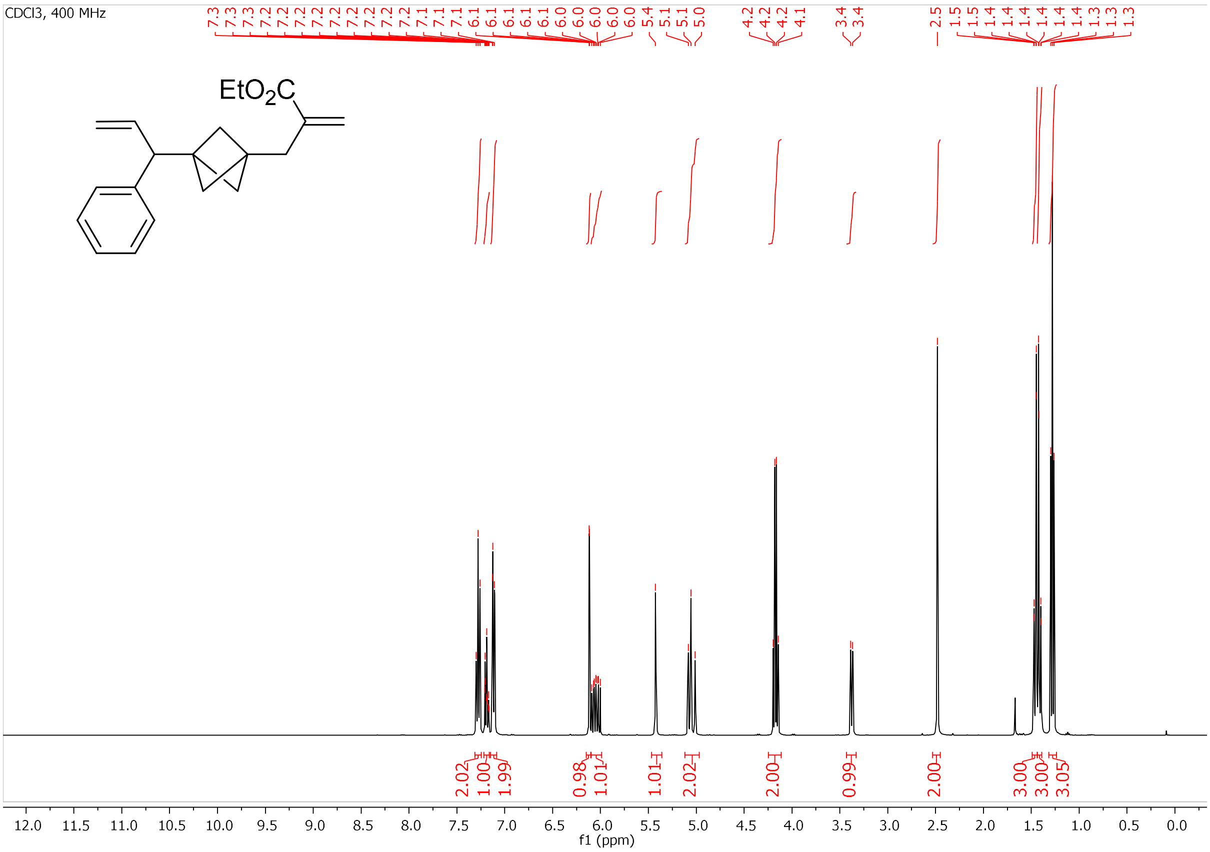

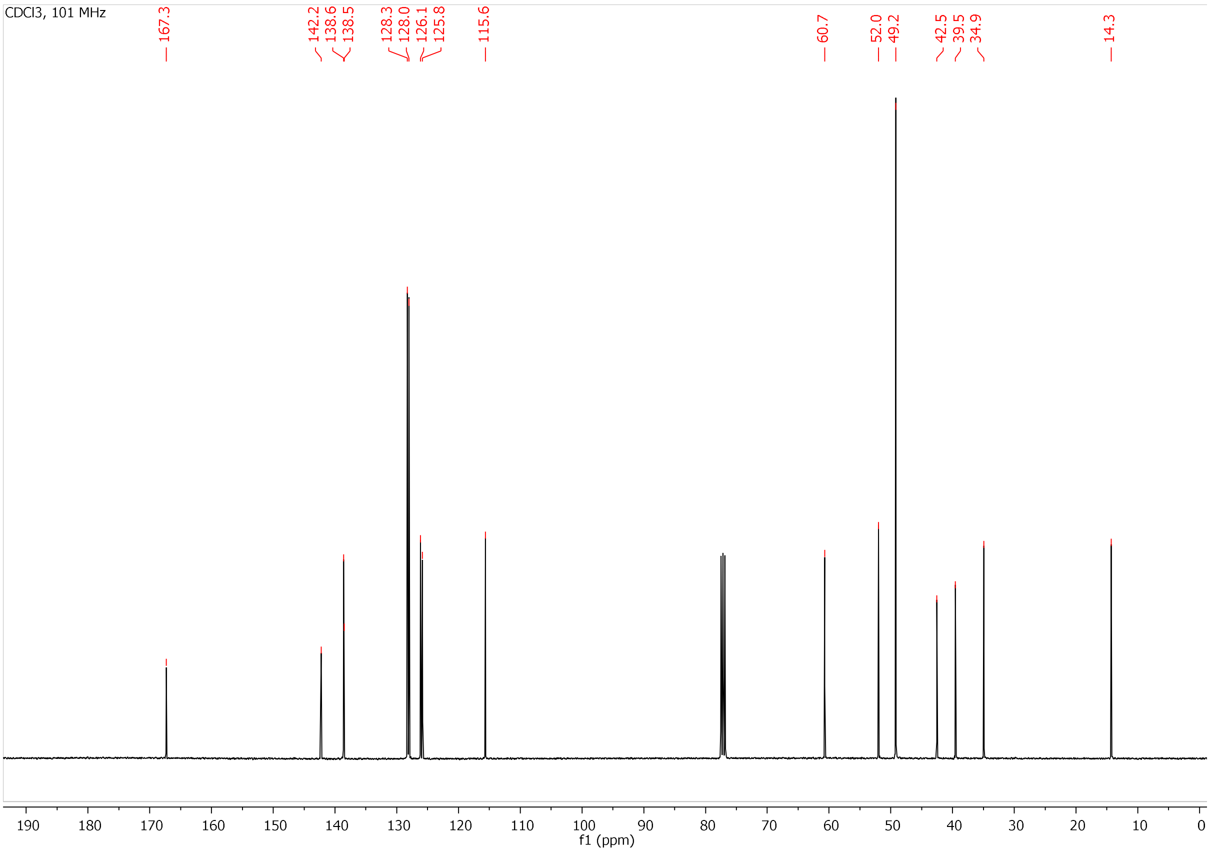

Methyl(3-(1-phenylallyl)bicyclo[1.1.1]pentan-1-yl)sulfane (9i)

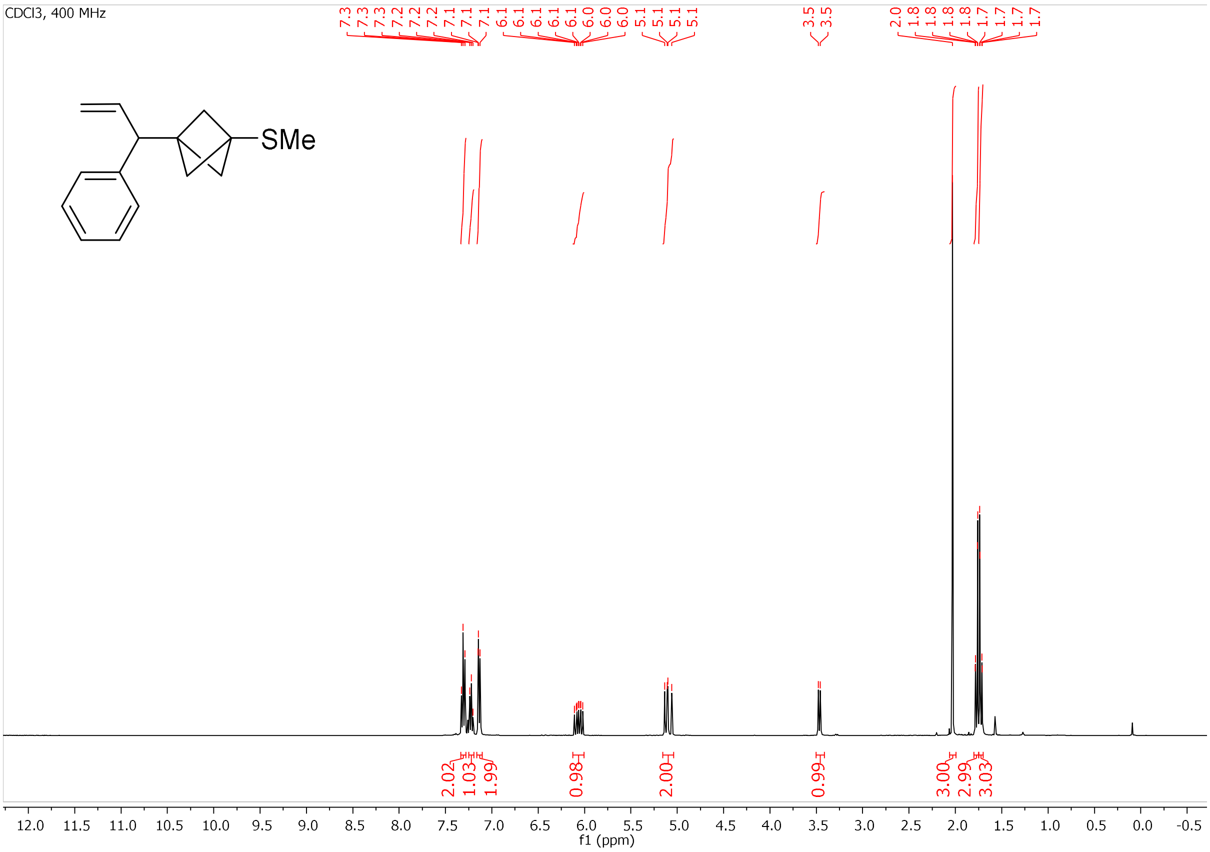

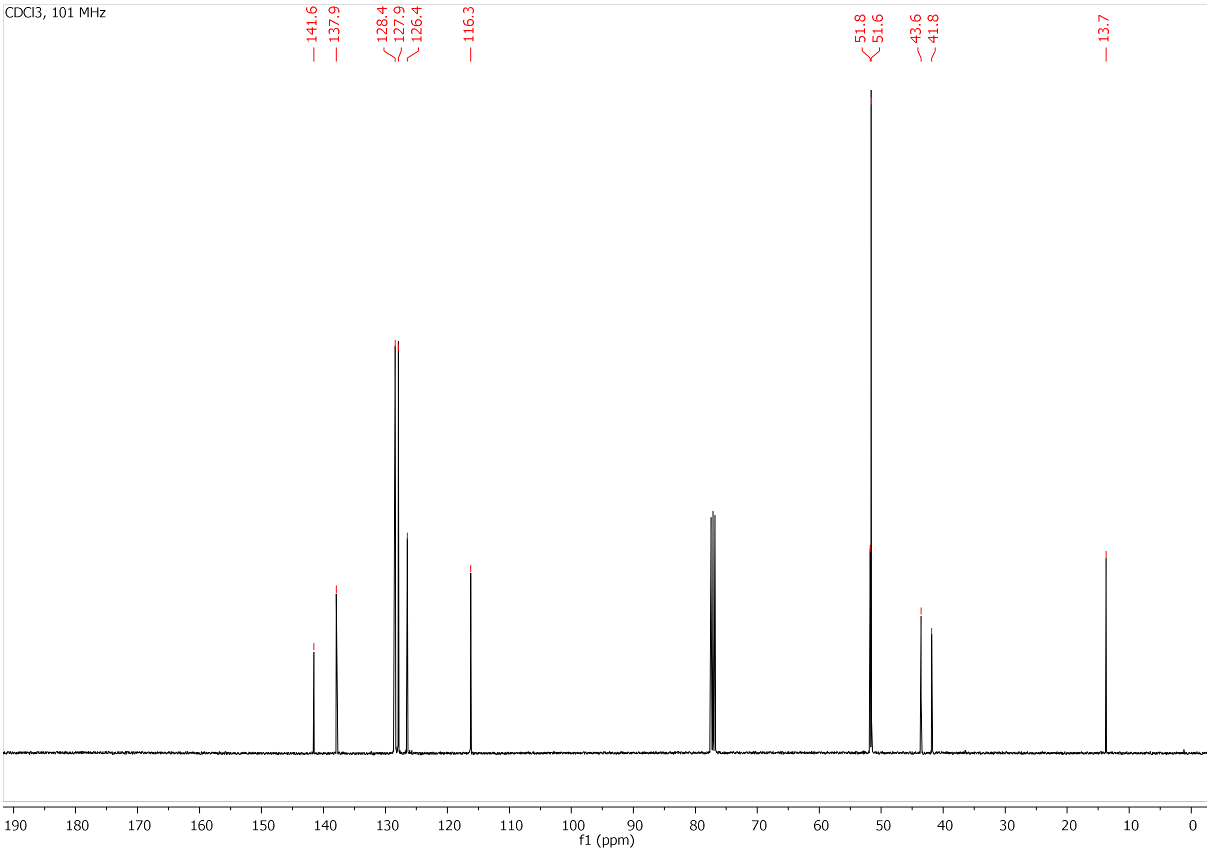

1-(3-(Cyclohex-2-en-1-yl)bicyclo[1.1.1]pentan-1-yl)propan-1-one (9j)

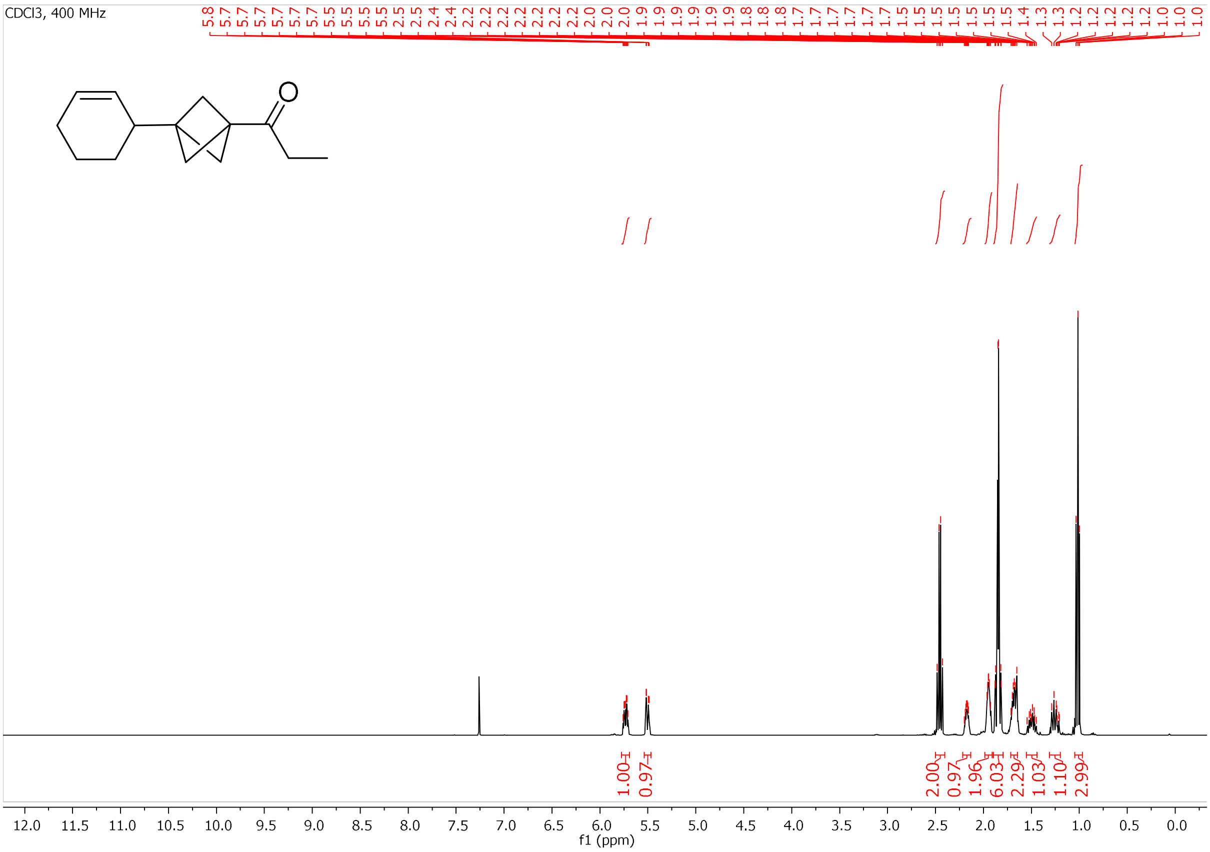

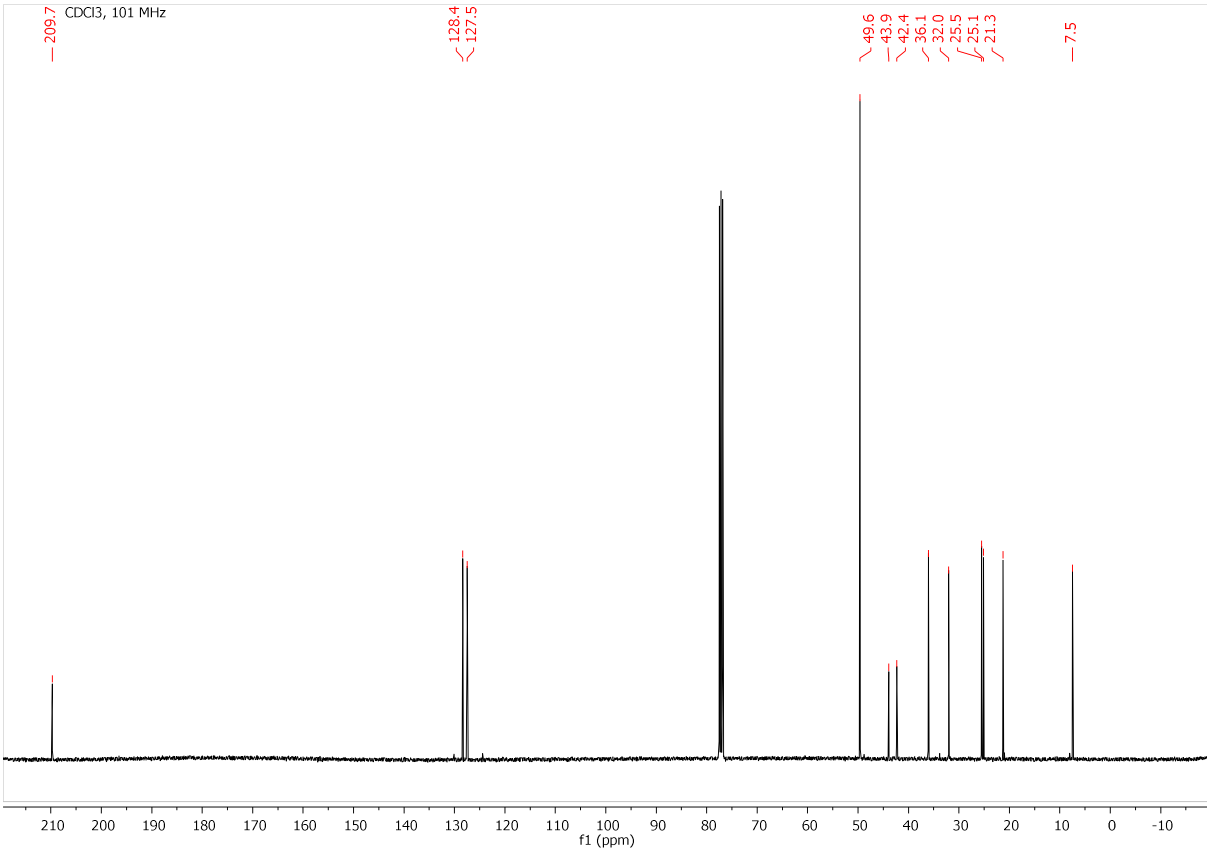

(3-(2-Methylbut-3-en-2-yl)bicyclo[1.1.1]pentan-1-yl)(phenyl)methanone (9k)

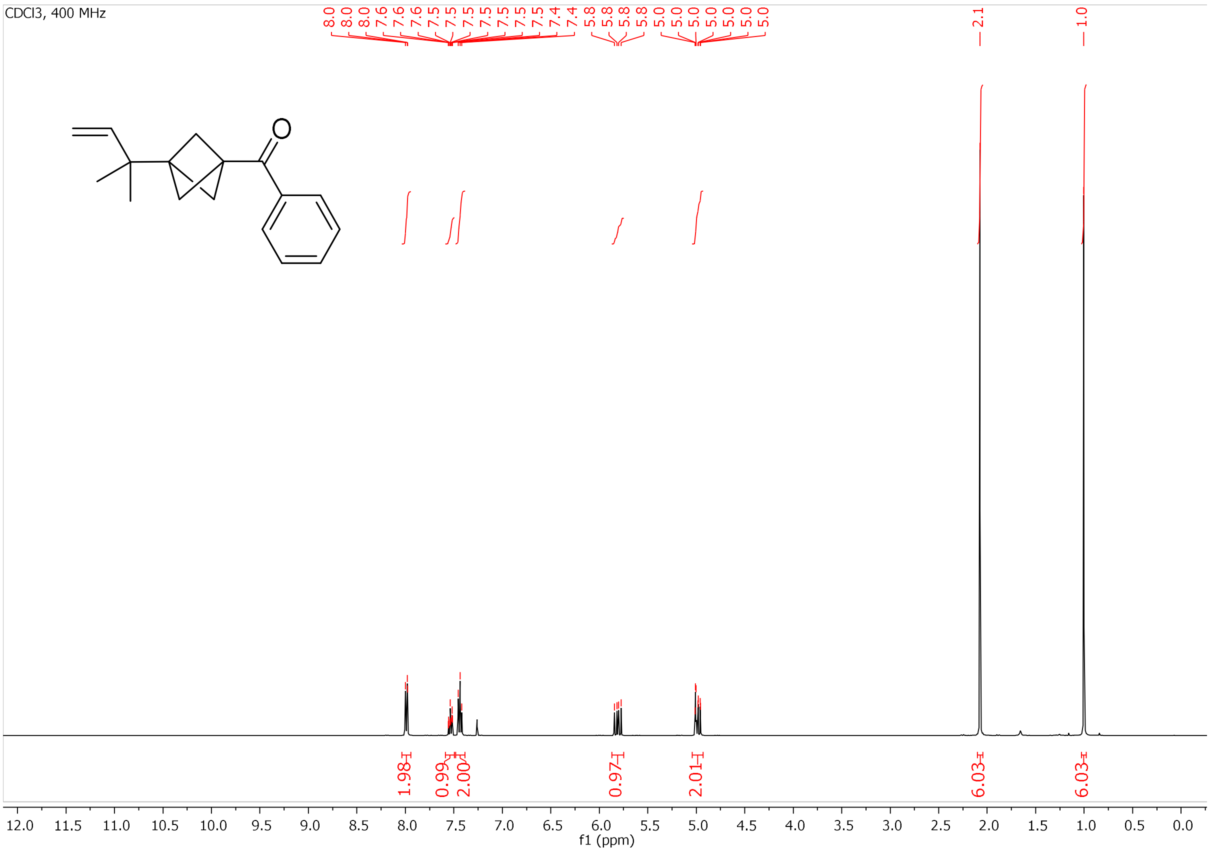

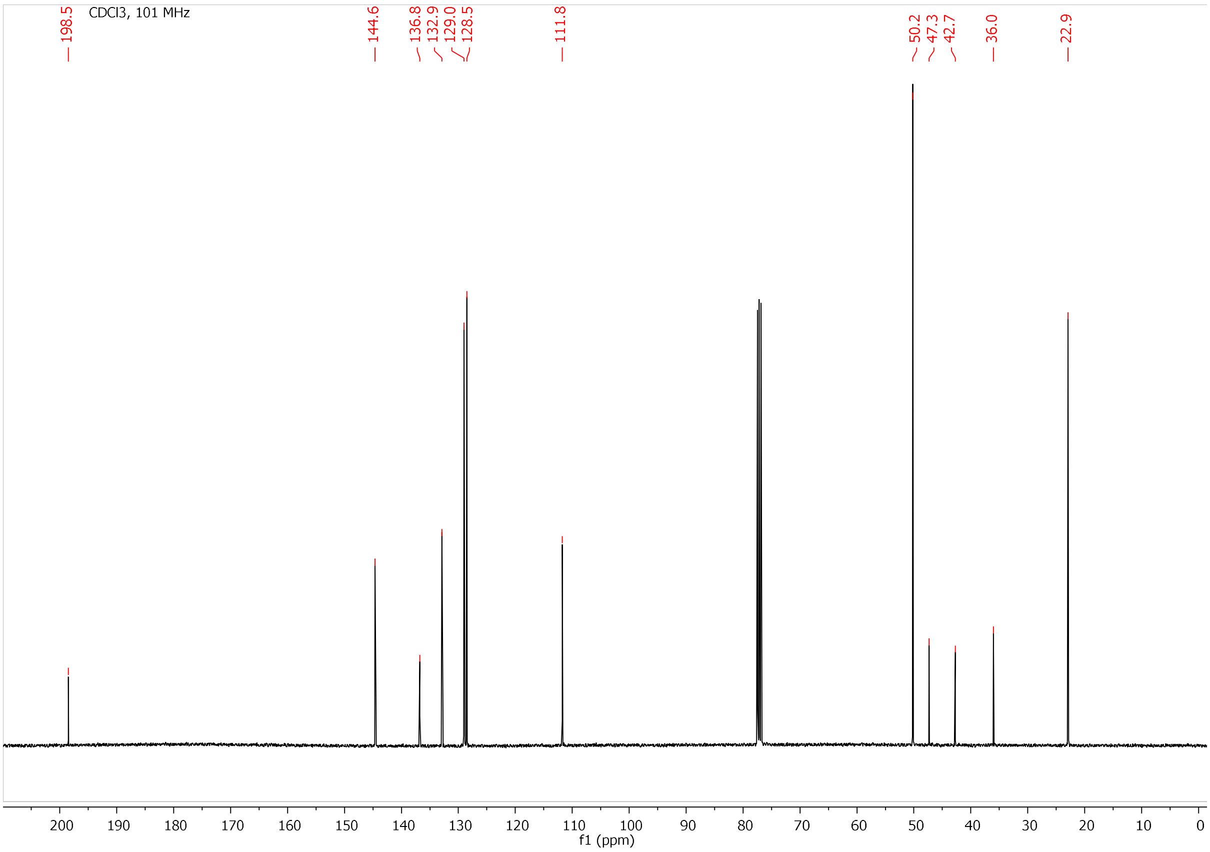

(3-(3,7-Dimethylocta-1,6-dien-3-yl)bicyclo[1.1.1]pentan-1-yl)(phenyl)methanone (9l)

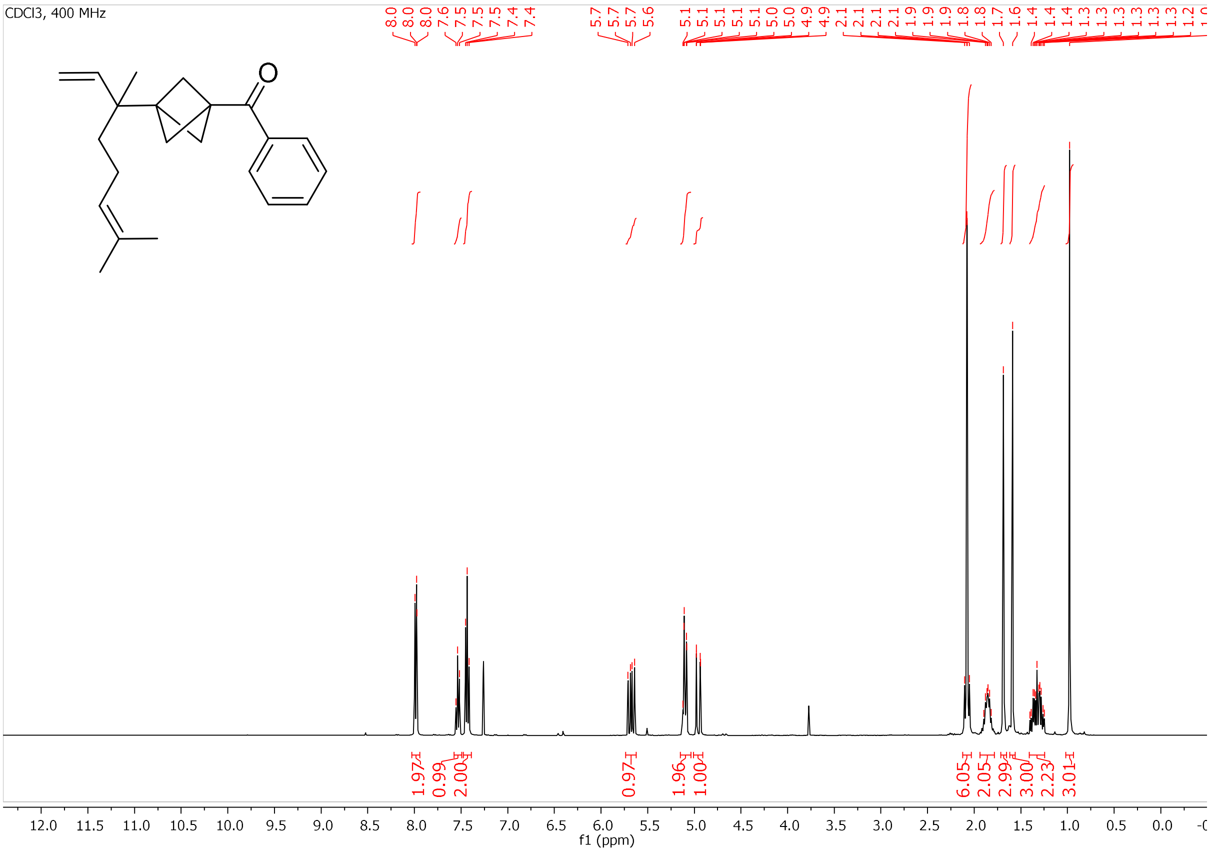

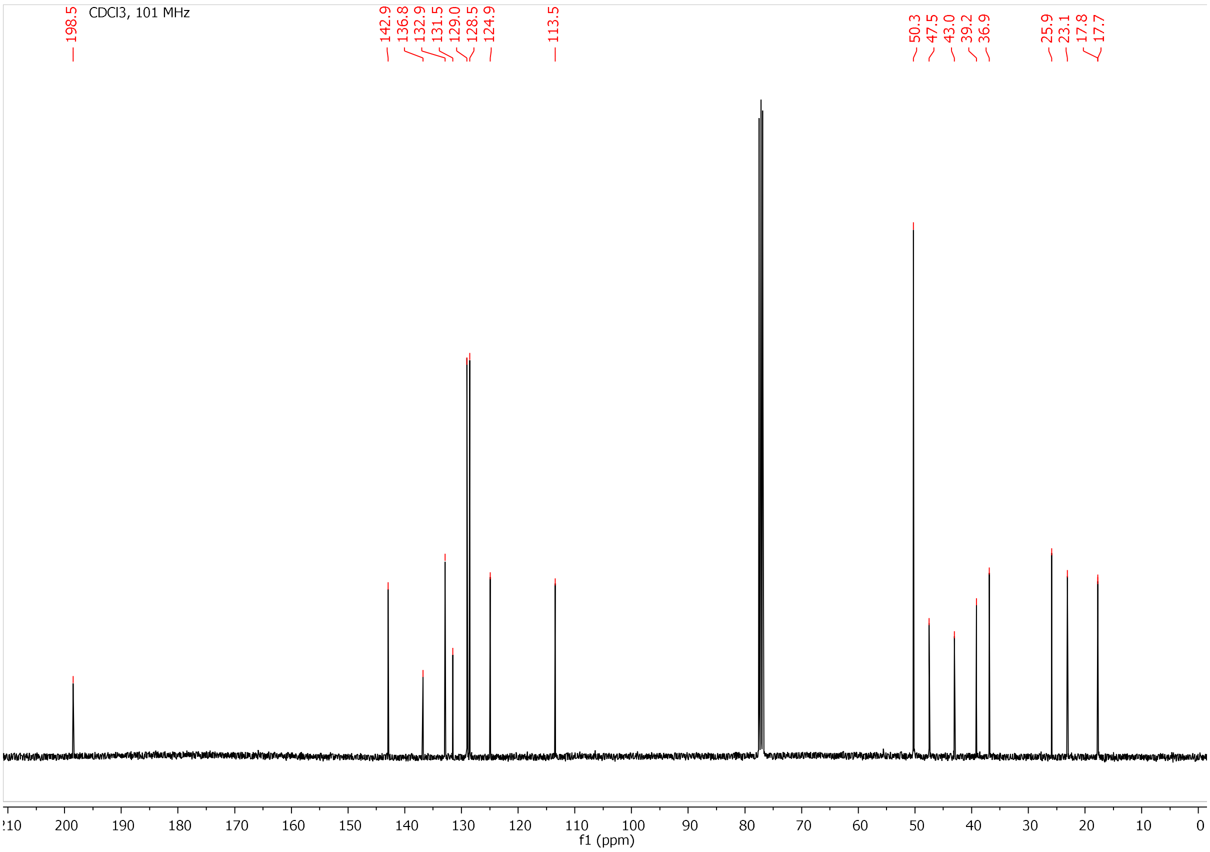

(1*R*,3*R*,5*R*)-3-(3-(4-Methoxyphenyl)bicyclo[1.1.1]pentan-1-yl)-6,6-dimethyl-2-methylene-bicyclo[3.1.1]heptane (9m)

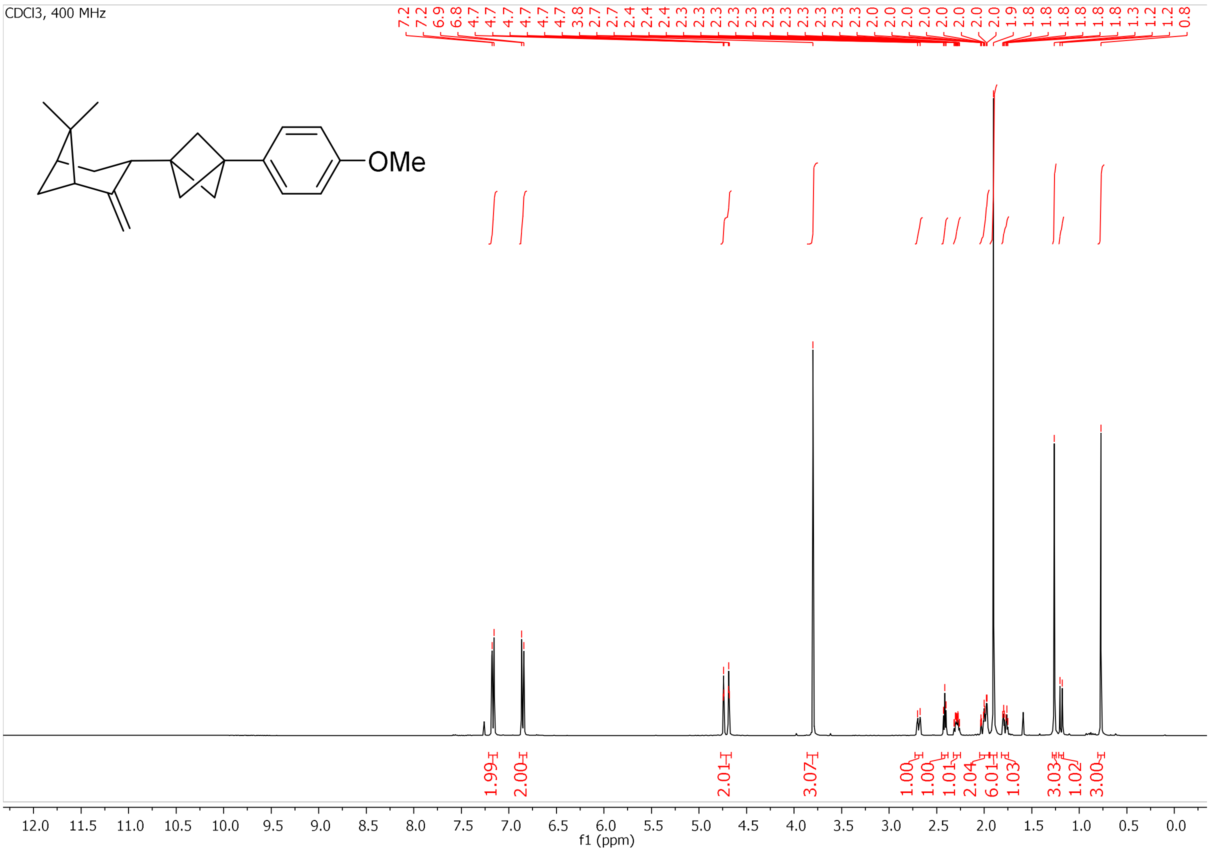

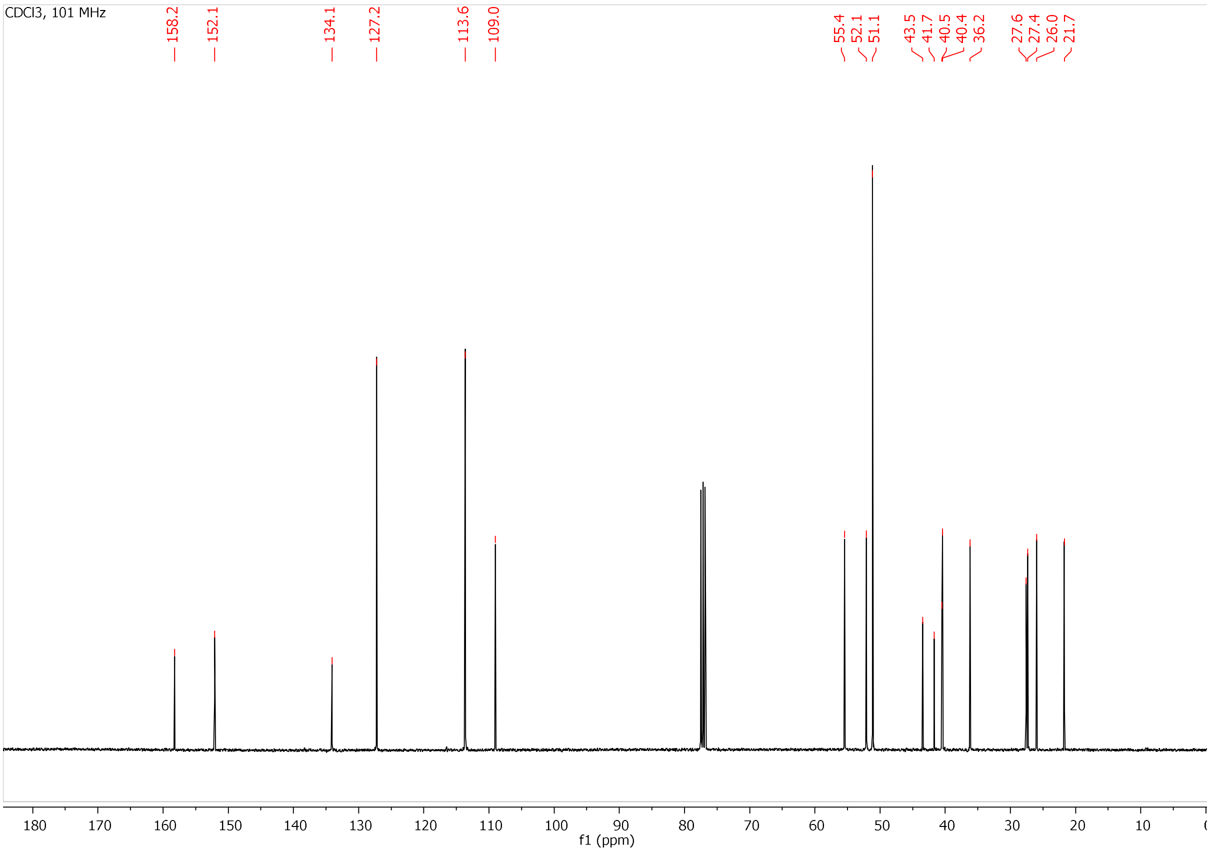

Ethyl 2-((3-((1*R*,3*R*,5*R*)-6,6-dimethyl-2-methylenebicyclo[3.1.1]heptan-3-yl)bicyclo-[1.1.1]pentan-1-yl)methyl)acrylate (9n)

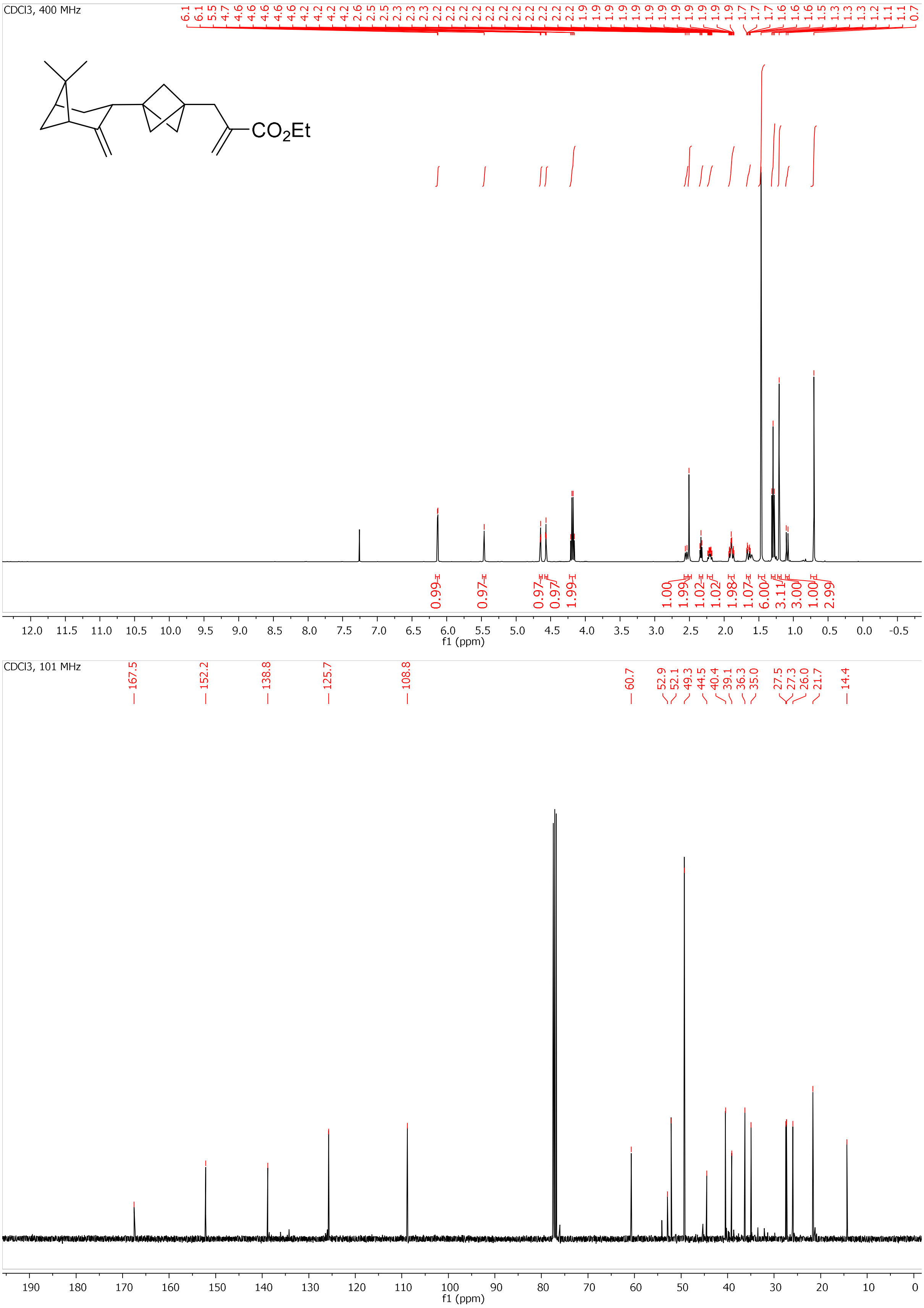

Ethyl 6-(3-(quinolin-2-yl)bicyclo[1.1.1]pentan-1-yl)cyclohex-1-ene-1-carboxylate (9o)

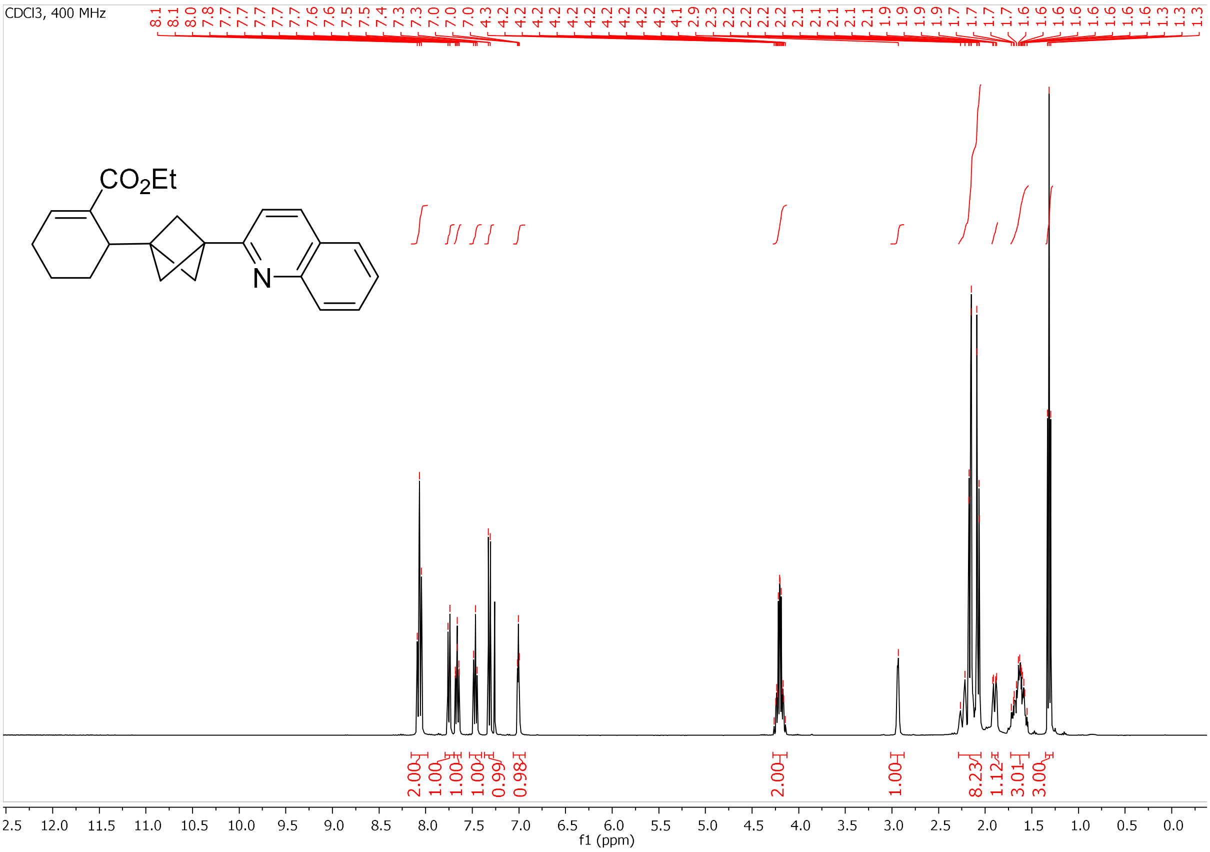

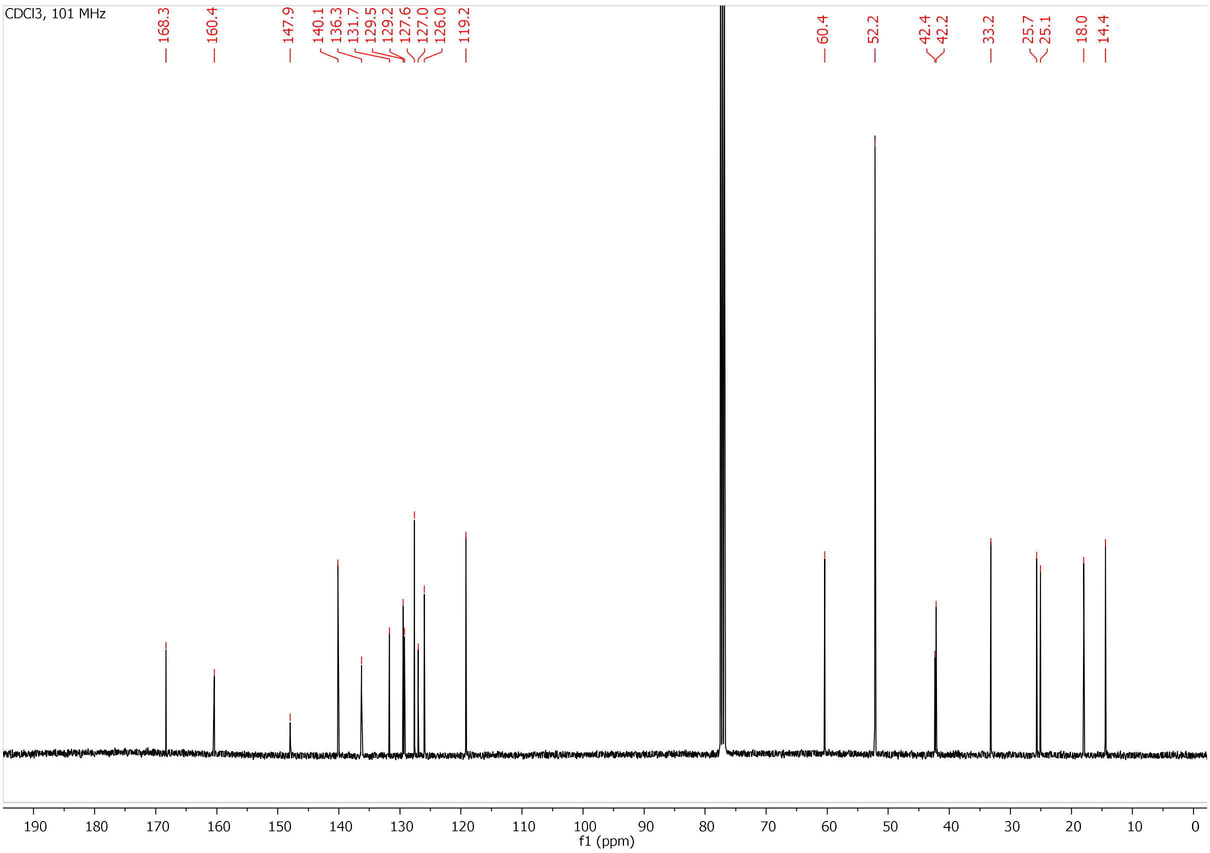

5-(3-(Cyclohex-2-en-1-yl)bicyclo[1.1.1]pentan-1-yl)cyclopent-1-ene-1-carbonitrile (9p)

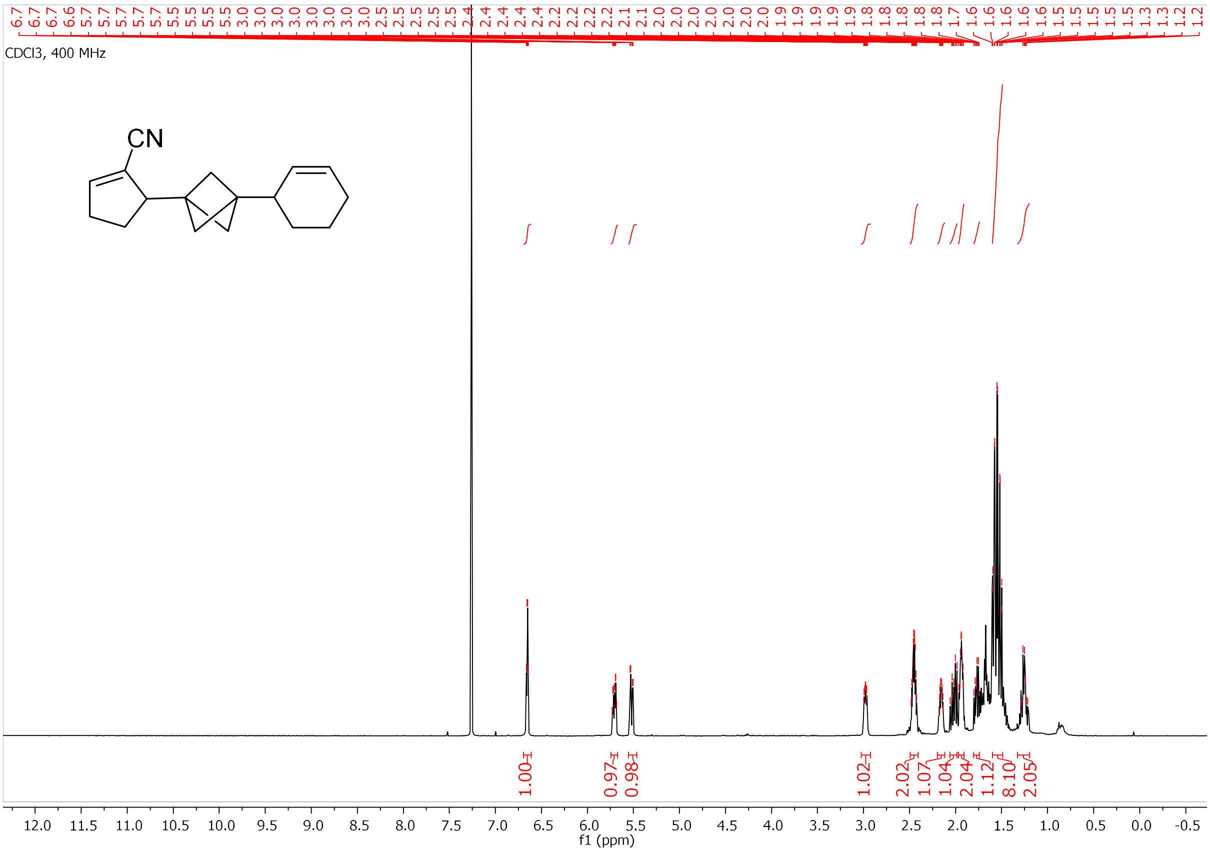

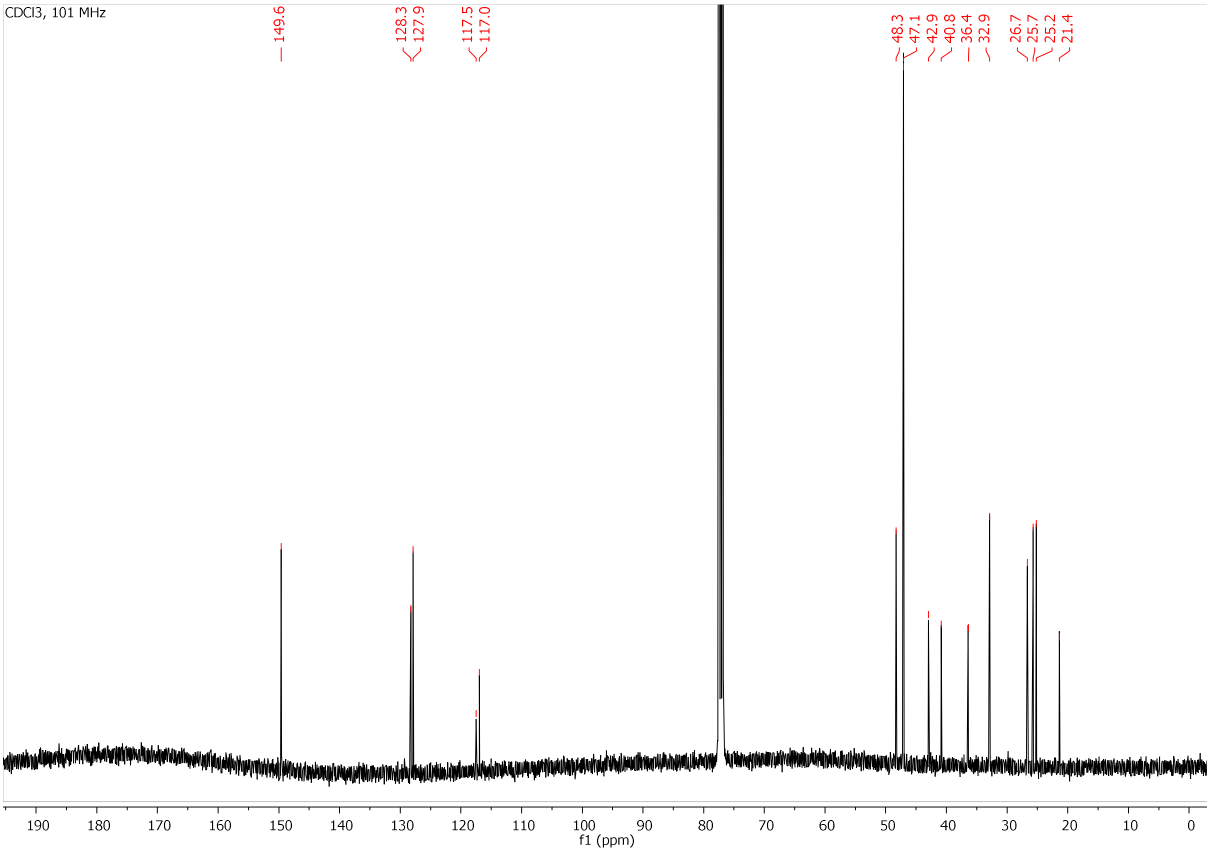

5-(3-(4-Methoxyphenyl)bicyclo[1.1.1]pentan-1-yl)cyclopent-1-ene-1-carbonitrile (9q)

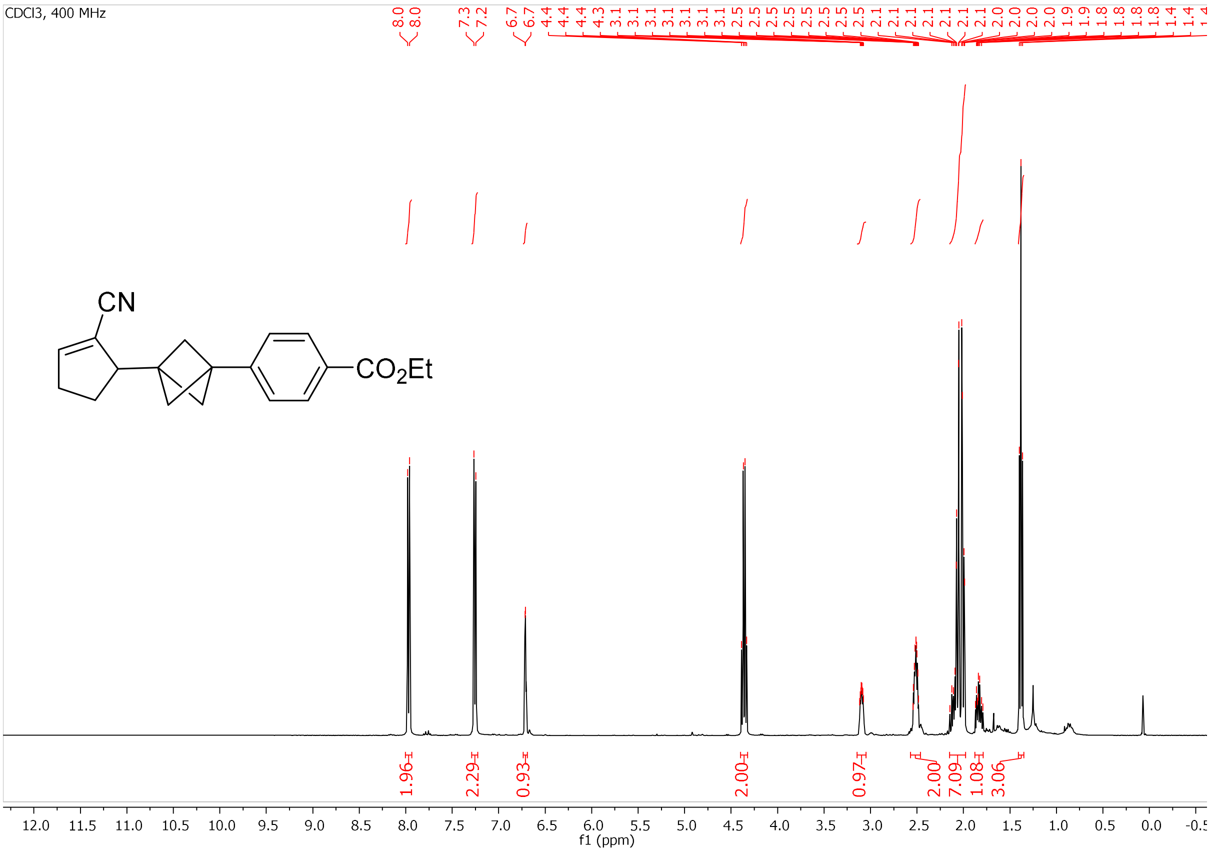

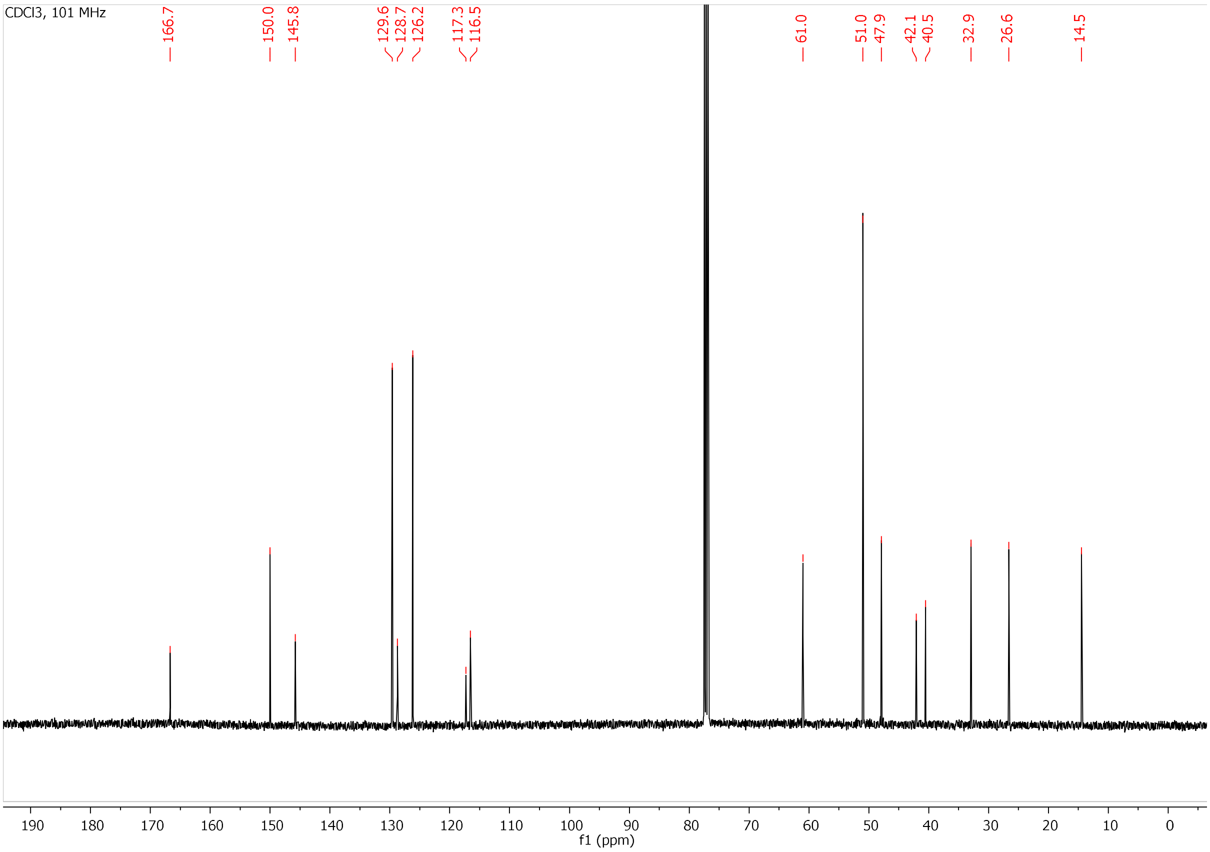

Phenyl(3-(1-(triisopropylsilyl)propa-1,2-dien-1-yl)bicyclo[1.1.1]pentan-1-yl)methanone (9r)

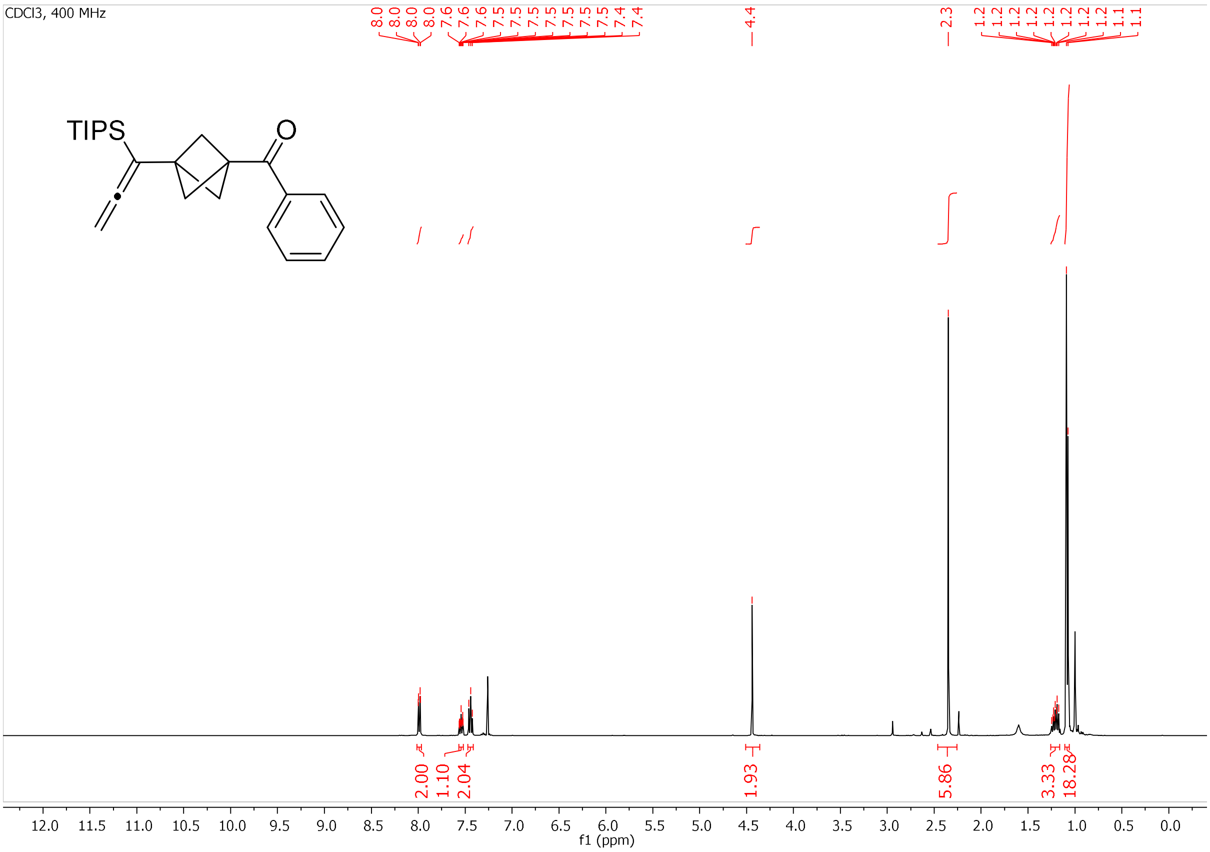

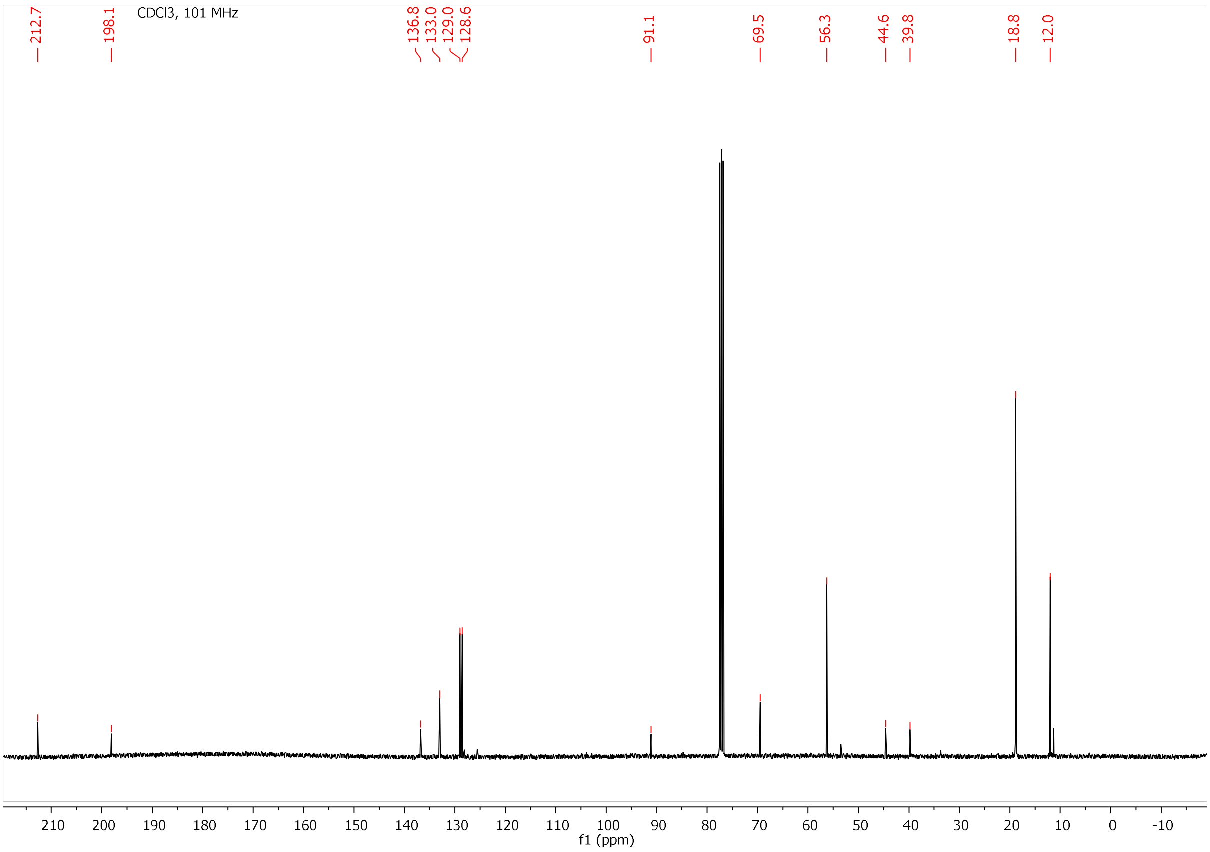

Phenyl(3-(3-(triisopropylsilyl)prop-2-yn-1-yl)bicyclo[1.1.1]pentan-1-yl)methanone (9s)

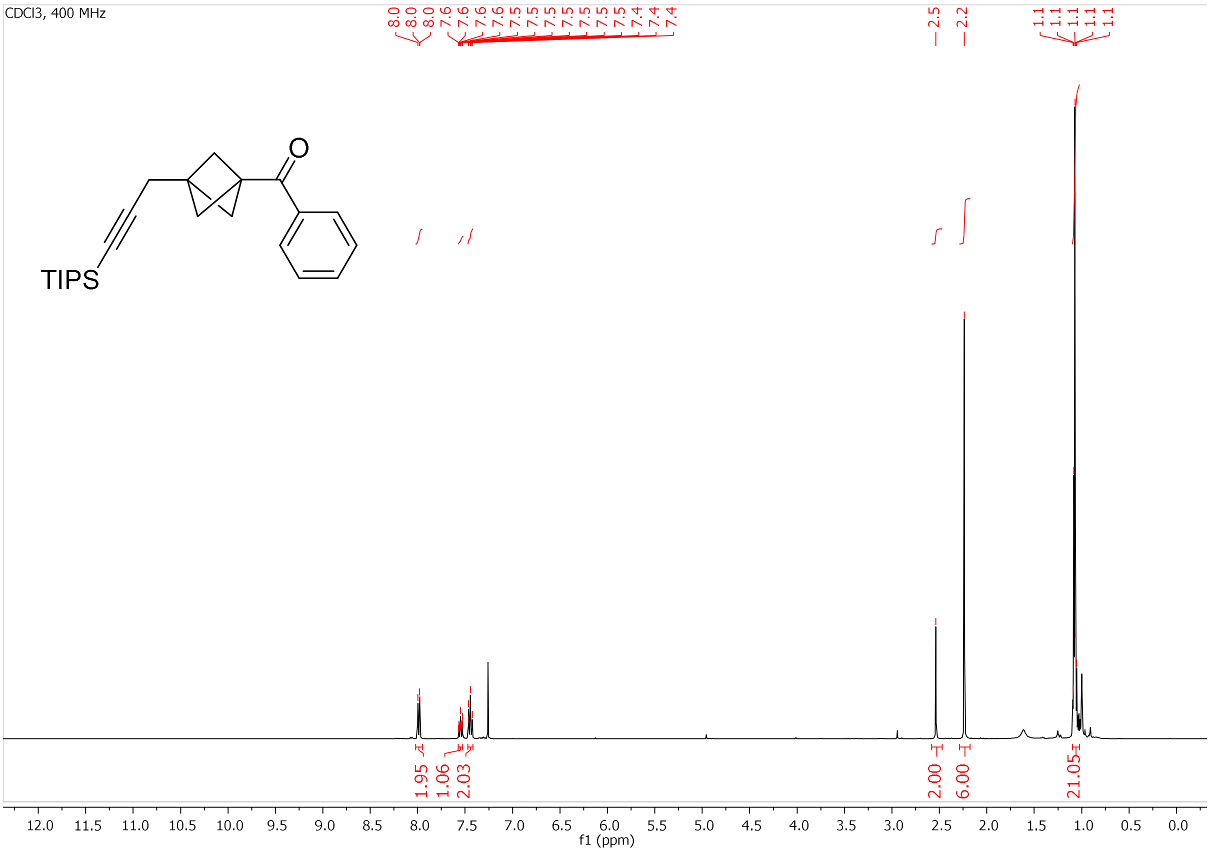

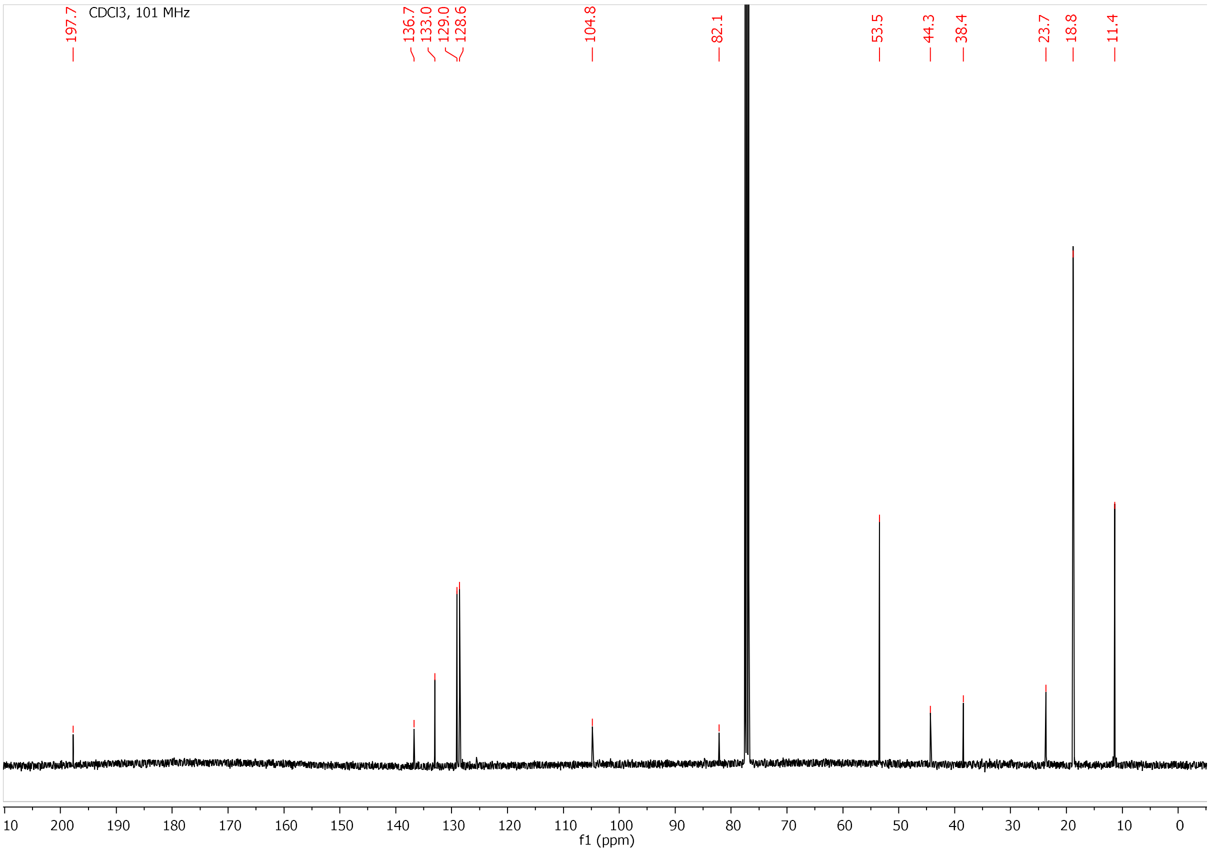

2-(Bicyclo[1.1.1]pentan-1-yl)cyclohexan-1-one (14a)

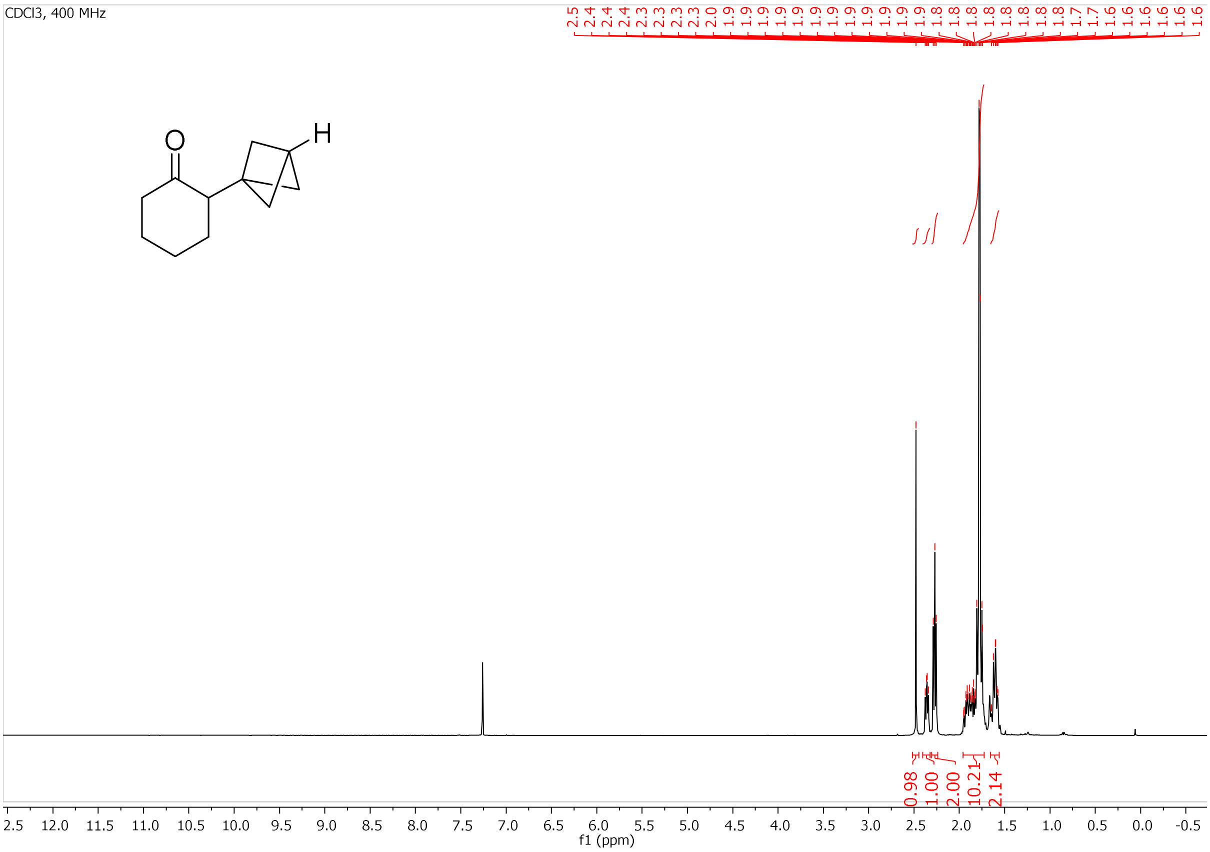

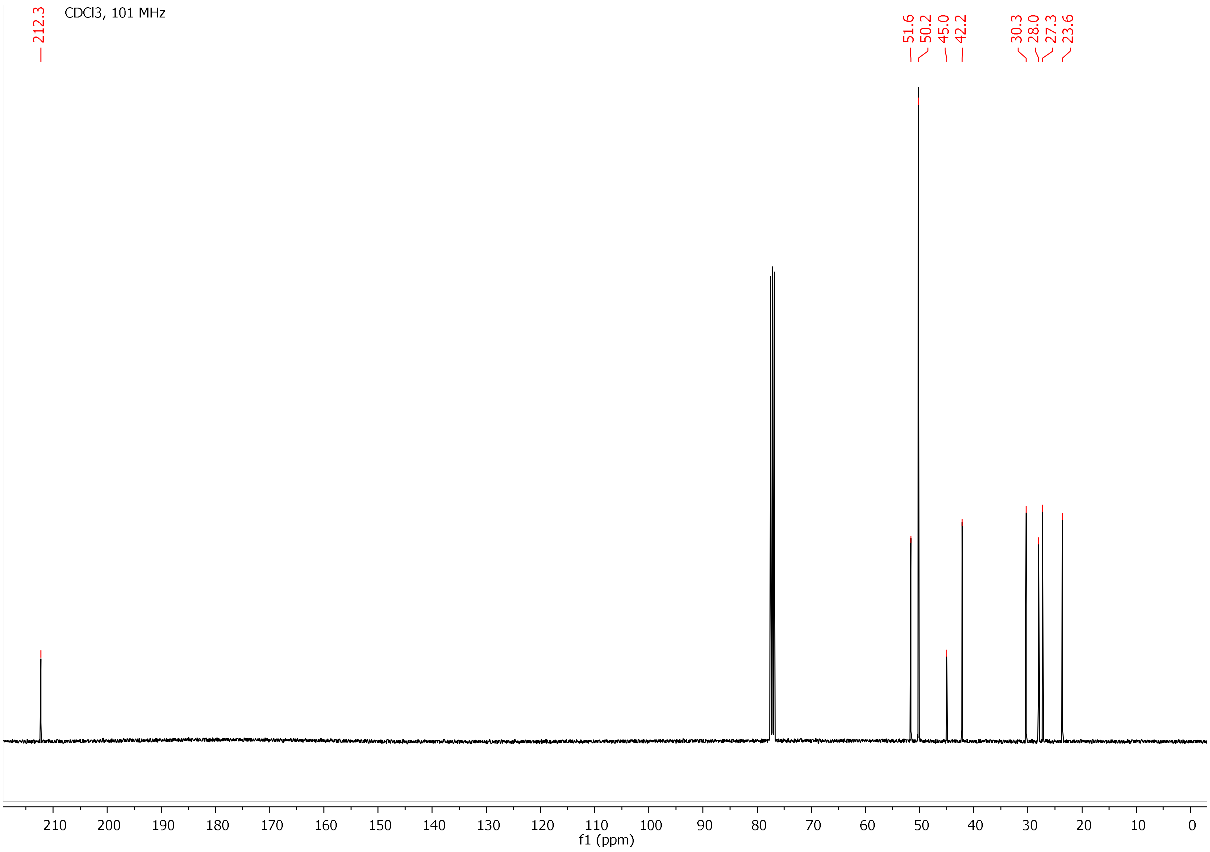

2-(3-Allylbicyclo[1.1.1]pentan-1-yl)cyclohexan-1-one (14b)

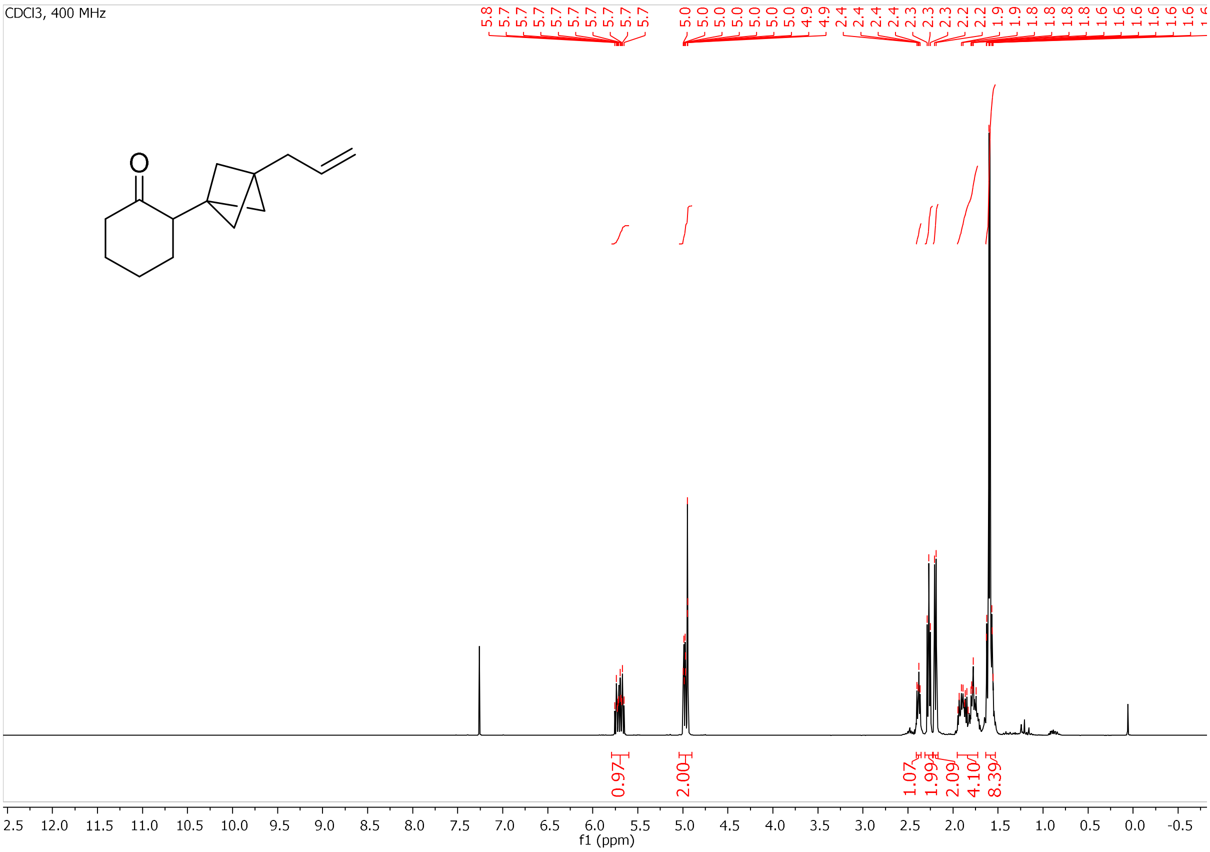

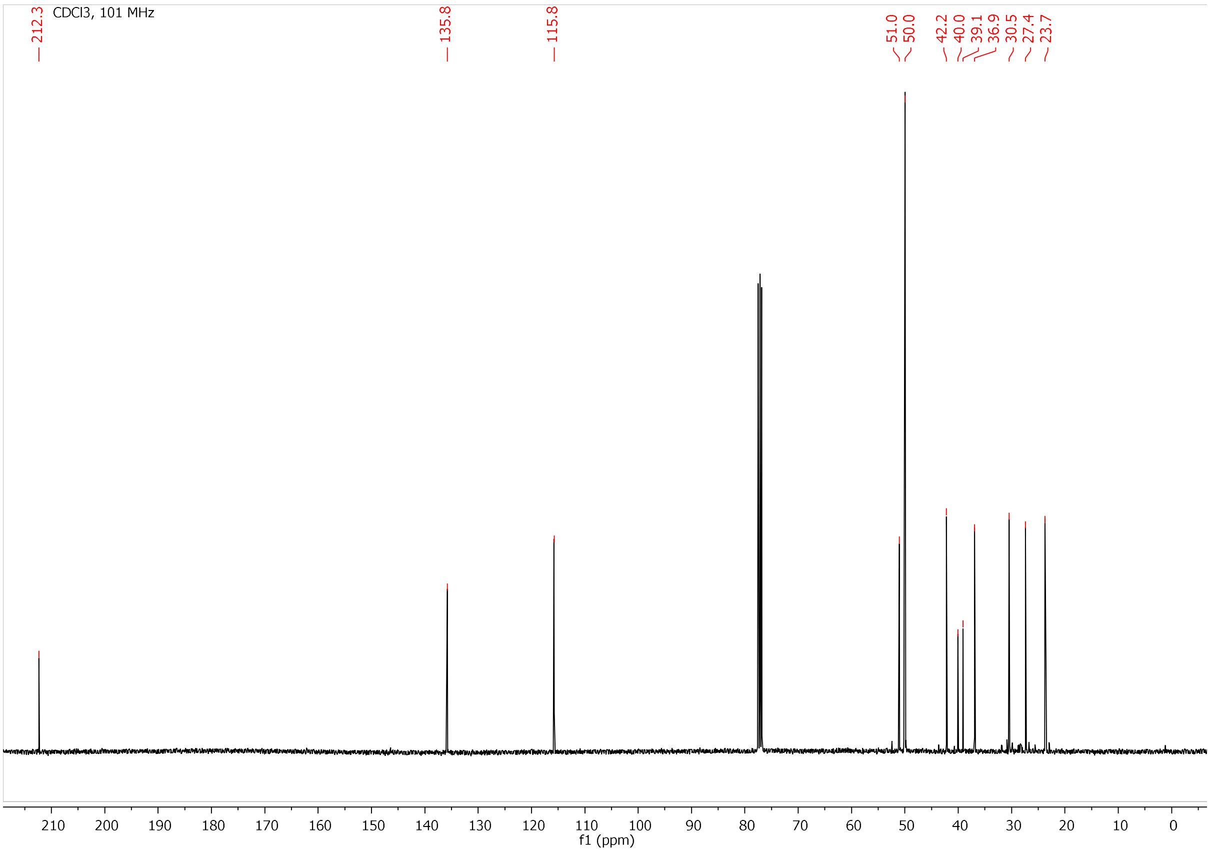

2-(3-(4-Methoxyphenyl)bicyclo[1.1.1]pentan-1-yl)cyclohexan-1-one (14c)

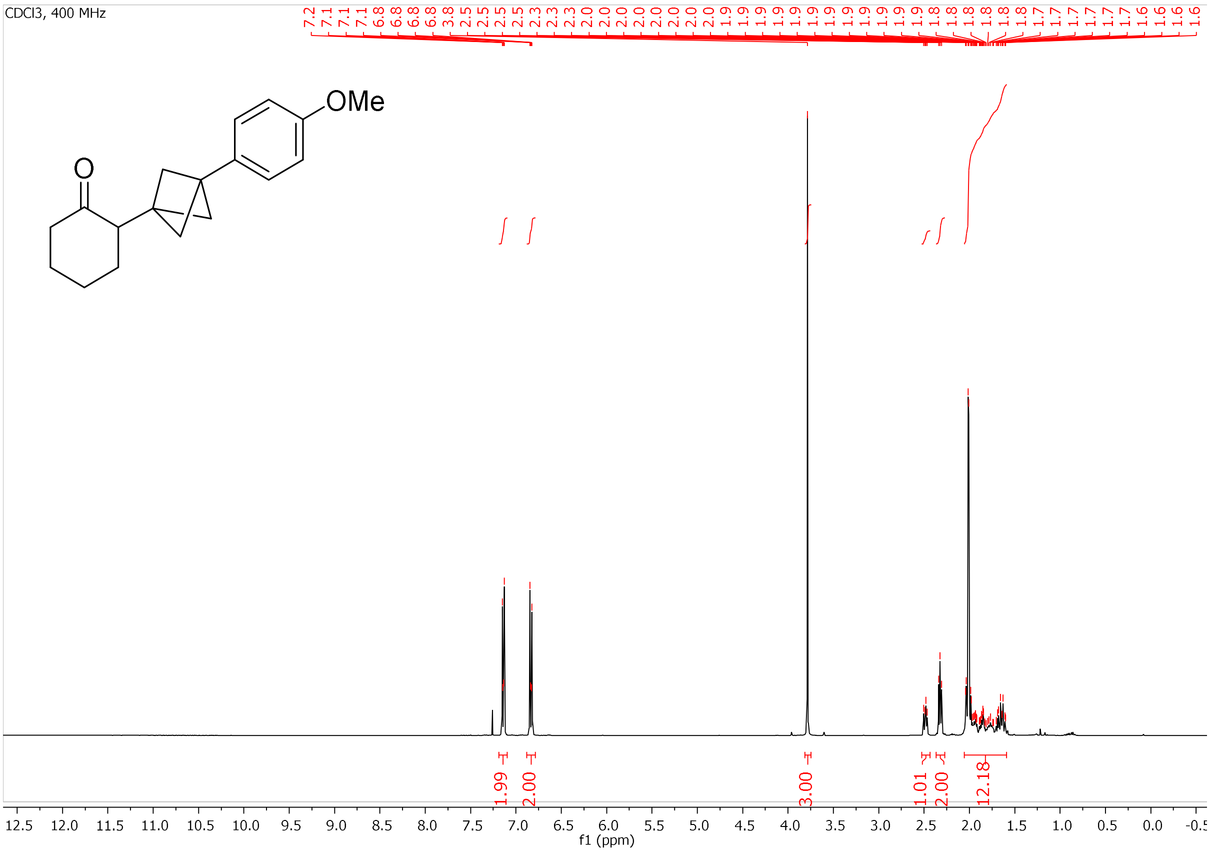

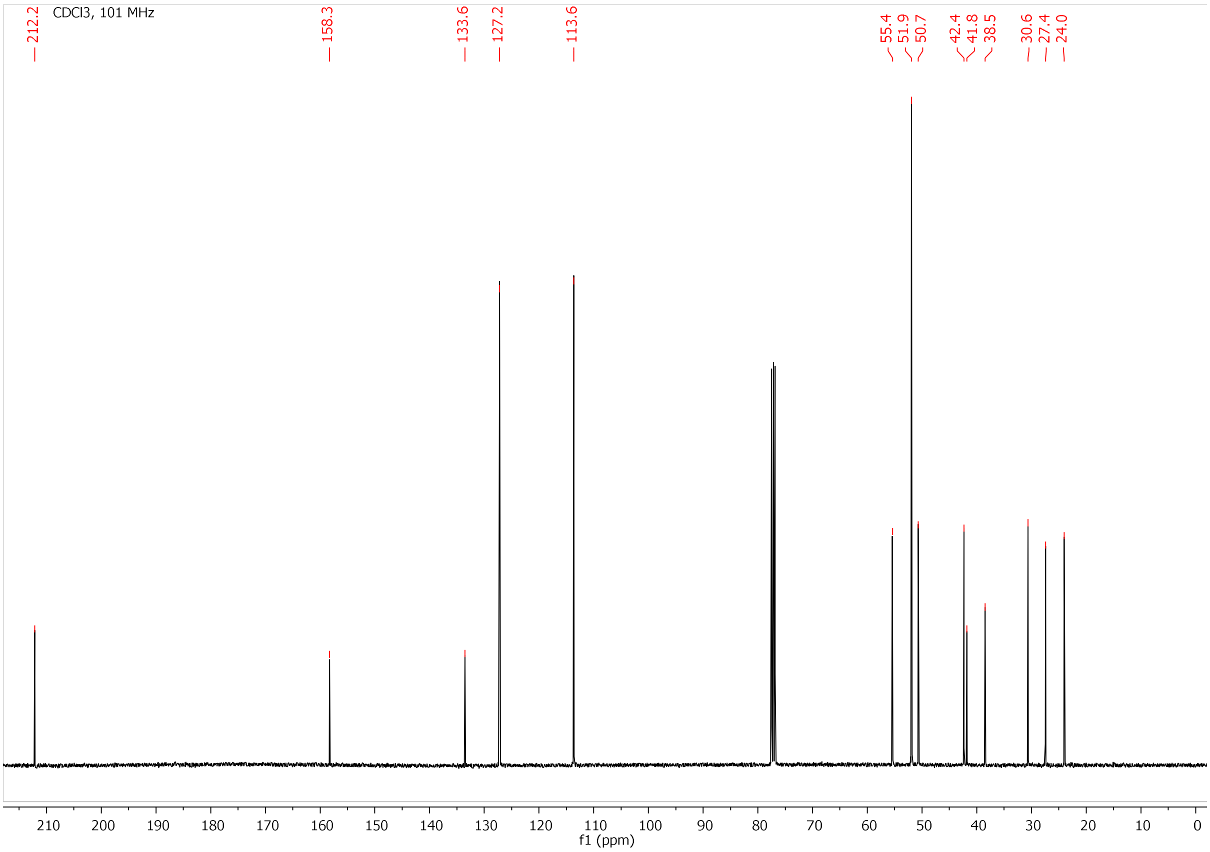

2-(3-Benzoylbicyclo[1.1.1]pentan-1-yl)cyclohexan-1-one (14d)

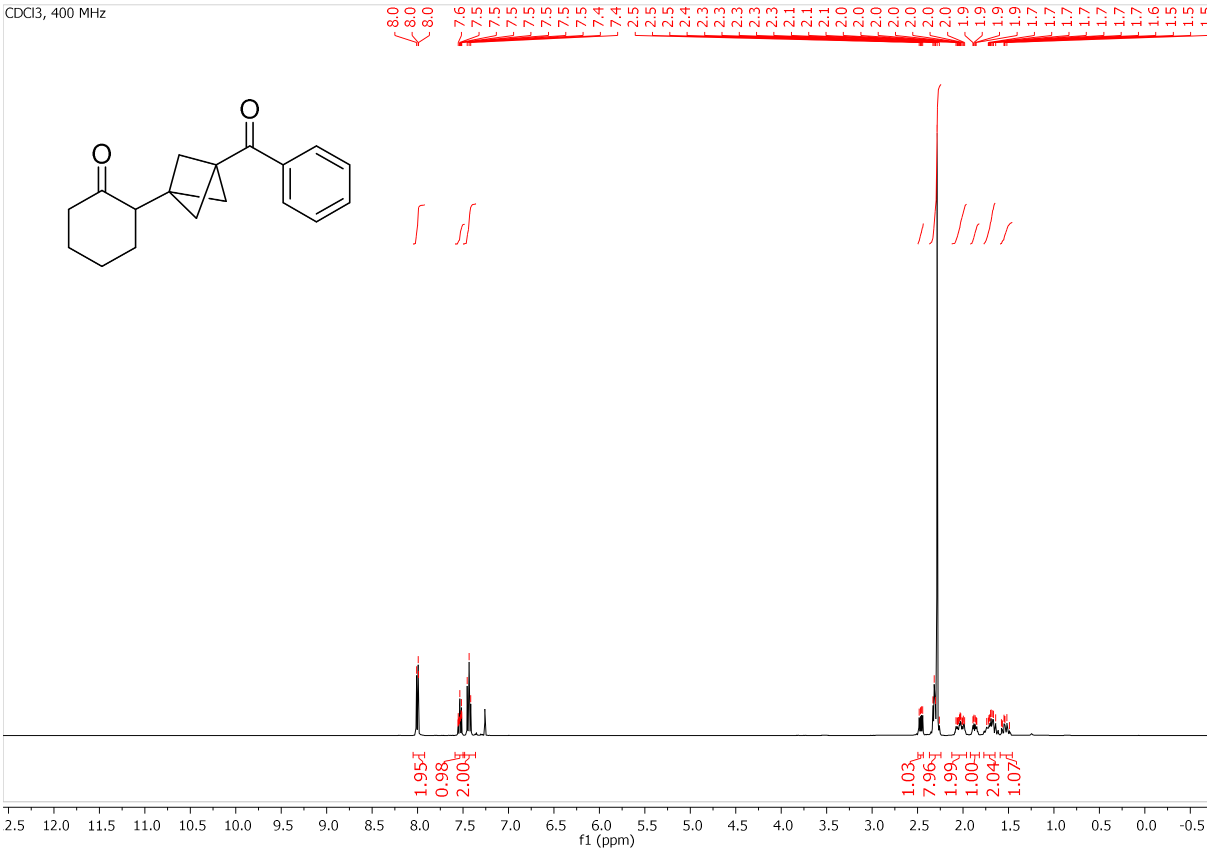

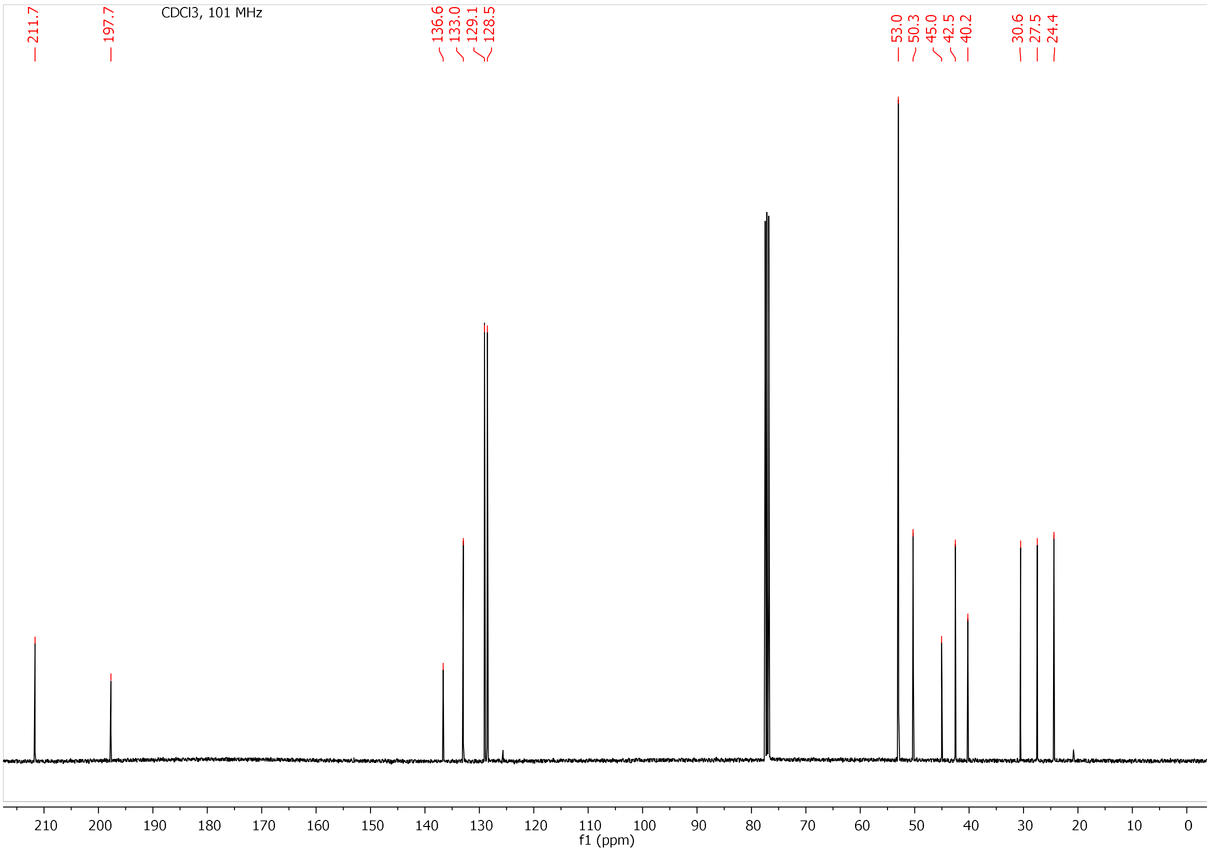

3-(2-Oxocyclohexyl)bicyclo[1.1.1]pentane-1-carbonitrile (14e)

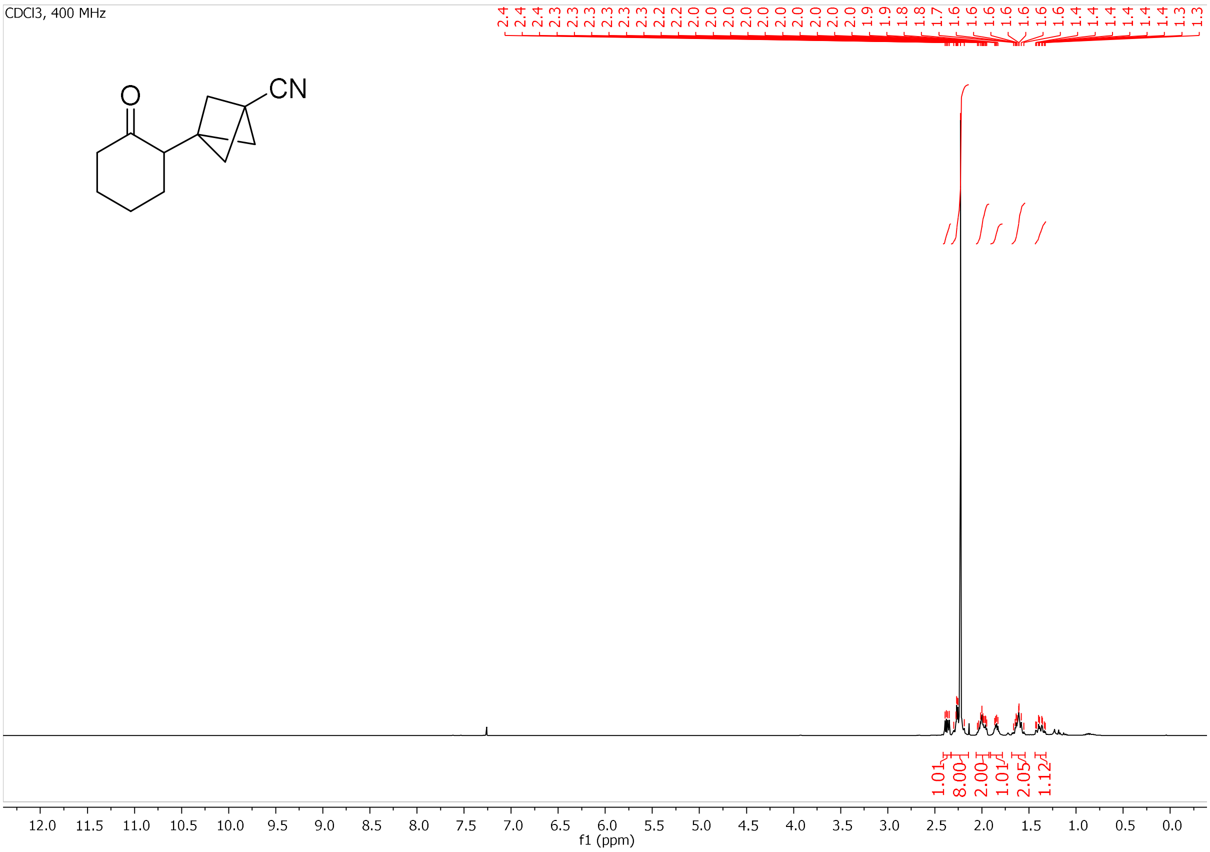

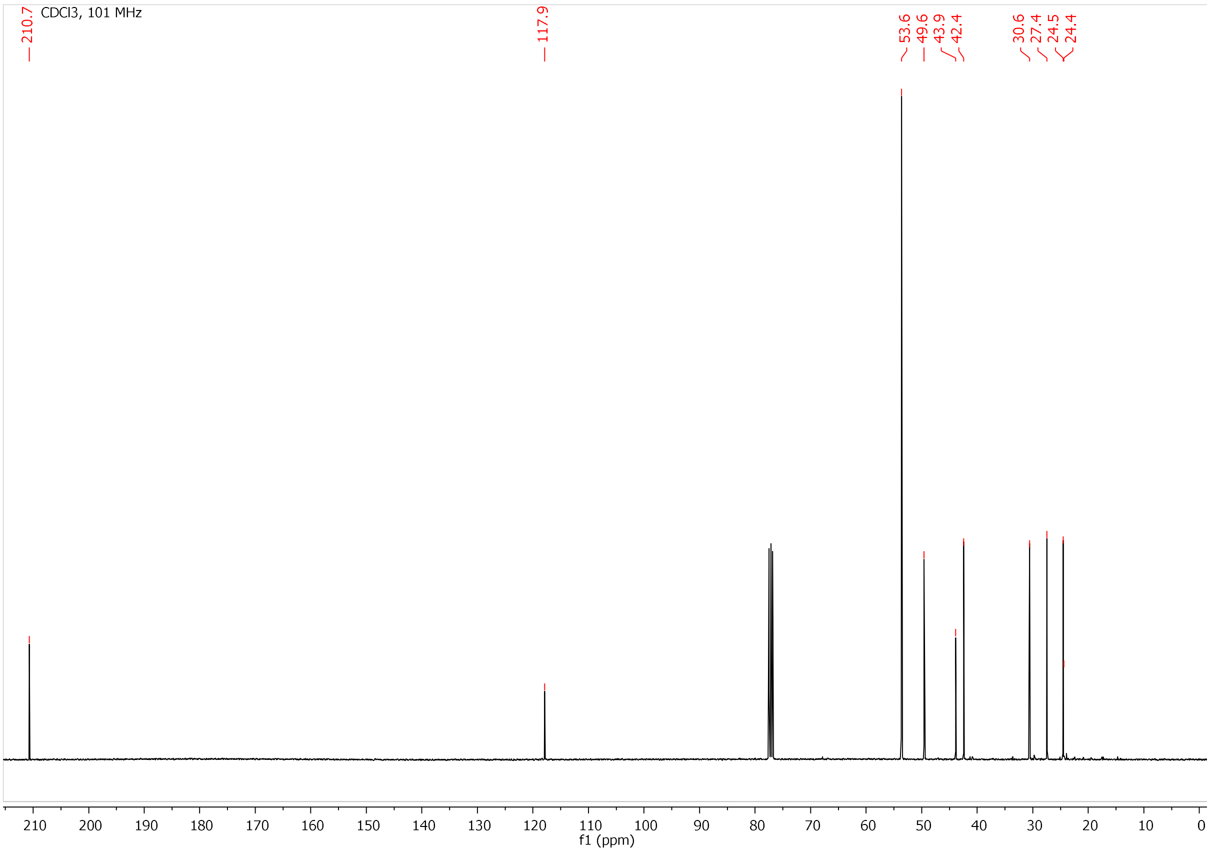

1-(3-Allylbicyclo[1.1.1]pentan-1-yl)-3-cyclohexylpropan-2-one (14f)

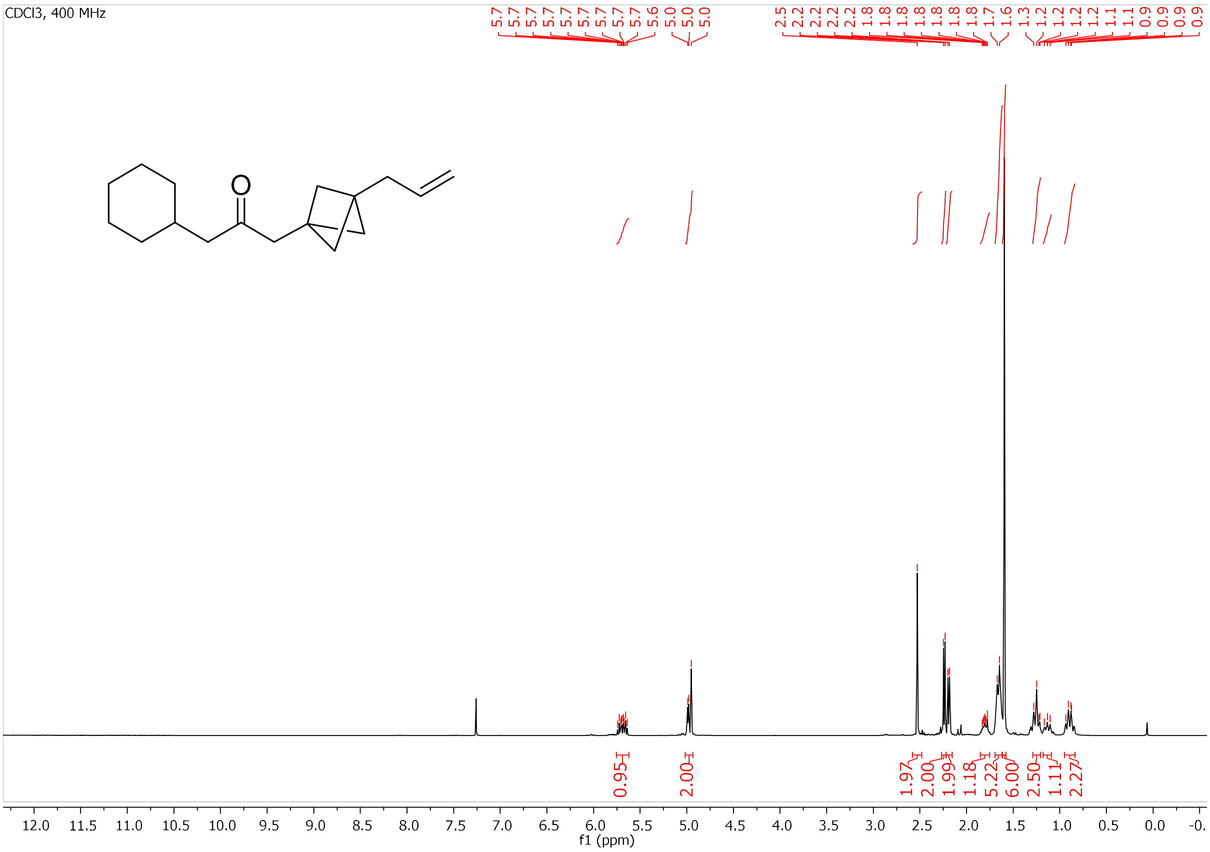

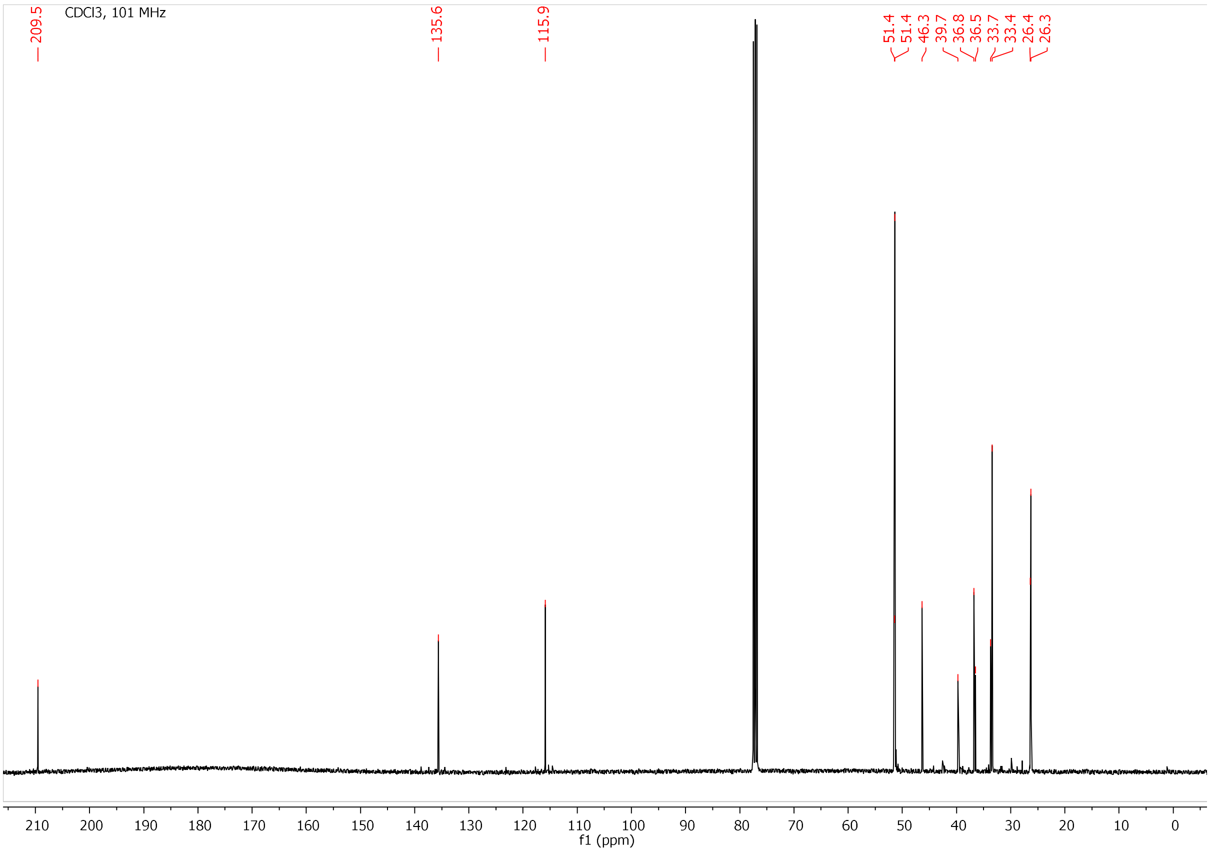

1-(Bicyclo[1.1.1]pentan-1-yl)-4-(2,6,6-trimethylcyclohex-1-en-1-yl)butan-2-one (14g)

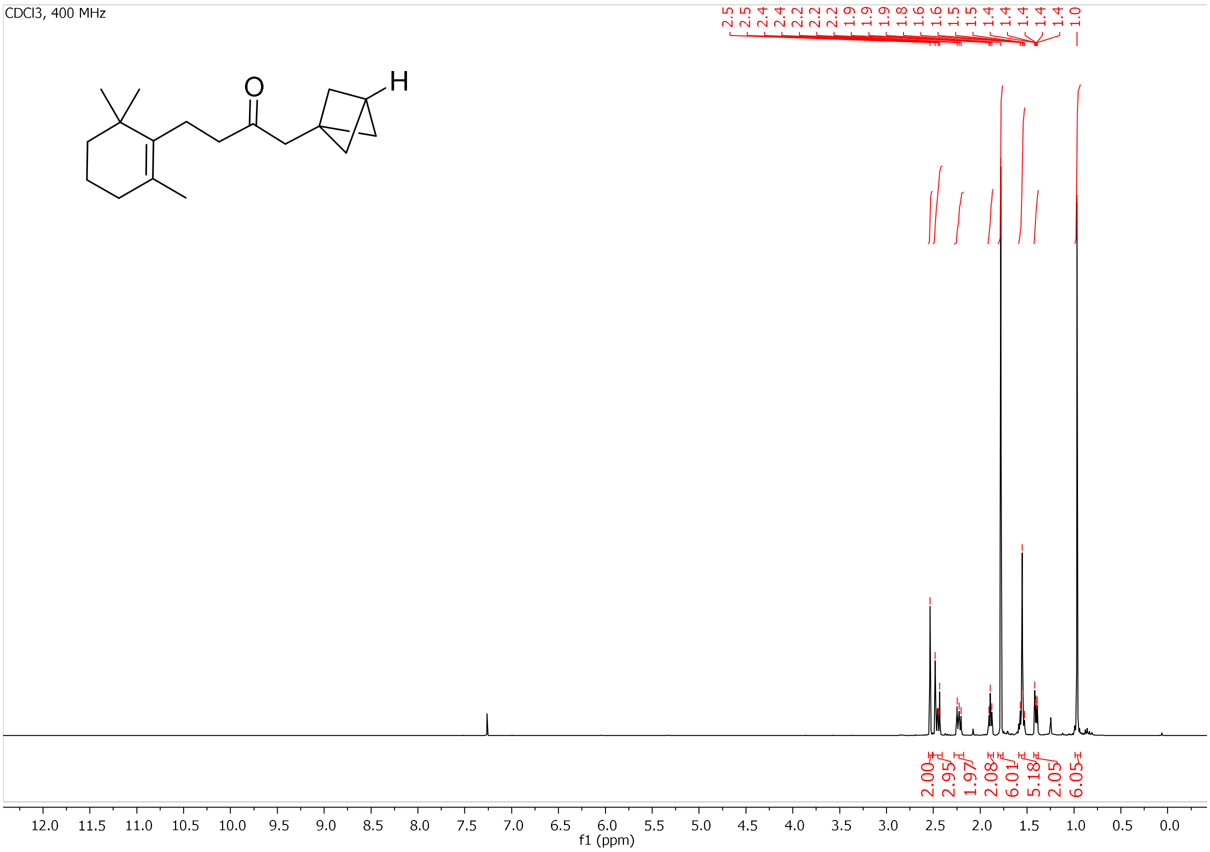

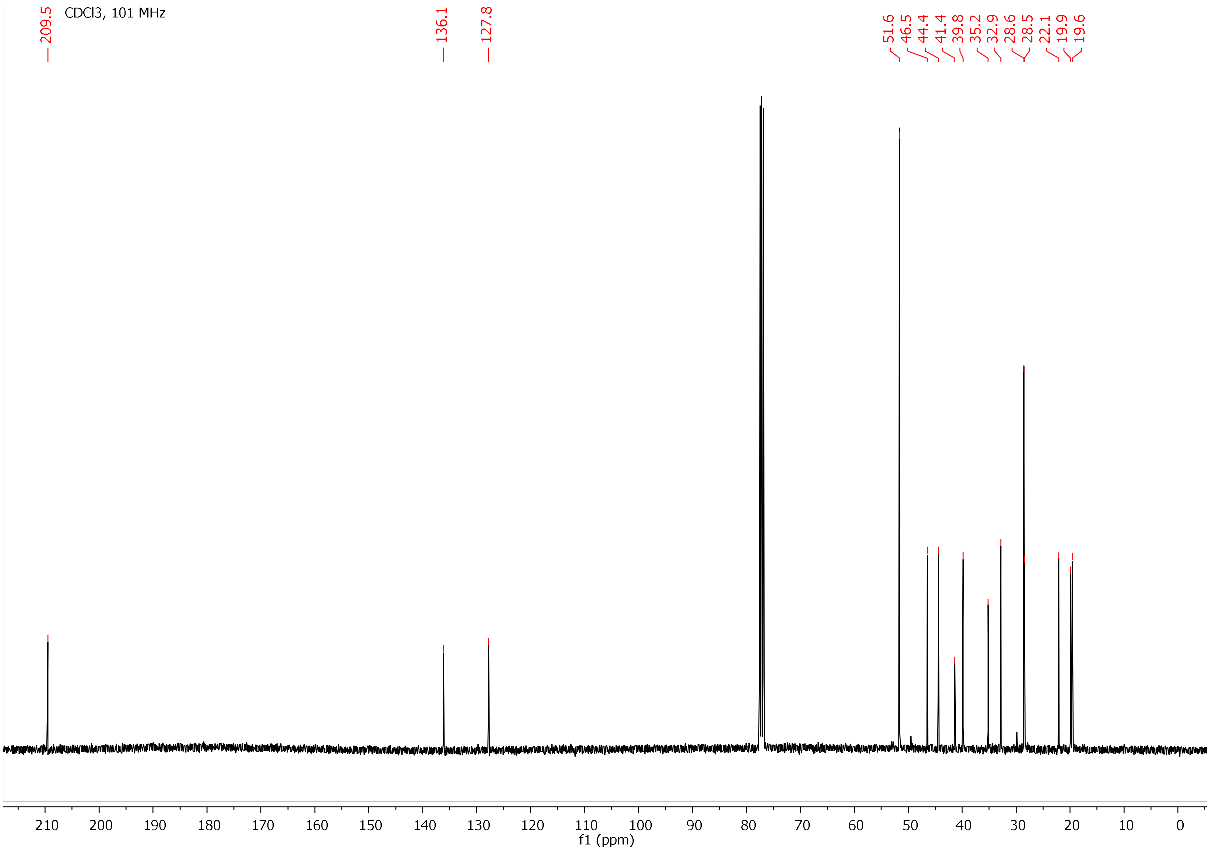

2-(3-Allylbicyclo[1.1.1]pentan-1-yl)-2-methyl-1-phenylpropan-1-one (14h)

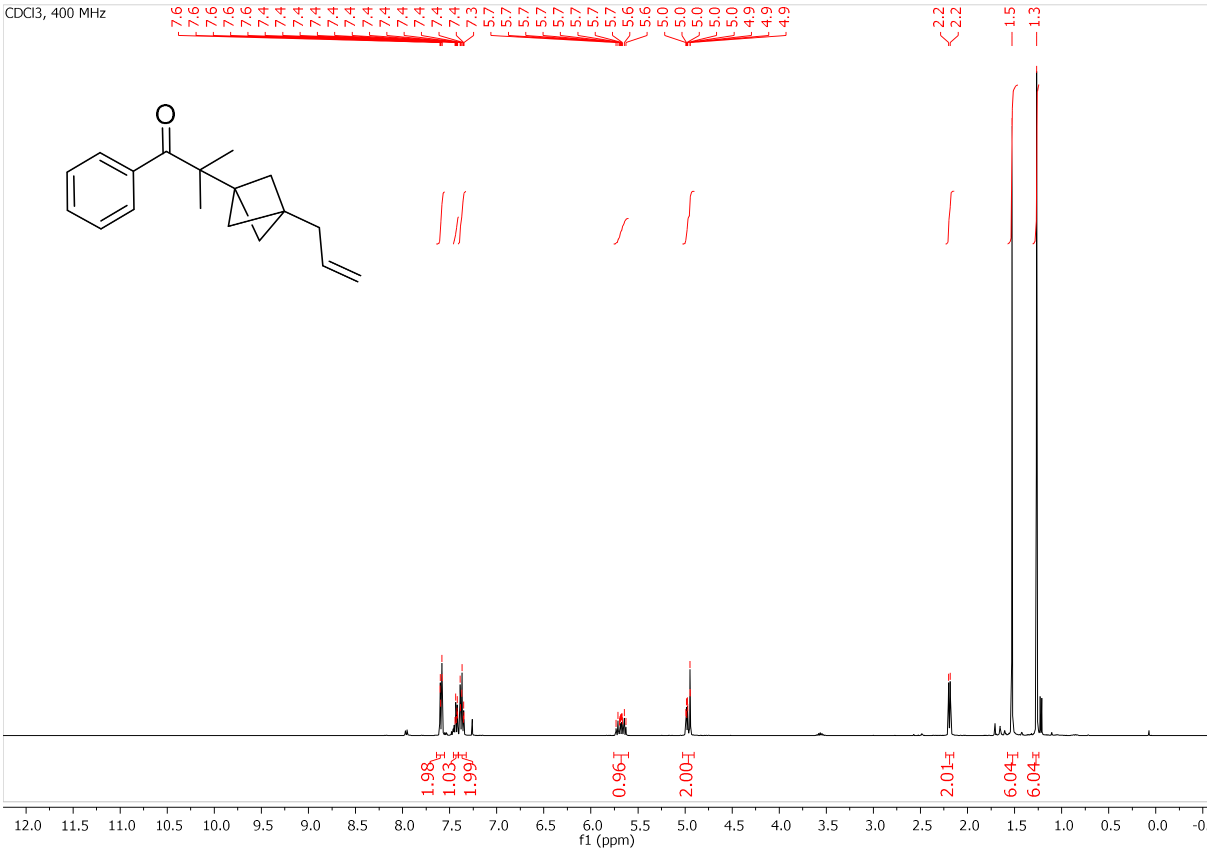

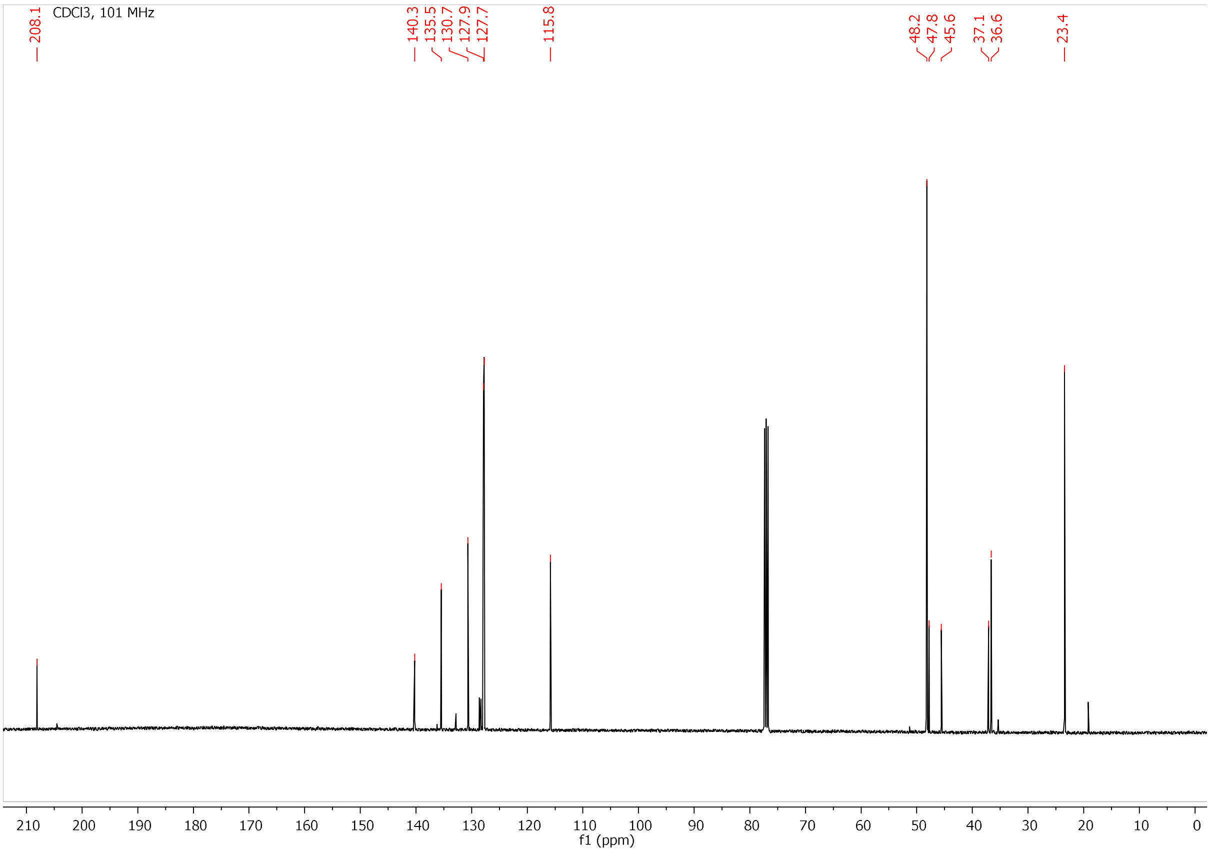

6-(3-Allylbicyclo[1.1.1]pentan-1-yl)cyclohex-2-en-1-one (14i)

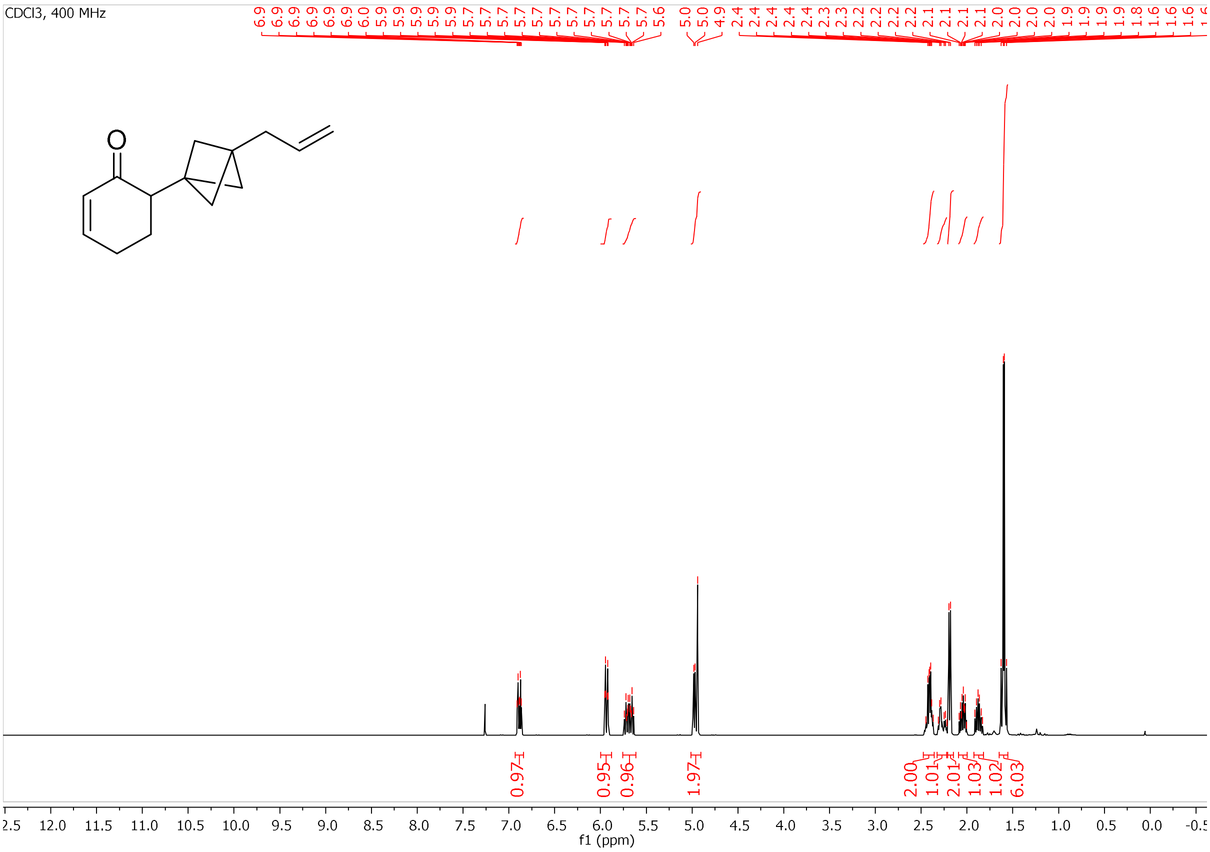

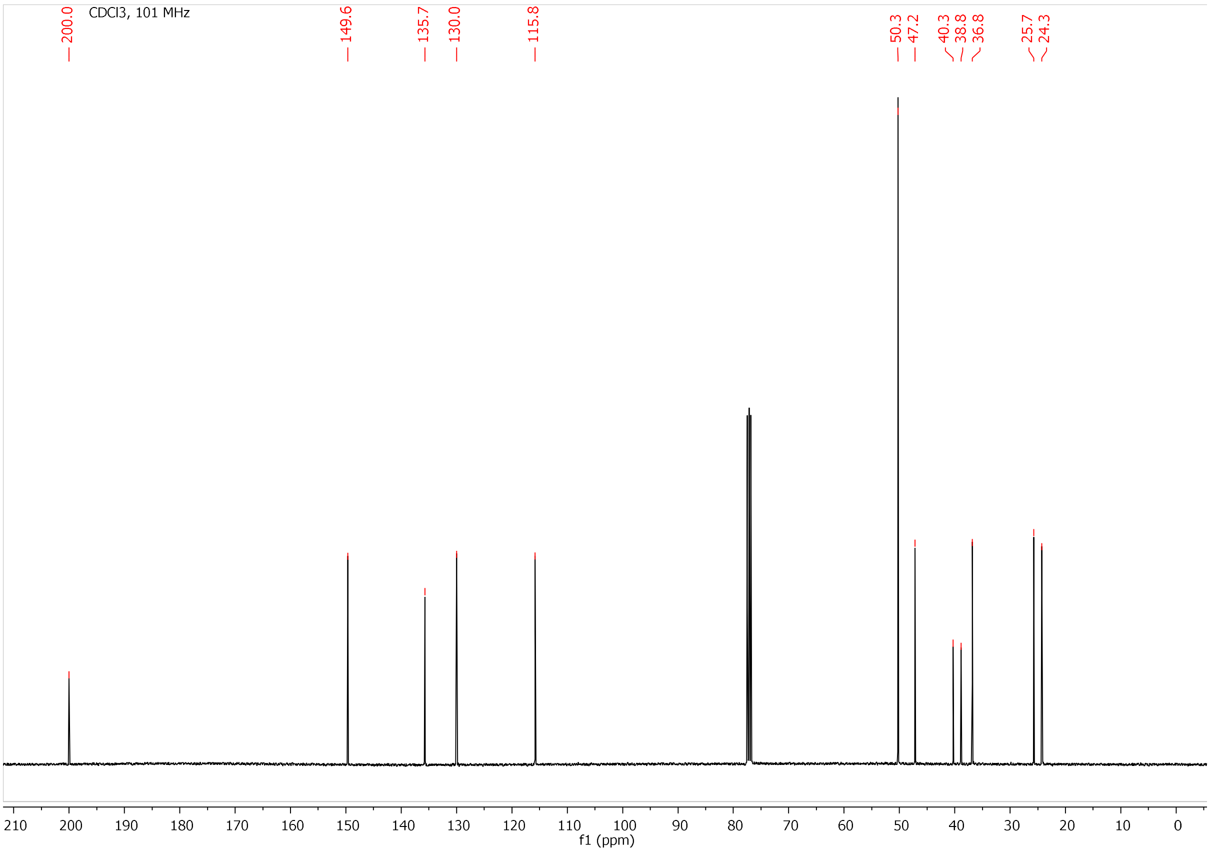

Ethyl 2-(3-allylbicyclo[1.1.1]pentan-1-yl)propanoate (15a)

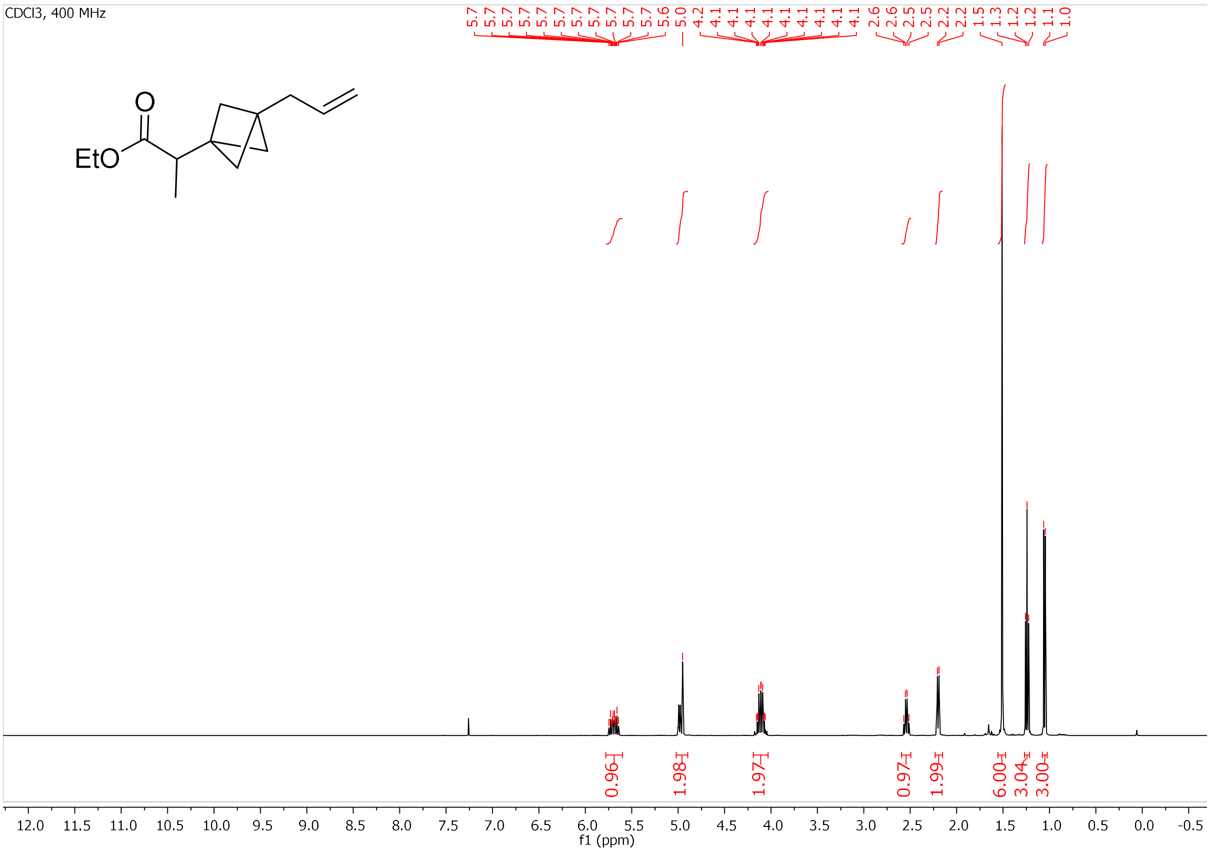

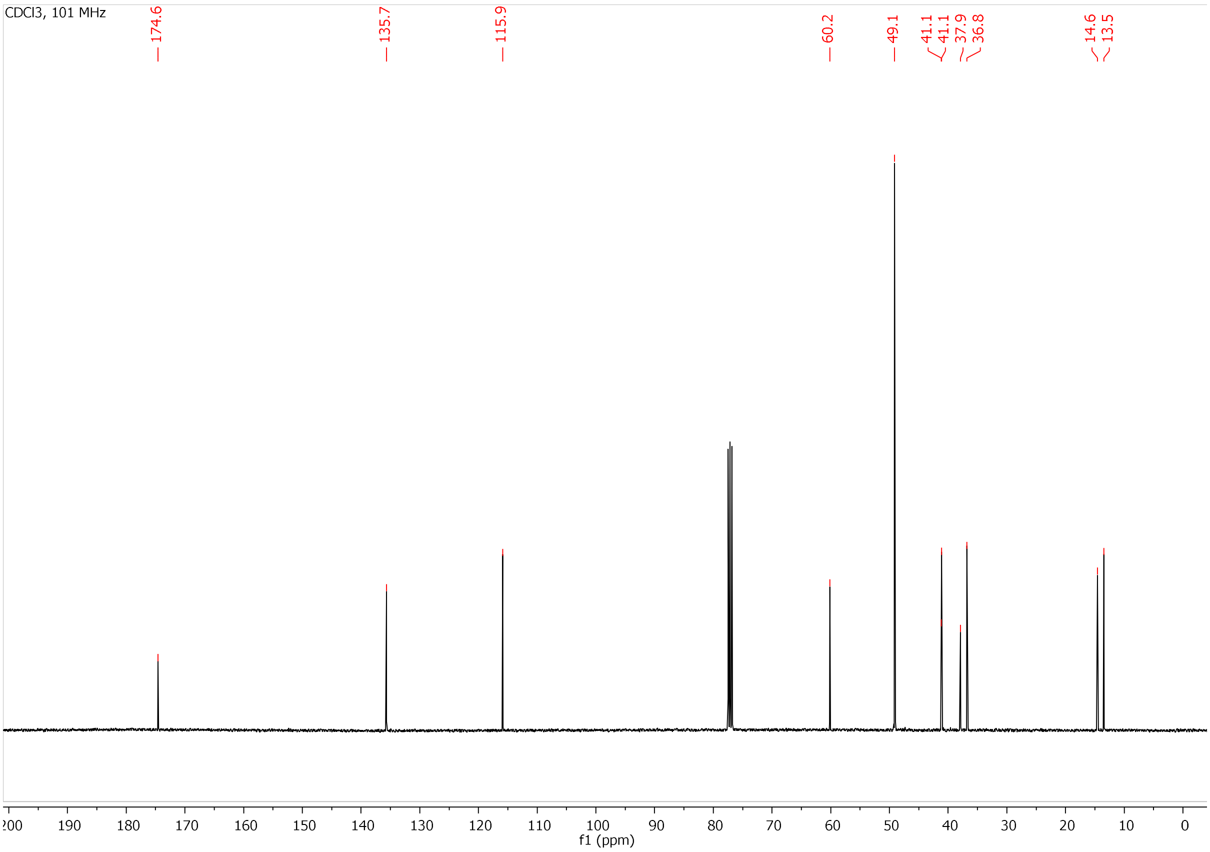

Ethyl 2-(3-allylbicyclo[1.1.1]pentan-1-yl)hept-6-enoate (15b)

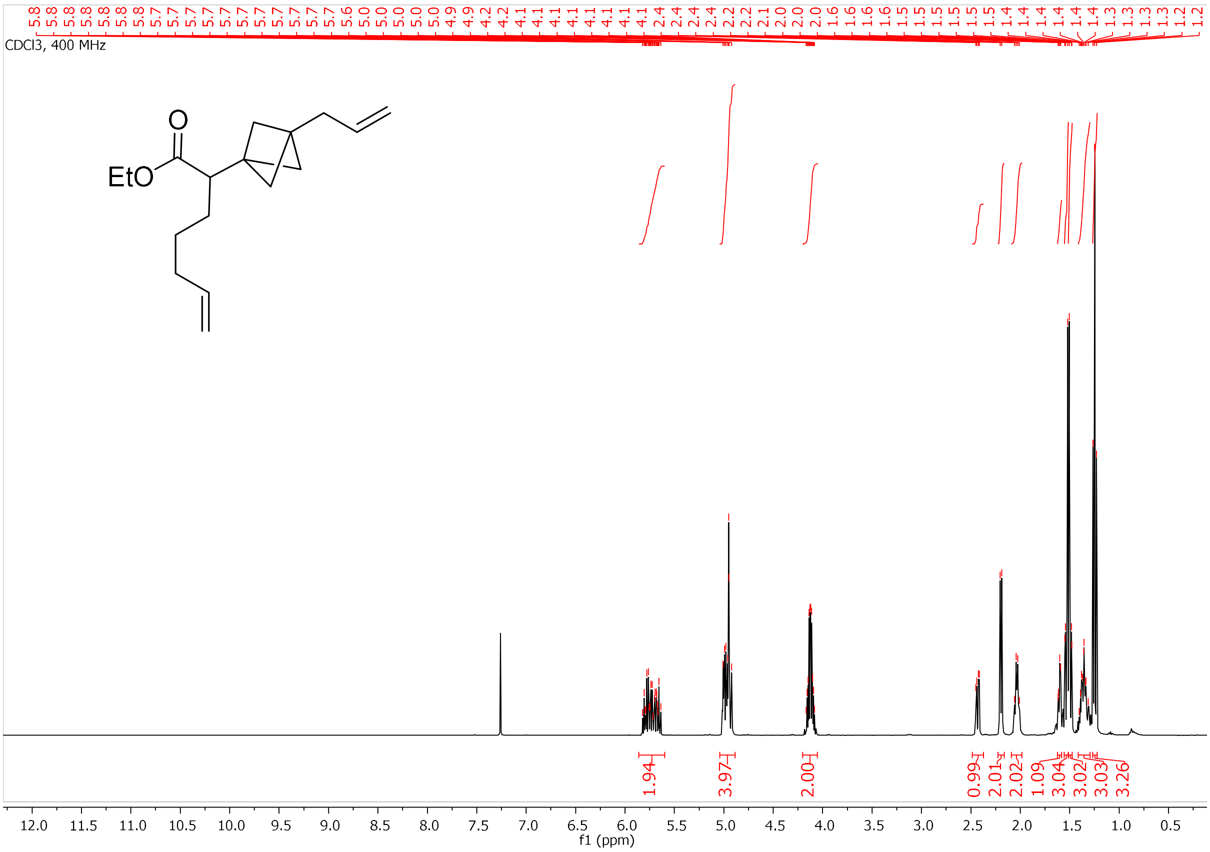

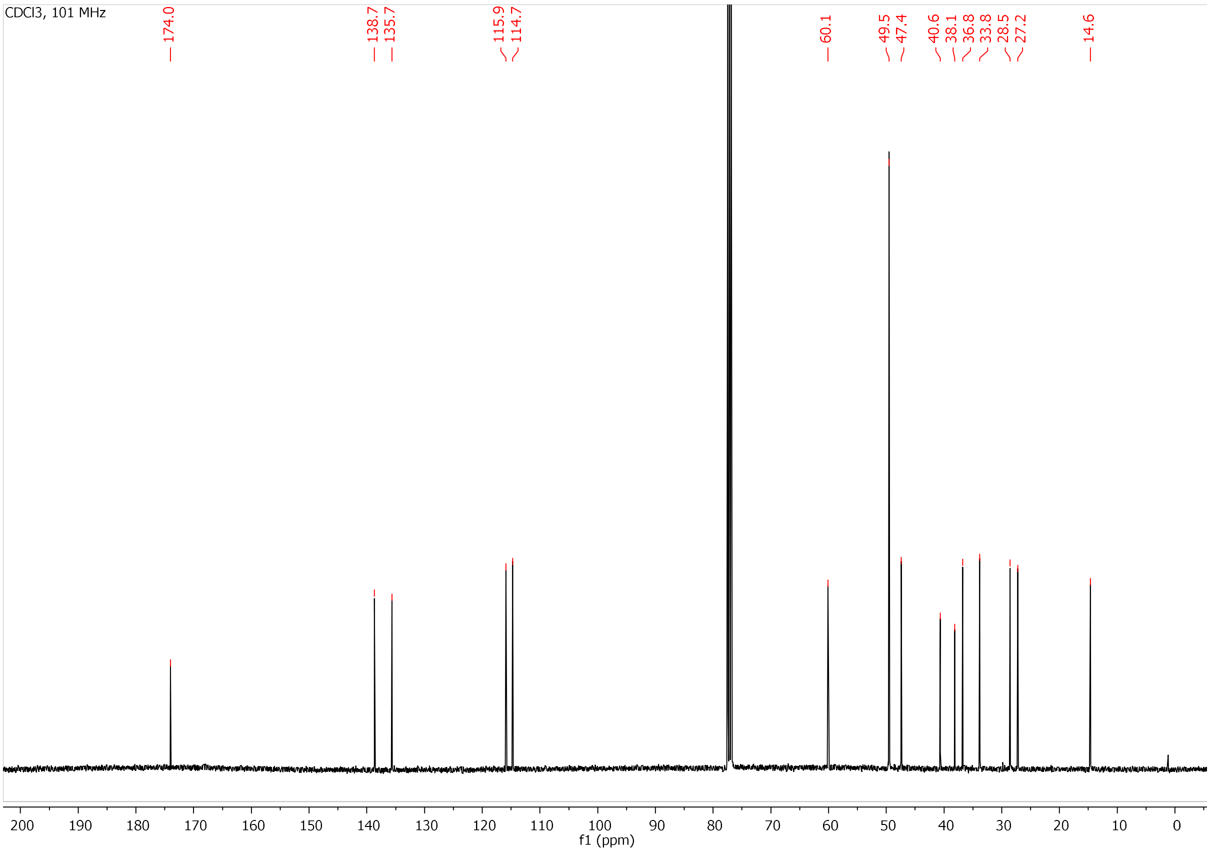

Ethyl 2-(3-allylbicyclo[1.1.1]pentan-1-yl)-2-(4-bromophenyl)acetate (15c)

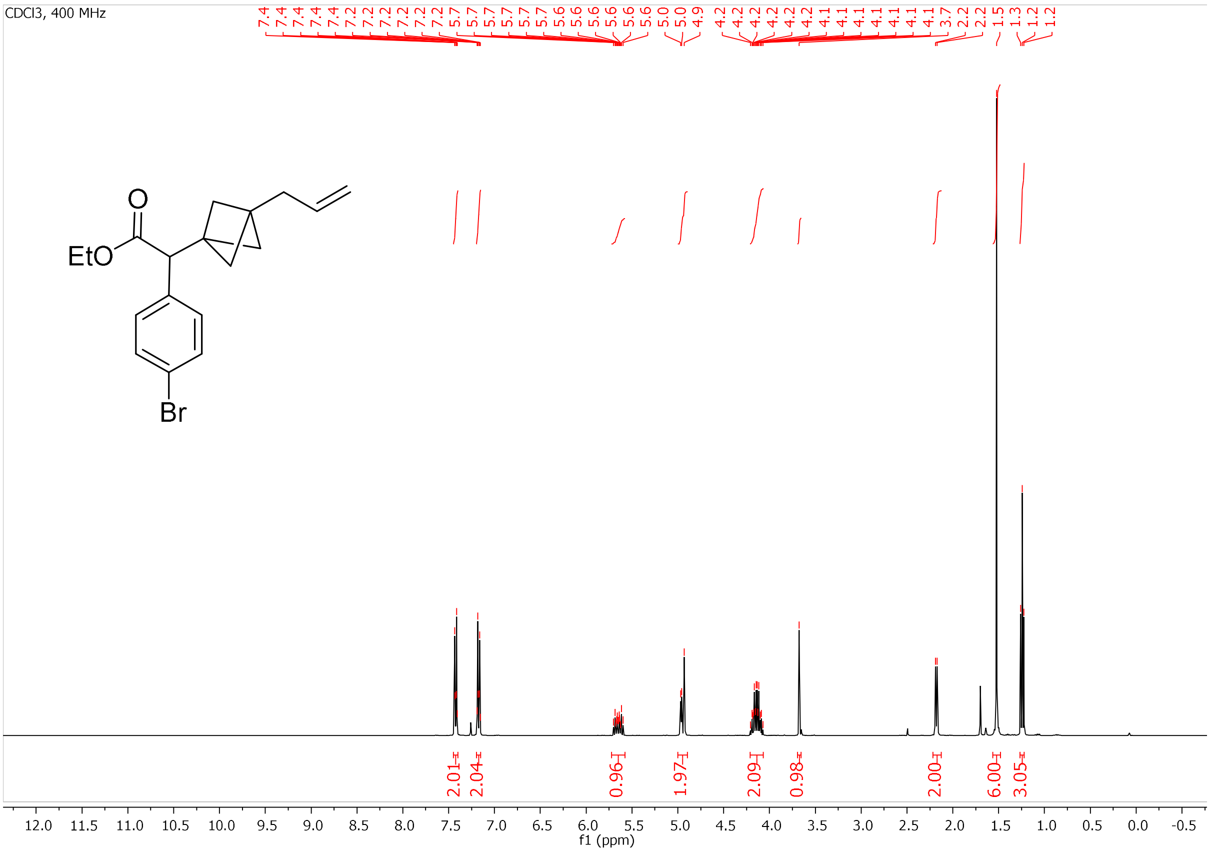

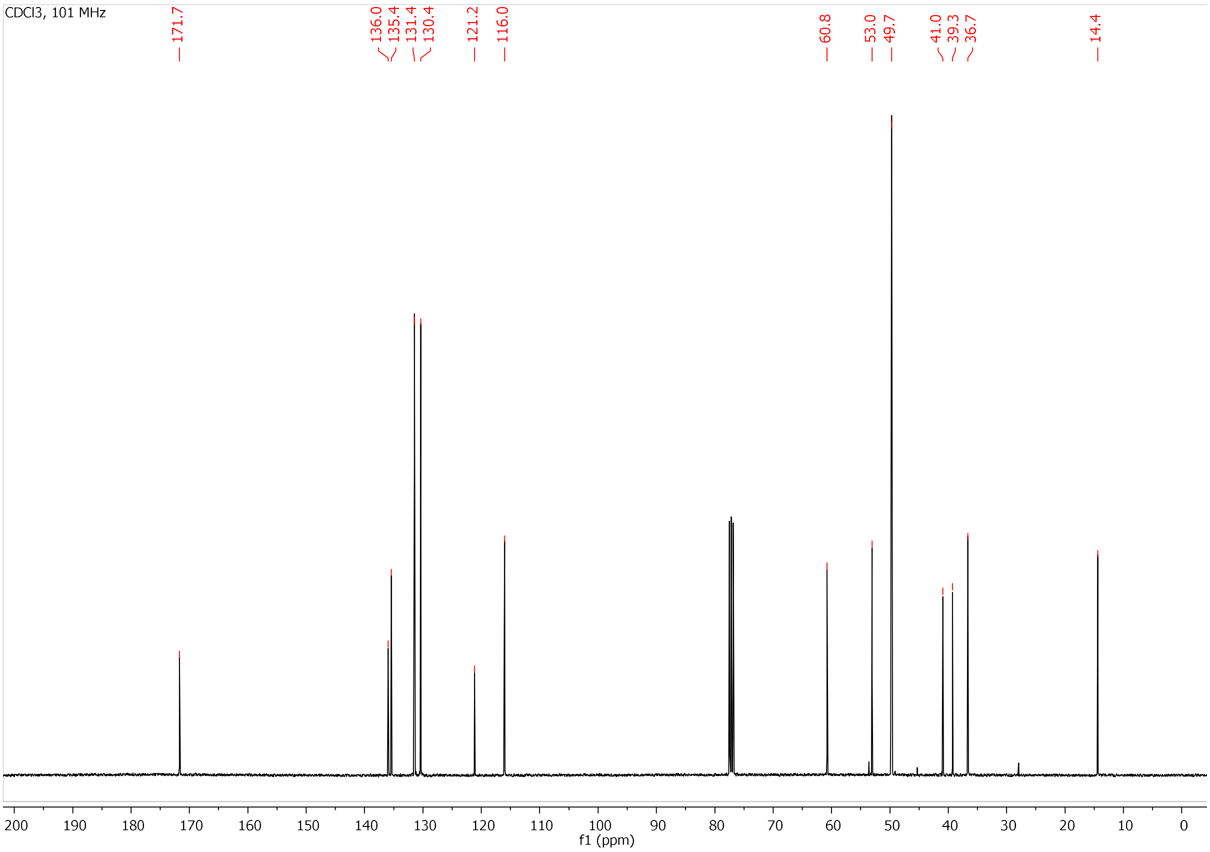

1-(3-Allylbicyclo[1.1.1]pentan-1-yl)cyclohexane-1-carbonitrile (22a)

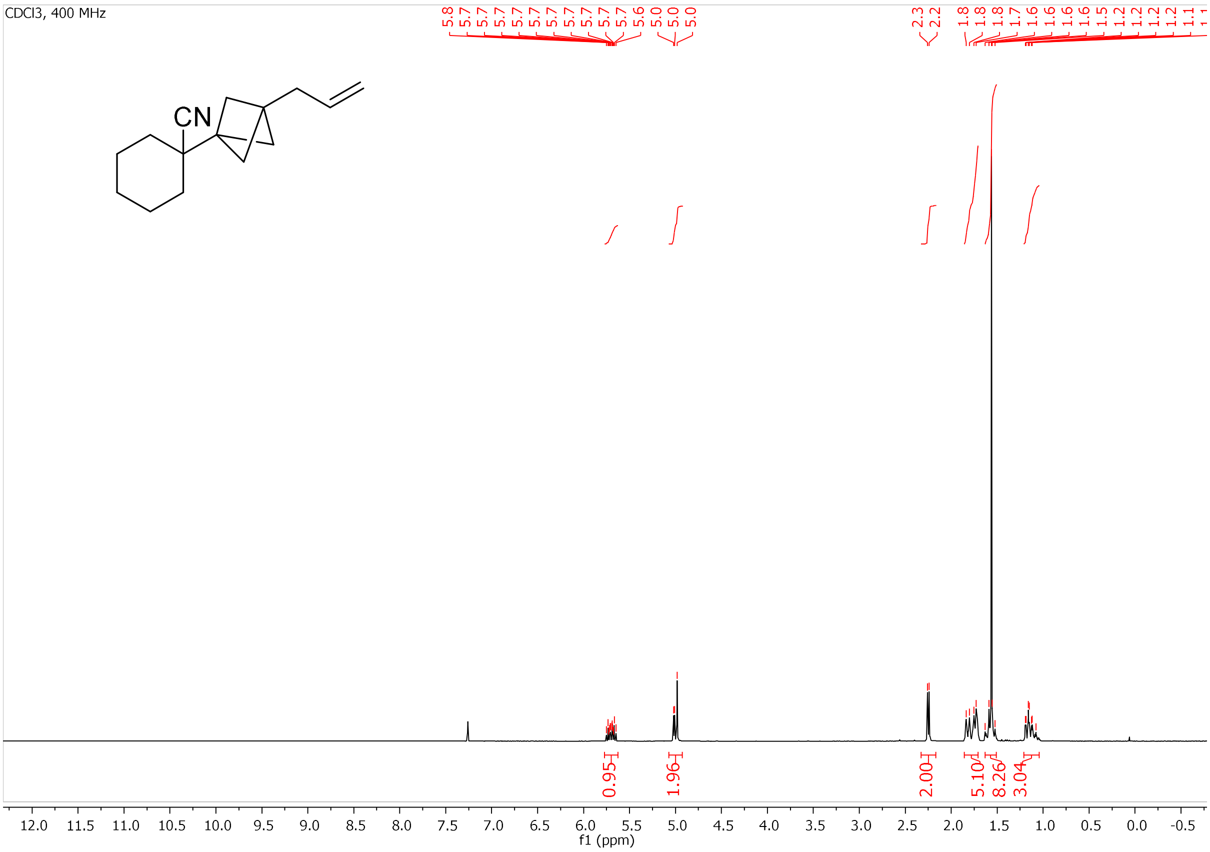

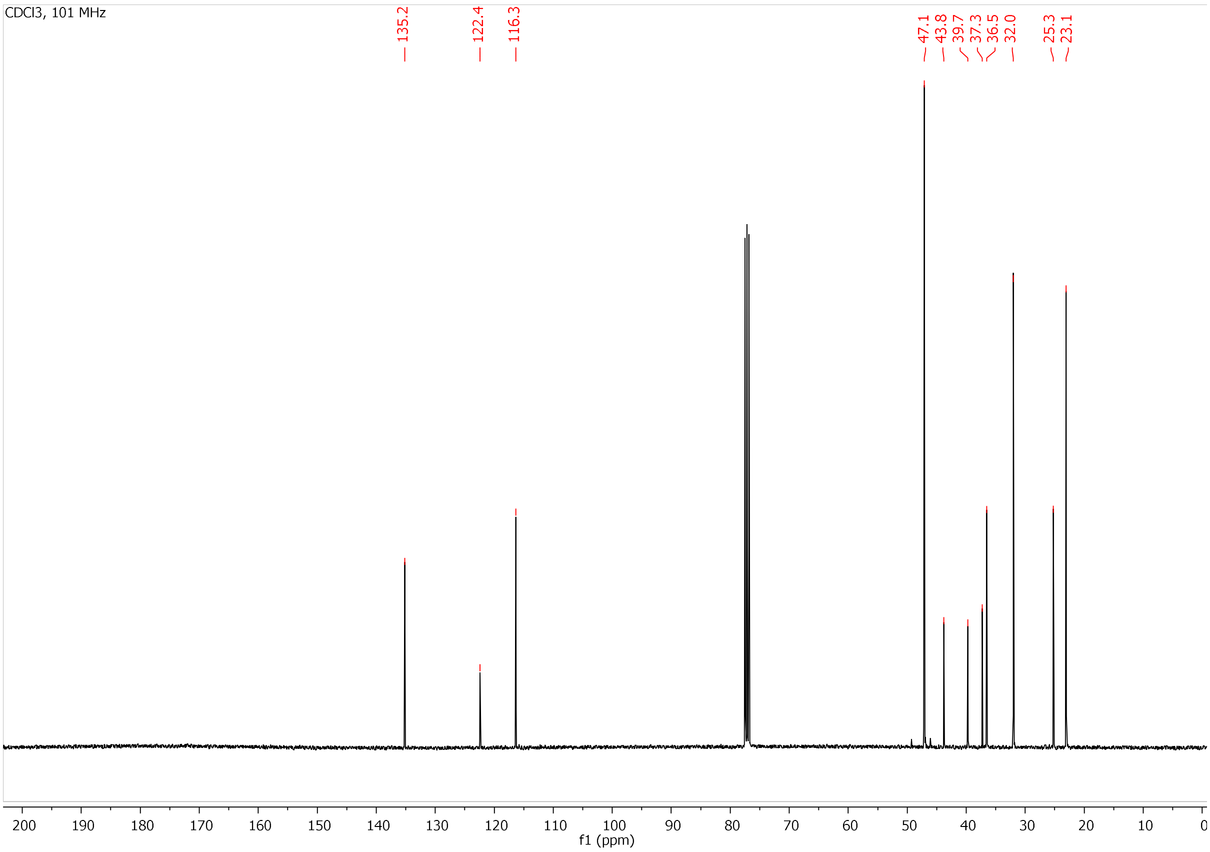

2-(3-Allylbicyclo[1.1.1]pentan-1-yl)-2-phenylpropanenitrile (22b)

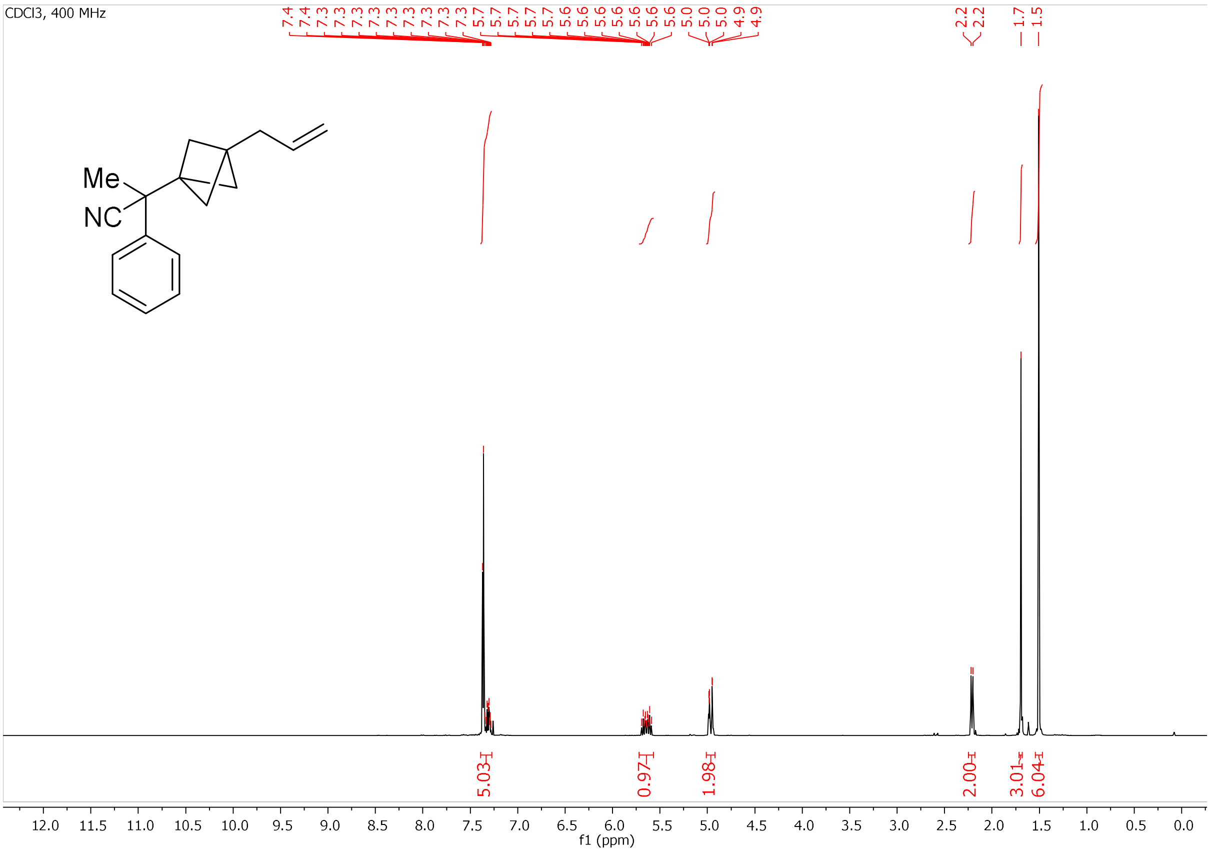

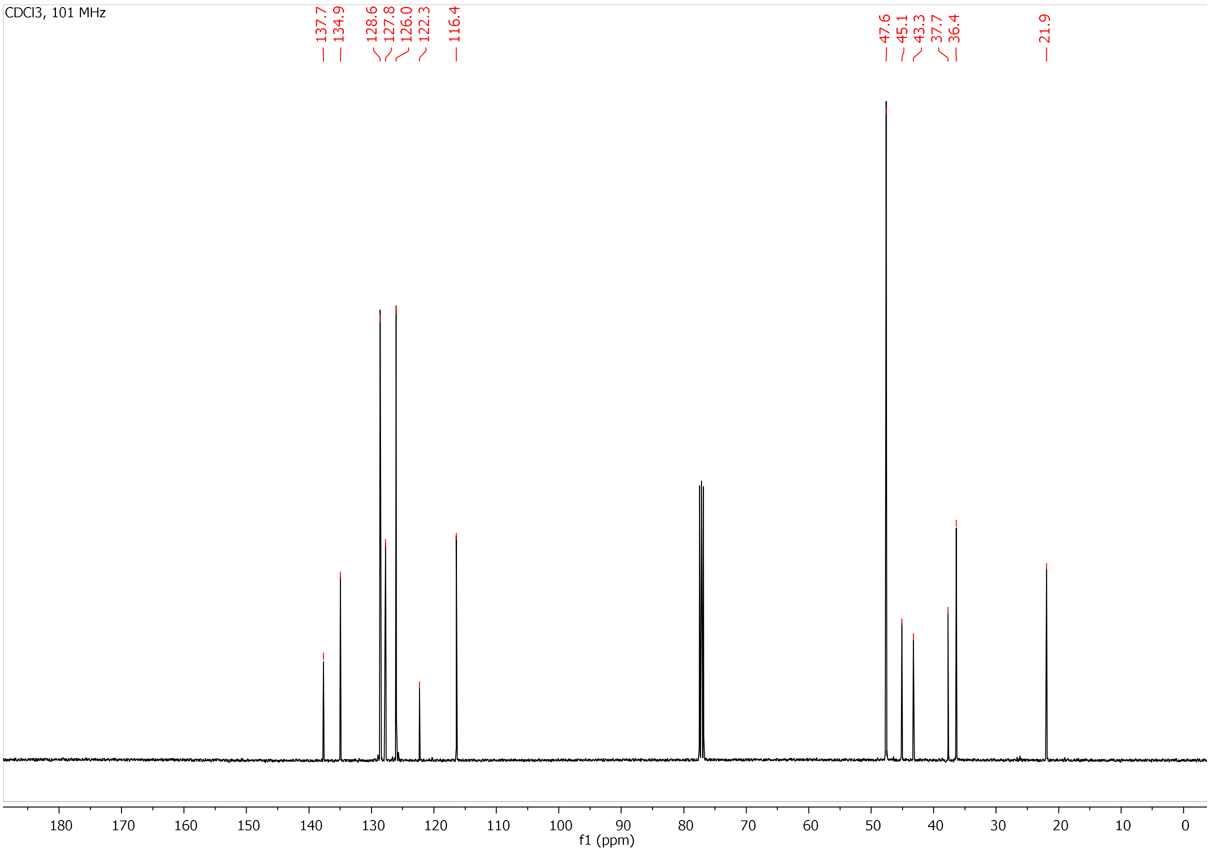

1-(3-Allylbicyclo[1.1.1]pentan-1-yl)cyclohex-2-ene-1-carbonitrile (22c)

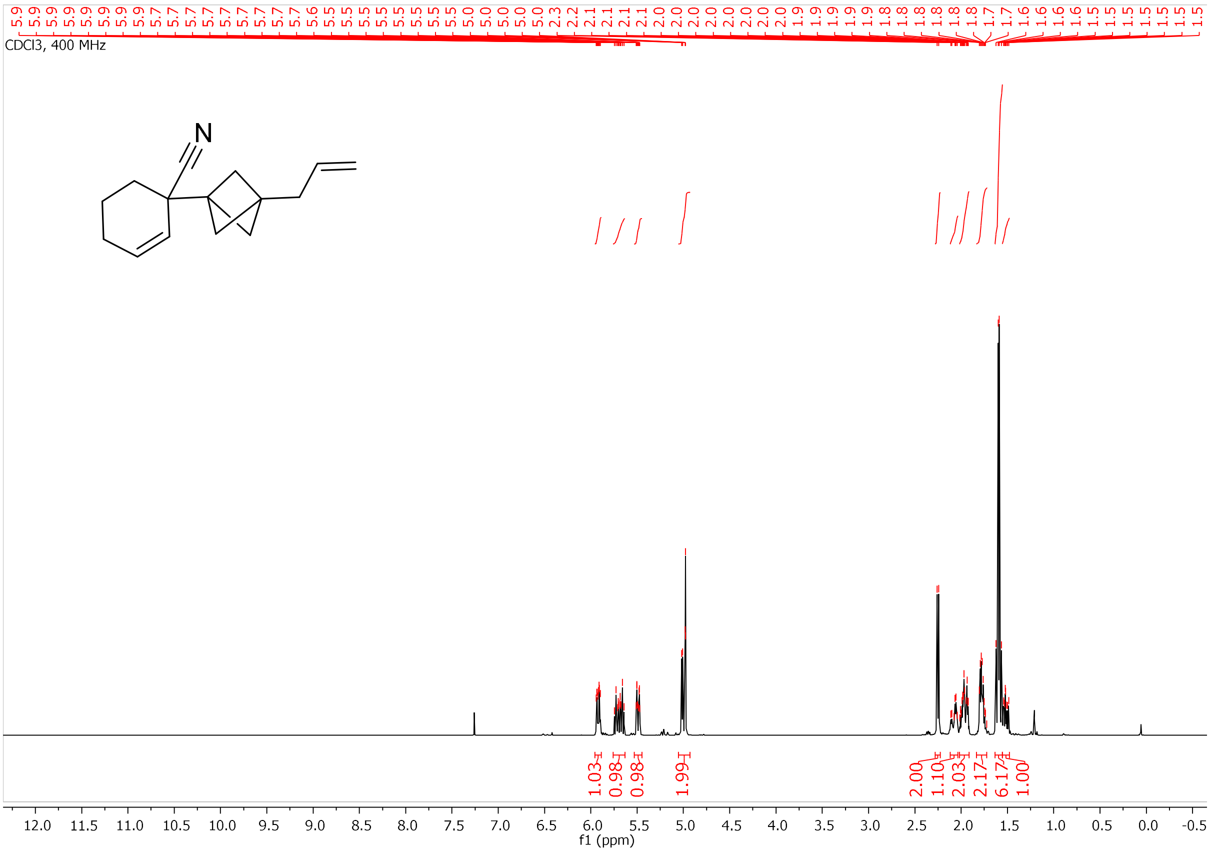

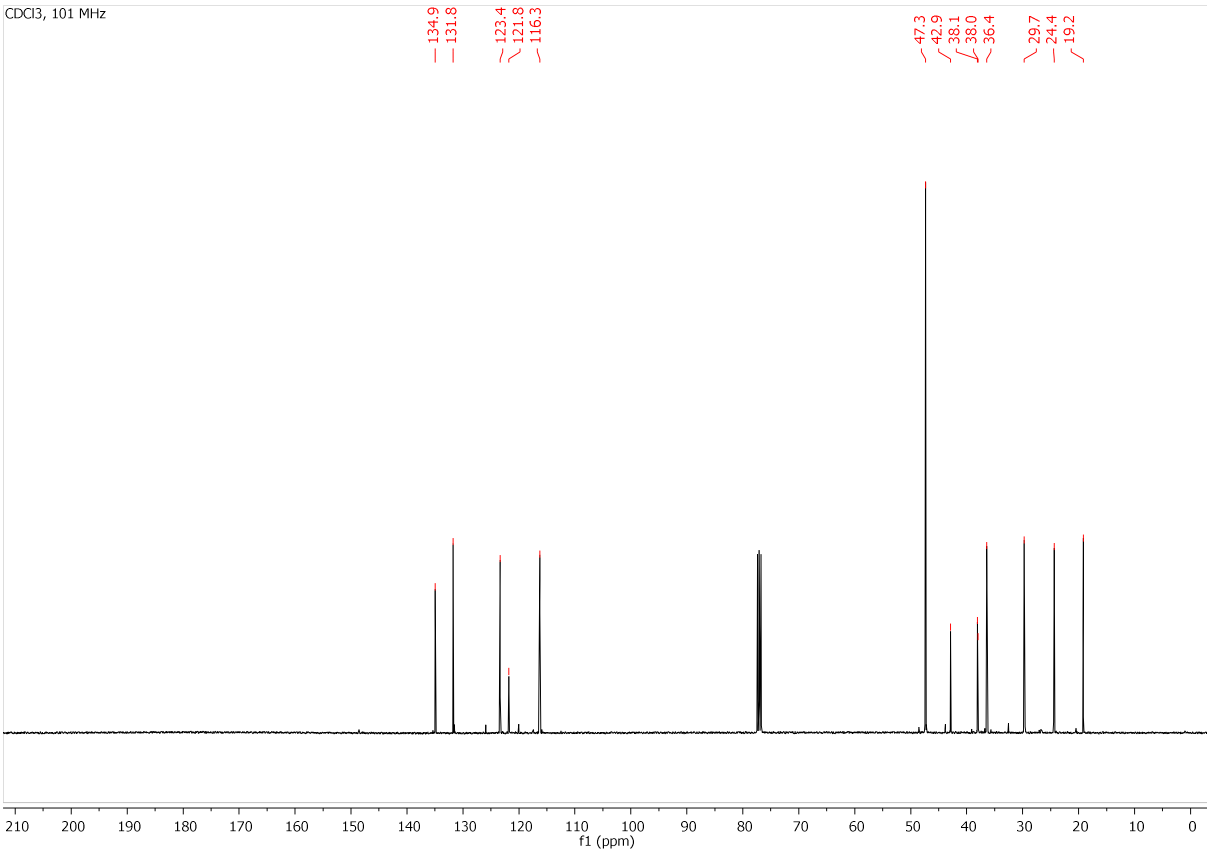

Ethyl 4-(bicyclo[1.1.1]pentan-1-yl)-1-methylpiperidine-4-carboxylate (6)

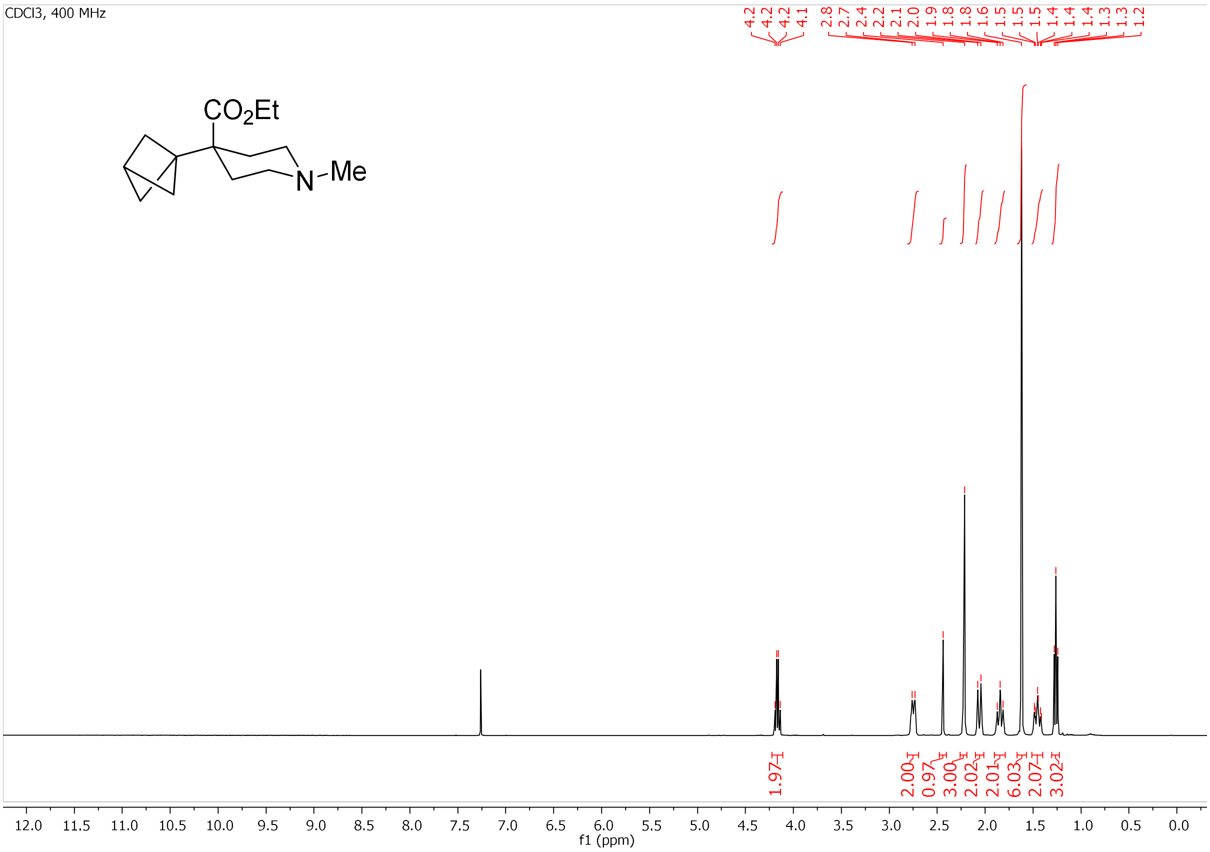

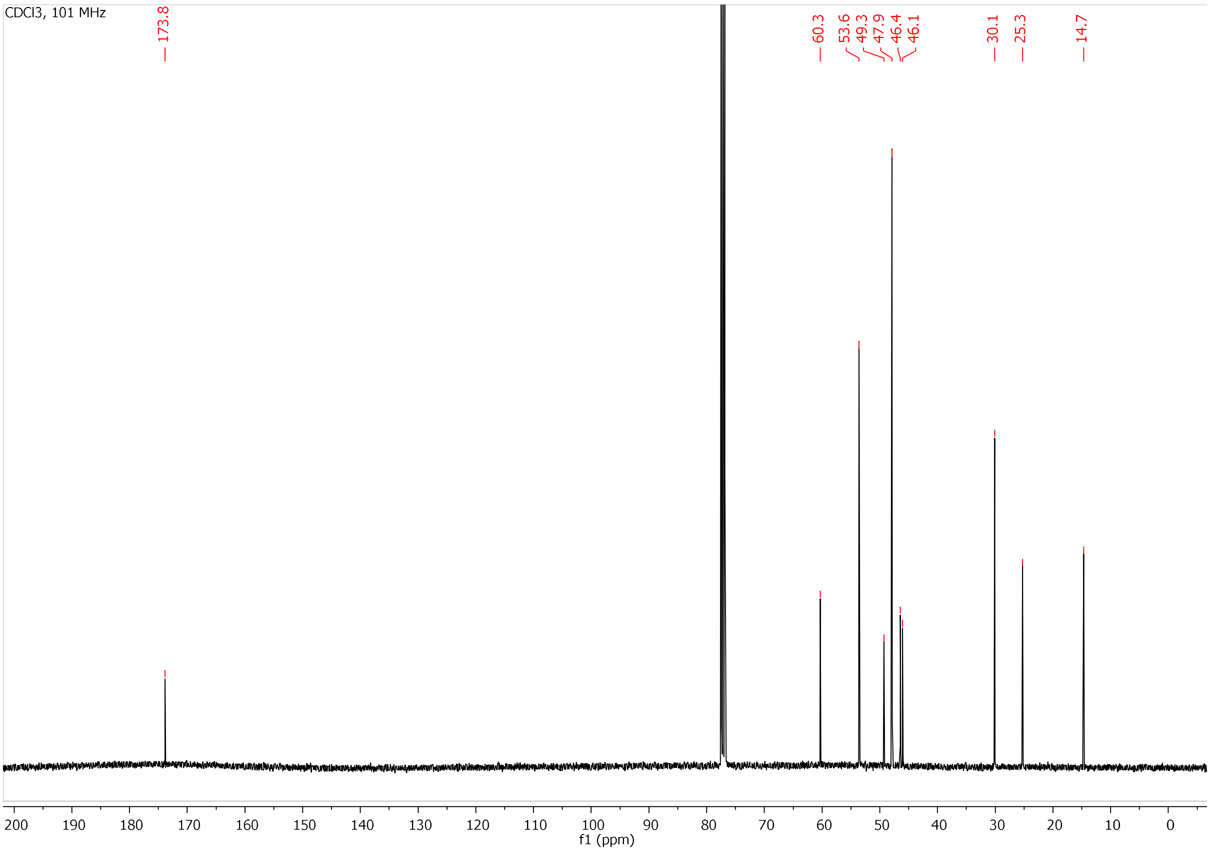

Details for X-ray data collection and structure refinement

Phenyl(3-(1-phenylallyl)bicyclo[1.1.1]pentan-1-yl)methanone (9b)

Table S3. Details for X-ray data collection and structure refinement for compound **9b**.

	**9b**
Empirical formula	$C_{21}H_{20}O$
Formula mass	288.37
T[K]	143(2)
Crystal size [mm]	0.40 × 0.10 × 0.10
Crystal description	colorless rod
Crystal system	monoclinic
Space group	*P*21/*n*
a [Å]	10.7427(6)
b [Å]	10.4700(4)
c [Å]	14.4147(7)
α [°]	90.0
β [°]	93.099(5)
γ [°]	90.0
V [$Å^3$]	1618.94(14)
Z	4
$\rho_{calcd.}$ [g cm^{-3}]	1.183
μ [mm^{-1}]	0.071
F(000)	616
Θ range [°]	4.27 – 25.24
Index ranges	$-13 \leq h \leq 12$
	$-12 \leq k \leq 10$
	$-16 \leq l \leq 17$
Reflns. collected	11265
Reflns. obsd.	2144
Reflns. unique	3050 (R_{int} = 0.0438)
R_1, wR_2 (2σ data)	0.0725, 0.1831
R_1, wR_2 (all data)	0.1006, 0.2079
GOOF on F^2	1.030
Peak/hole [e $Å^{-3}$]	0.776 / -0.251

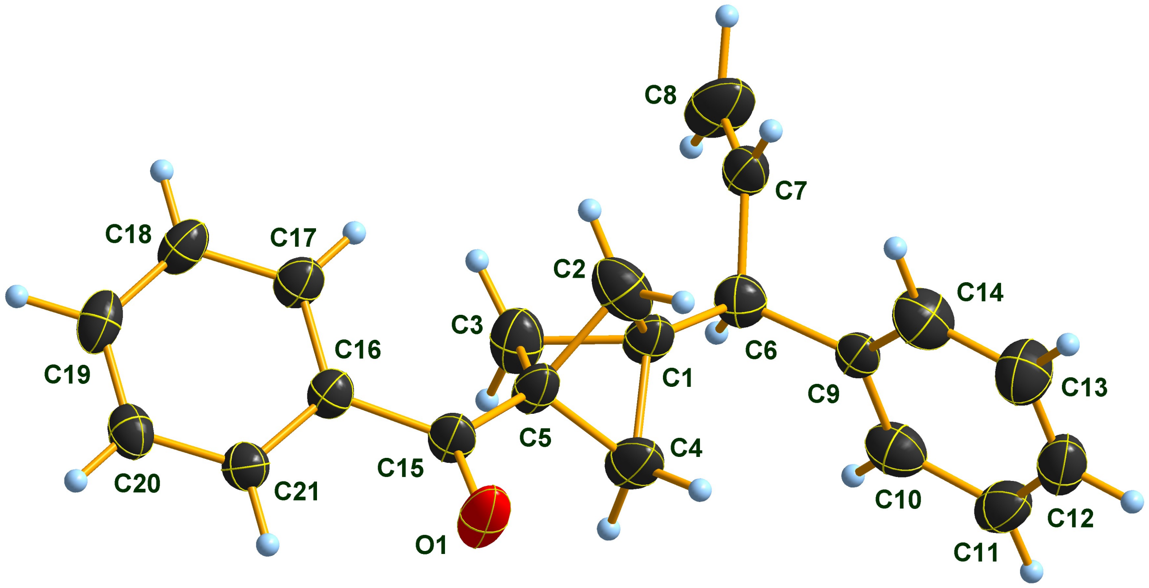

Figure S4. Molecular structure of compound **9b** in the crystal. DIAMOND[22] representation; thermal ellipsoids are drawn at 50 % probability level.

Table S4. Selected bond lengths (Å) of compound **9b**.

C9 – C14	1.364(4)	C5 – C2	1.527(4)
C9 – C10	1.387(4)	C5 – C4	1.553(4)
C9 – C6	1.531(3)	C5 – C3	1.573(4)
O1 – C15	1.212(3)	C11 – C12	1.361(5)
C21 – C20	1.382(4)	C11 – C10	1.444(4)
C21 – C16	1.393(3)	C12 – C13	1.349(5)
C16 – C17	1.399(4)	C13 – C14	1.355(5)
C16 – C15	1.487(3)	C1 – C2	1.558(4)
C15 – C5	1.498(3)	C1 – C5	1.877(4)
C17 – C18	1.395(4)	C20 – C19	1.384(4)
C1 – C6	1.517(4)	C19 – C18	1.387(4)
C1 – C3	1.533(4)	C7 – C8	1.282(4)
C1 – C4	1.555(4)	C7 – C6	1.493(4)

Table S5. Selected bond angles (°) of compound **9b**.

C14 – C9 – C10	118.8(3)	C4 – C5 – C3	85.5(2)
C14 – C9 – C6	123.5(3)	C15 – C5 – C1	174.9(2)
C10 – C9 – C6	117.7(2)	C2 – C5 – C1	53.3(2)

[22] DIAMOND, Crystal Impact GbR, Version 3.2i.

C20 – C21 – C16	121.1(2)	C4 – C5 – C1	52.9(2)
C21 – C16 – C17	119.1(2)	C3 – C5 – C1	51.9(2)
C21 – C16 – C15	118.3(2)	C19 – C18 – C17	120.2(3)
C17 – C16 – C15	122.6(2)	C12 – C11 – C10	117.4(3)
O1 – C15 – C16	120.7(2)	C9 – C10 – C11	119.4(3)
O1 – C15 – C5	118.6(2)	C13 – C12 – C11	122.5(3)
C16 – C15 – C5	120.6(2)	C5 – C4 – C1	74.3(2)
C18 – C17 – C16	119.7(2)	C1 – C3 – C5	74.4(2)
C6 – C1 – C3	126.6(2)	C12 – C13 – C14	119.8(3)
C6 – C1 – C4	126.3(2)	C13 – C14 – C9	122.1(3)
C3 – C1 – C4	86.8(2)	C5 – C2 – C1	74.9(2)
C6 – C1 – C2	128.8(2)	C8 – C7 – C6	127.1(3)
C3 – C1 – C2	87.2(2)	C7 – C6 – C1	110.7(2)
C4 – C1 – C2	87.6(2)	C7 – C6 – C9	112.6(2)
C6 – C1 – C5	179.1(2)	C1 – C6 – C9	109.9(2)
C3 – C1 – C5	53.8(2)	C15 – C5 – C2	128.2(2)
C4 – C1 – C5	52.8(2)	C15 – C5 – C4	122.2(2)
C2 – C1 – C5	51.8(2)	C2 – C5 – C4	88.8(2)
C21 – C20 – C19	119.7(3)	C15 – C5 – C3	131.4(2)
C20 – C19 – C18	120.3(2)	C2 – C5 – C3	86.9(2)

Table S6. Selected torsion angles (°) of compound **9b**.

C20 – C21 – C16 – C17	1.2(4)	C2 – C1 – C5 – C4	-122.1(3)
C20 – C21 – C16 – C15	-178.5(2)	C4 – C1 – C5 – C3	-117.9(3)
C21 – C16 – C15 – O1	-21.7(4)	C2 – C1 – C5 – C3	120.0(3)
C17 – C16 – C15 – O1	158.6(3)	C20 – C19 – C18 – C17	1.5(4)
C21 – C16 – C15 – C5	155.9(2)	C16 – C17 – C18 – C19	-1.2(4)
C17 – C16 – C15 – C5	-23.8(4)	C14 – C9 – C10 – C11	0.5(4)
C21 – C16 – C17 – C18	-0.1(4)	C6 – C9 – C10 – C11	-176.8(2)
C15 – C16 – C17 – C18	179.6(2)	C12 – C11 – C10 – C9	0.0(4)
C16 – C21 – C20 – C19	-1.0(4)	C10 – C11 – C12 – C13	-0.7(5)
C21 – C20 – C19 – C18	-0.3(4)	C15 – C5 – C4 – C1	-178.6(3)
C8 – C7 – C6 – C1	-114.1(3)	C2 – C5 – C4 – C1	-42.8(2)
C8 – C7 – C6 – C9	122.4(3)	C3 – C5 – C4 – C1	44.2(2)
C3 – C1 – C6 – C7	77.1(4)	C6 – C1 – C4 – C5	-179.9(3)
C4 – C1 – C6 – C7	-165.7(3)	C3 – C1 – C4 – C5	-45.6(2)
C2 – C1 – C6 – C7	-44.1(4)	C2 – C1 – C4 – C5	41.8(2)
C3 – C1 – C6 – C9	-157.9(3)	C6 – C1 – C3 – C5	178.9(3)
C4 – C1 – C6 – C9	-40.6(4)	C4 – C1 – C3 – C5	44.8(2)

C2 – C1 – C6 – C9	80.9(3)	C2 – C1 – C3 – C5	-42.9(2)
C14 – C9 – C6 – C7	50.3(3)	C15 – C5 – C3 – C1	-174.9(3)
C10 – C9 – C6 – C7	-132.4(3)	C2 – C5 – C3 – C1	44.0(2)
C14 – C9 – C6 – C1	-73.6(3)	C4 – C5 – C3 – C1	-45.0(2)
C10 – C9 – C6 – C1	103.6(3)	C11 – C12 – C13 – C14	0.8(5)
O1 – C15 – C5 – C2	-88.8(4)	C12 – C13 – C14 – C9	-0.2(5)
C16 – C15 – C5 – C2	93.5(3)	C10 – C9 – C14 – C13	-0.5(4)
O1 – C15 – C5 – C4	28.8(4)	C6 – C9 – C14 – C13	176.8(3)
C16 – C15 – C5 – C4	-148.9(3)	C15 – C5 – C2 – C1	173.9(3)
O1 – C15 – C5 – C3	144.2(3)	C4 – C5 – C2 – C1	42.5(2)
C16 – C15 – C5 – C3	-33.5(4)	C3 – C5 – C2 – C1	-43.0(2)
C3 – C1 – C5 – C2	-120.0(3)	C6 – C1 – C2 – C5	-179.0(3)
C4 – C1 – C5 – C2	122.1(3)	C3 – C1 – C2 – C5	44.4(2)
C3 – C1 – C5 – C4	117.9(3)	C4 – C1 – C2 – C5	-42.5(2)

CCDC-2011116 contains supplementary crystallographic data for this compound. These data can be obtained free of charge from The Cambridge Crystallographic Data Centre *via* www.ccdc.cam.ac.uk/data_request/cif.

(1*R*,3*R*,5*R*)-3-(3-(4-Methoxyphenyl)bicyclo[1.1.1]pentan-1-yl)-6,6-dimethyl-2-methylene-bicyclo[3.1.1]heptane (9m)

Table S7. Details for X-ray data collection and structure refinement for compound **9m**.

	**9m**
Empirical formula	$C_{22}H_{28}O$
Formula mass	308.44
T[K]	143(2)
Crystal size [mm]	0.40 × 0.15 × 0.02
Crystal description	colorless platelet
Crystal system	monoclinic
Space group	*P*21
a [Å]	8.0515(6)
b [Å]	6.4231(5)
c [Å]	17.0787(16)
α [°]	90.0

β [°]	92.434(8)
γ [°]	90.0
V [Å^3]	882.44(13)
Z	2
$\rho_{calcd.}$ [g cm^{-3}]	1.161
μ [mm^{-1}]	0.069
F(000)	336
Θ range [°]	4.20 – 25.24
Index ranges	-8 ≤ *h* ≤ 10
	-7 ≤ *k* ≤ 8
	-21 ≤ *l* ≤ 21
Reflns. collected	5879
Reflns. obsd.	2613
Reflns. unique	3357 (R_{int} = 0.0380)
R_1, wR_2 (2σ data)	0.0530, 0.0876
R_1, wR_2 (all data)	0.0788, 0.0995
GOOF on F^2	1.054
Peak/hole [e Å^{-3}]	0.215 / -0.178

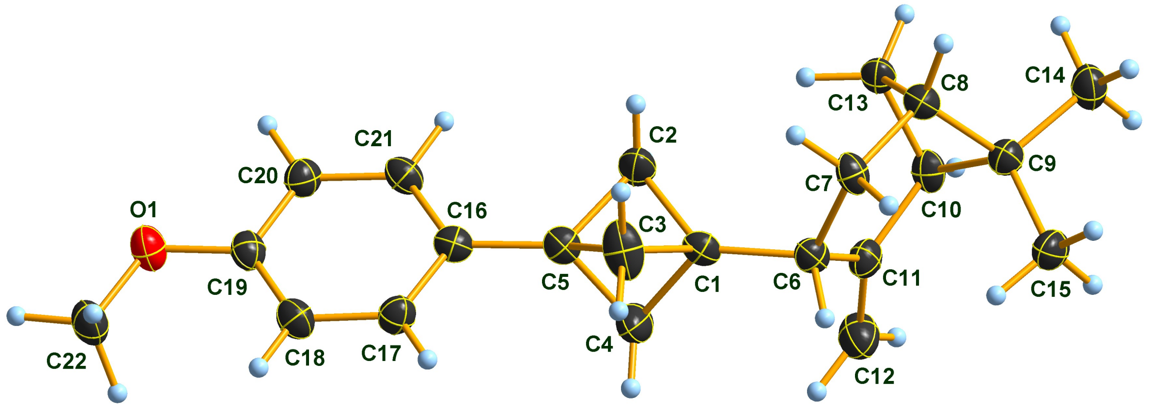

Figure S5. Molecular structure of compound **9m** in the crystal. DIAMOND representation; thermal ellipsoids are drawn at 50 % probability level.

Table S8. Selected bond lengths (Å) of compound **9m**.

O1 – C19	1.368(3)	C16 – C21	1.387(4)
O1 – C22	1.429(4)	C16 – C17	1.396(4)
C13 – C8	1.543(4)	C11 – C12	1.327(5)
C13 – C10	1.548(4)	C15 – C9	1.521(4)
C10 – C11	1.492(4)	C18 – C19	1.379(5)
C10 – C9	1.567(4)	C18 – C17	1.385(4)
C6 – C1	1.511(4)	C9 – C14	1.530(5)
C6 – C11	1.533(4)	C1 – C5	1.900(4)
C6 – C7	1.558(4)	C5 – C16	1.480(4)
C8 – C7	1.519(4)	C5 – C3	1.548(4)
C8 – C9	1.558(4)	C5 – C2	1.551(5)
C1 – C4	1.548(4)	C5 – C4	1.552(4)
C1 – C2	1.550(4)	C20 – C21	1.379(4)
C1 – C3	1.558(5)	C20 – C19	1.394(5)

Table S9. Selected bond angles (°) of compound **9m**.

C19 – O1 – C22	117.6(3)	C12 – C11 – C10	122.7(3)
C8 – C13 – C10	86.6(2)	C12 – C11 – C6	121.9(3)
C11 – C10 – C13	109.9(3)	C10 – C11 – C6	115.3(3)
C11 – C10 – C9	109.4(3)	C20 – C21 – C16	121.6(3)
C13 – C10 – C9	87.1(2)	C19 – C18 – C17	119.6(3)
C1 – C6 – C11	111.4(2)	C15 – C9 – C14	109.0(3)
C1 – C6 – C7	112.2(3)	C15 – C9 – C8	119.6(3)
C11 – C6 – C7	112.7(3)	C14 – C9 – C8	111.8(3)
C7 – C8 – C13	108.6(3)	C15 – C9 – C10	117.6(3)
C7 – C8 – C9	111.5(3)	C14 – C9 – C10	111.8(3)
C13 – C8 – C9	87.6(2)	C8 – C9 – C10	85.4(2)
C6 – C1 – C4	126.7(3)	C1 – C4 – C5	75.6(2)
C6 – C1 – C2	130.8(3)	C18 – C17 – C16	121.7(3)
C4 – C1 – C2	86.2(2)	O1 – C19 – C18	124.6(3)
C6 – C1 – C3	125.9(3)	O1 – C19 – C20	115.6(3)
C4 – C1 – C3	86.5(3)	C18 – C19 – C20	119.7(3)
C2 – C1 – C3	86.2(2)	C5 – C3 – C1	75.4(2)
C6 – C1 – C5	176.9(3)	C2 – C5 – C4	86.1(3)
C4 – C1 – C5	52.3(2)	C16 – C5 – C1	179.4(3)
C2 – C1 – C5	52.2(2)	C3 – C5 – C1	52.5(2)
C3 – C1 – C5	52.1(2)	C2 – C5 – C1	52.2(2)

C8 – C7 – C6	113.3(3)	C4 – C5 – C1	52.1(2)
C16 – C5 – C3	127.7(3)	C21 – C20 – C19	119.8(3)
C16 – C5 – C2	128.2(3)	C21 – C16 – C17	117.5(3)
C3 – C5 – C2	86.6(2)	C21 – C16 – C5	122.7(3)
C16 – C5 – C4	127.3(3)	C17 – C16 – C5	119.7(3)
C3 – C5 – C4	86.7(3)	C1 – C2 – C5	75.6(2)

Table S10. Selected torsion angles (°) of compound **9m**.

C8 – C13 – C10 – C11	82.6(3)	C17 – C16 – C21 – C20	-0.8(5)
C8 – C13 – C10 – C9	-27.0(2)	C5 – C16 – C21 – C20	178.0(3)
C10 – C13 – C8 – C7	-84.7(3)	C7 – C8 – C9 – C15	-37.1(4)
C10 – C13 – C8 – C9	27.2(2)	C13 – C8 – C9 – C15	-146.2(3)
C11 – C6 – C1 – C4	59.5(4)	C7 – C8 – C9 – C14	-166.1(3)
C7 – C6 – C1 – C4	-173.0(3)	C13 – C8 – C9 – C14	84.8(3)
C11 – C6 – C1 – C2	-62.8(4)	C7 – C8 – C9 – C10	82.2(3)
C7 – C6 – C1 – C2	64.7(4)	C13 – C8 – C9 – C10	-26.9(2)
C11 – C6 – C1 – C3	176.0(3)	C11 – C10 – C9 – C15	37.9(4)
C7 – C6 – C1 – C3	-56.5(4)	C13 – C10 – C9 – C15	147.9(3)
C13 – C8 – C7 – C6	42.4(4)	C11 – C10 – C9 – C14	165.1(3)
C9 – C8 – C7 – C6	-52.5(3)	C13 – C10 – C9 – C14	-84.8(3)
C1 – C6 – C7 – C8	-115.6(3)	C11 – C10 – C9 – C8	-83.3(3)
C11 – C6 – C7 – C8	11.2(4)	C13 – C10 – C9 – C8	26.8(2)
C3 – C5 – C16 – C21	130.8(4)	C6 – C1 – C4 – C5	176.4(3)
C2 – C5 – C16 – C21	9.9(6)	C2 – C1 – C4 – C5	-43.5(2)
C4 – C5 – C16 – C21	-109.4(4)	C3 – C1 – C4 – C5	43.0(2)
C3 – C5 – C16 – C17	-50.5(5)	C16 – C5 – C4 – C1	-179.8(3)
C2 – C5 – C16 – C17	-171.3(3)	C3 – C5 – C4 – C1	-43.3(2)
C4 – C5 – C16 – C17	69.3(5)	C2 – C5 – C4 – C1	43.5(2)
C6 – C1 – C2 – C5	-179.2(4)	C19 – C18 – C17 – C16	-0.3(5)
C4 – C1 – C2 – C5	43.6(2)	C21 – C16 – C17 – C18	1.1(5)
C3 – C1 – C2 – C5	-43.2(2)	C5 – C16 – C17 – C18	-177.7(3)
C16 – C5 – C2 – C1	-179.4(4)	C22 – O1 – C19 – C18	-1.2(5)
C3 – C5 – C2 – C1	43.5(2)	C22 – O1 – C19 – C20	178.6(3)
C4 – C5 – C2 – C1	-43.4(2)	C17 – C18 – C19 – O1	178.9(3)
C13 – C10 – C11 – C12	145.0(3)	C17 – C18 – C19 – C20	-0.8(5)
C9 – C10 – C11 – C12	-120.9(3)	C21 – C20 – C19 – O1	-178.7(3)
C13 – C10 – C11 – C6	-37.9(4)	C21 – C20 – C19 – C18	1.1(5)
C9 – C10 – C11 – C6	56.2(3)	C16 – C5 – C3 – C1	179.2(4)
C1 – C6 – C11 – C12	-69.1(4)	C2 – C5 – C3 – C1	-43.3(2)

C7 – C6 – C11 – C12	163.7(3)	C4 – C5 – C3 – C1	43.0(2)
C1 – C6 – C11 – C10	113.8(3)	C6 – C1 – C3 – C5	-177.2(3)
C7 – C6 – C11 – C10	-13.5(4)	C4 – C1 – C3 – C5	-43.1(2)
C19 – C20 – C21 – C16	-0.3(5)	C2 – C1 – C3 – C5	43.3(2)

CCDC-2011113 contains supplementary crystallographic data for this compound. These data can be obtained free of charge from The Cambridge Crystallographic Data Centre *via* www.ccdc.cam.ac.uk/data_request/cif.

2-(3-(4-Methoxyphenyl)bicyclo[1.1.1]pentan-1-yl)cyclohexan-1-one (14c)

Table S11. Details for X-ray data collection and structure refinement for compound **14c**.

	**14c**
Empirical formula	$C_{18}H_{22}O_2$
Formula mass	270.35
T[K]	143(2)
Crystal size [mm]	0.40 × 0.35 × 0.25
Crystal description	colorless block
Crystal system	monoclinic
Space group	*P*21/*c*
a [Å]	12.8464(4)
b [Å]	7.7678(3)
c [Å]	15.0626(4)
α [°]	90.0
β [°]	99.686(3)
γ [°]	90.0
V [$Å^3$]	1481.64(8)
Z	4
$\rho_{calcd.}$ [g cm^{-3}]	1.212
μ [mm^{-1}]	0.077
F(000)	584
Θ range [°]	3.41 – 25.24
Index ranges	$-16 \leq h \leq 16$
	$-9 \leq k \leq 9$

	$-18 \leq l \leq 18$
Reflns. collected	20402
Reflns. obsd.	2429
Reflns. unique	2863 (R_{int} = 0.0292)
R_1, wR_2 (2σ data)	0.0767, 0.2031
R_1, wR_2 (all data)	0.0863, 0.2142
GOOF on F^2	1.027
Peak/hole [e Å^{-3}]	0.733 / -0.280

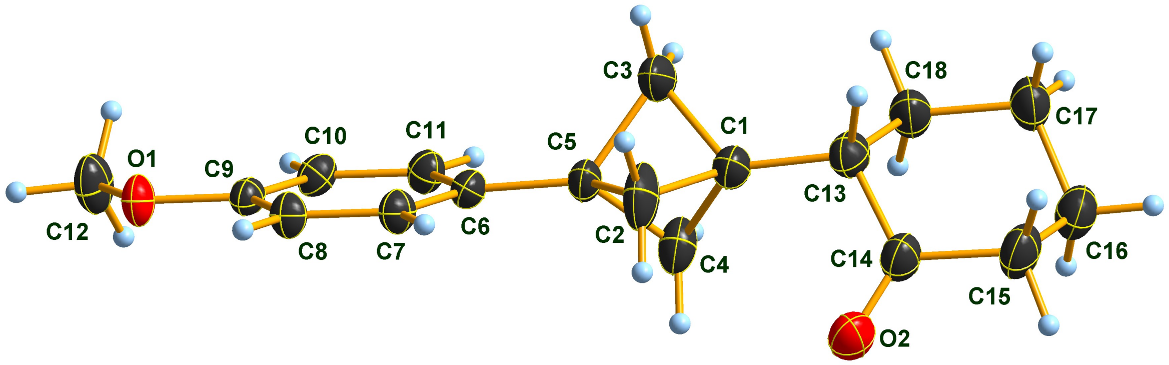

Figure S6. Molecular structure of compound **14c** in the crystal. DIAMOND representation; thermal ellipsoids are drawn at 50 % probability level.

Table S12. Selected bond lengths (Å) of compound **14c**.

O1 – C9	1.372(2)	C4 – C5	1.545(3)
O1 – C12	1.427(3)	C16 – C15	1.527(4)
C9 – C8	1.397(3)	C15 – C14	1.514(3)
C9 – C10	1.397(3)	C5 – C2	1.550(3)
C10 – C11	1.391(3)	C5 – C3	1.572(3)
C11 – C6	1.397(3)	C18 – C13	1.542(4)
C7 – C8	1.383(3)	C13 – C14	1.521(3)
C7 – C6	1.396(3)	C1 – C4	1.570(4)
C6 – C5	1.489(3)	C1 – C5	1.895(3)
C1 – C13	1.517(3)	O2 – C14	1.188(3)
C1 – C2	1.524(4)	C17 – C18	1.527(3)
C1 – C3	1.553(3)	C17 – C16	1.537(4)

Table S13. Selected bond angles (°) of compound **14c**.

C9 – O1 – C12	116.9(2)	C6 – C5 – C4	128.9(2)
O1 – C9 – C8	115.5(2)	C6 – C5 – C2	126.9(2)
O1 – C9 – C10	124.8(2)	C4 – C5 – C2	86.4(2)
C8 – C9 – C10	119.7(2)	C6 – C5 – C3	127.4(2)
C11 – C10 – C9	119.2(2)	C4 – C5 – C3	87.4(2)
C10 – C11 – C6	121.9(2)	C2 – C5 – C3	85.4(2)
C8 – C7 – C6	121.4(2)	C6 – C5 – C1	177.7(2)
C7 – C6 – C11	117.8(2)	C4 – C5 – C1	53.1(1)
C7 – C6 – C5	120.3(2)	C2 – C5 – C1	51.3(1)
C11 – C6 – C5	121.9(2)	C3 – C5 – C1	52.2(1)
C13 – C1 – C2	128.7(2)	C17 – C18 – C13	112.5(2)
C13 – C1 – C3	125.5(2)	C1 – C13 – C14	112.9(2)
C2 – C1 – C3	87.0(2)	C1 – C13 – C18	110.9(2)
C13 – C1 – C4	128.2(2)	C14 – C13 – C18	109.8(2)
C2 – C1 – C4	86.4(2)	O2 – C14 – C15	121.6(2)
C3 – C1 – C4	87.2(2)	O2 – C14 – C13	123.3(2)
C13 – C1 – C5	178.5(2)	C15 – C14 – C13	115.0(2)
C2 – C1 – C5	52.6(1)	C1 – C3 – C5	74.7(2)
C3 – C1 – C5	53.1(1)	C1 – C2 – C5	76.1(2)
C4 – C1 – C5	51.9(1)	C5 – C4 – C1	74.9(2)
C18 – C17 – C16	110.1(2)	C15 – C16 – C17	110.0(2)
C7 – C8 – C9	120.1(2)	C14 – C15 – C16	110.9(2)

Table S14. Selected torsion angles (°) of compound **14c**.

C12 – O1 – C9 – C8	-178.8(2)	C2 – C1 – C5 – C3	119.4(3)
C12 – O1 – C9 – C10	1.7(3)	C4 – C1 – C5 – C3	-120.7(3)
O1 – C9 – C10 – C11	179.0(2)	C16 – C17 – C18 – C13	57.6(3)
C8 – C9 – C10 – C11	-0.6(3)	C2 – C1 – C13 – C14	61.7(3)
C9 – C10 – C11 – C6	-0.1(3)	C3 – C1 – C13 – C14	-179.1(3)
C8 – C7 – C6 – C11	-0.9(3)	C4 – C1 – C13 – C14	-60.1(3)
C8 – C7 – C6 – C5	177.1(2)	C2 – C1 – C13 – C18	-174.6(3)
C10 – C11 – C6 – C7	0.8(3)	C3 – C1 – C13 – C18	-55.4(3)
C10 – C11 – C6 – C5	-177.1(2)	C4 – C1 – C13 – C18	63.6(3)
C6 – C7 – C8 – C9	0.3(3)	C17 – C18 – C13 – C1	-177.8(2)
O1 – C9 – C8 – C7	-179.1(2)	C17 – C18 – C13 – C14	-52.3(3)
C10 – C9 – C8 – C7	0.5(3)	C16 – C15 – C14 – O2	128.7(3)
C13 – C1 – C4 – C5	-178.0(2)	C16 – C15 – C14 – C13	-53.7(3)

C2 – C1 – C4 – C5	43.6(2)	C1 – C13 – C14 – O2	-7.4(4)
C3 – C1 – C4 – C5	-43.6(2)	C18 – C13 – C14 – O2	-131.7(3)
C18 – C17 – C16 – C15	-58.8(3)	C1 – C13 – C14 – C15	175.0(2)
C17 – C16 – C15 – C14	56.2(3)	C18 – C13 – C14 – C15	50.7(3)
C7 – C6 – C5 – C4	121.7(3)	C13 – C1 – C3 – C5	179.1(3)
C11 – C6 – C5 – C4	-60.4(3)	C2 – C1 – C3 – C5	-43.9(2)
C7 – C6 – C5 – C2	1.4(4)	C4 – C1 – C3 – C5	42.7(2)
C11 – C6 – C5 – C2	179.3(2)	C6 – C5 – C3 – C1	177.2(2)
C7 – C6 – C5 – C3	-115.2(3)	C4 – C5 – C3 – C1	-43.6(2)
C11 – C6 – C5 – C3	62.7(4)	C2 – C5 – C3 – C1	43.0(2)
C1 – C4 – C5 – C6	-178.9(2)	C13 – C1 – C2 – C5	178.9(3)
C1 – C4 – C5 – C2	-42.7(2)	C3 – C1 – C2 – C5	44.3(2)
C1 – C4 – C5 – C3	42.9(2)	C4 – C1 – C2 – C5	-43.1(2)
C2 – C1 – C5 – C4	-119.9(2)	C6 – C5 – C2 – C1	-178.3(2)
C3 – C1 – C5 – C4	120.7(3)	C4 – C5 – C2 – C1	44.0(2)
C3 – C1 – C5 – C2	-119.4(3)	C3 – C5 – C2 – C1	-43.7(2)
C4 – C1 – C5 – C2	119.9(2)		

CCDC-2011115 contains supplementary crystallographic data for this compound. These data can be obtained free of charge from The Cambridge Crystallographic Data Centre *via* www.ccdc.cam.ac.uk/data_request/cif.

Ethyl 4-(bicyclo[1.1.1]pentan-1-yl)-1-methylpiperidine-4-carboxylate (6)

Table S15. Details for X-ray data collection and structure refinement for compound **6**.

	**6**
Empirical formula	$C_{14}H_{23}NO_2$
Formula mass	237.33
T[K]	123(2)
Crystal size [mm]	0.40 × 0.40 × 0.35
Crystal description	colorless block
Crystal system	monoclinic
Space group	*P*21/*c*
a [Å]	15.4834(7)
b [Å]	5.9795(3)
c [Å]	14.7917(7)

α [°]	90.0
β [°]	97.994(4)
γ [°]	90.0
V [Å^3]	1356.15(11)
Z	4
$\rho_{calcd.}$ [g cm^{-3}]	1.162
μ [mm^{-1}]	0.077
F(000)	520
Θ range [°]	2.66 – 25.24
Index ranges	$-22 \leq h \leq 22$
	$-8 \leq k \leq 8$
	$-21 \leq l \leq 21$
Reflns. collected	26420
Reflns. obsd.	3121
Reflns. unique	4124 (R_{int} = 0.0472)
R_1, wR_2 (2σ data)	0.0452, 0.1104
R_1, wR_2 (all data)	0.0658, 0.1242
GOOF on F^2	1.026
Peak/hole [e Å^{-3}]	0.377 / -0.198

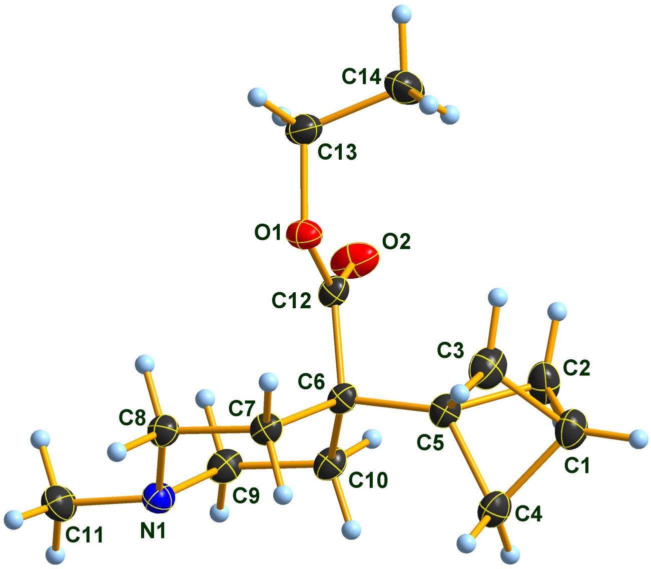

Figure S7. Molecular structure of compound **6** in the crystal. DIAMOND representation; thermal ellipsoids are drawn at 50 % probability level.

Table S16. Selected bond lengths (Å) of compound **6**.

O1 – C12	1.343(1)	C13 – C14	1.508(2)
O1 – C13	1.460(1)	C8 – C7	1.523(2)
C6 – C5	1.526(2)	C5 – C3	1.562(2)
C6 – C12	1.529(1)	C5 – C1	1.881(2)
C6 – C7	1.535(1)	C9 – C10	1.523(2)
C6 – C10	1.540(1)	C1 – C4	1.548(2)
O2 – C12	1.207(1)	C1 – C2	1.553(2)
N1 – C11	1.460(2)	C1 – C3	1.554(2)
N1 – C9	1.462(2)	C5 – C2	1.557(2)
N1 – C8	1.463(1)	C5 – C4	1.561(2)

Table S17. Selected bond angles (°) of compound **6**.

C12 – O1 – C13	116.6(1)	O1 – C13 – C14	110.5(1)
C5 – C6 – C12	106.0(1)	N1 – C8 – C7	110.3(1)
C5 – C6 – C7	110.3(1)	C8 – C7 – C6	112.2(1)
C12 – C6 – C7	113.0(1)	C1 – C3 – C5	74.3(1)
C5 – C6 – C10	109.7(1)	C1 – C4 – C5	74.5(1)
C12 – C6 – C10	109.3(1)	C1 – C2 – C5	74.4(1)
C7 – C6 – C10	108.6(1)	C4 – C5 – C1	52.5(1)
C11 – N1 – C9	110.0(1)	C3 – C5 – C1	52.7(1)
C11 – N1 – C8	110.5(1)	N1 – C9 – C10	111.4(1)
C9 – N1 – C8	110.5(1)	C4 – C1 – C2	87.4(1)
C6 – C5 – C2	128.0(1)	C4 – C1 – C3	87.6(1)
C6 – C5 – C4	125.9(1)	C2 – C1 – C3	87.6(1)
C2 – C5 – C4	86.8(1)	C4 – C1 – C5	53.1(1)
C6 – C5 – C3	128.2(1)	C2 – C1 – C5	52.9(1)
C2 – C5 – C3	87.2(1)	C3 – C1 – C5	53.1(1)
C4 – C5 – C3	86.9(1)	C9 – C10 – C6	112.0(1)
C6 – C5 – C1	178.4(1)	O2 – C12 – O1	123.5(1)
C2 – C5 – C1	52.7(1)	O2 – C12 – C6	124.2(1)
O1 – C12 – C6	112.2(1)		

Table S18. Selected torsion angles (°) of compound **6**.

C12 – C6 – C5 – C2	-53.4(1)	C5 – C6 – C12 – O1	-87.2(1)
C7 – C6 – C5 – C2	-175.9(1)	C7 – C6 – C12 – O1	33.7(1)
C10 – C6 – C5 – C2	64.5(1)	C10 – C6 – C12 – O1	154.6(1)
C12 – C6 – C5 – C4	-171.9(1)	C12 – O1 – C13 – C14	-82.4(1)
C7 – C6 – C5 – C4	65.5(1)	C11 – N1 – C8 – C7	177.3()

C10 – C6 – C5 – C4	-54.0(1)	C9 – N1 – C8 – C7	-60.8(1)
C12 – C6 – C5 – C3	69.0(1)	N1 – C8 – C7 – C6	58.1(1)
C7 – C6 – C5 – C3	-53.6(1)	C5 – C6 – C7 – C8	-172.5(1)
C10 – C6 – C5 – C3	-173.1(1)	C12 – C6 – C7 – C8	69.1(1)
C11 – N1 – C9 – C10	-177.6(1)	C10 – C6 – C7 – C8	-52.3(1)
C8 – N1 – C9 – C10	60.2(1)	C4 – C1 – C3 – C5	-43.9(1)
C2 – C5 – C1 – C4	-119.8(1)	C2 – C1 – C3 – C5	43.6(1)
C3 – C5 – C1 – C4	120.0(1)	C6 – C5 – C3 – C1	178.3(1)
C4 – C5 – C1 – C2	119.8(1)	C2 – C5 – C3 – C1	-43.5(1)
C3 – C5 – C1 – C2	-120.2(1)	C4 – C5 – C3 – C1	43.4(1)
C2 – C5 – C1 – C3	120.2(1)	C2 – C1 – C4 – C5	-43.9(1)
C4 – C5 – C1 – C3	-120.0(1)	C3 – C1 – C4 – C5	43.8(1)
N1 – C9 – C10 – C6	-55.9(1)	C6 – C5 – C4 – C1	179.8(1)
C5 – C6 – C10 – C9	171.5(1)	C2 – C5 – C4 – C1	43.8(1)
C12 – C6 – C10 – C9	-72.7(1)	C3 – C5 – C4 – C1	-43.6(1)
C7 – C6 – C10 – C9	50.9(1)	C4 – C1 – C2 – C5	44.0(1)
C13 – O1 – C12 – O2	-3.5(2)	C3 – C1 – C2 – C5	-43.7(1)
C13 – O1 – C12 – C6	173.5(1)	C6 – C5 – C2 – C1	-178.1(1)
C5 – C6 – C12 – O2	89.8(1)	C4 – C5 – C2 – C1	-43.6(1)
C7 – C6 – C12 – O2	-149.4(1)	C3 – C5 – C2 – C1	43.5(1)
C10 – C6 – C12 – O2	-28.4(1)		

CCDC-2011117 contains supplementary crystallographic data for this compound. These data can be obtained free of charge from The Cambridge Crystallographic Data Centre *via* www.ccdc.cam.ac.uk/data_request/cif.

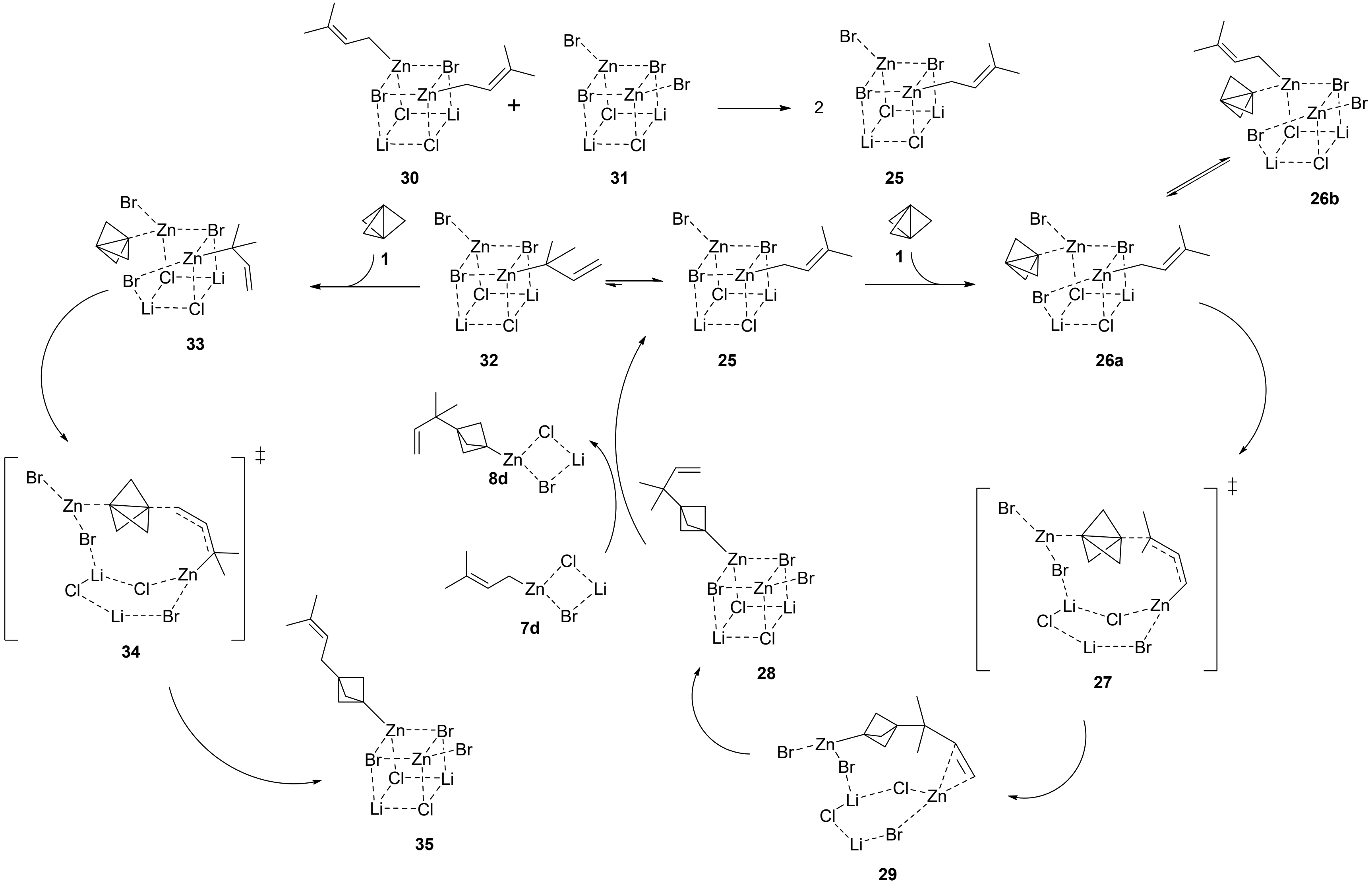

Figure S1. Reaction mechanism for the addition of propellane (**1**) to the prenylzinc reagent **7d** via the cubic cluster **25**.

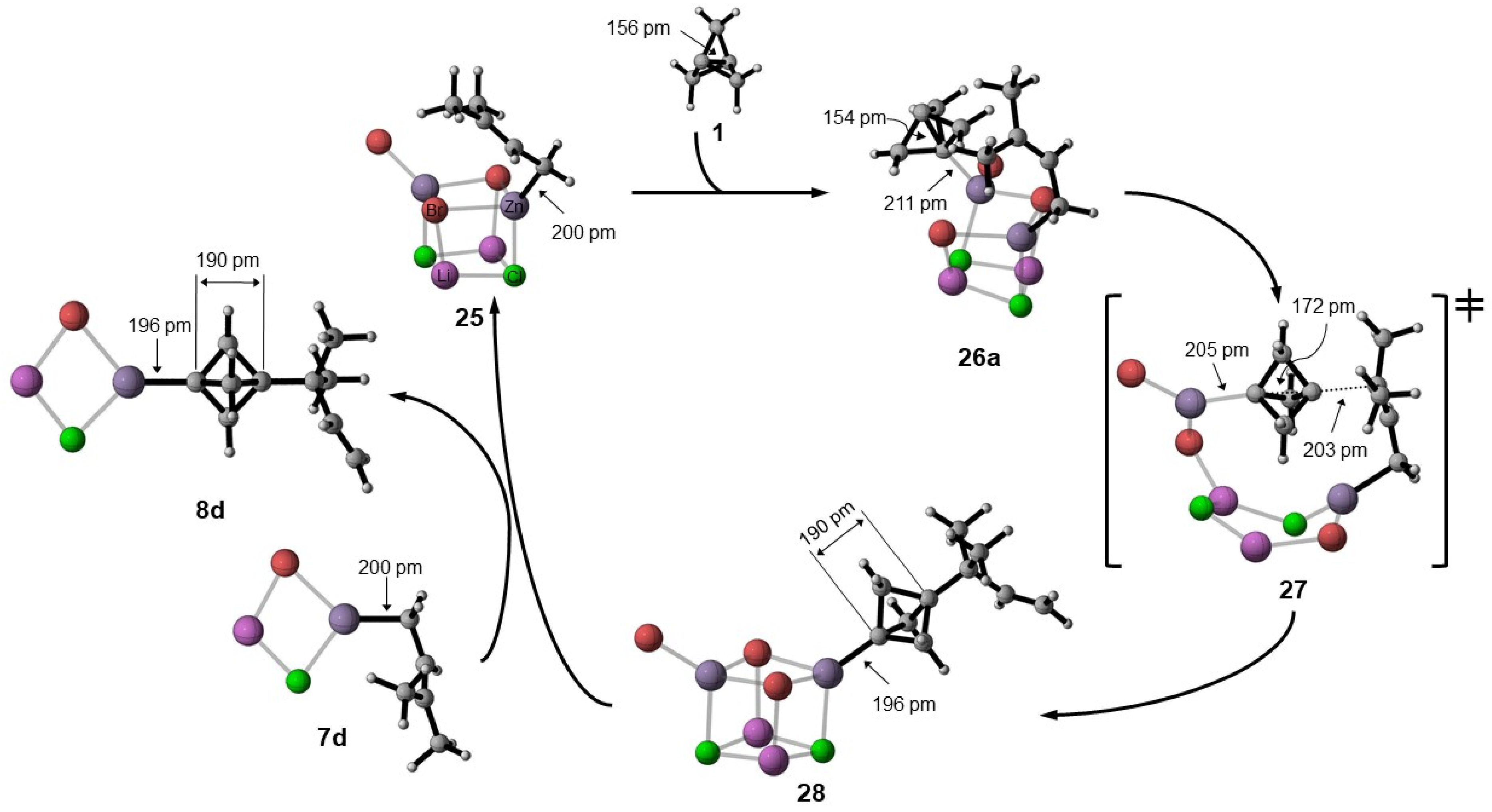

Figure S2. Calculated geometries of selected intermediates shown in Figure S1 (B3LYP-D3/def2SVP).

Table S1a. Total energies and enthalpies for the systems shown in Figure S1 (in kJ/mol).

**system**	ΔH_{298} SMD(THF)/ B2PLYP-D3/ def2TZVPP// [kJ/mol]	ΔG_{298}(qh,1M) SMD(THF)/ B2PLYP-D3/ def2TZVPP// [kJ/mol]
**1+25**	0.0	0.0
**1+32**	+27.9	+28.4
**26a**	-47.3	-3.1
**26b**	-68.8	-25.5
**27**	-0.1	+52.0
**29**	-35.0	+19.6
**28**	-96.6	-42.7
**33**	-20.9	+25.1
**34**	+44.6	+95.6
**35**	-107.6	-56.5

Table S1b. Total energies and enthalpies for the systems shown in Figure S1 (in kJ/mol).

**system**	ΔH_{298} SMD(THF)/ B2PLYP-D3/ def2TZVPP// [kJ/mol]	ΔG_{298}(qh,1M) SMD(THF)/ B2PLYP-D3/ def2TZVPP// [kJ/mol]
**Eq. 32 > 25**	-27.9	-28.4
**30 + 31 > 2 x 25**	-6.9	-4.4
**Step 1: 1 + 25 > 26a**	-47.3	-3.1
**Step 2: 26a > 27**	+47.2	+55.1
**Step 3: 27 > 29**	-34.9	-32.4
**Step 4: 29 > 28**	-61.6	-62.3
**Step 5: 28 + 7d > 25 + 8d**	-2.5	-2.5
**sum of steps (1 + 7d > 8d)**	-99.1	-45.2
**Step 1′: 1 + 32 > 33**	-48.8	-3.3

**Step 2′: 33 > 34**	+65.5	+70.5
**Step 3′ + 4′: 34 > 35**	-152.1	-152.1

Table S1c. Total energies and enthalpies for the systems shown in Figure S1 (in Hartree).

**system**	E_{tot} B3LYP-D3/ def2SVP	H_{298} B3LYP-D3/ def2SVP	G_{298} B3LYP-D3/ def2SVP	<S^2> B3LYP-D3/ def2SVP	G_{298}(qh) B3LYP-D3/ def2SVP	E_{tot} B2PLYP-D3/ def2TZVPP// B3LYP-D3/ def2SVP	<S^2> B2PLYP-D3/ def2TZVPP// B3LYP-D3/ def2SVP
**1**							
kn13_005	**-193.87237324**	-193.774506	-193.803999	0.00	-193.803999	-193.9042284	0.00
kn13_002	-193.87235922	-193.774498	-193.805682	0.00	-193.805682	-193.9042207	0.00
**25**							
ks01_085	**-12411.5110482**	-12411.352496	-12411.432143	0.00	-12411.427599	-12411.0653400	0.00
ks01_084	-12411.5104746	-12411.351721	-12411.431056	0.00	-12411.426373	-12411.0641721	0.00
ks01_086	-12411.5094574	-12411.351089	-12411.432633	0.00	-12411.426892	-12411.0649448	0.00
ks01_006	-12411.5090628	-12411.350600	-12411.432506	0.00	-12411.426177	-12411.0654693	0.00
ks01_002	-12411.5089516	-12411.350515	-12411.432601	0.00	-12411.426285	-12411.0649217	0.00
ks01_005	-12411.5086638	-12411.350324	-12411.431980	0.00	-12411.426091		0.00
**26a**							
ks01_024 (from ks01_023)	**-12605.4152459**	-12605.156649	-12605.251456	0.00	-12605.243086	-12604.998245	0.00
ks01_023	-12605.4151470	-12605.156301	-12605.249776	0.00	-12605.242495	-12604.9975198	0.00
ks01_090	-12605.4148468	-12605.156225	-12605.249669	0.00	-12605.242501		0.00
ks01_034 (from ks01_021)	-12605.4140028	-12605.155483	-12605.249663	0.00	-12605.242096	-12604.996697	0.00
ks01_021	-12605.4137674	-12605.155215	-12605.248320	0.00	-12605.241204	-12604.9959609	0.00
ks01_036 (from ks01_013)	-12605.4137559	-12605.155175	-12605.249919	0.00	-12605.241970		0.00
ks01_091	-12605.4135722	-12605.154924	-12605.250584	0.00			0.00
ks01_037 (from ks01_020)	-12605.4135522	-12605.154932	-12605.251021	0.00	-12605.242016		0.00

ks01_035 (from ks01_019)	-12605.4133826	-12605.154734	-12605.249638	0.00	-12605.241274		0.00
ks01_019	-12605.4132439	-12605.154567	-12605.248122	0.00	-12605.240648		0.00
ks01_013	-12605.4131086	-12605.154491	-12605.248727	0.00	-12605.240952		0.00
ks01_020	-12605.4130679	-12605.154399	-12605.249756	0.00	-12605.241095		0.00
ks01_027 (from ks01_022)	-12605.4126888	-12605.154128	-12605.249455	0.00	-12605.240981		0.00
ks01_022	-12605.4119964	-12605.153462	-12605.248601	0.00	-12605.240038		0.00
**26b**							
ks01_026	**-12605.4188114**	-12605.160220	-12605.253088	0.00	-12605.246978	-12605.0022938	0.00
ks01_040	-12605.4186432	-12605.160117	-12605.253158	0.00	-12605.246883	-12605.0025190	0.00
ks01_042	-12605.4098360	-12605.151405	-12605.245772	0.00	-12605.238174		0.00
ks01_041	-12605.4098354	-12605.151404	-12605.245781	0.00	-12605.238174		0.00
**27**							
ks01_050	-12605.3825283	-12605.124945	-12605.216249	0.00	-12605.208368	-12604.9667026	0.00
**29**							
ks01_051	-12605.4032362	-12605.142944	-12605.231908	0.00	-12605.225409	-12604.9869363	0.00
**28**							
ks01_014	**-12605.4371295**	-12605.177082	-12605.268961	0.00	-12605.259814	-12605.0211343	0.00
ks01_011	-12605.4371134	-12605.177034	-12605.268950	0.00	-12605.259735	-12605.0211232	0.00
ks01_012	-12605.4370486	-12605.177082	-12605.269143	0.00	-12605.259890		0.00
ks01_010	-12605.4370146	-12605.176998	-12605.268294	0.00	-12605.259761		0.00
ks01_038 (from ks01_010)	-12605.4367909	-12605.176699	-12605.267519	0.00	-12605.259407		0.00
ks01_015	-12605.4336522	-12605.173626	-12605.265466	0.00	-12605.256550		0.00
**7d**							
kn13_009	-5016.6575135	-5016.514420	-5016.572439	0.00	-5016.569117	-5016.4976610	0.00
kn13_008	-5016.6573729	-5016.514302	-5016.572214	0.00	-5016.569054	-5016.4980160	0.00
kn13_004	-5016.6573728	-5016.514302	-5016.572214	-	-	-	-
kn13_001	-5016.6567449	-5016.513644	-5016.572378	0.00	-5016.568774	-5016.4975508	0.00

**8d**							
kn13_013	-5210.5856109	-5210.340783	-5210.408077	0.00	-5210.403184	-5210.4549339	0.00
kn13_011	-5210.5855788	-5210.341796	-5210.406118	0.00	-5210.402634	-5210.4549318	0.00
**30**							
ks02_042	**-10033.3929298**	-10033.104189	-10033.194703	0.00	-10033.189135	-10033.0573258	0.00
ks02_038	-10033.3927713	-10033.104375	-10033.196645	0.00	-10033.190157	-10033.0571647	0.00
ks02_039	-10033.3922736	-10033.103966	-10033.196127	0.00			0.00
ks02_003	-10033.3921815	-10033.103941	-10033.198071	0.00	-10033.189771	-10033.0560727	0.00
ks02_037	-10033.3908662	-10033.102662	-10033.195757	0.00	-10033.188428		0.00
ks02_040	-10033.3906193	-10033.102392	-10033.196595	0.00			0.00
ks02_041	-10033.3894988	-10033.101210	-10033.195488	0.00			0.00
**31**							
ks01_108	-14789.6266544	-14789.598119	-14789.666955	0.00	-14789.663493	-14789.0735976	0.00
**32**							
ks01_083	**-12411.5003651**	-12411.341884	-12411.424430	0.00	-12411.416821	-12411.5285638	0.00
ks01_097	-12411.5002209	-12411.341705	-12411.423409	0.00	-12411.416611	-12411.5283789	0.00
**33**							
ks01_110 (from ks01_024)	-12605.4058751	-12605.147206	-12605.240677	0.00	-12605.232991	-12604.988232	0.00
**34**							
ks01_111 (from ks01_050)	-12605.3733249	-12605.115643	-12605.208393	0.00	-12605.199519	-12604.95576	0.00
**35**							
ks01_112	-12605.4421728	-12605.182058	-12605.275534	0.00	-12605.265748	-12605.023727	0.00
ks01_113	-12605.4422142	-12605.182165	-12605.276281	0.00	-12605.266013	-12605.023752	0.00
ks01_114	-12605.4421319	-12605.182082	-12605.275692	0.00			0.00

Table S1d. Total energies and enthalpies for the systems shown in Figure S1 (in Hartree).

**system**	E_{tot} B3LYP-D3/ def2SVP	H_{298} B3LYP-D3/ def2SVP	G_{298}(qh) B3LYP-D3/ def2SVP (qh)	E_{tot} B2PLYP-D3/ def2TZVPP// B3LYP-D3/ def2SVP	E_{tot} SMD(THF)/ B3LYP-D3/ def2SVP	G_{298}(qh,1M) SMD(THF)/ B2PLYP-D3/ def2TZVPP// B3LYP-D3/ def2SVP
**1**						
kn13_005	**-193.87237324**	-193.774506	-193.803999	-193.9042284	-193.88523394	-193.8456964
kn13_002	-193.87235922	-193.774498	-193.805682	-193.9042207	-193.88523469	**-193.8474005**
**25**						
ks01_085	**-12411.5110482**	-12411.352496	-12411.427599	-12411.0653400	-12411.5408086	**-12411.0086327**
ks01_084	-12411.5104746	-12411.351721	-12411.426373	-12411.0641721	-12411.5412006	-12411.0077780
ks01_086	-12411.5094574	-12411.351089	-12411.426892	-12411.0649448	-12411.5373398	-12411.0072433
ks01_006	-12411.5090628	-12411.350600	-12411.426177	-12411.0654693	-12411.5368335	-12411.0073357
ks01_002	-12411.5089516	-12411.350515	-12411.426285	-12411.0649217	-12411.5367619	-12411.0070469
ks01_005	-12411.5086638	-12411.350324	-12411.426091		-12411.5363737	
**26a**						
ks01_024 (from ks01_023)	**-12605.4152459**	-12605.156649	-12605.243086	-12604.998245	-12605.4493860	**-12604.8572067**
ks01_023	-12605.4151470	-12605.156301	-12605.242495	-12604.9975198	-12605.4480332	-12604.8547355
ks01_090	-12605.4148468	-12605.156225	-12605.242501	-12604.9980338	-12605.4492291	-12604.8570518
ks01_034 (from ks01_021)	-12605.4140028	-12605.155483	-12605.242096	-12604.996697	-12605.4483264	-12604.8560953
ks01_021	-12605.4137674	-12605.155215	-12605.241204	-12604.9959609	-12605.4473458	-12604.8539574
ks01_036 (from ks01_013)	-12605.4137559	-12605.155175	-12605.241970		-12605.4476853	
ks01_037 (from ks01_020)	-12605.4135522	-12605.154932	-12605.242016		-12605.4473518	
ks01_035 (from ks01_019)	-12605.4133826	-12605.154734	-12605.241274		-12605.4483837	
ks01_019	-12605.4132439	-12605.154567	-12605.240648		-12605.4475184	
ks01_013	-12605.4131086	-12605.154491	-12605.240952		-12605.4465225	

ks01_020	-12605.4130679	-12605.154399	-12605.241095		-12605.4465365	
ks01_027 (from ks01_022)	-12605.4126888	-12605.154128	-12605.240981		-12605.4470863	
ks01_022	-12605.4119964	-12605.153462	-12605.240038		-12605.4459708	
**26b**						
ks01_026	-12605.4188114	-12605.160220	-12605.246978	-12605.0022938	-12605.4571148	**-12604.8657453**
ks01_040	-12605.4186432	-12605.160117	-12605.246883	-12605.002519	-12605.4565069	-12604.8656040
ks01_042	-12605.4098360	-12605.151405	-12605.238174		-12605.4472447	
ks01_041	-12605.4098354	-12605.151404	-12605.238174		-12605.4472396	
**27**						
ks01_050	-12605.3825283	-12605.124945	-12605.208368	-12604.9667026	-12605.429241	**-12604.8362365**
**29**						
ks01_051	-12605.4032362	-12605.142944	-12605.225409	-12604.9869363	-12605.4457196	**-12604.8485740**
**28**						
ks01_014	**-12605.4371295**	-12605.177082	-12605.259814	-12605.0211343	-12605.4686426	**-12604.8723134**
ks01_011	-12605.4371134	-12605.177034	-12605.259735	-12605.0211232	-12605.4685560	-12604.8721689
ks01_012	-12605.4370486	-12605.177082	-12605.259890		-12605.4680948	
ks01_010	-12605.4370146	-12605.176998	-12605.259761		-12605.4681572	
ks01_038 (from ks01_010)	-12605.4367909	-12605.176699	-12605.259407		-12605.4682164	
ks01_015	-12605.4336522	-12605.173626	-12605.256550		-12605.4646365	
**7d**						
kn13_009	-5016.6575135	-5016.514420	-5016.569117	-5016.4976610	-5016.6817050	-5016.4304375
kn13_008	-5016.6573729	-5016.514302	-5016.569054	-5016.4980160	-5016.6821803	**-5016.4314860**
kn13_001	-5016.6567449	-5016.513644	-5016.568774	-5016.4975508	-5016.6810878	-5016.4309043
**8d**						
kn13_013	-5210.5856109	-5210.340783	-5210.403184	-5210.4549339	-5210.6122602	**-5210.2961378**
kn13_011	-5210.5855788	-5210.341796	-5210.402634	-5210.4549318	-5210.6121604	-5210.2955501
**30**						
ks02_042	-10033.3929298	-10033.104189	-10033.189135	-10033.0571647	-10033.4190869	-10032.8765085
ks02_038	-10033.3927713	-10033.104375	-10033.190157	-10033.0571647	-10033.4186489	**-10032.8774095**

ks02_039	-10033.3922736	-10033.103966				
ks02_003	-10033.3921815	-10033.103941	-10033.189771	-10033.0560727	-10033.4172632	-10032.8757254
ks02_037	-10033.3908662	-10033.102662	-10033.188428			
ks02_040	-10033.3906193	-10033.102392				
ks02_041	-10033.3894988	-10033.101210				
**31**						
ks01_108	-14789.6266544	-14789.598119	-14789.663493	-14789.0735976	-14789.6574140	-14789.1381773
**32**						
ks01_083	-12411.5003651	-12411.341884	-12411.416821	-12411.0561902	-12411.5285638	**-12410.9978263**
ks01_097	-12411.5002209	-12411.341705	-12411.416611	-12411.0559051	-12411.5283789	-12410.9974347
**33**						
ks01_110 (from ks01_024)	-12605.4058751	-12605.147206	-12605.232991	-12604.988232	-12605.4400322	-12604.8464865
**34**						
ks01_111 (from ks01_050)	-12605.3733249	-12605.115643	-12605.199519	-12604.95576	-12605.4140140	-12604.8196247
**35**						
ks01_113	-12605.4422142	-12605.182165	-12605.266013	-12605.023752	-12605.4752537	**-12604.7967423**
ks01_112	-12605.4421728	-12605.182058	-12605.265748	-12605.023727	-12605.4751313	-12604.7965707
ks01_114	-12605.4421319	-12605.182082				

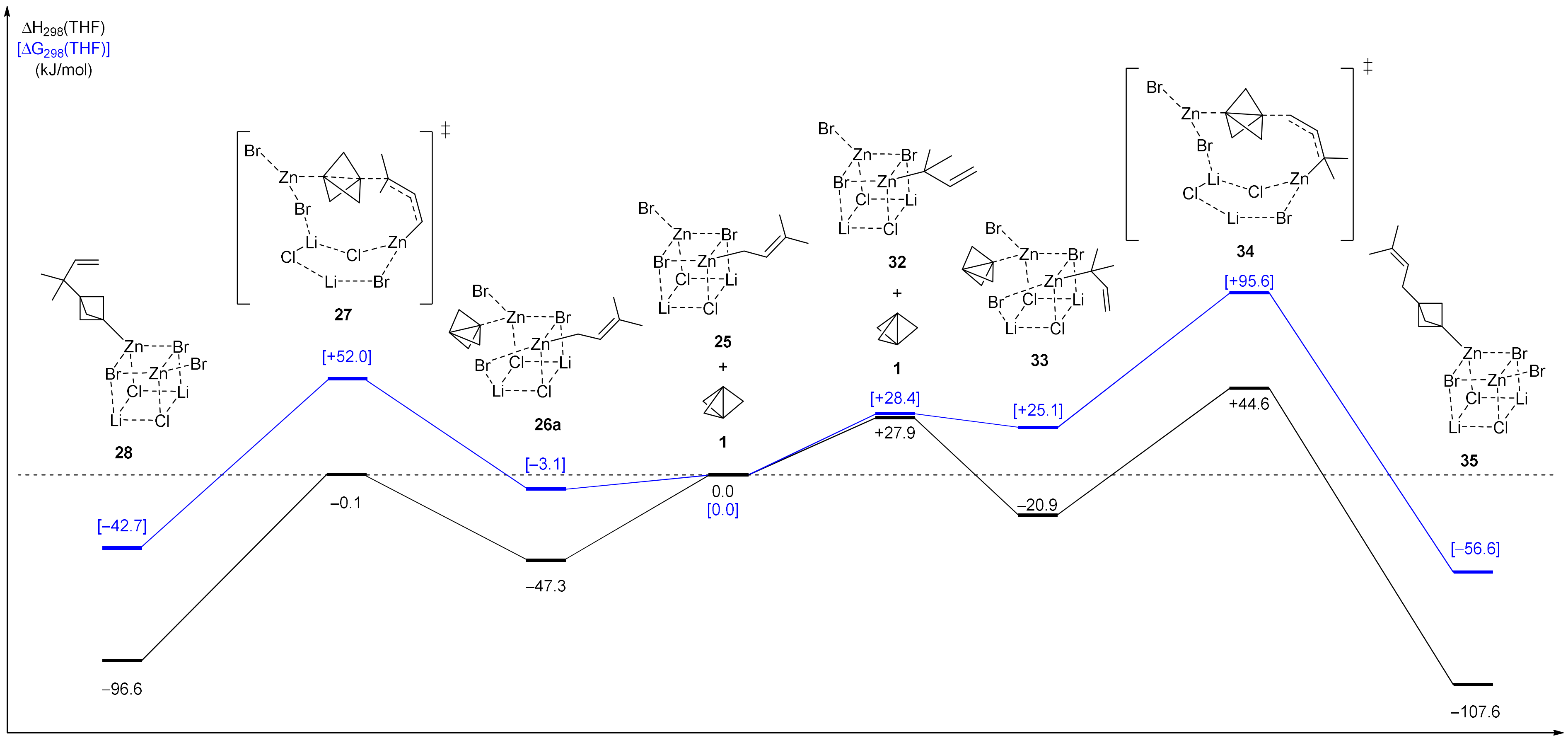

Figure S3. Comparison of the Enthalpies and Free Energies of the reactions of the two regioisomeric clusters **25** and **32** (SMD(THF)/B2PLYP-D3/def2TZVPP)

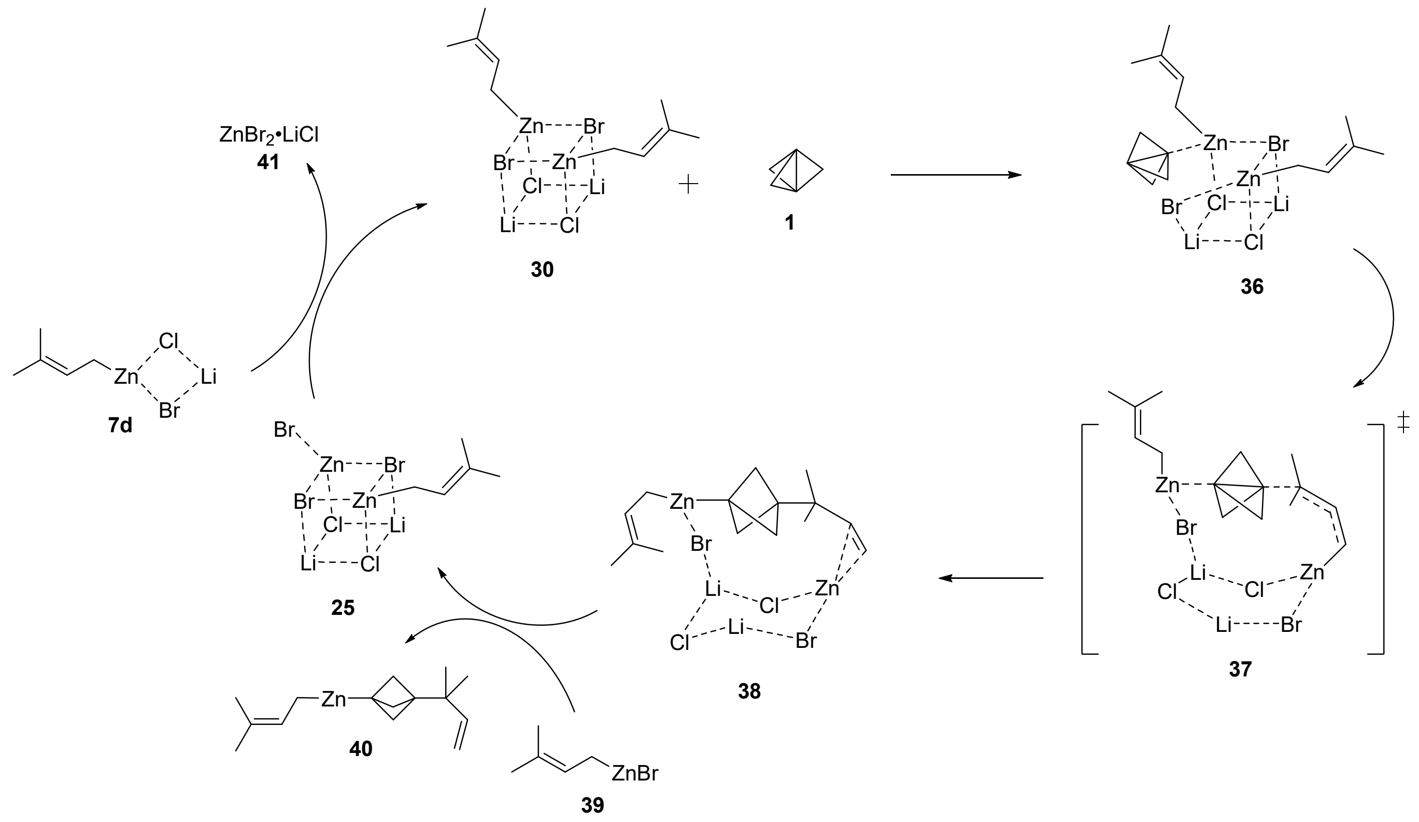

Figure S4. Reaction mechanism for the addition of propellane (**1**) to the prenylzinc reagent **7d** via the cubic cluster **30**.

Table S2a. Total energies and enthalpies for the systems shown in Figure S4 (in kJ/mol).

system	ΔH_{298} SMD(THF)/ B2PLYP-D3/ def2TZVPP// [kJ/mol]	ΔG_{298}(qh,1M) SMD(THF)/ B2PLYP-D3/ def2TZVPP// [kJ/mol]
**1+30**	0.0	0.0
**36**	-58.4	-11.3
**36a**	-54.5	-8.4
**37**	+16.0	+71.6
**38**	-14.9	+44.1

Table S2b. Total energies and enthalpies for the systems shown in Figure S4 (in kJ/mol).

system	ΔH_{298} SMD(THF)/ B2PLYP-D3/ def2TZVPP// [kJ/mol]	ΔG_{298}(qh,1M) SMD(THF)/ B2PLYP-D3/ def2TZVPP// [kJ/mol]
**Step 1: 1 + 30 > 36**	-58.4	-11.3
**Step 2: 36 > 37**	+74.4	+82.9
**Step 3: 37 > 38**	-30.9	-27.5
**38 + 39 > 25 + 40**	-103.5	-107.7
**25 + 7d > 41 + 30**	+11.4	+10.5
**sum of steps (1 + 39 + 7d > 40 + 41)**	-106.9	-53.1

Table S2c. Total energies and enthalpies for the systems shown in Figure S4 (in Hartree).

**system**	E_{tot} B3LYP-D3/ def2SVP	H_{298} B3LYP-D3/ def2SVP	G_{298} B3LYP-D3/ def2SVP	<S^2> B3LYP-D3/ def2SVP	G_{298}(qh) B3LYP-D3/ def2SVP	E_{tot} B2PLYP-D3/ def2TZVPP// B3LYP-D3/ def2SVP	<S^2> B2PLYP-D3/ def2TZVPP// B3LYP-D3/ def2SVP
**1**							
kn13_005	**-193.87237324**	-193.774506	-193.803999	0.00	-193.803999	-193.9042284	0.00
kn13_002	-193.87235922	-193.774498	-193.805682	0.00	-193.805682	-193.9042207	0.00
**30**							
ks02_042	**-10033.3929298**	-10033.104189	-10033.194703	0.00	-10033.189135	-10033.0573258	0.00
ks02_038	-10033.3927713	-10033.104375	-10033.196645	0.00	-10033.190157	-10033.0571647	0.00
ks02_039	-10033.3922736	-10033.103966	-10033.196127	0.00			0.00
ks02_003	-10033.3921815	-10033.103941	-10033.198071	0.00	-10033.189771	-10033.0560727	0.00
ks02_037	-10033.3908662	-10033.102662	-10033.195757	0.00	-10033.188428		0.00
ks02_040	-10033.3906193	-10033.102392	-10033.196595	0.00			0.00
ks02_041	-10033.3894988	-10033.101210	-10033.195488	0.00			0.00
**36**							
ks02_012	**-10227.3052237**	-10226.916679	-10227.021301	0.00	-10227.012715	-10226.9960038	0.00
ks02_010	-10227.2984725	-10226.910081	-10227.014552	0.00	-10227.006872	-10226.9905268	0.00
ks02_001	-10227.2978486	-10226.909531	-10227.013319	0.00	-10227.005947	-10226.9896733	0.00
ks02_009	-10227.2975953	-10226.909347	-10227.013587	0.00			0.00
ks02_014	-10227.2975313	-10226.909173	-10227.012846	0.00			0.00
ks02_013	-10227.2968184	-10226.908633	-10227.013779	0.00			0.00
ks02_011	-10227.2956843	-10226.907444	-10227.014436	0.00			0.00
ks02_015	-10227.2917191	-10226.903294	-10227.008026	0.00			0.00
ks02_016	-10227.2902996	-10226.901975	-10227.008498	0.00			0.00
**37**							
ks02_018	-10227.2626470	-10227.875338	-10227.975248	0.00	-10226.967933	-10226.9542079	0.00
ks02_008	-10227.2612208	-10226.873876	-10226.974168	0.00	-10226.966646	-10226.9529420	0.00
**38**							
ks02_033	-10227.2808404	-10226.891156	-10226.989474	0.00	-10226.982642	-10226.9713185	0.00
**39**							
ks02_044	-4548.92915993	-4548.792373	-4548.841518	0.00	-4548.839792	-4548.7779531	0.00

**40**							
ks02_035	-2364.73274204	-2364.364799	-2364.436727	0.00	-2364.430222	-2364.7183248	0.00
**25**							
ks01_085	**-12411.5110482**	-12411.352496	-12411.432143	0.00	-12411.427599	-12411.0653400	0.00
ks01_084	-12411.5104746	-12411.351721	-12411.431056	0.00	-12411.426373	-12411.0641721	0.00
ks01_086	-12411.5094574	-12411.351089	-12411.432633	0.00	-12411.426892	-12411.0649448	0.00
ks01_006	-12411.5090628	-12411.350600	-12411.432506	0.00	-12411.426177	-12411.0654693	0.00
ks01_002	-12411.5089516	-12411.350515	-12411.432601	0.00	-12411.426285	-12411.0649217	0.00
ks01_005	-12411.5086638	-12411.350324	-12411.431980	0.00	-12411.426091		0.00
**7d**							
kn13_009	-5016.6575135	-5016.514420	-5016.572439	0.00	-5016.569117	-5016.4976610	0.00
kn13_008	-5016.6573729	-5016.514302	-5016.572214	0.00	-5016.569054	-5016.4980160	0.00
kn13_004	-5016.6573728	-5016.514302	-5016.572214	-	-	-	-
kn13_001	-5016.6567449	-5016.513644	-5016.572378	0.00	-5016.568774	-5016.4975508	0.00
**41**							
ks02_030	-7394.77658141	-7394.763260	-7394.808503	0.00	-7394.807686	-7394.5089863	0.00

Table S2d. Total energies and enthalpies for the systems shown in Figure S4 (in Hartree).

**system**	E_{tot} B3LYP-D3/ def2SVP	H_{298} B3LYP-D3/ def2SVP	G_{298}(qh) B3LYP-D3/ def2SVP (qh)	E_{tot} B2PLYP-D3/ def2TZVPP// B3LYP-D3/ def2SVP	E_{tot} SMD(THF)/ B3LYP-D3/ def2SVP	G_{298}(qh,1M) SMD(THF)/ B2PLYP-D3/ def2TZVPP// B3LYP-D3/ def2SVP
**1**						
kn13_005	**-193.87237324**	-193.774506	-193.803999	-193.9042284	-193.88523394	-193.8456964
kn13_002	-193.87235922	-193.774498	-193.805682	-193.9042207	-193.88523469	**-193.8474005**
**30**						
ks02_042	-10033.3929298	-10033.104189	-10033.189135	-10033.0571647	-10033.4190869	-10032.8765085
ks02_038	-10033.3927713	-10033.104375	-10033.190157	-10033.0571647	-10033.4186489	**-10032.8774095**
ks02_039	-10033.3922736	-10033.103966				
ks02_003	-10033.3921815	-10033.103941	-10033.189771	-10033.0560727	-10033.4172632	-10032.8757254
ks02_037	-10033.3908662	-10033.102662	-10033.188428			
ks02_040	-10033.3906193	-10033.102392				
ks02_041	-10033.3894988	-10033.101210				

**36**						
ks02_012	-10227.3052237	-10226.916679	-10227.012715	-10226.9960038	-10227.3338794	**-10226.7291323**
ks02_010	-10227.2984725	-10226.910081	-10227.006872	-10226.9905268	-10227.3305239	-10226.7279592
ks02_001	-10227.2978486	-10226.909531	-10227.005947	-10226.9896733	-10227.3311231	-10226.7280277
ks02_009	-10227.2975953	-10226.909347				
ks02_014	-10227.2975313	-10226.909173				
ks02_013	-10227.2968184	-10226.908633				
ks02_011	-10227.2956843	-10226.907444				
ks02_015	-10227.2917191	-10226.903294				
ks02_016	-10227.2902996	-10226.901975				
**37**						
ks02_008	-10227.2612208	-10226.873876	-10226.966646	-10226.9529420	-10227.3034165	**-10226.6975444**
ks02_018	-10227.2626470	-10227.875338	-10226.967933	-10226.9542079	-10227.3024963	-10226.6963247
**38**						
ks02_033	-10227.2808404	-10226.891156	-10226.982642	-10226.9713185	-10227.3187442	-10226.7080054
**39**						
ks02_044	-4548.92915993	-4548.792373	-4548.839792	-4548.7779531	-4548.94937872	-4548.7057855
**40**						
ks02_035	-2364.73274204	-2364.364799	-2364.430222	-2364.7183248	-2364.76613737	-2364.4461816
**25**						
ks01_085	**-12411.5110482**	-12411.352496	-12411.427599	-12411.0653400	-12411.5408086	**-12411.0086327**
ks01_084	-12411.5104746	-12411.351721	-12411.426373	-12411.0641721	-12411.5412006	-12411.0077780
ks01_086	-12411.5094574	-12411.351089	-12411.426892	-12411.0649448	-12411.5373398	-12411.0072433
ks01_006	-12411.5090628	-12411.350600	-12411.426177	-12411.0654693	-12411.5368335	-12411.0073357
ks01_002	-12411.5089516	-12411.350515	-12411.426285	-12411.0649217	-12411.5367619	-12411.0070469
ks01_005	-12411.5086638	-12411.350324	-12411.426091		-12411.5363737	
**7d**						
kn13_009	-5016.6575135	-5016.514420	-5016.569117	-5016.4976610	-5016.6817050	-5016.4304375
kn13_008	-5016.6573729	-5016.514302	-5016.569054	-5016.4980160	-5016.6821803	**-5016.4314860**
kn13_001	-5016.6567449	-5016.513644	-5016.568774	-5016.4975508	-5016.6810878	-5016.4309043
**41**						
ks02_030	-7394.77658141	-7394.763260	-7394.807686	-7394.5089863	-7394.79822615	-7394.5587171

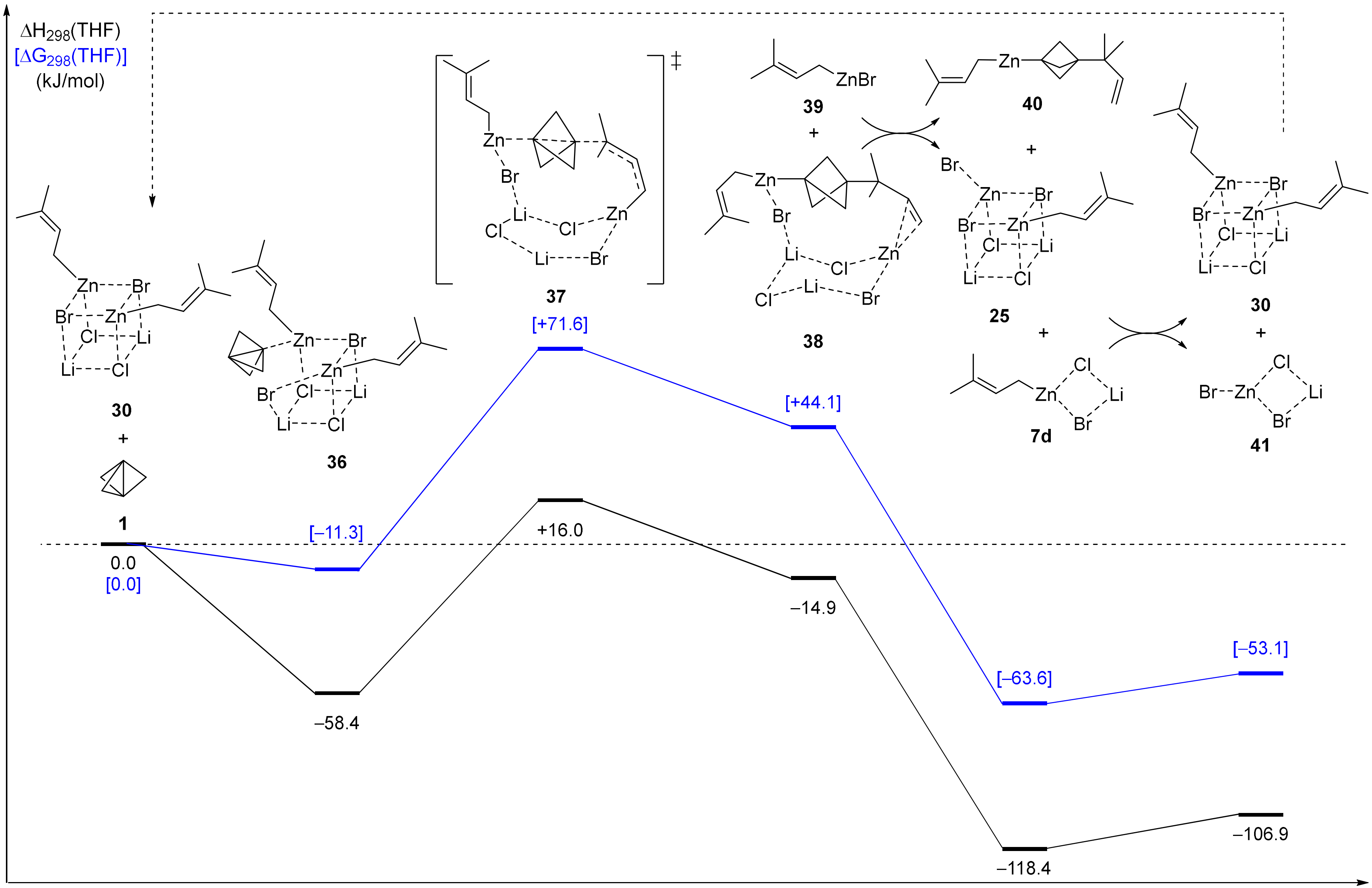

Figure S5. Enthalpies and Free Energies for the reaction with the symmetric cluster **30** (SMD(THF)/B2PLYP-D3/def2TZVPP//B3LYP-D3/def2SVP)

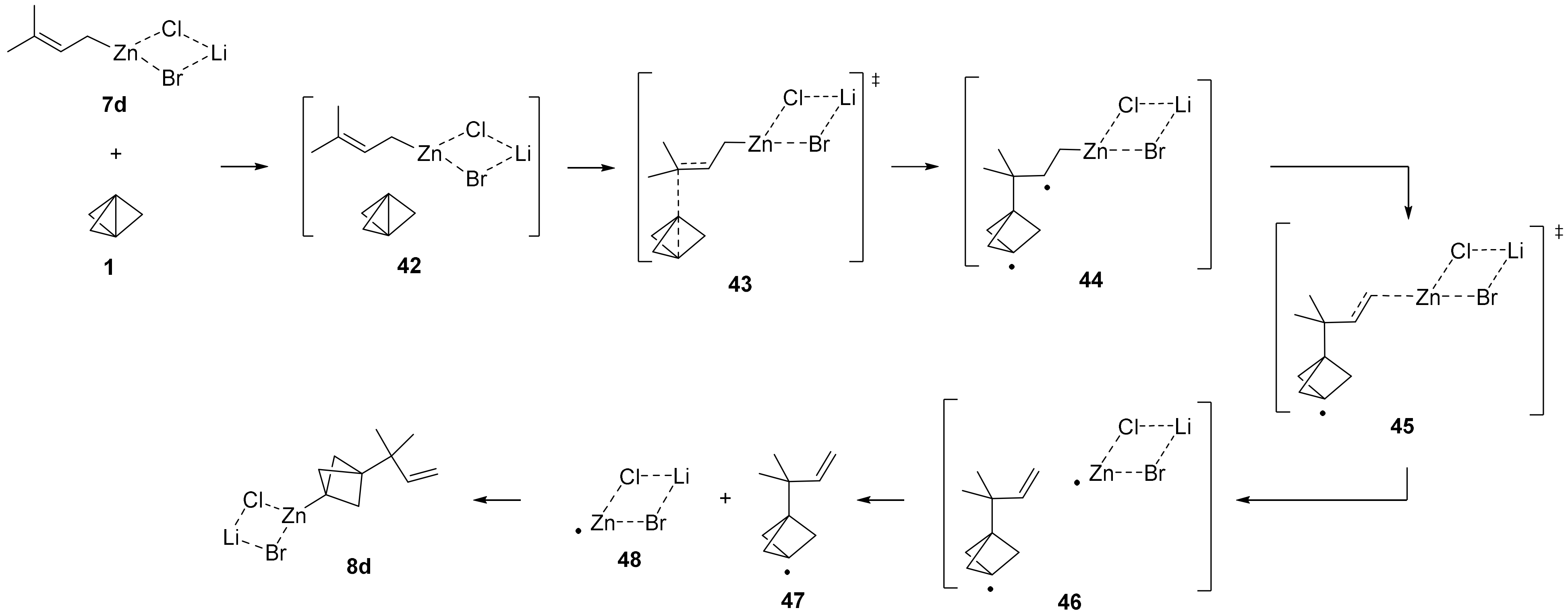

Figure S6. Reaction of prenylzinc bromide (**7d**) with [1.1.1]propellane (**1**) via a radical mechanism.

Table S3a. Total energies and enthalpies for the systems shown in Figure S6 (in Hartree).

system	ΔH298 SMD(THF)/ B2PLYP-D3/ def2TZVPP// [kJ/mol]	ΔG298(qh,1M) SMD(THF)/ B2PLYP-D3/ def2TZVPP// [kJ/mol]
**7d + 1**	0.0	0.0
**44**	+145.6	+200.1
**47 + 48**	+171.8	+173.0
**8d**	-101.7	-45.3

Table S3b. Total energies and enthalpies for the systems shown in Figure S6 (in Hartree).

**system**	E_{tot} B3LYP-D3/ def2SVP	H_{298} B3LYP-D3/ def2SVP	G_{298} B3LYP-D3/ def2SVP	$\langle S^2 \rangle$ B3LYP-D3/ def2SVP	G_{298}(qh) B3LYP-D3/ def2SVP	E_{tot} B2PLYP-D3/ def2TZVPP// B3LYP-D3/ def2SVP	$\langle S^2 \rangle$ B2PLYP-D3/ def2TZVPP// B3LYP-D3/ def2SVP
**7d**							
kn13_009	-5016.6575135	-5016.514420	-5016.572439	0.00	-5016.569117	-5016.4976610	0.00
kn13_008	-5016.6573729	-5016.514302	-5016.572214	0.00	-5016.569054	-5016.4980160	0.00
kn13_004	-5016.6573728	-5016.514302	-5016.572214	-	-	-	-
kn13_001	-5016.6567449	-5016.513644	-5016.572378	0.00	-5016.568774	-5016.4975508	0.00
**1**							
kn13_002	-193.87235922	-193.774498	-193.805682	0.00	-193.805682	-193.9042207	0.00
kn13_005	-193.87237324	-193.774506	-193.803999	0.00	-193.803999	-193.9042284	0.00
**42**							
-	-	-	-	-	-	-	-
**43**							
-	-	-	-	-	-	-	-
**44**							
kn13_029	-5210.4979245	-5210.255658	-5210.321041	0.8787	-5210.317893	-5210.3559403	0.9737
kn13_019	-5210.4979244	-5210.255658	-5210.321040	0.8789	-	-	-
kn13_021	-5210.4977604	-5210.255460	-5210.320982	0.8746	-5210.317633	-5210.3561191	0.9723
kn13_003	-5210.4922501	-5210.250295	-5210.317661	0.9158	-5210.312855	-5210.3527084	0.9870
kn13_014	-5210.4918486	-5210.249941	-5210.317646	0.9179	-5210.312424	-5210.3528726	0.9879
kn13_023	-5210.4904601	-5210.248236	-5210.315354	1.0052	-5210.310635	-5210.3504417	1.0197
**45**							
-	-	-	-	-	-	-	-
**46**							
-	-	-	-	-	-	-	-
**47**							
kn13_012	-389.6625870	-389.430669	-389.477303	0.7525	-389.476447	-389.7053962	0.7551
kn13_015	-389.6625870	-	-	-	-	-	-

**48**							
kn13_006	-4820.8040892	-4820.793801	-4820.833484	0.7511	-4820.833484	-4820.6335342	0.7523
**8d**							
kn13_013	-5210.5856109	-5210.340783	-5210.408077	0.00	-5210.403184	-5210.4549339	0.00
kn13_011	-5210.5855788	-5210.341796	-5210.406118	0.00	-5210.402634	-5210.4549318	0.00

Table S3c. Total energies and enthalpies for the systems shown in Figure S6 (in Hartree).

**system**	E_{tot} B3LYP-D3/ def2SVP	H_{298} B3LYP-D3/ def2SVP	G_{298}(qh) B3LYP-D3/ def2SVP (qh)	E_{tot} B2PLYP-D3/ def2TZVPP// B3LYP-D3/ def2SVP	E_{tot} SMD(THF)/ B3LYP-D3/ def2SVP	G_{298}(qh,1M) SMD(THF)/ B2PLYP-D3/ def2TZVPP// B3LYP-D3/ def2SVP
**7d**						
kn13_009	-5016.6575135	-5016.514420	-5016.569117	-5016.4976610	-5016.6817050	-5016.4304375
kn13_008	-5016.6573729	-5016.514302	-5016.569054	-5016.4980160	-5016.6821803	**-5016.4314860**
kn13_001	-5016.6567449	-5016.513644	-5016.568774	-5016.4975508	-5016.6810878	-5016.4309043
**1**						
kn13_002	-193.87235922	-193.774498	-193.805682	-193.9042207	-193.88523469	**-193.8474005**
kn13_005	-193.87237324	-193.774506	-193.803999	-193.9042284	-193.88523394	-193.8456964
**44**						
kn13_029	-5210.4979245	-5210.255658	-5210.317893	-5210.3559403	-5210.5274382	-5210.2024040
kn13_021	-5210.4977604	-5210.255460	-5210.317633	-5210.3561191	-5210.5274685	**-5210.2026813**
kn13_003	-5210.4922501	-5210.250295	-5210.312855	-5210.3527084	-5210.5218034	-5210.1998481
kn13_014	-5210.4918486	-5210.249941	-5210.312424	-5210.3528726	-5210.5219050	-5210.2004859
kn13_023	-5210.4904601	-5210.248236	-5210.310635	-5210.3504417	-5210.5189325	-5210.1960705
**47**						
kn13_012	-389.6625870	-389.430669	-389.476447	-389.7053962	-389.67206072	**-389.5257114**
**48**						
kn13_006	-4820.8040892	-4820.793801	-4820.833484	-4820.6335342	-4820.8314527	**-4820.6872740**
**8d**						

kn13_013	-5210.5856109	-5210.340783	-5210.403184	-5210.4549339	-5210.6122602	**-5210.2961378**
kn13_011	-5210.5855788	-5210.341796	-5210.402634	-5210.4549318	-5210.6121604	-5210.2955501

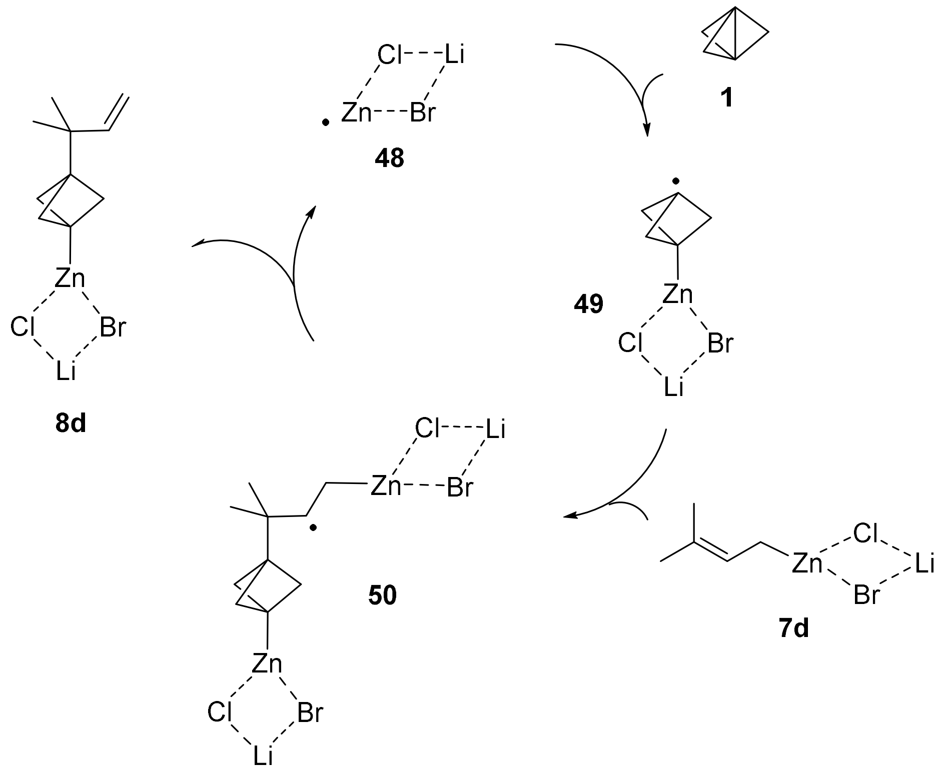

Figure S7. Reaction of prenylzinc bromide (**7d**) with [1.1.1]propellane (**1**) via a radical chain mechanism.

Table S4a. Total energies and enthalpies for the systems shown in Figure S7 (in Hartree).

**system**	ΔH_{298} SMD(THF)/ B2PLYP-D3/ def2TZVPP// [kJ/mol]	ΔG_{298}(qh,1M) SMD(THF)/ B2PLYP-D3/ def2TZVPP// [kJ/mol]
**Step 1: 48 + 1 > 49**	-20.3	+24.2
**Step 2:**	-91.4	-26.3

**49 + 7d >50**		
**Step 3:**<br>**50 > 48 + 8d**	+10.0	-43.2
**sum of steps**<br>**(7d + 1 > 8d)**	-101.7	-45.3

Table S4b. Total energies and enthalpies for the systems shown in Figure S7 (in Hartree).

**system**	E_{tot} B3LYP-D3/ def2SVP	H_{298} B3LYP-D3/ def2SVP	G_{298} B3LYP-D3/ def2SVP	$\langle S^2 \rangle$ B3LYP-D3/ def2SVP	G_{298} (qh) B3LYP-D3/ def2SVP	E_{tot} B2PLYP-D3/ def2TZVP// B3LYP-D3/ def2SVP	$\langle S^2 \rangle$ B2PLYP-D3/ def2TZVPP// B3LYP-D3/ def2SVP
**48**							
kn13_006	-4820.8040892	-4820.793801	-4820.833484	0.7511	-4820.833484	-5016.4976610	0.00
**1**							
kn13_002	-193.87235922	-193.774498	-193.805682	0.00	-193.805682	-193.9042207	0.00
kn13_005	-193.87237324	-193.774506	-193.803999	0.00	-193.803999	-193.9042284	0.00
**49**							
kn13_007	-5014.7046547	-5014.595760	-5014.650143	0.7562	-5014.646674	-5014.5559087	0.7631
**7d**							
kn13_009	-5016.6575135	-5016.514420	-5016.572439	0.00	-5016.569117	-5016.4976610	0.00
kn13_008	-5016.6573729	-5016.514302	-5016.572214	0.00	-5016.569054	-5016.4980160	0.00
kn13_004	-5016.6573728	-5016.514302	-5016.572214	-	-	-	-
kn13_001	-5016.6567449	-5016.513644	-5016.572378	0.00	-5016.568774	-5016.4975508	0.00
**50**							
kn13_010	-10031.4125535	-10031.158248	-10031.244208	0.7578	-10031.235813	-10031.0997167	0.7667
kn13_039	-10031.4125212	-10031.157294	-10031.248128	0.7581	-10031.236528	-10031.0996262	0.7674
kn13_018	-10031.4125210	-10031.157299	-10031.249558	0.7581	-10031.236533	-10031.0996262	0.7674
kn13_040	-10031.4125136	-10031.158256	-10031.244640	0.7579	-10031.235862	-10031.0996624	0.7669
kn13_017	-10031.4122760	-10031.157089	-10031.248437	0.7581	-10031.236324	-10031.0999184	0.7674
kn13_016	-10031.4122215	-10031.158074	-10031.244701	0.7579	-10031.235710	-10031.0998546	0.7670
**8d**							
kn13_013	-5210.5856109	-5210.340783	-5210.408077	0.00	-5210.403184	-5210.4549339	0.00
kn13_011	-5210.5855788	-5210.341796	-5210.406118	0.00	-5210.402634	-5210.4549318	0.00

Table S4c. Total energies and enthalpies for the systems shown in Figure S7 (in Hartree).

**system**	E_{tot} B3LYP-D3/ def2SVP	H_{298} B3LYP-D3/ def2SVP	G_{298}(qh) B3LYP-D3/ def2SVP (qh)	E_{tot} B2PLYP-D3/ def2TZVPP// B3LYP-D3/ def2SVP	E_{tot} SMD(THF)/ B3LYP-D3/ def2SVP	ΔG_{298}(qh,1M) SMD(THF)/ B2PLYP-D3/ def2TZVPP// B3LYP-D3/ def2SVP [kJ/mol]
**48**						
kn13_006	-4820.8040892	-4820.793801	-4820.833484	-4820.6335342	-4820.8314527	**-4820.6872740**
**1**						
kn13_002	-193.87235922	-193.774498	-193.805682	-193.9042207	-193.88523469	**-193.8474005**
kn13_005	-193.87237324	-193.774506	-193.803999	-193.9042284	-193.88523394	-193.8456964
**49**						
kn13_007	-5014.7046547	-5014.595760	-5014.646674	-5014.5559087	-5014.73520148	**-5014.5254563**
**7d**						
kn13_009	-5016.6575135	-5016.514420	-5016.569117	-5016.4976610	-5016.6817050	-5016.4304375
kn13_008	-5016.6573729	-5016.514302	-5016.569054	-5016.4980160	-5016.6821803	**-5016.4314860**
kn13_001	-5016.6567449	-5016.513644	-5016.568774	-5016.4975508	-5016.6810878	-5016.4309043
**50**						
kn13_010	-10031.4125535	-10031.158248	-10031.235813	-10031.0997167	-10031.4579035	-10030.9653077
kn13_039	-10031.4125212	-10031.157294	-10031.236528	-10031.0996262	-10031.4572714	-10030.9653647
kn13_040	-10031.4125136	-10031.158256	-10031.235862	-10031.0996624	-10031.4580250	-10030.9655037
kn13_017	-10031.4122760	-10031.157089	-10031.236324	-10031.0999184	-10031.4583017	**-10030.9669736**
kn13_016	-10031.4122215	-10031.158074	-10031.235710	-10031.0998546	-10031.4586582	-10030.9667613
**8d**						
kn13_013	-5210.5856109	-5210.340783	-5210.403184	-5210.4549339	-5210.6122602	**-5210.2961378**
kn13_011	-5210.5855788	-5210.341796	-5210.402634	-5210.4549318	-5210.6121604	-5210.2955501

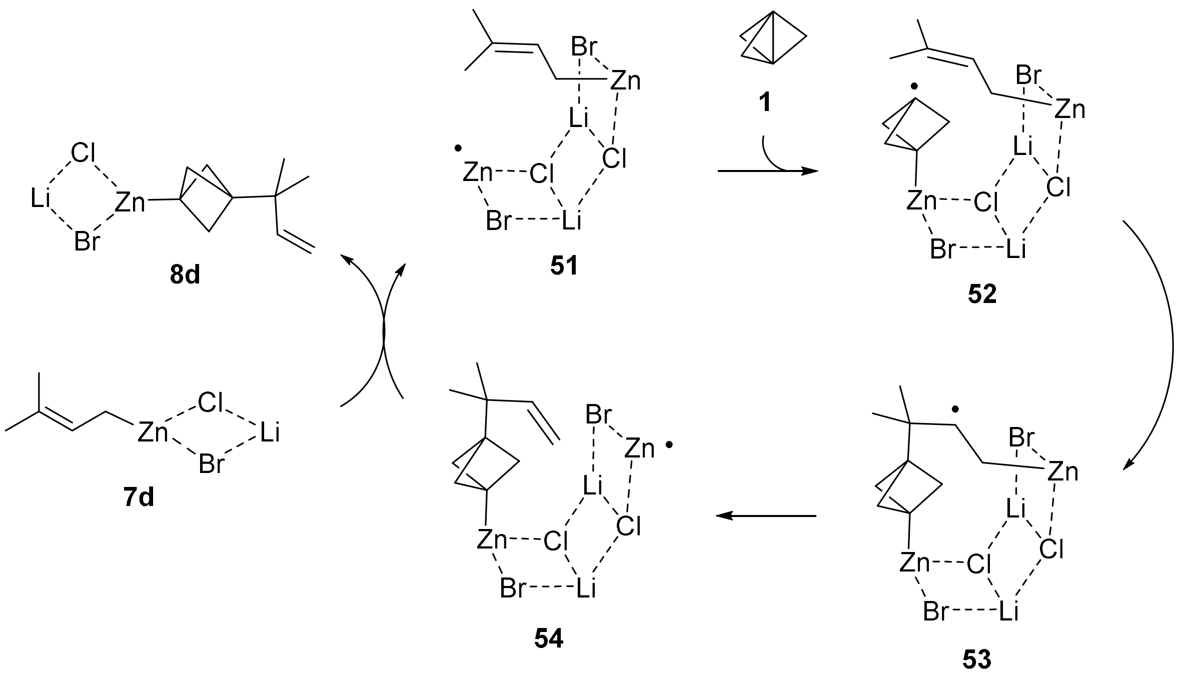

Figure S8. Reaction of prenylzinc bromide (**7d**) with [1.1.1]propellane (**1**) via a radical chain mechanism involving a cluster.

Table S5a. Total energies and enthalpies for the systems shown in Figure S8 (in Hartree).

**system**	ΔH_{298} SMD(**THF**)/ B2PLYP-D3/ def2TZVPP// [kJ/mol]	ΔG_{298}(qh,1M) SMD(**THF**)/ B2PLYP-D3/ def2TZVPP// [kJ/mol]
**Step 1: 51 + 1 > 52**	-28.4	+18.0
**Step 2: 52 > 53**	-42.2	-30.8
**Step 3: 53 > 54**	-28.6	-30.7
**Step 4: 54 + 7d > 51 + 8d**	-2.5	-1.7
**sum of steps (7d + 1 > 8d)**	-101.7	-45.3

Table S5b. Total energies and enthalpies for the systems shown in Figure S8 (in Hartree).

**system**	E_{tot} B3LYP-D3/ def2SVP	H_{298} B3LYP-D3/ def2SVP	G_{298} B3LYP-D3/ def2SVP	$<S^2>$ B3LYP-D3/ def2SVP	G_{298} (qh) B3LYP-D3/ def2SVP	E_{tot} B2PLYP-D3/ def2TZVPP// B3LYP-D3/ def2SVP	$<S^2>$ B2PLYP-D3/ def2TZVPP// B3LYP-D3/ def2SVP
**7d**							
kn13_009	-5016.6575135	-5016.514420	-5016.572439	0.00	-5016.569117	-5016.4976610	0.00
kn13_008	-5016.6573729	-5016.514302	-5016.572214	0.00	-5016.569054	-5016.4980160	0.00
kn13_004	-5016.6573728	-5016.514302	-5016.572214	0.00	-	-	-
kn13_001	-5016.6567449	-5016.513644	-5016.572378	0.00	-5016.568774	-5016.4975508	0.00
**1**							
kn13_002	-193.87235922	-193.774498	-193.805682	0.00	-193.805682	-193.9042207	0.00
kn13_005	-193.87237324	-193.774506	-193.803999	0.00	-193.803999	-193.9042284	0.00
**8d**							
kn13_013	-5210.5856109	-5210.340783	-5210.408077	0.00	-5210.403184	-5210.4549339	0.00
kn13_011	-5210.5855788	-5210.341796	-5210.406118	0.00	-5210.402634	-5210.4549318	0.00
**51**							
kn13_106	-9837.5360323	-9837.380757	-9837.456997	0.7510	-9837.452738	-9837.1873612	0.7519
kn13_127	-9837.5359207	-9837.380600	-9837.456611	0.7510	-9837.452594	-9837.1875931	0.7519
kn13_107	-9837.5354000	-9837.380096	-9837.458579	0.7510	-9837.452317	-9837.1876535	0.7519
kn13_126	-9837.5353226	-9837.380024	-9837.456684	0.7510	-9837.452210	-9837.1870391	0.7519
kn13_103	-9837.5352570	-9837.379918	-9837.458072	0.7510	-9837.452070	-9837.1879195	0.7519
kn13_104	-9837.5352570	-9837.379918	-9837.458073	0.7510	-	-	-
kn13_105	-9837.5313825	-9837.375987	-9837.451770	0.7510	-9837.447615	-9837.1834967	0.7518
**52**							
kn13_092	-10031.4390829	-10031.184185	-10031.272439	0.7611	-10031.266702	-10031.1148367	0.7852
kn13_090	-10031.4390827	-10031.184186	-10031.272438	0.7611	-	-	-
kn13_091	-10031.4385555	-10031.183784	-10031.272830	0.7619	-10031.266619	-10031.1138462	0.7885
kn13_112	-10031.4373972	-10031.183406	-10031.276740	0.7564	-10031.265361	-10031.1124650	0.7628
kn13_113	-10031.4373186	-10031.183352	-10031.276639	0.7564	-10031.265608	-10031.1118802	0.7629
kn13_025	-10031.4371231	-10031.182378	-10031.271254	0.7617	-10031.265056	-10031.1127045	0.7876
**53**							
kn13_028	-10031.4513468	-10031.196312	-10031.279104	0.7591	-10031.274659	-10031.1256547	0.7697

kn13_022	-10031.4489077	-10031.193429	-10031.276311	0.7598	-10031.271481	-10031.1222567	0.7716
kn13_020	-10031.4448730	-10031.189344	-10031.272328	0.7596	-10031.267704	-10031.1213740	0.7709
**54**							
kn13_089	-10031.4635772	-10031.206610	-10031.293936	0.7510	-10031.285848	-10031.1438322	0.7519
kn13_024	-10031.4593003	-10031.202430	-10031.288862	0.7509	-10031.281588	-10031.1411317	0.7518

Table S5c. Total energies and enthalpies for the systems shown in Figure S8 (in Hartree).

**system**	E_{tot} B3LYP-D3/ def2SVP	H_{298} B3LYP-D3/ def2SVP	G_{298}(qh) B3LYP-D3/ def2SVP (qh)	E_{tot} B2PLYP-D3/ def2TZVPP// B3LYP-D3/ def2SVP	E_{tot} SMD(THF)/ B3LYP-D3/ def2SVP	ΔG_{298}(qh,1M) SMD(THF)/ B2PLYP-D3/ def2TZVPP// B3LYP-D3/ def2SVP
**1**						
kn13_009	-5016.6575135	-5016.514420	-5016.569117	-5016.4976610	-5016.6817050	-5016.4304375
kn13_008	-5016.6573729	-5016.514302	-5016.569054	-5016.4980160	-5016.6821803	**-5016.4314860**
kn13_001	-5016.6567449	-5016.513644	-5016.568774	-5016.4975508	-5016.6810878	-5016.4309043
**1**						
kn13_002	-193.87235922	-193.774498	-193.805682	-193.9042207	-193.88523469	**-193.8474005**
kn13_005	-193.87237324	-193.774506	-193.803999	-193.9042284	-193.88523394	-193.8456964
**8d**						
kn13_013	-5210.5856109	-5210.340783	-5210.403184	-5210.4549339	-5210.6122602	**-5210.2961378**
kn13_011	-5210.5855788	-5210.341796	-5210.402634	-5210.4549318	-5210.6121604	-5210.2955501
**51**						
kn13_106	-9837.5360323	-9837.380757	-9837.452738	-9837.1873612	-9837.56568037	-9837.1306965
kn13_127	-9837.5359207	-9837.380600	-9837.452594	-9837.1875931	-9837.56442338	-9837.1297506
kn13_107	-9837.5354000	-9837.380096	-9837.452317	-9837.1876535	-9837.56452836	-9837.1306804
kn13_126	-9837.5353226	-9837.380024	-9837.452210	-9837.1870391	-9837.56416830	-9837.1297537
kn13_103	-9837.5352570	-9837.379918	-9837.452070	-9837.1879195	-9837.56524372	**-9837.1317007**
kn13_105	-9837.5313825	-9837.375987	-9837.447615	-9837.1834967	-9837.56351678	-9837.1288450
**52**						
kn13_092	-10031.4390829	-10031.184185	-10031.266702	-10031.1148367	-10031.4717550	-10030.9721094
kn13_091	-10031.4385555	-10031.183784	-10031.266619	-10031.1138462	-10031.4719252	**-10030.9722609**
kn13_112	-10031.4373972	-10031.183406	-10031.265361	-10031.1124650	-10031.4680725	-10030.9680856

kn13_113	-10031.4373186	-10031.183352	-10031.265608	-10031.1118802	-10031.4680725	-10030.9679050
kn13_025	-10031.4371231	-10031.182378	-10031.265056	-10031.1127045	-10031.4699381	-10030.9704339
**53**						
kn13_028	-10031.4513468	-10031.196312	-10031.274659	-10031.1256547	-10031.4894009	**-10030.9840025**
kn13_022	-10031.4489077	-10031.193429	-10031.271481	-10031.1222567	-10031.4870196	-10030.9799234
kn13_020	-10031.4448730	-10031.189344	-10031.267704	-10031.1213740	-10031.4831715	-10030.9794850
**54**						
kn13_089	-10031.4635772	-10031.206610	-10031.285848	-10031.1438322	-10031.4955658	-10030.9950731
kn13_024	-10031.4593003	-10031.202430	-10031.281588	-10031.1411317	-10031.4946135	**-10030.9957141**

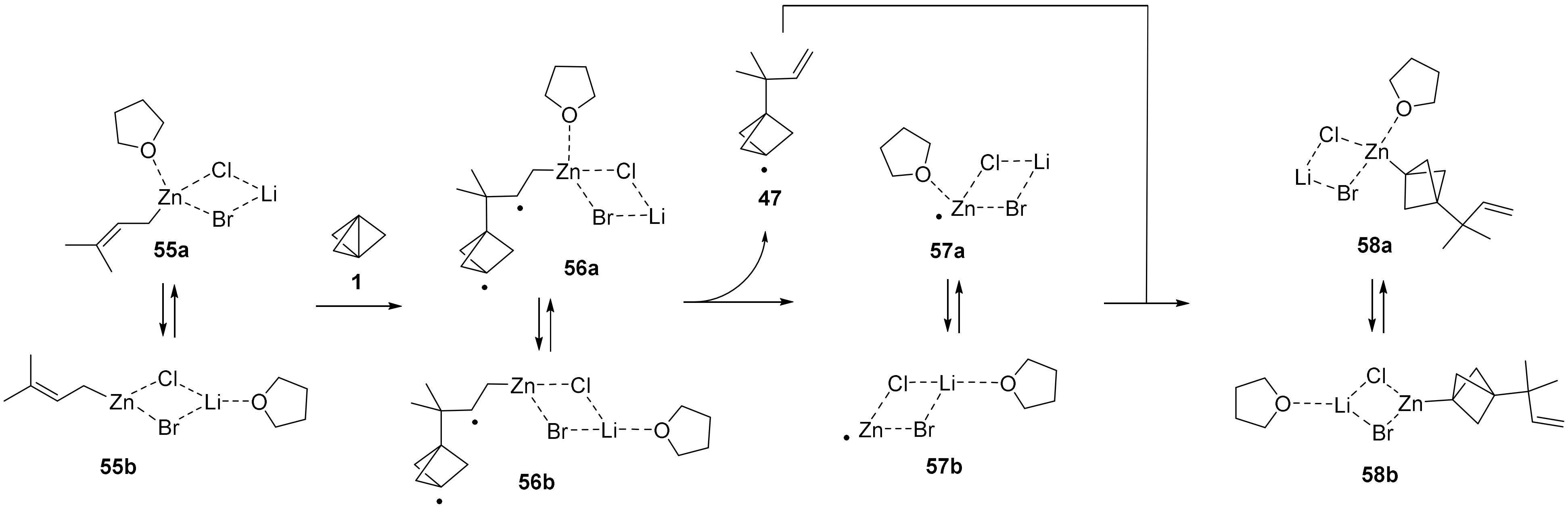

Figure S9. Radical reaction of prenylzinc bromide with [1.1.1]propellane (**1**) including one explicit THF solvent molecule.

Table S6a. Total energies and enthalpies for the systems shown in Figure S9 (in Hartree).

system	ΔH_{298} SMD(**THF**)/ B2PLYP-D3/ def2TZVPP// [kJ/mol]	ΔG_{298}(qh) SMD(**THF**)/ B2PLYP-D3/ def2TZVPP// [kJ/mol]
**55a + 1**	0.0	0.0
**56a**	+158.4	+215.0
**47 + 57a**	+197.3	+196.3
**58a**	-94.7	-40.0
**55a > 55b**	-25.6	-23.5
**55b + 1**	0.0	0.0
**56b**	+138.9	+193.3
**47 + 57b**	+176.0	+173.2
**58b**	-96.8	-43.5

Table S6b. Total energies and enthalpies for the systems shown in Figure S9 (in Hartree).

system	E_{tot} B3LYP-D3/ def2SVP	H_{298} B3LYP-D3/ def2SVP	G_{298} B3LYP-D3/ def2SVP	$\langle S^2 \rangle$ B3LYP-D3/ def2SVP	G_{298}(qh) B3LYP-D3/ def2SVP	E_{tot} B2PLYP-D3/ def2TZVP// B3LYP-D3/ def2SVP	$\langle S^2 \rangle$ B2PLYP-D3/ def2TZVPP// B3LYP-D3/ def2SVP
**55a**							
kn13_060	-5248.9831315	-5248.715080	-5248.786691	0.00	-5248.782716	-5248.8711815	0.00
kn13_027	-5248.9830006	-5248.715083	-5248.787465	0.00	-5248.782913	-5248.8711270	0.00
kn13_037	-5248.9828440	-5248.714958	-5248.787009	0.00	-5248.782750	-5248.8712516	0.00
kn13_041	-5248.9827442	-5248.714781	-5248.787140	0.00	-5248.782491	-5248.8712013	0.00
kn13_042	-5248.9827442	-5248.714781	-5248.787140	0.00	-	-	-
kn13_061	-5248.9827442	-5248.714781	-5248.787138	0.00	-	-	-
kn13_043	-5248.9823420	-5248.714183	-5248.785526	0.00	-5248.781635	-	-
kn13_035	-5248.9770430	-5248.709326	-5248.783682	0.00	-5248.777193	-	-
**55b**							
kn13_062	-5248.9946701	-5248.726218	-5248.797349	0.00	-5248.793099	-5248.8822551	0.00
kn13_046	-5248.9943181	-5248.725837	-5248.796800	0.00	-5248.792813	-5248.8824015	0.00
kn13_044	-5248.9943180	-	-	-	-	-	-
kn13_038	-5248.9910619	-5248.722834	-5248.794589	0.00	-5248.789665	-5248.8791425	0.00
kn13_045	-5248.9907218	-5248.722392	-5248.793733	0.00	-5248.788992	-5248.8781097	0.00

kn13_026	-5248.9905310	-5248.722443	-5248.794697	0.00	-5248.789508	-5248.8787039	0.00
kn13_036	-5248.9887542	-5248.720742	-5248.796740	0.00	-5248.788368	-5248.8781256	0.00
**1**							
kn13_005	-193.8723732	-193.774506	-193.803999	0.00	-193.805682	-193.9042207	0.00
kn13_002	-193.8723592	-193.774498	-193.805682	0.00	-193.803999	-193.9042284	0.00
**56a**							
kn13_053	-5442.8182123	-5442.451325	-5442.530139	0.8547	-5442.525663	-5442.7242285	0.9728
kn13_063	-5442.8180545	-5442.451108	-5442.529629	0.8576	-5442.525376	-5442.7242433	0.9737
kn13_047	-5442.8180068	-5442.451259	-5442.530355	0.8623	-5442.525677	-5442.7243900	0.9747
kn13_055	-5442.8174230	-5442.450578	-5442.529952	0.8584	-5442.525097	-5442.7239807	0.9734
kn13_059	-5442.8172243	-5442.450264	-5442.531323	0.8452	-5442.524958	-	-
kn13_054	-5442.8153662	-5442.448638	-5442.529542	0.8759	-5442.523534	-	-
kn13_058	-5442.8123383	-5442.445572	-5442.526305	0.9039	-5442.520453	-	-
**56b**							
kn13_064	-5442.8352743	-5442.467831	-5442.547205	0.8222	-5442.542267	-5442.7425286	0.9607
kn13_048	-5442.8352018	-5442.467922	-5442.548142	0.8212	-5442.542421	-5442.7421672	0.9608
kn13_032	-5442.8351673	-5442.467924	-5442.548751	0.8288	-5442.542539	-5442.7419256	0.9636
kn13_056	-5442.8288949	-5442.461726	-5442.544567	0.8698	-5442.536482	-5442.7365069	0.9703
kn13_057	-5442.8286805	-5442.461447	-5442.544050	0.8717	-5442.536186	-5442.7366042	0.9710
kn13_052	-5442.8267653	-5442.459623	-5442.540296	0.9101	-5442.534314	-5442.7355927	0.9861
kn13_051	-5442.8265647	-5442.459531	-5442.541231	0.9088	-5442.534360	-	-
kn13_050	-5442.8242542	-5442.456778	-5442.538058	1.0027	-5442.531307	-	-
kn13_049	-5442.8235884	-5442.456201	-5442.538009	0.9996	-5442.530818	-	-
**47**							
kn13_012	-389.6625870	-389.430669	-389.477303	0.7525	-389.476447	-389.7053962	0.7551
**57a**							
kn13_034	-5053.1213217	-5052.986121	-5053.041332	0.7510	-5053.039576	-5052.9980774	0.7518
kn13_067	-5053.1212391	-5052.986018	-5053.040996	0.7510	-5053.039435	-5052.9976731	0.7518
kn13_066	-5053.1209993	-5052.985781	-5053.040752	0.7510	-5053.039257	-5052.9976452	0.7518
kn13_065	-5053.1208822	-5052.985762	-5053.042323	0.7510	-5053.039331	-5052.9979986	0.7518
**57b**							
kn13_070	-5053.1351681	-5052.999744	-5053.056320	0.7512	-5053.052599	-5053.0128081	0.7523
kn13_068	-5053.1351681	-5052.999744	-5053.056320	0.7512	-	-	-
kn13_033	-5053.1342492	-5052.998957	-5053.056252	0.7512	-5053.052381	-5053.0134510	0.7523

kn13_069	-5053.1342492	-5052.998957	-5053.056252	0.7512	-	-	-
**58a**							
kn13_072	-5442.9080571	-5442.538542	-5442.621054	0.00	-5442.613531	-5442.8244587	0.00
kn13_073	-5442.9079957	-5442.538590	-5442.620865	0.00	-5442.613712	-5442.8251357	0.00
kn13_074	-5442.9079957	-5442.538590	-5442.620865	0.00	-5442.613712	-	-
kn13_071	-5442.9079956	-5442.538590	-5442.620868	0.00	-5442.613712	-	-
kn13_031	-5442.9078824	-5442.538359	-5442.620518	0.00	-5442.613297	-5442.8250127	0.00
**58b**							
kn13_077	-5442.9199336	-5442.549928	-5442.629466	0.00	-5442.623731	-5442.8358706	0.00
kn13_078	-5442.9196890	-5442.549707	-5442.629776	0.00	-5442.623611	-5442.8356435	0.00
kn13_075	-5442.9166914	-5442.546919	-5442.631267	0.00	-5442.621728	-5442.8352407	0.00
kn13_076	-5442.9166862	-5442.547004	-5442.632688	0.00	-5442.621921	-5442.8352062	0.00
kn13_030	-5442.9166539	-5442.546907	-5442.631349	0.00	-5442.621707	-5442.8352015	0.00

Table S6c. Total energies and enthalpies for the systems shown in Figure S9 (in Hartree).

**system**	E_{tot} B3LYP-D3/ def2SVP	H_{298} B3LYP-D3/ def2SVP	G_{298}(qh) B3LYP-D3/ def2SVP	E_{tot} B2PLYP-D3/ def2TZVP// B3LYP-D3/ def2SVP	E_{tot} SMD(THF)/ B3LYP-D3/ def2SVP	ΔG_{298}(qh,1M) SMD(THF)/ B2PLYP-D3/ def2TZVPP// B3LYP-D3/ def2SVP
**55a**						
kn13_060	-5248.9831315	-5248.715080	-5248.782716	-5248.8711815	-5249.0049712	-5248.6895872
kn13_027	-5248.9830006	-5248.715083	-5248.782913	-5248.8711270	-5249.0044741	-5248.6894944
kn13_037	-5248.9828440	-5248.714958	-5248.782750	-5248.8712516	-5249.0046552	**-5248.6899503**
kn13_041	-5248.9827442	-5248.714781	-5248.782491	-5248.8712013	-5249.0043873	-5248.6895727
**55b**						
kn13_062	-5248.9946701	-5248.726218	-5248.793099	-5248.8822551	-5249.0156537	-5248.6986491
kn13_046	-5248.9943181	-5248.725837	-5248.792813	-5248.8824015	-5249.0153295	**-5248.6988893**
kn13_038	-5248.9910619	-5248.722834	-5248.789665	-5248.8791425	-5249.0140124	-5248.6976776
kn13_045	-5248.9907218	-5248.722392	-5248.788992	-5248.8781097	-5249.0137079	-5248.6963475
kn13_026	-5248.9905310	-5248.722443	-5248.789508	-5248.8787039	-5249.0132180	-5248.6973494
kn13_036	-5248.9887542	-5248.720742	-5248.788368	-5248.8781256	-5249.0117253	-5248.6976920
**1**						
kn13_005	-193.8723732	-193.774506	-193.805682	-193.9042207	-193.88523469	**-193.8474005**
kn13_002	-193.8723592	-193.774498	-193.803999	-193.9042284	-193.88523394	-193.8456964

**56a**						
kn13_053	-5442.8182123	-5442.451325	-5442.525663	-5442.7242285	-5442.8437415	-5442.4541899
kn13_063	-5442.8180545	-5442.451108	-5442.525376	-5442.7242433	-5442.8435904	-5442.4540822
kn13_047	-5442.8180068	-5442.451259	-5442.525677	-5442.7243900	-5442.8444454	**-5442.4554803**
kn13_055	-5442.8174230	-5442.450578	-5442.525097	-5442.7239807	-5442.8434984	-5442.4547116
**56b**						
kn13_064	-5442.8352743	-5442.467831	-5442.542267	-5442.7425286	-5442.8614470	**-5442.4726755**
kn13_048	-5442.8352018	-5442.467922	-5442.542421	-5442.7421672	-5442.8613675	-5442.4725336
kn13_032	-5442.8351673	-5442.467924	-5442.542539	-5442.7419256	-5442.8606535	-5442.4717650
kn13_056	-5442.8288949	-5442.461726	-5442.536482	-5442.7365069	-5442.8572188	-5442.4693994
kn13_057	-5442.8286805	-5442.461447	-5442.536186	-5442.7366042	-5442.8573462	-5442.4697569
kn13_052	-5442.8267653	-5442.459623	-5442.534314	-5442.7355927	-5442.8552141	-5442.4685717
**47**						
kn13_012	-389.6625870	-389.430669	-389.476447	-389.7053962	-389.6720607	**-389.5257114**
**57a**						
kn13_034	-5053.1213217	-5052.986121	-5053.039576	-5052.9980774	-5053.1445312	-5052.9365227
kn13_067	-5053.1212391	-5052.986018	-5053.039435	-5052.9976731	-5053.1442977	-5052.9359091
kn13_066	-5053.1209993	-5052.985781	-5053.039257	-5052.9976452	-5053.1442483	-5052.9361334
kn13_065	-5053.1208822	-5052.985762	-5053.039331	-5052.9979986	-5053.1443221	**-5052.9368688**
**57b**						
kn13_070	-5053.1351681	-5052.999744	-5053.052599	-5053.0128081	-5053.1601845	-5052.9522369
kn13_033	-5053.1342492	-5052.998957	-5053.052381	-5053.0134510	-5053.1602775	**-5052.9545926**
**58a**						
kn13_072	-5442.9080571	-5442.538542	-5442.613531	-5442.8244587	-5442.9328037	-5442.5516607
kn13_073	-5442.9079957	-5442.538590	-5442.613712	-5442.8251357	-5442.9327654	**-5442.5526032**
kn13_031	-5442.9078824	-5442.538359	-5442.613297	-5442.8250127	-5442.9325815	-5442.5521079
**58b**						
kn13_077	-5442.9199336	-5442.549928	-5442.623731	-5442.8358706	-5442.9446857	-5442.5614016
kn13_078	-5442.9196890	-5442.549707	-5442.623611	-5442.8356435	-5442.9446937	-5442.5615517
kn13_075	-5442.9166914	-5442.546919	-5442.621728	-5442.8352407	-5442.9422601	-5442.5628275
kn13_076	-5442.9166862	-5442.547004	-5442.621921	-5442.8352062	-5442.9421142	**-5442.5628505**
kn13_030	-5442.9166539	-5442.546907	-5442.621707	-5442.8352015	-5442.9421679	-5442.5627501

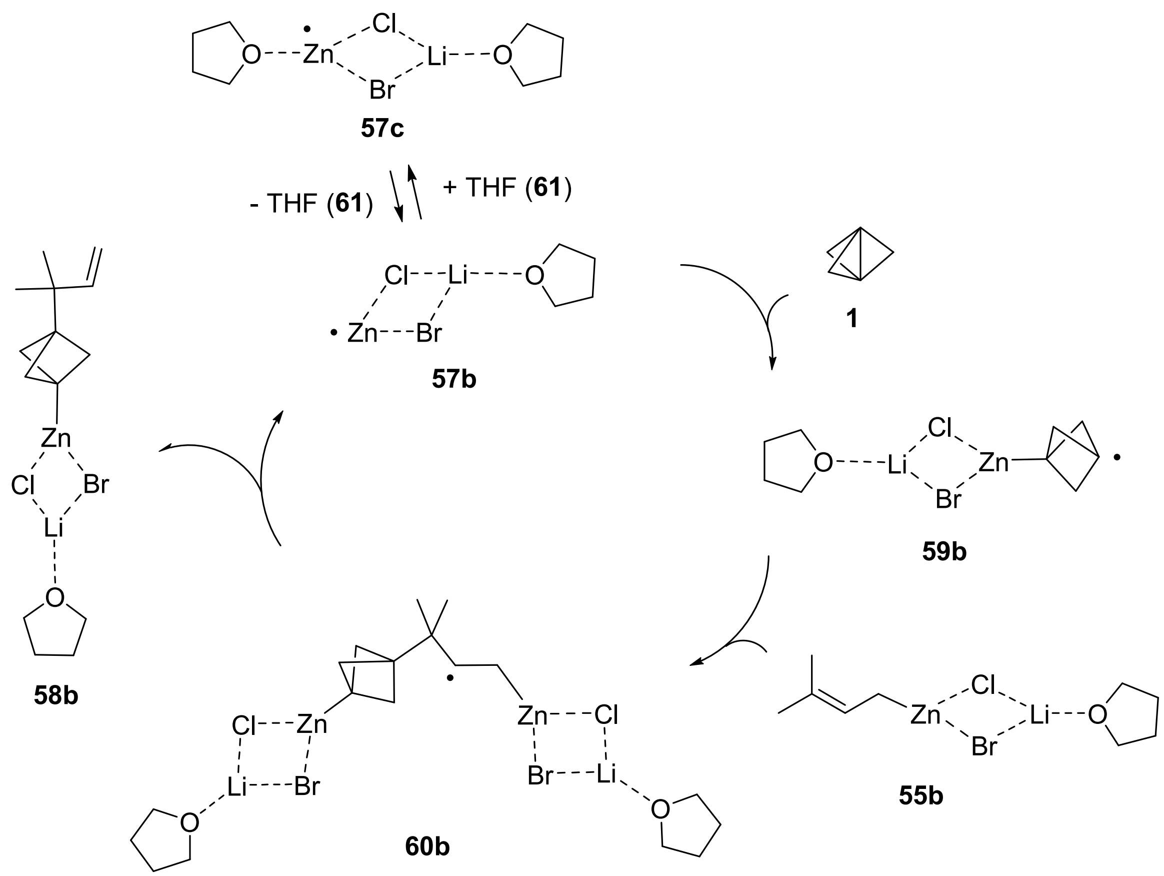

Figure S10. Radical chain reaction of the allylic zinc reagent **55b** with [1.1.1]propellane (**1**) including one explicit THF solvent molecule.

Table S7a. Reaction energies for the systems shown in Figure S10 (in Hartree).

**system**	ΔH_{298}<br>SMD(**THF**)/<br>B2PLYP-D3/<br>def2TZVPP//<br>[kJ/mol]	ΔG_{298}(qh,1M)<br>SMD(**THF**)/<br>B2PLYP-D3/<br>def2TZVPP//<br>[kJ/mol]
**Equ.**<br>**57c > 61 + 57b**	+27.4	-19.5
**Step 1:**<br>**1 + 57b > 59b**	-23.5	+23.7
**Step 2:**<br>**59b + 55b > 60b**	-116.7	-50.3
**Step 3:**<br>**60b > 58b + 57b**	+43.3	-16.9
**sum of steps**<br>**(1 + 55b > 58b)**	-96.9	-43.5

Table S7b. Total energies and enthalpies for the systems shown in Figure S10 (in Hartree).

**system**	E_{tot}<br>B3LYP-D3/<br>def2SVP	H_{298}<br>B3LYP-D3/<br>def2SVP	G_{298}<br>B3LYP-D3/<br>def2SVP	$\langle S^2 \rangle$<br>B3LYP-D3/<br>def2SVP	G_{298}(qh)<br>B3LYP-D3/<br>def2SVP	E_{tot}<br>B2PLYP-D3/<br>def2TZVP//<br>B3LYP-D3/<br>def2SVP	$\langle S^2 \rangle$<br>B2PLYP-D3/<br>def2TZVPP//<br>B3LYP-D3/<br>def2SVP
**55b**							
kn13_062	-5248.9946701	-5248.726218	-5248.797349	0.00	-5248.793099	-5248.8822551	0.00
kn13_046	-5248.9943181	-5248.725837	-5248.796800	0.00	-5248.792813	-5248.8824015	0.00
kn13_044	-5248.9943180	-	-	-	-	-	-
kn13_038	-5248.9910619	-5248.722834	-5248.794589	0.00	-5248.789665	-5248.8791425	0.00
kn13_045	-5248.9907218	-5248.722392	-5248.793733	0.00	-5248.788992	-5248.8781097	0.00
kn13_026	-5248.9905310	-5248.722443	-5248.794697	0.00	-5248.789508	-5248.8787039	0.00
kn13_036	-5248.9887542	-5248.720742	-5248.796740	0.00	-5248.788368	-5248.8781256	0.00
**1**							
kn13_005	-193.8723732	-193.774506	-193.803999	0.00	-193.805682	-193.9042207	0.00
kn13_002	-193.8723592	-193.774498	-193.805682	0.00	-193.803999	-193.9042284	0.00
**57b**							
kn13_070	-5053.1351681	-5052.999744	-5053.056320	0.7512	-5053.052599	-5053.0128081	0.7523
kn13_068	-5053.1351681	-5052.999744	-5053.056320	0.7512	-	-	-

kn13_033	-5053.1342492	-5052.998957	-5053.056252	0.7512	-5053.052381	-5053.0134510	0.7523
kn13_069	-5053.1342492	-5052.998957	-5053.056252	0.7512	-	-	-
**57c**							
kn13_086	-5285.4524346	-5285.192172	-5285.261675	0.7510	-5285.257488	-5285.377114	0.7519
kn13_096	-5285.4524346	-5285.192172	-5285.261675	0.7510	-5285.257488	-	-
kn13_087	-5285.4523493	-5285.192012	-5285.261767	0.7510	-5285.258098	-5285.3781864	0.7519
kn13_088	-5285.4522980	-5285.192199	-5285.261585	0.7510	-5285.257580	-5285.3769100	0.7519
kn13_095	-5285.4522443	-5285.192013	-5285.262277	0.7510	-5285.258210	-5285.3780758	0.7519
kn13_093	-5285.4518137	-5285.191665	-5285.262784	0.7510	-5285.258056	-5285.3778422	0.7519
kn13_094	-5285.4511173	-5285.190852	-5285.261922	0.7510	-5285.257069	-5285.3774787	0.7518
kn13_108	-5285.4505726	-5285.190479	-5285.260948	0.7510	-5285.256642	-5285.3768677	0.7518
**58b**							
kn13_077	-5442.9199336	-5442.549928	-5442.629466	0.00	-5442.623731	-5442.8358706	0.00
kn13_078	-5442.9196890	-5442.549707	-5442.629776	0.00	-5442.623611	-5442.8356435	0.00
kn13_075	-5442.9166914	-5442.546919	-5442.631267	0.00	-5442.621728	-5442.8352407	0.00
kn13_076	-5442.9166862	-5442.547004	-5442.632688	0.00	-5442.621921	-5442.8352062	0.00
kn13_030	-5442.9166539	-5442.546907	-5442.631349	0.00	-5442.621707	-5442.8352015	0.00
**59b**							
kn13_079	-5247.0383217	-5246.804275	-5246.871711	0.7551	-5246.866930	-5246.936414	0.7607
kn13_080	-5247.0383217	-5246.804275	-5246.871711	0.7551	-5246.866930	-	-
kn13_081	-5247.0358079	-5246.801899	-5246.873044	0.7551	-5246.865538	-5246.9364388	0.7608
kn13_082	-5247.0357732	-5246.801895	-5246.874147	0.7551	-5246.865553	-5246.9364224	0.7608
kn13_083	-5247.0357710	-	-	-	-	-	-
**61**							
kn13_084	-232.28518104	-232.162929	-232.197875	0.00	-232.196466	-232.3426885	0.00
kn13_085	-232.28590059	-232.163823	-232.197673	0.00	-232.197444	-232.3428943	0.00
**60b**							
kn13_111	-10496.0997375	-10495.593893	-10495.705959	0.7565	-10495.695279	-10495.8756169	0.7636
kn13_109	-10496.0984338	-10495.592443	-10495.703714	0.7566	-10495.694269	-	-
kn13_110	-10496.0984333	-10495.592444	-10495.703728	0.7566	-10495.694273	-10495.8760979	0.7635
kn13_114	-10496.0972934	-10495.591468	-10495.705275	0.7567	-10495.693770	-10495.8752033	0.7640
kn13_102	-10496.0825707	-10495.576784	-10495.692804	0.7575	-10495.678756	-10495.8644445	0.7660
kn13_097	-10496.0805365	-10495.575102	-10495.691484	0.7574	-10495.677244	-10495.8633397	0.7661
kn13_101	-10496.0792827	-10495.573682	-10495.694942	0.7586	-10495.676712	-10495.8638378	0.7696
kn13_098	-10496.0770840	-10495.571773	-10495.692999	0.7575	-10495.674846	-10495.8623509	0.7663

kn13_099	-10496.0770169	-10495.571880	-10495.692761	0.7575	-10495.675082	-10495.8623260	0.7664
kn13_100	-10496.0769800	-10495.571815	-10495.693523	0.7576	-10495.674981	-10495.8622572	0.7664

Table S7c. Total energies and enthalpies for the systems shown in Figure S10 (in Hartree).

**system**	E_{tot} B3LYP-D3/ def2SVP	H_{298} B3LYP-D3/ def2SVP	G_{298}(qh) B3LYP-D3/ def2SVP	E_{tot} B2PLYP-D3/ def2TZVP// B3LYP-D3/ def2SVP	E_{tot} SMD(THF)/ B3LYP-D3/ def2SVP	ΔG_{298}(qh,1M) SMD(THF)/ B2PLYP-D3/ def2TZVPP// B3LYP-D3/ def2SVP
**55b**						
kn13_062	-5248.9946701	-5248.726218	-5248.793099	-5248.8822551	-5249.0156537	-5248.6986491
kn13_046	-5248.9943181	-5248.725837	-5248.792813	-5248.8824015	-5249.0153295	**-5248.6988893**
kn13_038	-5248.9910619	-5248.722834	-5248.789665	-5248.8791425	-5249.0140124	-5248.6976776
kn13_045	-5248.9907218	-5248.722392	-5248.788992	-5248.8781097	-5249.0137079	-5248.6963475
kn13_026	-5248.9905310	-5248.722443	-5248.789508	-5248.8787039	-5249.0132180	-5248.6973494
kn13_036	-5248.9887542	-5248.720742	-5248.788368	-5248.8781256	-5249.0117253	-5248.6976920
**1**						
kn13_005	-193.8723732	-193.774506	-193.805682	-193.9042207	-193.88523469	**-193.8474005**
kn13_002	-193.8723592	-193.774498	-193.803999	-193.9042284	-193.88523394	-193.8456964
**57b**						
kn13_070	-5053.1351681	-5052.999744	-5053.052599	-5053.0128081	-5053.1601845	-5052.9522369
kn13_033	-5053.1342492	-5052.998957	-5053.052381	-5053.0134510	-5053.1602775	**-5052.9545926**
**57c**						
kn13_086	-5285.4524346	-5285.192172	-5285.257488	-5285.377114	-5285.4731077	-5285.1998220
kn13_087	-5285.4523493	-5285.192012	-5285.258098	-5285.3781864	-5285.47518840	-5285.2037557
kn13_088	-5285.4522980	-5285.192199	-5285.257580	-5285.3769100	-5285.47313633	-5285.2000118
kn13_095	-5285.4522443	-5285.192013	-5285.258210	-5285.3780758	-5285.47559374	-5285.2043724
kn13_093	-5285.4518137	-5285.191665	-5285.258056	-5285.3778422	-5285.47529170	-5285.2045440
kn13_094	-5285.4511173	-5285.190852	-5285.257069	-5285.3774787	-5285.47427075	-5285.2035654
kn13_108	-5285.4505726	-5285.190479	-5285.256642	-5285.3768677	-5285.47525669	**-5285.2046027**
**58b**						
kn13_077	-5442.9199336	-5442.549928	-5442.623731	-5442.8358706	-5442.9446857	-5442.5614016
kn13_078	-5442.9196890	-5442.549707	-5442.623611	-5442.8356435	-5442.9446937	-5442.5615517
kn13_075	-5442.9166914	-5442.546919	-5442.621728	-5442.8352407	-5442.9422601	-5442.5628275

kn13_076	-5442.9166862	-5442.547004	-5442.621921	-5442.8352062	-5442.9421142	**-5442.5628505**
kn13_030	-5442.9166539	-5442.546907	-5442.621707	-5442.8352015	-5442.9421679	-5442.5627501
**59b**						
kn13_079	-5247.0383217	-5246.804275	-5246.866930	-5246.936414	-5247.06701339	-5246.7906955
kn13_081	-5247.0358079	-5246.801899	-5246.865538	-5246.9364388	-5247.06564249	**-5246.7929850**
kn13_082	-5247.0357732	-5246.801895	-5246.865553	-5246.9364224	-5247.06541779	-5246.7928283
**61**						
kn13_084	-232.28518104	-232.162929	-232.196466	-232.3426885	-232.291007918	-232.2567818
kn13_085	-232.28590059	-232.163823	-232.197444	-232.3428943	-232.291909237	**-232.2574279**
**60b**						
kn13_111	-10496.0997375	-10495.593893	-10495.695279	-10495.8756169	-10496.1399818	-10495.5083842
kn13_110	-10496.0984333	-10495.592444	-10495.694273	-10495.8760979	-10496.1362879	-10495.5067737
kn13_114	-10496.0972934	-10495.591468	-10495.693770	-10495.8752033	-10496.1396495	**-10495.5110175**
kn13_102	-10496.0825707	-10495.576784	-10495.678756	-10495.8644445	-10496.1251774	-10495.5002180
kn13_097	-10496.0805365	-10495.575102	-10495.677244	-10495.8633397	-10496.1231295	-10495.4996217
kn13_101	-10496.0792827	-10495.573682	-10495.676712	-10495.8638378	-10496.1230554	-10495.5020213
kn13_098	-10496.0770840	-10495.571773	-10495.674846	-10495.8623509	-10496.1207865	-10495.5007969
kn13_099	-10496.0770169	-10495.571880	-10495.675082	-10495.8623260	-10496.1201571	-10495.5005128
kn13_100	-10496.0769800	-10495.571815	-10495.674981	-10495.8622572	-10496.1203103	-10495.5005700

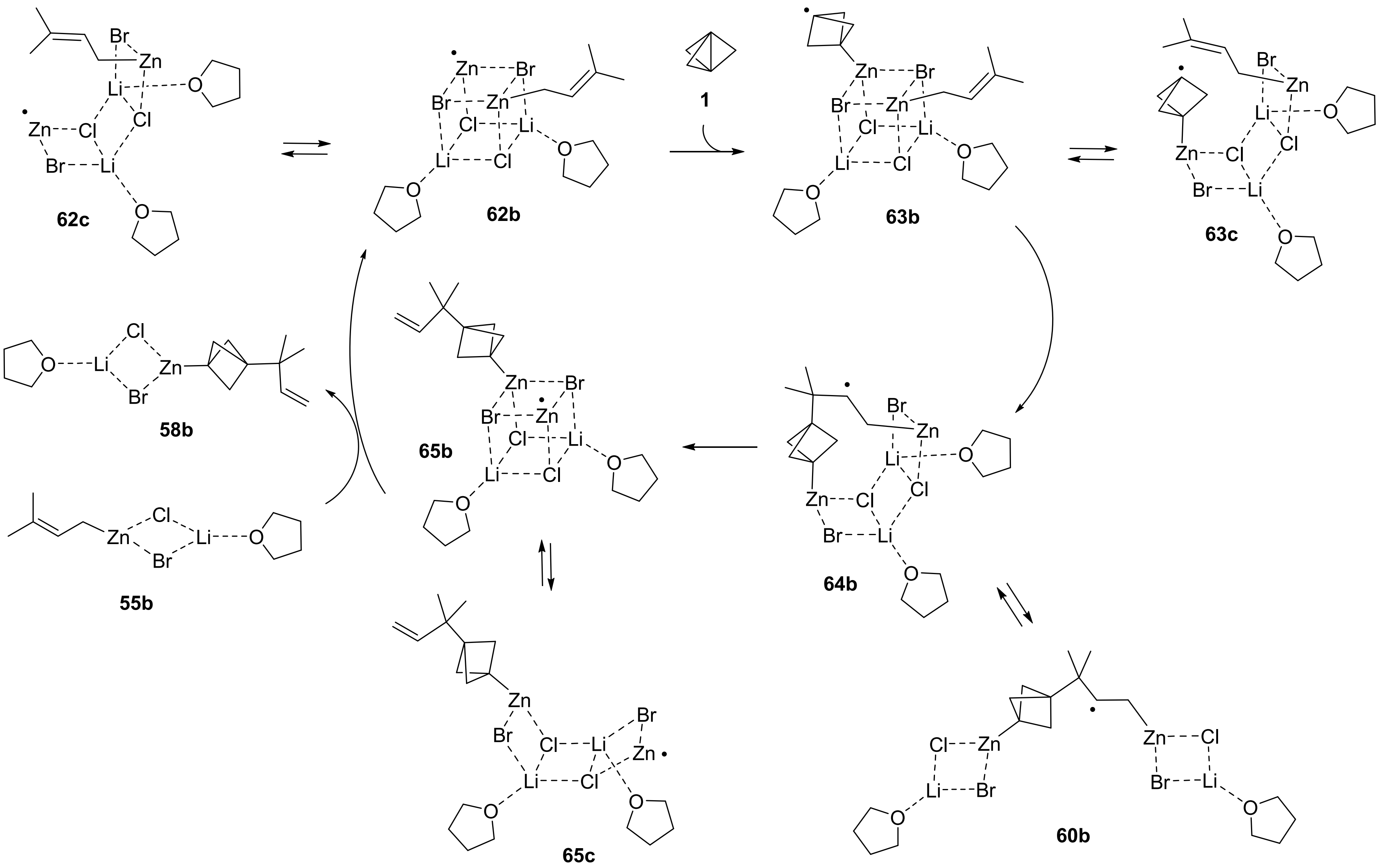

Figure S11. Radical chain reaction of the allylzinc reagent **55b** with [1.1.1]propellane **2** including explicit THF solvent molecules.

Table S8a. Reaction energies for the systems shown in Figure S11 (in Hartree).

**system**	ΔH_{298} SMD(**THF**)/ B2PLYP-D3/ def2TZVPP// [kJ/mol]	ΔG_{298}(qh,1M) SMD(**THF**)/ B2PLYP-D3/ def2TZVPP// [kJ/mol]
**Eq. 60b > 64b**	-16.1	-18.3
**Eq. 65c > 65b**	-13.8	-15.1
**Step 1: 1 + 62b > 63b**	-29.4	+20.3
**Step 2: 63b > 64b**	-36.0	-27.5
**Step 3: 64b > 65b**	-26.9	-29.8
**Step 4: 65b + 55b > 58b + 62b**	-4.5	-6.4
**sum of steps (1 + 55b > 58b)**	-96.8	-43.4

Table S8b. Total energies and enthalpies for the systems shown in Figure S11 (in Hartree).

**system**	E_{tot} B3LYP-D3/ def2SVP	H_{298} B3LYP-D3/ def2SVP	G_{298} B3LYP-D3/ def2SVP	$<S^2>$ B3LYP-D3/ def2SVP	G_{298}(qh) B3LYP-D3/ def2SVP	E_{tot} B2PLYP-D3/ def2TZVP// B3LYP-D3/ def2SVP	$<S^2>$ B2PLYP-D3/ def2TZVPP// B3LYP-D3/ def2SVP
**55b**							
kn13_062	-5248.9946701	-5248.726218	-5248.797349	0.00	-5248.793099	-5248.8822551	0.00
kn13_046	-5248.9943181	-5248.725837	-5248.796800	0.00	-5248.792813	**-5248.8824015**	0.00
kn13_044	-5248.9943180	-	-	-	-	-	-
kn13_038	-5248.9910619	-5248.722834	-5248.794589	0.00	-5248.789665	-5248.8791425	0.00
kn13_045	-5248.9907218	-5248.722392	-5248.793733	0.00	-5248.788992	-5248.8781097	0.00
kn13_026	-5248.9905310	-5248.722443	-5248.794697	0.00	-5248.789508	-5248.8787039	0.00
kn13_036	-5248.9887542	-5248.720742	-5248.796740	0.00	-5248.788368	-5248.8781256	0.00
**1**							
kn13_005	-193.8723732	-193.774506	-193.803999	0.00	-193.805682	-193.9042207	0.00
kn13_002	-193.8723592	-193.774498	-193.805682	0.00	-193.803999	**-193.9042284**	0.00

**58b**							
kn13_077	-5442.9199336	-5442.549928	-5442.629466	0.00	-5442.623731	**-5442.8358706**	0.00
kn13_078	-5442.9196890	-5442.549707	-5442.629776	0.00	-5442.623611	-5442.8356435	0.00
kn13_075	-5442.9166914	-5442.546919	-5442.631267	0.00	-5442.621728	-5442.8352407	0.00
kn13_076	-5442.9166862	-5442.547004	-5442.632688	0.00	-5442.621921	-5442.8352062	0.00
kn13_030	-5442.9166539	-5442.546907	-5442.631349	0.00	-5442.621707	-5442.8352015	0.00
**60b**							
kn13_111	-10496.0997375	-10495.593893	-10495.705959	0.7565	-10495.695279	-10495.8756169	0.7636
kn13_109	-10496.0984338	-10495.592443	-10495.703714	0.7566	-10495.694269	-	-
kn13_110	-10496.0984333	-10495.592444	-10495.703728	0.7566	-10495.694273	**-10495.8760979**	0.7635
kn13_114	-10496.0972934	-10495.591468	-10495.705275	0.7567	-10495.693770	-10495.8752033	0.7640
kn13_102	-10496.0825707	-10495.576784	-10495.692804	0.7575	-10495.678756	-10495.8644445	0.7660
kn13_097	-10496.0805365	-10495.575102	-10495.691484	0.7574	-10495.677244	-10495.8633397	0.7661
kn13_101	-10496.0792827	-10495.573682	-10495.694942	0.7586	-10495.676712	-10495.8638378	0.7696
kn13_098	-10496.0770840	-10495.571773	-10495.692999	0.7575	-10495.674846	-10495.8623509	0.7663
kn13_099	-10496.0770169	-10495.571880	-10495.692761	0.7575	-10495.675082	-10495.8623260	0.7664
kn13_100	-10496.0769800	-10495.571815	-10495.693523	0.7576	-10495.674981	-10495.8622572	0.7664
**62b**							
kn13_134 (from kn13_117)	-10302.1998505	-10301.793972	-10301.899259	0.7510	-10301.890463	-10301.9509943	0.7519
kn13_165 (from kn13_134)	-10302.1997837	-10301.794102	-10301.899084	0.7510	-10301.890619	**-10301.951075**	0.7519
kn13_131 (from kn13_115)	-10302.1992152	-10301.793268	-10301.898827	0.7510	-10301.889679	-10301.9505531	0.7519
kn13_116 (from kn13_107)	-10302.1986391	-10301.793190	-10301.900645	0.7510	-10301.890398	-10301.9498011	0.7520
kn13_132 (from kn13_115)	-10302.1985914	-10301.793171	-10301.900659	0.7510	-10301.890577	-10301.9506078	0.7520
kn13_133 (from kn13_116)	-10302.1985855	-10301.793133	-10301.899722	0.7510	-10301.890295	-10301.9502018	0.7520
kn13_157 (from	-10302.1980690	-10301.792472	-10301.899107	0.7510	-10301.889617	-10301.9504959	0.7520

kn13_120)							
kn13_129 (from kn13_116)	-10302.1978781	-10301.792410	-10301.898921	0.7510	-10301.889707	-10301.9482851	0.7520
kn13_161 (from kn13_121)	-10302.1973917	-10301.792881	-10301.898879	0.7510	-10301.888878	-10301.9494329	0.7520
kn13_136 (from kn13_127)	-10302.1962515	-10301.790968	-10301.899056	0.7510	-10301.888491	-10301.9468741	0.7520
kn13_135 (from kn13_126)	-10302.1961726	-10301.790894	-10301.899254	0.7510	-10301.888277	-10301.9467181	0.7520
kn13_115 (from kn13_106)	-10302.1950996	-10301.789671	-10301.896983	0.7510	-10301.886894	-	-
kn13_117 (from kn13_103)	-10302.1944445	-10301.789797	-10301.894165	0.7510	-10301.885623	-	-
kn13_130 (from kn13_117)	-10302.1941742	-10301.789647	-10301.897079	0.7510	-10301.885419	-	-
**63b**							
kn13_164 (from kn13_120)	-10496.1040316	-10495.599685	-10495.719105	0.7549	-10495.706159	**-10495.8784268**	0.7599
kn13_120 (from kn13_112)	-10496.1035396	-10495.599321	-10495.718535	0.7547	-10495.706136	-10495.8780792	0.7596
kn13_121 (from kn13_113)	-10496.1025401	-10495.598467	-10495.718130	0.7552	-10495.705714	-10495.8765850	0.7607
kn13_145 (from kn13_120)	-10496.1016152	-10495.597429	-10495.719106	0.7553	-10495.704254	-10495.8752567	0.7606
kn13_144 (from kn13_121)	-10496.1013651	-10495.597292	-10495.719461	0.7548	-10495.704458	-10495.8749291	0.7598
kn13_142 (from	-10496.0998522	-10495.596659	-10495.713981	0.7549	-10495.701562	-10495.8749699	0.7600

kn13_119)							
kn13_143 (from kn13_118)	-10496.0998517	-10495.596661	-10495.713997	0.7549	-10495.701563	-	-
kn13_156 (from kn13_149)	-10496.0988511	-10495.594558	-10495.715437	0.7554	-10495.701063	-10495.8734056	0.7609
kn13_118 (from kn13_092)	-10496.0984540	-10495.594202	-10495.714893	0.7554	-10495.701062	-10495.8719415	0.7610
kn13_119 (from kn13_091)	-10496.0977645	-10495.593733	-10495.716732	0.7550	-10495.700862	-	-
kn13_122 (from kn13_025)	-10496.0954837	-10495.591607	-10495.715038	0.7569	-10495.698851	-	-
**64b**							
kn13_149 (from kn13_141)	-10496.1104851	-10495.605402	-10495.718202	0.7573	-10495.708489	**-10495.883292**	0.7650
kn13_123 (from kn13_028)	-10496.1104499	-10495.605608	-10495.719602	0.7576	-10495.708758	-10495.8827494	0.7655
kn13_153 (from kn13_141)	-10496.1103479	-10495.605398	-10495.718633	0.7572	-10495.708376	-10495.8830799	0.7648
kn13_141 (from kn13_123)	-10496.1096450	-10495.604413	-10495.717634	0.7577	-10495.707231	-10495.8817631	0.7660
kn13_154 (from kn13_123)	-10496.1096447	-10495.604415	-10495.717647	0.7577	-	-	-
kn13_160 (from kn13_149)	-10496.1082549	-10495.603098	-10495.716885	0.7573	-10495.706078	-10495.8802115	0.7647
kn13_140 (from kn13_124)	-10496.1069233	-10495.601355	-10495.714479	0.7572	-10495.704241	-10495.8788103	0.7646
kn13_150 (from	-10496.1069231	-10495.601353	-10495.714475	0.7572	-	-	-

kn13_140)							
kn13_124 (from kn13_022)	-10496.1067891	-10495.601331	-10495.714764	0.7573	-10495.703969	-10495.8782482	0.7647
kn13_139 (from kn13_125)	-10496.1032309	-10495.597836	-10495.712076	0.7569	-10495.700909	-	-
kn13_125 (from kn13_020)	-10496.1019341	-10495.596495	-10495.710191	0.7570	-10495.699517	-	-
**65b**							
kn13_155 (from kn13_137)	-10496.1245156	-10495.617317	-10495.733493	0.7510	-10495.721152	**-10495.9046242**	0.7520
kn13_159 (from kn13_155)	-10496.1243697	-10495.617169	-10495.733109	0.7510	-10495.720843	-10495.9043811	0.7520
kn13_158 (from kn13_155)	-10496.1242374	-10495.617005	-10495.733002	0.7510	-10495.720706	-10495.9042635	0.7520
kn13_137 (from kn13_128)	-10496.1230788	-10495.615860	-10495.731602	0.7510	-10495.720016	-10495.9021539	0.7520
kn13_148 (from kn13_137)	-10496.1230560	-10495.615927	-10495.733355	0.7510	-10495.720215	-10495.9021554	0.7520
kn13_152 (from kn13_137)	-10496.1229484	-10495.615888	-10495.733790	0.7510	-10495.720220	-10495.9018844	0.7520
kn13_128 (from kn13_089)	-10496.1229066	-10495.616064	-10495.734260	0.7510	-10495.720369	-10495.9025219	0.7520
kn13_151 (from kn13_128)	-10496.1226843	-10495.615846	-10495.733647	0.7510	-10495.720130	-10495.9022391	0.7520
kn13_147 (from kn13_128)	-10496.1226316	-10495.615786	-10495.734599	0.7510	-10495.720218	-10495.9022666	0.7520
kn13_138 (from	-10496.1225929	-10495.615740	-10495.734312	0.7510	-10495.720045	-	-

kn13_128)							
kn13_146 (from kn13_138)	-10496.1224513	-10495.615581	-10495.733904	0.7510	-10495.719817	-	-
**65c**							
kn13_163 (from kn13_162)	-10496.1075118	-10495.600663	-10495.716742	0.7512	-10495.704454	**-10495.8861128**	0.7524
kn13_162 (from kn13_149)	-10496.1059922	-10495.599165	-10495.718470	0.7512	-10495.703954	-10495.8853932	0.7524

Table S8c. Total energies and enthalpies for the systems shown in Figure S11 (in Hartree).

**system**	E_{tot} B3LYP-D3/ def2SVP	H_{298} B3LYP-D3/ def2SVP	G_{298}(qh) B3LYP-D3/ def2SVP	E_{tot} B2PLYP-D3/ def2TZVP// B3LYP-D3/ def2SVP	E_{tot} SMD(THF)/ B3LYP-D3/ def2SVP	ΔG_{298}(qh,1M) SMD(THF)/ B2PLYP-D3/ def2TZVPP// B3LYP-D3/ def2SVP
**55b**						
kn13_062	**-5248.9946701**	-5248.726218	-5248.793099	-5248.8822551	-5249.0156537	-5248.6986491
kn13_046	-5248.9943181	-5248.725837	-5248.792813	-5248.8824015	-5249.0153295	**-5248.6988893**
kn13_038	-5248.9910619	-5248.722834	-5248.789665	-5248.8791425	-5249.0140124	-5248.6976776
kn13_045	-5248.9907218	-5248.722392	-5248.788992	-5248.8781097	-5249.0137079	-5248.6963475
kn13_026	-5248.9905310	-5248.722443	-5248.789508	-5248.8787039	-5249.0132180	-5248.6973494
kn13_036	-5248.9887542	-5248.720742	-5248.788368	-5248.8781256	-5249.0117253	-5248.6976920
**1**						
kn13_005	**-193.8723732**	-193.774506	-193.805682	-193.9042207	-193.88523469	**-193.8474005**
kn13_002	-193.8723592	-193.774498	-193.803999	-193.9042284	-193.88523394	-193.8456964
**58b**						
kn13_077	**-5442.9199336**	-5442.549928	-5442.623731	-5442.8358706	-5442.9446857	-5442.5614016
kn13_078	-5442.9196890	-5442.549707	-5442.623611	-5442.8356435	-5442.9446937	-5442.5615517
kn13_075	-5442.9166914	-5442.546919	-5442.621728	-5442.8352407	-5442.9422601	-5442.5628275
kn13_076	-5442.9166862	-5442.547004	-5442.621921	-5442.8352062	-5442.9421142	**-5442.5628505**
kn13_030	-5442.9166539	-5442.546907	-5442.621707	-5442.8352015	-5442.9421679	-5442.5627501

**60b**						
kn13_111	**-10496.0997375**	-10495.593893	-10495.695279	-10495.8756169	-10496.1399818	-10495.5083842
kn13_110	-10496.0984333	-10495.592444	-10495.694273	-10495.8760979	-10496.1362879	-10495.5067737
kn13_114	-10496.0972934	-10495.591468	-10495.693770	-10495.8752033	-10496.1396495	**-10495.5110175**
kn13_102	-10496.0825707	-10495.576784	-10495.678756	-10495.8644445	-10496.1251774	-10495.5002180
kn13_097	-10496.0805365	-10495.575102	-10495.677244	-10495.8633397	-10496.1231295	-10495.4996217
kn13_101	-10496.0792827	-10495.573682	-10495.676712	-10495.8638378	-10496.1230554	-10495.5020213
kn13_098	-10496.0770840	-10495.571773	-10495.674846	-10495.8623509	-10496.1207865	-10495.5007969
kn13_099	-10496.0770169	-10495.571880	-10495.675082	-10495.8623260	-10496.1201571	-10495.5005128
kn13_100	-10496.0769800	-10495.571815	-10495.674981	-10495.8622572	-10496.1203103	-10495.5005700
**62b**						
kn13_134	**-10302.1998505**	-10301.793972	-10301.890463	-10301.9509943	-10302.2273434	-10301.6660812
kn13_165	-10302.1997837	-10301.794102	-10301.890619	**-10301.9510750**	-10302.2267326	-10301.6658407
kn13_131	-10302.1992152	-10301.793268	-10301.889679	-10301.9505531	-10302.2267171	-10301.6655003
kn13_116	-10302.1986391	-10301.793190	-10301.890398	-10301.9498011	-10302.2276115	-10301.6675139
kn13_132	-10302.1985914	-10301.793171	-10301.890577	-10301.9506078	-10302.2260649	-10301.6670484
kn13_133	-10302.1985855	-10301.793133	-10301.890295	-10301.9502018	-10302.2275403	**-10301.6678476**
kn13_157	-10302.1980690	-10301.792472	-10301.889617	-10301.9504959	-10302.2258338	-10301.6667902
kn13_129	-10302.1978781	-10301.792410	-10301.889707	-10301.9482851	-10302.2277115	-10301.6669289
kn13_161	-10302.1973917	-10301.792881	-10301.888878	-10301.9494329	-10302.2259573	-10301.6664663
kn13_136	-10302.1962515	-10301.790968	-10301.888491	-10301.9468741	-10302.2268094	-10301.6666530
kn13_135	-10302.1961726	-10301.790894	-10301.888277	-10301.9467181	-10302.2263533	-10301.6659847
**63b**						
kn13_164	**-10496.1040316**	-10495.599685	-10495.706159	-10495.8784268	-10496.1327708	-10495.5062749
kn13_120	-10496.1035396	-10495.599321	-10495.706136	-10495.8780792	-10496.1333920	**-10495.5075095**
kn13_121	-10496.1025401	-10495.598467	-10495.705714	-10495.8765850	-10496.1328949	-10495.5070952
kn13_144	-10496.1013651	-10495.597292	-10495.704458	-10495.8749291	-10496.1337843	-10495.5074227
kn13_142	-10496.0998522	-10495.596659	-10495.701562	-10495.8749699	-10496.1322032	-10495.5060122
kn13_156	-10496.0988511	-10495.594558	-10495.701063	-10495.8734056	-10496.1327334	-10495.5064813
kn13_145	-10496.1016152	-10495.597429	-10495.704254	-10495.8752567	-10496.1323346	-10495.5055964
kn13_118	-10496.0984540	-10495.594202	-10495.701062	-10495.8719415	-10496.1339144	-10495.5069914
**64b**						
kn13_149	**-10496.1104851**	-10495.605402	-10495.708489	**-10495.883292**	-10496.1490383	-10495.5168306
kn13_123	-10496.1104499	-10495.605608	-10495.708758	-10495.8827494	-10496.1503974	**-10495.5179865**
kn13_153	-10496.1103479	-10495.605398	-10495.708376	-10495.8830799	-10496.1499424	-10495.5176840
kn13_141	-10496.1096450	-10495.604413	-10495.707231	-10495.8817631	-10496.1479380	-10495.5146236
kn13_160	-10496.1082549	-10495.603098	-10495.706078	-10495.8802115	-10496.1483332	-10495.5150944

kn13_140	-10496.1069233	-10495.601355	-10495.704241	-10495.8788103	-10496.1463207	-10495.5125069
kn13_124	-10496.1067891	-10495.601331	-10495.703969	-10495.8782482	-10496.1466321	-10495.5122526
**65b**						
kn13_155	**-10496.1245156**	-10495.617317	-10495.721152	**-10495.9046242**	-10496.1544436	-10495.5281701
kn13_159	-10496.1243697	-10495.617169	-10495.720843	-10495.9043811	-10496.1543879	-10495.5278541
kn13_158	-10496.1242374	-10495.617005	-10495.720706	-10495.9042635	-10496.1538665	-10495.5273427
kn13_137	-10496.1230788	-10495.615860	-10495.720016	-10495.9021539	-10496.1562203	-10495.5292141
kn13_148	-10496.1230560	-10495.615927	-10495.720215	-10495.9021554	-10496.1561140	**-10495.5293539**
kn13_152	-10496.1229484	-10495.615888	-10495.720220	-10495.9018844	-10496.1559119	-10495.5291010
kn13_128	-10496.1229066	-10495.616064	-10495.720369	-10495.9025219	-10496.1548076	-10495.5288668
kn13_151	-10496.1226843	-10495.615846	-10495.720130	-10495.9022391	-10496.1543736	-10495.5283556
kn13_147	-10496.1226316	-10495.615786	-10495.720218	-10495.9022666	-10496.1548414	-10495.5290443
**65c**						
kn13_163	**-10496.1075118**	-10495.600663	-10495.704454	**-10495.8861128**	-10496.1510710	**-10495.5235957**
kn13_162	-10496.1059922	-10495.599165	-10495.703954	-10495.8853932	-10496.1486205	-10495.5229648

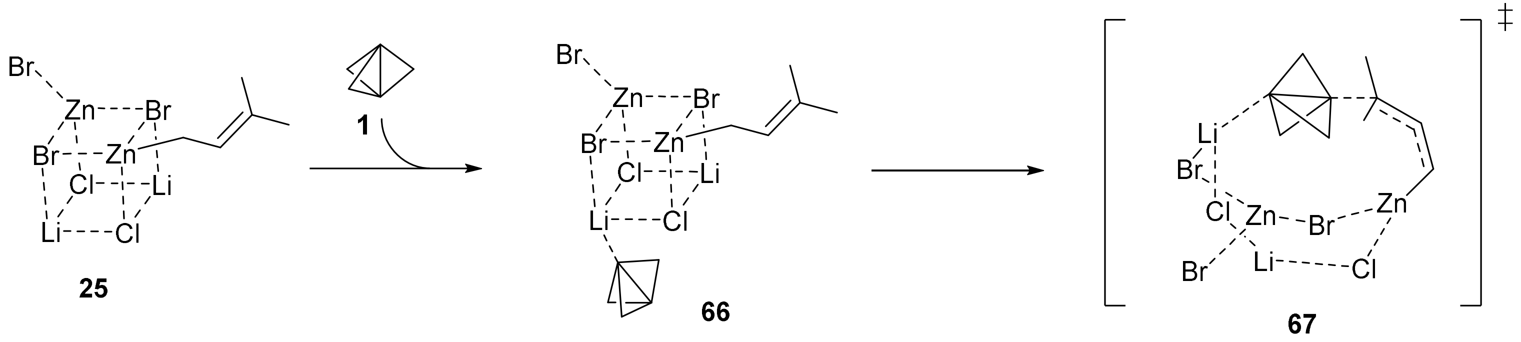

Figure S12. Coordination of [1.1.1]propellane on a lithium atom in cluster **25**, leading towards the transition state **67**.

Table S9a. Reaction energies for the systems shown in Figure S12 (in Hartree) and comparison to the system shown in Figure S1.

**system**	ΔH_{298} SMD(**THF**)/ B2PLYP-D3/ def2TZVPP// [kJ/mol]	ΔG_{298}(qh,1M) SMD(**THF**)/ B2PLYP-D3/ def2TZVPP// [kJ/mol]
**Step 1: 1 + 25 > 66**	-44.8	-2.0
**Step 2: 66 > 67**	+81.8	+137.6
**Figure S1:**		
**Step 1: 1 + 25 > 26a**	-47.3	-3.1
**Step 2: 26a > 27**	+47.2	+55.1

Table S9b. Total energies and enthalpies for the systems shown in Figure S12 (in Hartree).

**system**	E_{tot} B3LYP-D3/ def2SVP	H_{298} B3LYP-D3/ def2SVP	G_{298} B3LYP-D3/ def2SVP	$\langle S^2 \rangle$ B3LYP-D3/ def2SVP	G_{298}(qh) B3LYP-D3/ def2SVP	E_{tot} B2PLYP-D3/ def2TZVPP// B3LYP-D3/ def2SVP	$\langle S^2 \rangle$ B2PLYP-D3/ def2TZVPP// B3LYP-D3/ def2SVP
**1**							
kn13_005	**-193.87237324**	-193.774506	-193.803999	0.00	-193.803999	-193.9042284	0.00
kn13_002	-193.87235922	-193.774498	-193.805682	0.00	-193.805682	-193.9042207	0.00
**25**							
ks01_085	**-12411.5110482**	-12411.352496	-12411.432143	0.00	-12411.427599	-12411.0653400	0.00
ks01_084	-12411.5104746	-12411.351721	-12411.431056	0.00	-12411.426373	-12411.0641721	0.00
ks01_086	-12411.5094574	-12411.351089	-12411.432633	0.00	-12411.426892	-12411.0649448	0.00
ks01_006	-12411.5090628	-12411.350600	-12411.432506	0.00	-12411.426177	-12411.0654693	0.00
ks01_002	-12411.5089516	-12411.350515	-12411.432601	0.00	-12411.426285	-12411.0649217	0.00
ks01_005	-12411.5086638	-12411.350324	-12411.431980	0.00	-12411.426091		0.00
**66**							
ks03_002	-12605.4166014	-12605.157925	-12605.252647	0.00	-12605.244888	-12605.4474671	0.00
**67**							
ks03_011	-12605.3709199	-12605.114046	-12605.201209	0.00	-12605.196046	-12604.9528455	0.00

Table S9c. Total energies and enthalpies for the systems shown in Figure S12 (in Hartree).

**system**	E_{tot} B3LYP-D3/ def2SVP	H_{298} B3LYP-D3/ def2SVP	G_{298}(qh) B3LYP-D3/ def2SVP (qh)	E_{tot} B2PLYP-D3/ def2TZVPP// B3LYP-D3/ def2SVP	E_{tot} SMD(THF)/ B3LYP-D3/ def2SVP	G_{298}(qh,1M) SMD(THF)/ B2PLYP-D3/ def2TZVPP// B3LYP-D3/ def2SVP
**1**						
kn13_005	**-193.87237324**	-193.774506	-193.803999	-193.9042284	-193.88523394	-193.8456964
kn13_002	-193.87235922	-193.774498	-193.805682	-193.9042207	-193.88523469	**-193.8474005**
**25**						
ks01_085	**-12411.5110482**	-12411.352496	-12411.427599	-12411.0653400	-12411.5408086	**-12411.0086327**
ks01_084	-12411.5104746	-12411.351721	-12411.426373	-12411.0641721	-12411.5412006	-12411.0077780

ks01_086	-12411.5094574	-12411.351089	-12411.426892	-12411.0649448	-12411.5373398	-12411.0072433
ks01_006	-12411.5090628	-12411.350600	-12411.426177	-12411.0654693	-12411.5368335	-12411.0073357
ks01_002	-12411.5089516	-12411.350515	-12411.426285	-12411.0649217	-12411.5367619	-12411.0070469
ks01_005	-12411.5086638	-12411.350324	-12411.426091		-12411.5363737	
**66**						
ks03_002	-12605.4166014	-12605.157925	-12605.244888	-12605.0006701	-12605.4474671	-12604,8568039
**67**						
ks03_011	-12605.3709199	-12605.114046	-12605.196046	-12604.9528455	-12605.3995730	-12604,8036062

Analysis of the bonding situation and charge distribution in selected intermediates

Table S10. Wiberg Bond Index (WBI) and distances for reaction centers.

Structure	WBI of central C-C bond (B3LYP-D3/def2-SVP)	WBI of central C-C bond (B3LYP-D3/def2-TZVP)	WBI of C-M-bond (B3LYP-D3/def2-SVP)	WBI of C-M-bond (B3LYP-D3/def2-TZVP)	distance between bridgehead carbons (B3LYP-D3/def2-SVP) [pm]
**1**	0.7691	0.7906	-	-	156.3
**26a**	0.6557	0.6638	0.1291	0.1489	154.2
**27**	0.3393	0.3216	0.2194	0.2452	171.9
**28**	0.0579	0.0509	0.4775	0.5092	189.7
**66**	0.7260	0.7395	0.0374	0.0639	154.8

The coordination of [1.1.1]propellane (**1**) to zinc (**26a**) results in a reduction of the bonding order of the central bond indicated by a decrease of the Wiberg Bond Index (WBI). However, at the same time

the length of the bond decreases by 2 pm. This is in accordance with the results of Jemmis,[8] who observed similar results when investigating complexes of [1.1.1]propellane with electron-accepting halogen-bond donors. Overall, the removal of electron density from the mostly non-bonding HOMO seems to increase the strength of the central bond (for a detailed explanation see reference [8]).

As expected, the WBI of the central bond decreases significantly when moving to the transition state **27**. In the product cluster **28** there is almost no bonding interaction between the bridgehead carbons. The WBI of the carbon-zinc bond in the intermediate **26a** is approximately 4 times smaller than the one of the carbon-zinc bond in the product cluster **28**. Similar trends were observed for the coordination of [1.1.1]propellane to lithium (**66**), albeit on a significantly smaller scale.

The NBO presentation of the central bond in [1.1.1]propellane includes two partially occupied lone pairs at the bridgehead carbon atoms. A strong donor-acceptor interaction between the lone pair of one of the bridgehead carbons and a vacant orbital of the neighboring zinc in intermediate **26a** was confirmed by the NBO analysis:

```
113. (1.60735) LP ( 1) C 25          s( 53.79%)p 0.86( 46.12%)d 0.00( 0.08%)f 0.00( 0.01%)
141. (0.50812) LV ( 1)Zn  7          s( 99.67%)p 0.00( 0.17%)d 0.00( 0.16%)f 0.00( 0.00%)
144. (0.74142) LV ( 1) C 26          s( 9.16%)p 9.87( 90.35%)d 0.05( 0.47%)f 0.00( 0.02%)
```

[8] J. Joy, E. Akhil, E. D. Jemmis, *Phys. Chem. Chem. Phys.* **2018**, *20*, 25792-25798.

```
SECOND ORDER PERTURBATION THEORY ANALYSIS OF FOCK MATRIX IN NBO BASIS
     Threshold for printing:   0.50 kcal/mol
                                                                E(2)      E(NL)-E(L)        F(L,NL)
      Donor (L) NBO                Acceptor (NL) NBO      kcal/mol        a.u.               a.u.
 ======================================================================
      113. LP ( 1) C 25           141. LV ( 1)Zn  7        68.37         0.30              0.129
```

The method used for geometry optimizations (B3LYP-D3/def2-SVP) led to a minor underestimation of the central bond length in [1.1.1]-propellane. This underestimation remained mostly unaffected when moving to a bigger basis set (see table S11).

Table S11. Calculated length of the central bond in [1.1.1]propellane using different basis sets.

method	B3LYP-D3/def2-SVP	B3LYP-D3/def2-TZVPP	B3LYP-D3/def2-QZVPP	experimental value[9]
length of the central C-C bond in **1** [pm]	156.3	156.7	156.6	159.4

The Natural Population Analysis (NPA) was employed to examine the charge distribution in selected intermediates (Table S12).

[9] L. Hedberg, K. Hedberg, *J. Am. Chem. Soc.* **1985**, *107*, 7257-7260.

Table S12. Charges of fragments in [1.1.1]propellane in selected intermediates as determined by Natural Population Analysis.

Intermediate	charge of the bridgehead carbon next to zinc [a.u.]	charge of the bridgehead carbon opposite to zinc [a.u.]	charge of the bridge CH_2-groups (hydrogens summed into carbons) [a.u.]	total charge of the cage [a.u.]
**1**	−0.023	−0.023	+0.015	0
**26a**	−0.322	+0.086	+0.120/+0.119/+0.105	+0.108
**27**	−0.390	+0.019	+0.054/+0.067/+0.015	−0.233

The coordination of [1.1.1]propellane to the metal cluster in intermediate **26a** results in a localization of electron density at the bridgehead carbon adjacent to the zinc. This is compensated by a slight positive charge on the opposite bridgehead carbon and all of the CH_2-units, resulting in a total charge for the propellane unit of +0.108 e. In the transition state the negative charge on the bridgehead carbon next to zinc is even larger. The remaining cage still holds a positive charge. This is in contrast to previously reported reactions of anions with [1.1.1]propellane, where the delocalization of additional electron density onto the bridge carbons and the resulting electronic repulsion was found to be responsible for the relatively high activation barriers.[10]

[10] A. J. Sterling, A. B. Dürr, R. C. Smith, E. A. Anderson, F. Duarte, *Chem. Sci.* **2020**, *11*, 4895-4903.
